# Deleterious mutation/epimutation–selection balance with and without inbreeding: a population (epi)genetics model

**DOI:** 10.1093/genetics/iyae080

**Published:** 2024-05-11

**Authors:** Gregory Chernomas, Cortland K Griswold

**Affiliations:** Department of Integrative Biology, University of Guelph, Guelph, Ontario N1G 2W1, Canada; Department of Integrative Biology, University of Guelph, Guelph, Ontario N1G 2W1, Canada

**Keywords:** population genetics, epigenetics, epimutation, paramutation, inbreeding

## Abstract

Epigenetics in the form of DNA methylation and other processes is an established property of genotypes and a focus of empirical research. Yet, there remain fundamental gaps in the evolutionary theory of epigenetics. To support a comprehensive understanding of epigenetics, this paper investigates theoretically the combined effects of deleterious mutation and epimutation with and without inbreeding. Both spontaneous epimutation and paramutation are considered to cover a broader range of epigenetic phenomena. We find that inbreeding generally reduces the amount of segregating deleterious genetic and epigenetic variation at equilibrium, although interestingly inbreeding can also increase the amount of deleterious genetic or epigenetic variation. Furthermore, we also demonstrate that epimutation indirectly can cause increased or decreased deleterious genetic variation at equilibrium relative to classic expectations, which is particularly evident when paramutation is occurring. With the addition of deleterious epimutation, there may be significantly increased purging of deleterious variation in more inbred populations and a significantly increased amount of segregating deleterious variation in more outbred populations, with notable exceptions.

## Introduction

There is a growing literature whereby epigenetic phenomena are incorporated into theoretical evolutionary models. For epigenetic inheritance to be of evolutionary significance, it has to be heritable, variable in populations, and to have nonnegligible effects on fitness (Bonduriansky and Day 2018). Several previous works have presented results pertaining to the adaptive significance of epialleles and conditions that would favor epigenetic variation as a source of adaptive variation (Klironomos *et al*. 2013; Uller *et al*. 2015; Kronholm and Collins 2016; Greenspoon and Spencer 2018; Smithson *et al*. 2019). This sort of work is of great interest; however, a key additional consideration is the deleterious effects of epimutation, and with explicit predictions on the resulting amounts of segregating deleterious epigenetic variation in populations. Some studies have accounted for the possibility that epialleles can be adaptive and/or deleterious (Stenøien and Pedersen 2005; Geoghegan and Spencer 2012, 2013a, 2013b, 2013c; Furrow 2014; Webster 2024); see discussion for details. Yet, the consequences of inbreeding on deleterious epigenetic variation and its interaction with other processes, such as mutation and paramutation, have not been systematically studied, theoretically.

The effects of inbreeding and breeding systems in general may be of special relevance to *K. Plantae*, as they are a clade more likely to house heritable epigenetic variation (Charlesworth *et al*. 2017), and where breeding systems are quite variable (Charlesworth 2006). Several epialleles have been identified in plants (Jacobsen and Meyerowitz 1997; Cubas *et al*. 1999; Soppe *et al*. 2000; Manning *et al*. 2006; Martin *et al*. 2009; Miura *et al*. 2009; Silveira *et al*. 2013; Quadrana *et al*. 2014). These studies indicate a range of observations of the fitness effects of epialleles, including effects that are likely to be deleterious and others which may be a source of deleterious or adaptive fitness variation. Nevertheless, more direct fitness measurements of naturally occurring, heritable, nonneutral epialleles are needed to characterize the distribution of fitness effects of epigenetic variation in natural populations.

Two specific forms of epimutation are spontaneous epimutations, which are analogous to random mutations, and paramutation, which is analogous to gene conversion (Geoghegan and Spencer 2013c). Specifically, spontaneous epimutation refers to a random change in the state of an epigenetic modification, such as the methylation status of a particular nucleotide (van der Graaf *et al*. 2015). Paramutation is the conversion of the gene expression state of one allele to an identical or similar expression state as its homologous allele through an in trans interaction (Geoghegan and Spencer 2013c). Of note, paramutation can occur at higher rates than rates of mutation and spontaneous epimutation (Springer and McGinnis 2015; Hollick 2017).

Paramutation (or similar phenomena) has been discovered in a diverse set of plant species including maize (Brink 1956; Coe 1966; Pilu *et al*. 2009; Sidorenko *et al*. 2024), Arabidopsis (Luff *et al*. 1999), petunia (van Houwelingen *et al*. 1999), and tomato (Gouil and Baulcombe 2018). These examples relate to endogenous genes (Sidorenko *et al*. 2024). There have also been discoveries of paramutation occurring at transgenic loci (Mittelsten Scheid *et al*. 2003; Khaitová *et al*. 2011; Xue *et al*. 2012; Gao and Zhao 2013; Bente *et al*. 2021). This highlights the potential importance of paramutation in relation to genetically modified crops. In addition, paramutation-like processes have been observed to occur in mice (Rassoulzadegan *et al*. 2006) and Drosophila (Capovilla *et al*. 2017; Dorador *et al*. 2022), suggesting broader evolutionary implications for paramutation across distantly related clades. Other studies also suggest paramutation may be a more common phenomenon than currently realized and a cause of offspring having distinct gene expression patterns than either parent at a given locus (He *et al*. 2010; Groszmann *et al*. 2011; Barber *et al*. 2012; Shen *et al*. 2012; Li *et al*. 2013; Springer and McGinnis 2015). This paper focuses on the potential deleterious effects of paramutation through conversion of a wild-type gene expression pattern to an aberrant expression state (i.e. a deleterious epiallele).

With regard to breeding systems, one expects spontaneous epimutations to behave in a similar manner to classic work on deleterious mutation–selection balance (Crow and Kimura 2010), whereby inbreeding should increase the effectiveness of selection and reduce the amount of segregating deleterious epigenetic variation imposed by spontaneous epimutations. Inbreeding is also expected to reduce the amount of deleterious epigenetic variation imposed by the process of paramutation, but due to an additional reason (Springer and McGinnis 2015). Paramutation depends on an (epi)heterozygous pairing of a paramutagenic (epi)allele and a paramutable allele, and therefore, inbreeding (which decreases heterozygosity) is expected to reduce the effective paramutation rate and decrease the amount of deleterious epialleles generated through this process (Springer and McGinnis 2015).

A key aspect of epimutation in terms of distinctiveness relative to mutation is an increased rate of occurrence (van der Graaf *et al*. 2015) and highly variable reversion rates. With regard to reversion rates, epialleles can be highly unstable, reverting within a few generations (Bell and Hellmann 2019), or can stably segregate for many generations (Vastenhouw *et al*. 2006; Johannes *et al*. 2009; Hollick 2017). Both possibilities are considered in this paper. We also consider the degree of susceptibility to epimutation for different genetic variants (Springer and McGinnis 2015; Johannes and Schmitz 2019), such that epimutation may have an asymmetric fitness effect on a given genetic variant vs another.

To our knowledge, this paper is the first to provide a systematic theoretical treatment of deleterious epimutation–selection balance (both spontaneous mutation and epimutation) with the inclusion of inbreeding. As part of this systematic treatment, we incorporate paramutation to broaden the results for different epigenetic processes that may occur in nature. The structure of the paper is such that first a model of deleterious epimutation–selection balance is derived and then analytical results of equilibria states are presented, with complementary numerical analysis.

## Overview of genetic and epigenetic states

The model covers a general context where there is a most fit wild-type allele, with, for example, an optimized level of gene expression. In line with classical work (Crow and Kimura 2010), mutation occurs, acting as a deleterious force. In addition, epimutation in the form of both spontaneous epimutation and paramutation acts against selection for the wild-type allele as it modifies the expression state of the wild-type allele, converting it to an epiallelic variant. The epiallele is assumed to have a lower fitness than the wild-type allele, in line with the general expectation that epimutation is typically neutral or deleterious (Charlesworth *et al*. 2017).

All cases analyzed have two allelic states and one epiallelic state of the wild-type allele. This situation is expected if one allele has a sequence difference that makes it susceptible to epimutation, while the other allele is not epimutable (Springer and McGinnis 2015; Quadrana and Colot 2016; Johannes and Schmitz 2019). Specifically, for example, this may occur if allelic variants differ in the number and/or placement of cytosine residues in their sequences allowing for potentially different methylation patterns. Another possibility is if genetic variants differ as a result of a transposon or repetitive element mutation (Ong-Abdullah *et al*. 2015; Springer and McGinnis 2015; Quadrana and Colot 2016; Johannes and Schmitz 2019), such that epimutation only occurs on the genetic variant that possesses the transposon/repetitive element. An alternative context for the model is whereby spontaneous epimutation occurs at both alleles, but has a neutral fitness effect on one allele and that same allele is not paramutagenic (Springer and McGinnis 2015). In line with this, it is assumed the deleterious allele is not directly affected by the epimutation process, but the wild-type allele is directly impacted across all cases analyzed.

## Model

Consider a population that is infinite in size, diploid, and sexually reproducing. At a locus, there are three (epi)allelic states, including a wild-type allele (A), a deleterious allele (a), and an epiallelic variant of the wild-type allele (B). Table 1 presents all variables and parameters used in the model with symbols indicated.

**Table 1.** Names and symbols for variables and parameters.

Variable/Parameter	Symbol
Frequency of allele (A)	*p_A_*
Frequency of allele (a)	*p_a_*
Frequency of epiallele (B)	*p_B_*
Relative fitness of genotype i	$w_{i}$
Inbreeding coefficient	*f*
Paramutation rate	*m*
Forward spontaneous epimutation rate	*t*_1_
Reverse spontaneous epimutation rate	*t*_2_
Mutation rate of A ⇒ a	*u*
Mutation rate of a ⇒ A	*z*
Mutation rate of B ⇒ a	$u^{\prime }$

All variables and parameters are restricted to be between 0 and 1 in magnitude.

Forward spontaneous epimutation of the wild-type allele to the epiallele occurs at rate *t*_1_ and the reverse occurs at rate *t*_2_.


$$A\overset{t_{1}}{\to }\;B$$

$$A\overset{t_{2}}{\leftarrow }\;B$$

Paramutation involving only (epi)genotype A/B occurs at rate *m* (Geoghegan and Spencer 2013c):


$$A/B\overset{m}{\to }\;B/B$$

**Fig. 1.**
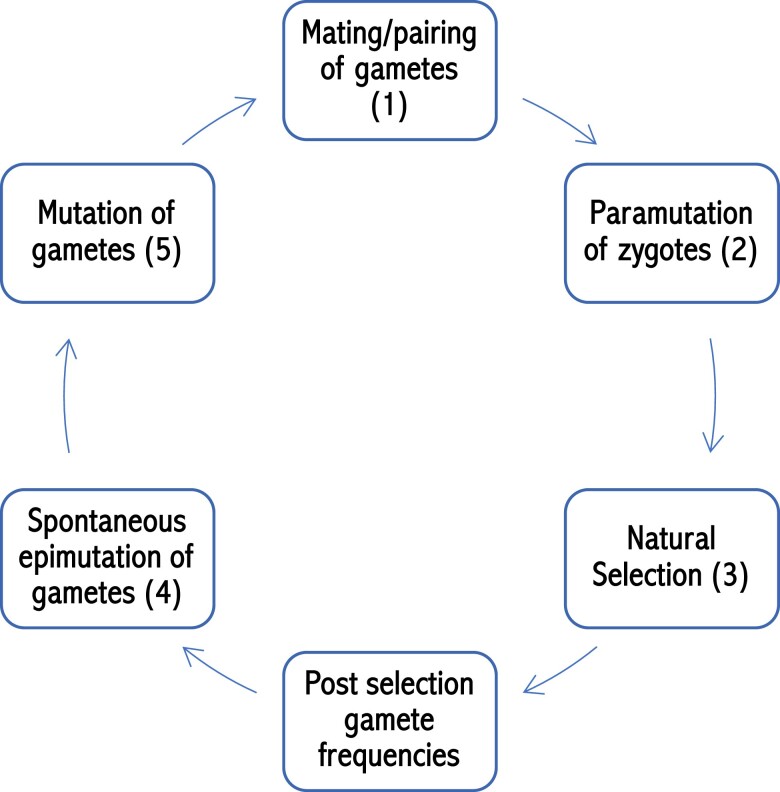
Life cycle diagram outlining the sequence of events for the model. Beginning at the top, the initial event is mating/pairing of gametes to generate zygotes. Zygotes then undergo paramutation at rate “*m*” if the genotype is A/B, but no other (epi)genotypes are affected. Selection then occurs based on assumed genotype fitness, to generate the postselection gamete frequencies. This is followed by forward and reverse spontaneous epimutation of gametes. Mutation of gametes is the final event, which generates the next generation of gamete frequencies prior to the life cycle repeating.

The assumed sequence of events is mating, paramutation, natural selection [normalized by mean fitness ($\bar{w}$)], spontaneous epimutation, and mutation (see Fig. 1). This is consistent with classic work and assumptions whereby selection occurs after mating, and mutation is assumed to occur after selection (Crow and Kimura 2010). Following this order of events, the epiallele homozygote genotype frequency (*p_B, B_)* is


$$p_{B,B}^{\left(1\right)}=p_{B}^{2}(1-f)+p_{B}f\;(\mathrm{following}\;\mathrm{mating})$$

$$p_{B,B}^{\left(2\right)}=p_{B,B}^{\left(1\right)}+p_{A,B}^{\left(1\right)}m\;\;\;\;(\mathrm{following}\;\mathrm{paramutation})$$

$$p_{B,B}^{\left(3\right)}=\frac{w_{4\;}p_{B,B}^{\left(2\right)}}{\bar{w}}\;(\mathrm{postselection})$$

Mating, the possibility of paramutation, and then selection were modeled for all genotypes, such that


$$p_{B}^{\left(3\right)}=\;p_{\mathrm{BB}}^{\left(3\right)}+\frac{1}{2}p_{\mathrm{AB}}^{\left(3\right)}+\frac{1}{2}p_{\mathrm{Ba}}^{\left(3\right)}\;\mathrm{and}\;p_{A}^{\left(3\right)}=\;p_{\mathrm{AA}}^{\left(3\right)}+\;\frac{1}{2}p_{\mathrm{AB}}^{\left(3\right)}+\;\frac{1}{2}p_{\mathrm{Aa}}^{\left(3\right)}.$$

Spontaneous epimutation occurs next, which for the epiallele results in a frequency of


$$p_{B}^{\left(4\right)}=(1-t_{2})\;p_{B}^{\left(3\right)}+t_{1}p_{A}^{\left(3\right)}\;\;\;$$

Mutation occurs as the final event in the life cycle. We assume mutation has the same effect on the wild-type allele and epiallele. In the case of the epiallele, mutation results in conversion to the “a” state, such that


$$p_{B}^{\left(5\right)}=p_{B}^{\left(4\right)}(1-u^{\prime })=\;{\;p}_{B}^{\prime }$$

The derivation of the full system of recursion equations consisting of the wild-type allele, epiallele, and deleterious allele frequencies, respectively, is provided in Supplementary File S1.

Relative fitnesses for all genotypes are given in Table 2. Due to the constraint *p_A_* + *p_B_* + *p_a_* = 1, the number of dimensions is reduced to 2, and only the deleterious allele and epiallele were directly tracked.

**Table 2.** Relative fitness symbols for all (epi)genotypes.

Allele/epiallele	A	B	a
A	$w_{1}$	$w_{2}$	$w_{3}$
B	$w_{2}$	$w_{4}$	$w_{5}$
a	$w_{3}$	$w_{5}$	$w_{6}$

Analytical techniques used to obtain equilibria and perform local stability analysis are presented in the Appendix, and detailed calculations are provided in the Supplementary Material (Supplementary File S2). For analytical tractability and based on reasonable biological assumptions, rates of forward spontaneous epimutation and mutation were assumed to be small in magnitude [i.e. *u*, *u*′, *z*, *t*_1_ ∼ O(ζ)] for all cases. This assumption allowed for Taylor's approximations in determining equilibria and stability terms (see Appendices A1 and A2 for details).

Despite the approximations, the eigenvalues were generally still complicated making analytical local stability interpretations difficult. Accordingly, numerical assessments of the approximated eigenvalues and complimentary numerical simulations of the recursion equations were performed. We find only one equilibrium is simultaneously biologically valid and locally stable for a given area of parameter space for cases A–D.

## Results

For the results of the main text, incomplete dominance on fitness was assumed (Table 3) for all cases (A–E). Supplementary File S3 examines a complete dominance on fitness scenario where the wild-type allele is completely dominant when present in an (epi)genotype and the epiallele is completely dominant when paired with the deleterious allele. All cases in the main text and Supplementary File S3 allow for two scenarios. One parameter scenario assumes all mutation and spontaneous epimutation rates are small in magnitude [∼ O(ζ)] (see Appendix A1 for details), whereas the second scenario assumes the reverse spontaneous epimutation is not small in magnitude (*t_2_ >> ζ*). This accounts for both relatively stable and unstable epialleles, both of which are known to occur (Vastenhouw *et al*. 2006; Hollick 2017; Bell and Hellmann 2019; Johannes and Schmitz 2019).

**Table 3.** Assumed fitness of (epi)genotypes for cases A–D.

Allele/epiallele	A	B	a
A	1	$1-h_{2}s_{2}$	1−hs
B	$1-h_{2}s_{2}$	$1-s_{2}$	$1-h_{2}s_{2}-hs+s_{3}$
a	1−hs	$1-h_{2}s_{2}-hs+s_{3}$	1 – *s*

### Cases A and B with no paramutation (*m* = 0)

#### Case A: random mating (*f* = 0)

This case assumes random mating and no paramutation to give baseline predictions of the allele and epiallele frequencies at equilibrium. There is only one biologically valid and stable equilibrium, which to first order is


$${\hat{\;p}}_{a}\;\approx \;\frac{u}{hs}+O[\zeta^{2}]$$

$${\hat{\;p}}_{B}\;\approx \;\frac{t_{1}}{h_{2}s_{2}}+O[\zeta^{2}]$$

(1) $${\hat{\;p}}_{A}\;\approx \;1-\frac{u}{hs}-\frac{t_{1}}{h_{2}s_{2}}+O[\zeta^{2}]$$

This equilibrium assumes selection is a stronger force than mutation and spontaneous epimutation causing the wild-type allele to be at a high frequency with the deleterious allele and epiallele at a low frequency at equilibrium. The deleterious allele equilibrium frequency is at the classic expectation of mutation–selection balance with incomplete dominance (Crow and Kimura 2010). The equilibrium frequency of the epiallele has the same form as the deleterious genetic allele. As selection against the epiallele increases or its dominance over the wild-type allele increases, it reaches a lower equilibrium frequency.

Overall, the process of spontaneous epimutation in addition to mutation results in increased segregating deleterious variation at equilibrium, relative to the classic two-allele case (Crow and Kimura 2010). Since, generally *t_1_* > *u* (van der Graaf *et al*. 2015), the deleterious epiallele may be at a higher frequency than the deleterious allele, causing an even larger amount of segregating deleterious variation.

Next, we allow the reverse spontaneous epimutation rate to not be small in magnitude (i.e. *t_2_ >> ζ*), assuming the forward rate and mutation rates are still relatively small in magnitude (i.e. u, z, u′, *t_1_* ∼ O(ζ)). This corresponds to when the epiallele is unstable. We find one stable equilibrium occurs (eq. 2) that has a similar form to the previous context (eq. 1).


$${\hat{\;p}}_{a}\;\approx \;\frac{u}{hs}+O[\zeta^{2}]$$

$${\hat{\;p}}_{B}\;\approx \;\frac{t_{1}}{h_{2}s_{2}(1-t_{2})+t_{2}}+O[\zeta^{2}]$$

(2) $${\hat{\;p}}_{A}\;\approx \;1-\frac{u}{hs}-\frac{t_{1}}{h_{2}s_{2}(1-t_{2})+t_{2}}+O[\zeta^{2}]$$

Unlike eq. 1, *t*_2_ now affects the wild-type and epiallele frequencies. A higher reverse rate lowers the equilibrium frequency of the epiallele and increases the wild-type frequency compared to eq. 1. As *t*_2_ approaches one (complete epiallele resetting), the epiallele equilibrium frequency is completely determined by the forward spontaneous epimutation rate *t*_1_ (see Table 4 for a summary of the case A equilibria). Of note, Stenøien and Pedersen (2005) gave explicit analytical results of deleterious epimutation–selection balance with incomplete dominance for diploids, with similar results to eq. 2 (see discussion for details).

**Table 4.** Summary of case A equilibria [no paramutation (*m* = 0) and no inbreeding (*f* = 0)].

Equilibria conditions	${\hat{p}}_{a}$	${\hat{p}}_{B}$	${\hat{p}}_{A}$	Eq. #
*w*_AA_ > *w*_Aa_ > *w*_aa_, *w*_AA_ > *w*_AB_ > *w*_BB_,	very low	low	high	1
*u* << *hs*,				
*t*_1_ << *h*_2_*s*_2_				
*Epiallele reversion rate is not* small (i.e. *t_2_ >> ζ*)				
*w*_AA_ > *w*_Aa_ > *w*_aa_, *w*_AA_ > *w*_AB_ > *w*_BB_,	very low	very low–low	high–very high	2
*u* << *hs*,				
*t*_1_ << *h*_2_*s*_2_, *t*_2_				

Separately, we also analyze a distinct fitness scenario where there is complete dominance of the wild-type allele and complete dominance of the epiallele when paired with deleterious allele. A major result of interest is a significantly lower frequency of the deleterious allele at equilibrium compared with the classic two allele expectations with complete dominance. This result likely stems from a higher frequency of the epiallele, due to masking by the wild-type allele, which then allows for more pairings of the epiallele with the deleterious allele, lowering its marginal fitness (see Supplementary File S3, case A for details).

#### Case B: with inbreeding (*f* > 0)

Here, we examine the joint effects of incomplete dominance and inbreeding on the equilibrium frequencies of the alleles and epiallele. In line with classic expectations, inbreeding acts to lower ${\hat{\;p}}_{a}\;$ and ${\hat{\;p}}_{B}$ through increased efficiency of selection with a corresponding increase in ${\hat{\;p}}_{A}$. The equilibrium approximated to first order is


$${\hat{\;p}}_{a}\;\approx \;\frac{u}{\;fs+hs(1-f)}+O[\zeta^{2}]$$

$${\hat{\;p}}_{B}\;\approx \;\frac{t_{1}}{\;fs_{2}+h_{2}s_{2}(1-f)}+O[\zeta^{2}]$$

(3) $${\hat{\;p}}_{A}\;\approx \;1-\frac{u}{\;fs+hs(1-f)}-\frac{t_{1}}{\;fs_{2}+h_{2}s_{2}(1-f)}+O[\zeta^{2}]$$

Overall, with inbreeding (*f* > 0), the amount of segregating deleterious genetic and epigenetic variation at equilibrium is reduced in comparison with when random mating occurs (*f* = 0).

Next, we consider the parameter scenario when the reverse spontaneous epimutation rate is not small (*t_2_ >> ζ*), resulting in the equilibrium


$${\hat{\;p}}_{a}\approx \;\frac{u}{\;fs+hs(1-f)}+O[\zeta^{2}]$$

$${\hat{\;p}}_{B}\;\approx \;\frac{t_{1}}{(\;fs_{2}+h_{2}s_{2}(1-f))(1-t_{2})+t_{2}}+O[\zeta^{2}]$$

(4) $${\hat{\;p}}_{A}\;\approx \;1-\frac{t_{1}}{(\;fs_{2}+h_{2}s_{2}(1-f))(1-t_{2})+t_{2}}-\frac{u}{\;fs+hs(1-f)}+O[\zeta^{2}]$$

Similar to eq. 2, as the reverse spontaneous epimutation rate increases, the effects of incomplete dominance, selection, and, additionally, inbreeding have less of an impact on the equilibrium frequency of the epiallele (see Table 5 for a summary of the case B equilibria).

**Table 5.** Summary of case B equilibria [no paramutation (*m* = 0) and with inbreeding (*f* > 0)].

Equilibria conditions	${\hat{p}}_{a}$	${\hat{p}}_{B}$	${\hat{p}}_{A}$	Eq. #
*w*_AA_ > *w*_Aa_ > *w*_aa_, *w*_AA_ > *w*_AB_ > *w*_BB_,	very low	low	high	3
*u* << *h*s, *f*s,				
*t*_1_ << *h*_2_s_2_, *f*s_2_				
*Epiallele reversion rate is not* small (i.e. *t_2_ >> ζ*)				
*w*_AA_ > *w*_Aa_ > *w*_aa_, *w*_AA_ > *w*_AB_ > *w*_BB_,	very low	very low–low	high–very high	4
*u* << *h*s, *f*s,				
*t*_1_ << *t*_2_, *h*_2_s_2_, *f*s_2_				

Separately, we analyze the context of complete dominance in fitness. Overall, the results for the equilibria are similar as with incomplete dominance, with marginal increases in the epiallele and deleterious allele due to increased masking by the wild-type allele (see Supplementary File S3, case B for details).

### Cases C and D with paramutation (*m* > 0)

#### Case C: random mating (*f* = 0)

Paramutation acts as an additional force that can generate epialleles. Importantly, the rate of paramutation can be high compared to genetic mutation and forward spontaneous epimutation (Hollick 2017). Overall, we identify five distinct equilibria when paramutation is incorporated (see Table 6 for a summary). The equilibria occur within mutually exclusive parameter constraints based on biological validity and/or local stability conditions. The distinct parameter conditions for the equilibria are largely a result of the relative fitness differences of the deleterious (epi)genotypes and the relative strength of paramutation compared to selection. Two equilibria are analytically tractable and are presented first; following this, numerical results for the other three equilibria are discussed.

**Table 6.** Summary of case C equilibria [with paramutation, (*m* > 0) and no inbreeding (*f* = 0)].

Equilibria conditions	${\hat{p}}_{a}$	${\hat{p}}_{B}$	${\hat{p}}_{A}$	Eq. #
*w*_AA_ > *w*_Aa_ > *w*_aa_, *w*_AA_ > *w*_AB_ > *w*_BB_, *u* << *w*_AA_ − *w*_Aa_, *t*_1_ << *w*_AA_ − *w*_AB_ + *m*(*w*_AB_ − 2*w*_BB_), *m*(2*w*_BB_ − *w*_AB_) < (*w*_AA_ − *w*_AB)_ (equilibrium 1)	very low	low	high	5
*w*_AA_ > *w*_Aa_, *w*_AB_ > *w*_BB_ > *w*_aa_, *w*_Ba_ and 2*w*_BB_ > *w*_AB_, *u*, *u*′, *z* < *t*_1_, *t*_2_, (*w*_AB_ − *w*_BB_)/*w*_AB_ > *m* > (*w*_AA_ − *w*_AB_)/(2*w*_BB_ − *w*_AB_) (equilibrium 2)	low	low–high	low–high	AN (e.g. see Supplementary File S2, fig. C1)
*w*_AA_ > *w*_Aa_, *w*_AB_ > *w*_Ba_ > *w*_aa,_ *w*_BB_, *u*, *u*′, *z* < *t*_1_, *t*_2_, *m* ≳ *w*_AA_ − *w*_BB_, *w*_AA_ − *w*_aa_ (equilibrium 3)	moderate–high	moderate–high	low	AN (e.g. see Fig. 2)
*w*_AA_ > *w*_Aa_ > *w*_aa_, *w*_AA_ > *w*_AB_ > *w*_BB_ > *w*_Ba_, *w*_AB_(1 − *m*), and 2*w*_BB_ > *w*_AB_, *u*′ << *w*_BB_ − *w*_Ba_, *t*_2_ << *w*_BB_ − *w*_AB_(1 − *m*), m > (*w*_AB_ − *w*_BB_)/*w*_AB_ (equilibrium 4)	very low	high	low	6
*w*_AA_ > *w*_Aa_ > *w*_aa_ > *w*_Ba,_ *w*_BB_, *u*, *u*′, *z* < *t*_1_, *t*_2_, *m* ≳ *w*_AA_ − *w*_BB_, *w*_AA_ − *w*_aa_ (equilibrium 5)	moderate–high	low–moderate	low	AN (e.g. see Fig. 3)

AN, analyzed numerically, approximation assumptions: *u*, *u*′, *z*, *t*_1_, *t*_2_ ∼ O[*ζ*].

Interestingly, two analytically tractable and biologically relevant equilibria occur roughly depending on whether paramutation is higher or lower than the strength of selection against the epiallele. Specifically, when *m*(2*w*_BB_ − *w*_AB_) < (*w*_AA_ − *w*_AB)_, the following equilibrium arises where the wild-type allele is at a high frequency (approximated to first order):


$${\hat{\;p}}_{a}\;\approx \;\frac{u}{1-w_{\mathrm{Aa}}}+O[\zeta^{2}]$$

$${\hat{\;p}}_{B}\;\approx \;\frac{t_{1}}{1-w_{\mathrm{AB}}+m(w_{\mathrm{AB}}-2w_{\mathrm{BB}})}+O[\zeta^{2}]$$

(5) $${\hat{\;p}}_{A}\;\approx \;1-\frac{u}{1-w_{\mathrm{Aa}}}-\frac{t_{1}}{1-w_{\mathrm{AB}}+m(w_{\mathrm{AB}}-2w_{\mathrm{BB}})}+O[\zeta^{2}]$$

where $w_{\mathrm{Aa}}=1-\mathrm{hs},\;w_{\mathrm{AB}}=1-h_{2}s_{2},\;w_{\mathrm{BB}}=1-s_{2}$.

The deleterious allele is at classic two-allele expectations of mutation–selection balance with incomplete dominance (Crow and Kimura 2010). Paramutation affects ${\hat{\;p}}_{B}$ and ${\hat{\;p}}_{A}$ through the term *m*(*w*_AB_ − 2*w*_BB_). In particular, if *w*_AB_ < 2*w*_BB_, paramutation increases ${\hat{\;p}}_{B}$ above the case when *m* = 0. In contrast, if *w*_AB_ > 2*w*_BB_, then paramutation *decreases*  ${\hat{\;p}}_{B}$.

A second equilibrium arises when paramutation is of sufficient magnitude to cause fixation of the epiallele which is balanced by reverse spontaneous epimutation to maintain the wild-type allele as well as mutation of the epiallele to maintain the deleterious allele. The equilibrium approximated to first order is


$${\hat{\;p}}_{a}\;\approx \;\frac{u^{\prime }w_{\mathrm{BB}}}{w_{\mathrm{BB}}-w_{\mathrm{Ba}}}+O[\zeta^{2}]$$

$${\hat{\;p}}_{B}\;\approx \;1-\frac{t_{2}w_{\mathrm{BB}}}{w_{\mathrm{BB}}-w_{\mathrm{AB}}(1-m)}-\frac{u^{\prime }w_{\mathrm{BB}}}{w_{\mathrm{BB}}-w_{\mathrm{Ba}}}\;+O[\zeta^{2}]$$

(6) $${\hat{\;p}}_{A}\;\;\approx \;\frac{t_{2}w_{\mathrm{BB}}}{w_{\mathrm{BB}}-w_{\mathrm{AB}}(1-m)}\;+\;O[\zeta^{2}]$$

where $w_{\mathrm{Ba}}=1-\mathrm{hs}-h_{2}s_{2}+s_{3},\;w_{\mathrm{AB}}=1-h_{2}s_{2},\;w_{\mathrm{BB}}=1-s_{2}$.

For the equilibrium to be biologically valid, two general constraints must hold: *w*_BB_ > *w*_Ba_ and *w*_BB._ > *w*_AB_(1 − *m*). The first constraint indicates the B/B (epi)genotype is required to have a higher relative fitness than the B/a (epi)genotype. Regarding the second constraint, the model assumes $w_{\mathrm{BB}}<w_{\mathrm{AB}}$, and then, paramutation effectively reduces the contribution of the relative fitness of the A/B (epi)genotype such that it can be less than the relative fitness of the epiallele homozygote (B/B).

${\hat{\;p}}_{A}$ and ${\hat{\;p}}_{a}$ are distinct from the classic two-allele case of mutation–selection balance because the epiallele is at the highest frequency. ${\hat{\;p}}_{A}$ and ${\hat{\;p}}_{a}$ largely depend on the difference in relative fitness of the epiallele homozygote and the relevant heterozygous pairing with the epiallele.

When the rate of paramutation is sufficiently high, such that the equilibrium corresponding to eq. 5 does not arise biologically [i.e. *m*(2*w*_BB_ − *w*_AB_) > (*w*_AA_ − *w*_AB_)], but where paramutation is still weaker than the strength of selection against the epiallele (specifically *m* < (*w*_AB_ − *w*_BB_)/*w*_AB_), an equilibrium arises where either the wild-type or epiallele can be at high frequency, or with moderate frequencies of both. Due to analytical complexity, this equilibrium was analyzed numerically. Based on the parameter values examined, the fitness constraint for this equilibrium is *w*_BB_ > *w*_aa_, *w*_Ba_. Paramutation directly increases the epiallele frequency at this equilibrium (see Supplementary File S2, pg. 23, figure C1), but we did not identify any notable parameter effects or interactions for this equilibrium. Based on numerical assessments, a separate equilibrium appears to occur within the fitness constraint: *w*_Ba_ > *w*_aa_, *w*_BB_ which implies a compensatory fitness effect when the deleterious allele and epiallele are joined in the same genotype. Of note, the fitness constraints for this equilibrium are mutually exclusive to the previous two equilibria. In terms of biological context, this could arise, for example, if a regulatory mutation and an epimutation both result in a deleterious change in gene expression relative to the wild-type pattern but where one increases gene expression and the other decreases gene expression. This could result in a partial recovery in fitness of the wild-type expression state. Also, in addition, paramutation is near or greater than the strength of selection for this equilibrium.

**Fig. 2.**
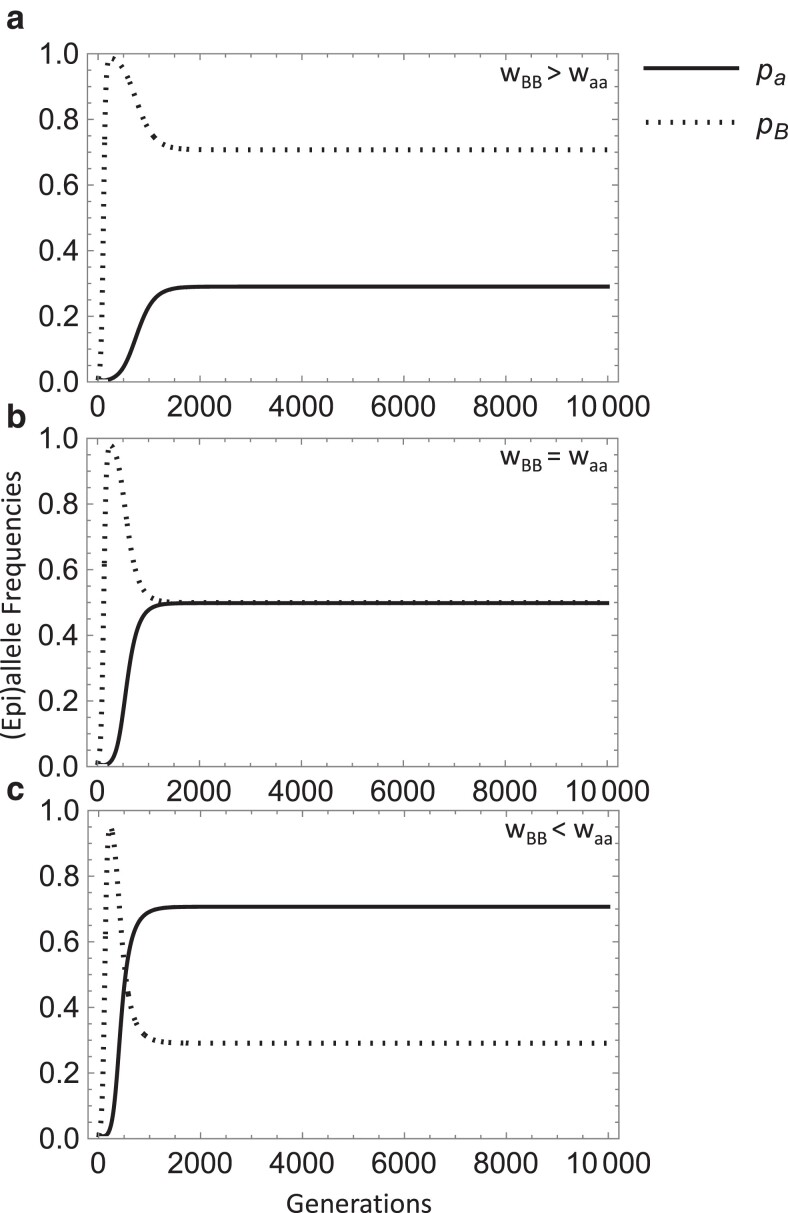
Plots of numerical simulations of the recursion equations for the deleterious allele frequency (solid line) and epiallele frequency (dotted line) across time. An equilibrium occurs approximately under parameter conditions: *m* > *s*, *s*_2_ and *w*_B, a_ > *w*_a, a_, *w*_B, B_. The fitness relationships presented are: *w*_B, B_ > *w*_a, a_ (top panel a, *s* = 0.02 and *s*_2_ = 0.01), *w*_B, B_ = *w*_a, a_ (middle panel b, *s* = *s*_2_ = 0.02), and *w*_B, B_ < *w*_a, a_ (bottom panel c, *s* = 0.01 and *s*_2_ = 0.02). Other parameters were held constant: *s*_3_ = 0.012, *t*_1_ = 10^−5^, *t*_2_ = 10^−4^, *u* = 10^−9^, *z* = 10^−9^, *u*′ = 10^−9^, *h* = 0.5, *h*_2_ = 0.5, *m* = 0.05.

Figure 2 demonstrates a parameter scenario for this equilibrium with the observed impact on *p*_a_ and *p*_B_ as the relative values of *w*_BB_ and *w*_aa_ vary. It appears that paramutation causes the epiallele to nearly replace the wild-type allele initially (see peaks in Fig. 2) and later maintains the wild-type allele at low frequency. Interestingly, as paramutation maintains the wild-type allele at low frequency, a balance is reached at equilibrium between the deleterious allele and epiallele based on the difference between *w*_aa_ and *w*_BB_, which determines whether one will be higher or lower, or if they reach near equal frequencies.

**Fig. 3.**
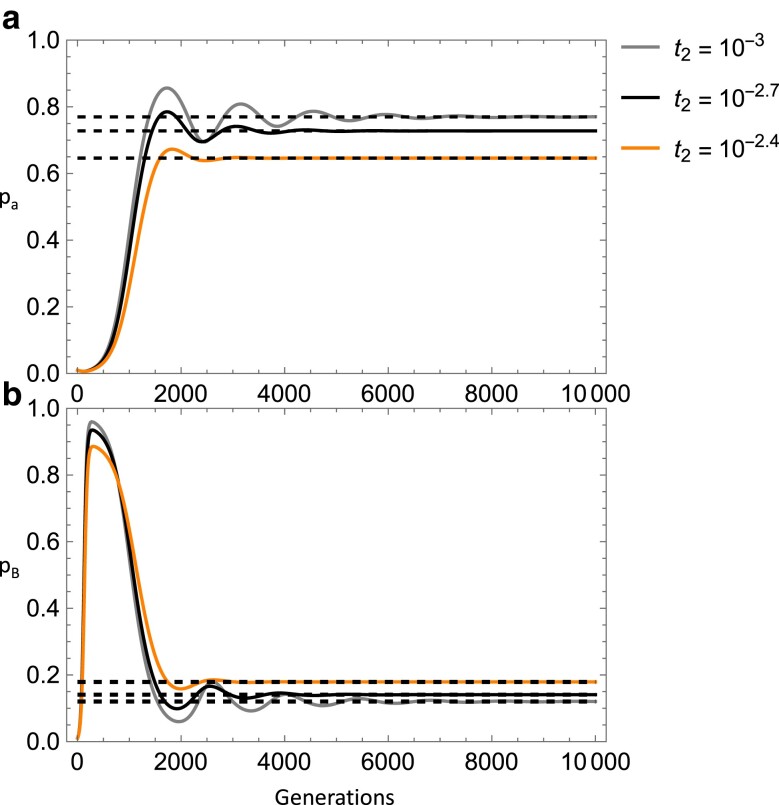
Top panel (a) shows deleterious allele frequency and bottom panel (b) shows epiallele frequency across time as the reverse spontaneous epimutation rate increases going from the gray line (*t*_2_ = 10^−3^), to the black line (*t*_2_ = 10^−2.7^), to the orange line (*t*_2_ = 10^−2.4^) with equilibria indicated by dashed lines. Other parameters were held constant: *s* = 0.01, *s*_2_ = 0.02, *s*_3_ = 0.001, *t*_1_ = 10^−5^, *u* = 10^−9^, *z* = 10^−9^, *u*′ = 10^−9^, *h* = 0.5, *h*_2_ = 0.5, *m* = 0.05. Note, the orange line asymptotes at the lowest frequency in panel a) and highest frequency in panel b).

Another equilibrium arises when *w*_aa_ > *w*_BB_ > *w*_Ba_ and when paramutation is near or stronger than the strength of selection against the deleterious allele or when *w*_aa_ > *w*_Ba_ > *w*_BB_ and when paramutation is stronger than selection (for the parameter range examined). Focusing on the second fitness condition, here the a/a genotype is of higher fitness than the B/a and B/B (epi)genotypes. Interestingly, *p*_a_ and *p*_B_ cycle as the equilibrium is approached (Fig. 3). Reverse spontaneous epimutation has a clear effect of *reducing* the deleterious allele equilibrium frequency and *increasing* the epiallele equilibrium frequency while also reducing the number of generations cycling occurs (for the given parameter values). This is a counterintuitive result, but may be due to the reverse epimutation rate reducing the effective paramutation rate which appears to *increase* the deleterious allele and *decrease* the epiallele frequency for this equilibrium (not shown). A possible reason for this effect of paramutation is that it simultaneously reduces the wild-type allele and increases the epiallele frequency in terms of its direct affect but also inputs epialleles into a relatively deleterious (epi)genotype (for this equilibriums' fitness constraints). Selection then causes the epiallele to reach an overall lower equilibrium frequency and a higher frequency of the deleterious allele. Since reverse spontaneous epimutation reduces the effective paramutation rate, this partly mitigates this effect.

Next, relaxing the assumption that the reverse spontaneous epimutation rate is small in magnitude (i.e. *t_2_ >> ζ*), an equilibrium occurs analogous to that of eq. 5:


$${\hat{\;p}}_{a}\;\approx \;\frac{u}{1-w_{\mathrm{Aa}}}+O[\zeta^{2}]$$

$${\hat{\;p}}_{B}\;\approx \;\frac{t_{1}}{1-(1-t_{2})(w_{\mathrm{AB}}-m(w_{\mathrm{AB}}-2w_{\mathrm{BB}}))}+O[\zeta^{2}]$$

(7) $${\hat{\;p}}_{A}\;\approx \;1-\frac{u}{1-w_{\mathrm{Aa}}}-\frac{t_{1}}{1-(1-t_{2})(w_{\mathrm{AB}}-m(w_{\mathrm{AB}}-2w_{\mathrm{BB}}))}+O[\zeta^{2}]$$

where $w_{\mathrm{Aa}}=1-\mathrm{hs},\;w_{\mathrm{AB}}=1-h_{2}s_{2},\;w_{\mathrm{BB}}=1-s_{2}$.

**Fig. 4.**
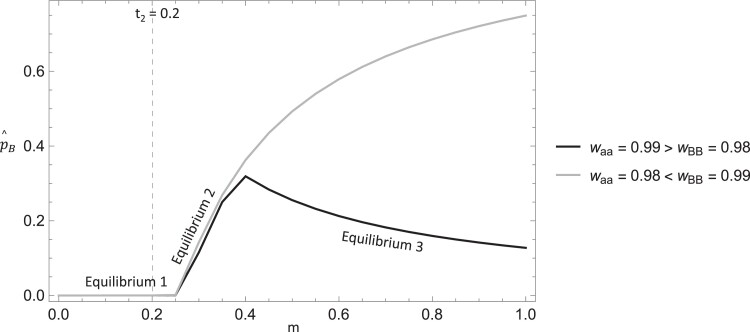
Plot of the epiallele equilibrium frequency against the paramutation rate (*m*) with different relative fitness relationships of the deleterious allele and epiallele. This demonstrates the interaction of parameters that allow for three distinct equilibria to arise biologically. The dashed line indicates the value of the epiallele reversion rate (*t*_2_) showing that paramutation must be greater than this rate for two of the equilibria to arise. Other parameters values were: *w*_AA_ = 1, *w*_Aa_ = 0.995 (gray line), *w*_Aa_ = 0.99 (black line), *w*_AB_ = 0.99 (gray line), *w*_AB_ = 0.995 (black line), *w*_Ba_ = 0.985, *t*_1_ = 10^−5^, *t*_2_ = 0.2, *u* = 10^−9^, *z* = 10^−9^, *u*′ = 10^−9^.

The reverse spontaneous epimutation rate directly reduces the impact of paramutation and tends to decrease ${\hat{\;p}}_{B}$ with a corresponding increase in ${\hat{\;p}}_{A}$. As a compliment to the analytical results in eq. 7, we plot the equilibrium frequencies of the epiallele as the paramutation rate varies and for different relationships of *w*_BB_ and *w*_aa_ (see Fig. 4). When 0 < *m* < 0.25, an equilibrium arises where the wild type is at a high frequency (eq. 7), and whether *w*_BB_ or *w*_aa_ is greater does not affect which equilibrium occurs biologically. Once paramutation is more than marginally greater than the epiallele reversion rate (i.e. *m* > 0.25, when *t*_2_ = 0.20, see Fig. 4), a second distinct equilibrium arises where the epiallele ranges from a moderate to high frequency as paramutation increases, at the expense of the wild-type allele. At this equilibrium, *w*_BB_ and *w*_aa_ do not effect which equilibrium arises until *m* becomes sufficiently greater than *t*_2_. This critical point depends on the magnitudes of the other parameters, but once it occurs, a distinct equilibrium can arise. Once this point is reached, if *w*_aa_ < *w*_BB_, the second equilibrium continues to hold, and the epiallele frequency increases with the paramutation rate, with lower frequencies of the wild-type allele and deleterious allele at equilibrium. Alternatively, if *w*_aa_ > *w*_BB_, the epiallele remains at a moderate equilibrium frequency and *decreases* as the paramutation rate increases (as observed in Fig. 4). In contrast, the deleterious allele is also at a moderate frequency at this equilibrium and increases with the paramutation rate (not shown). Overall, these results demonstrate three equilibria arise for case C (when *t_2_*  *>>*  *ζ*) and the parameter conditions that allow for each.

As a separate analysis, we obtained numerical equilibria results for case C in the context of complete dominance in fitness. A key result is that when the wild-type allele is at a high frequency, the deleterious allele is significantly below classic two-allele expectations with complete dominance. This likely occurs as result of the epiallele reaching a higher frequency, through masking by the wild-type allele, causing more pairings of the epiallele and deleterious allele, reducing the marginal fitness of the deleterious allele (see Supplementary File S3, case C for details and presentation of the other equilibria).

#### Case D: with inbreeding (*f* > 0)

This case offers insights into interactions between inbreeding and paramutation on deleterious epimutation–selection balance. Analogous to the previous case with random mating (*f* = 0), we identify five distinct equilibria (see Table 7 for a summary). The equilibria occur in mutually exclusive parameter conditions, such that more than one biologically valid and locally stable equilibrium is not observed for a given parameter scenario. In addition to the key impacts of relative fitness conditions and the paramutation rate on equilibrium outcomes, the levels of inbreeding also play a major role in determining which equilibrium arises. Two equilibria are analytically tractable and are presented first. Then, numerical results are presented and discussed for the other three equilibria.

**Table 7.** Summary of case D equilibria [with paramutation (*m* > 0) and inbreeding (*f* > 0)].

Equilibria conditions	${\hat{p}}_{a}$	${\hat{p}}_{B}$	${\hat{p}}_{A}$	Eq. #
*w*_AA_ > *w*_Aa_ > *w*_aa_, *w*_AA_ > *w*_AB_ > *w*_BB_, *u* << *w*_AA_ − *fw*_aa_ − (1 − *f*) *w*_Aa_, *t*_1_ << *w*_AA_ − *fw*_BB_ − (1 − *f*)(*w*_AB_ − *m*(*w*_AB_ − 2*w*_BB_)), *m*(1 − *f*) (2*w*_BB_ − *w*_AB_) < (*w*_AA_ − *fw*_BB_ − (1 − *f*)*w*_AB_) (equilibrium 1)	very low	low	high	8
*w*_AA_ > *w*_Aa_, *w*_AB_ > *w*_BB_ > *w*_aa_, *w*_Ba_, and 2*w*_BB_ > *w*_AB_, *u*, *u*′, *z* < *t*_1_, *t*_2_, (*f* − *w*_BB_ − (1 − *f*) *w*_AB_)/*w*_AB_ > *m* (1 − *f*) > (*w*_AA_ − *fw*_BB_ − (1 − *f*)*w*_AB_)/(2*w*_BB_ − *w*_AB_) (equilibrium 2)	low	moderate–high	moderate–high	AN (e.g. see Supplementary File S2, Fig. D1)
*w*_AA_ > *w*_Aa_, *w*_AB_ > *w*_Ba_ > *w*_aa,_ *w*_BB,_ *u*, *u*′, *z* < *t*_1_, *t*_2_, *m* (1 − *f*) ≳ *w*_AA_ − *w*_BB_, *w*_AA_ − *w*_aa_ (equilibrium 3)	low–high	low–high	low–moderate	AN (e.g. see Fig. 5)
*w*_AA_ > *w*_Aa_, *w*_AB_ > *w*_BB_ > *w*_aa_, *w*_Ba_, and 2*w*_BB_ > *w*_AB_, *u*′ << *w*_BB_ − *w*_Ba_ − *f*(*w*_aa_ − *w*_Ba_), *t*_2_ << *w*_BB_ − *f* − (1 − *f*)(1 − *m*) *w*_AB_, *m*(1 − *f*) > (*f* + (1 − *f*) *w*_AB_ − *w*_BB_)/*w*_AB_ (equilibrium 4)	very low–low	high	low	9
*w*_AA_ > *w*_Aa_, *w*_AB_ > *w*_aa_ > *w*_Ba_ > *w*_BB_, *u*, *u*′, *z* < *t*_1_, *t*_2_, *m* (1 − *f*) ≳ *w*_AA_ − *w*_BB_, *w*_AA_ − *w*_aa_ (equilibrium 5)	moderate–high	low–moderate	low–moderate	AN (e.g. see Fig. 6)

AN, analyzed numerically, approximation assumptions: *u*, *u*′, *z*, *t*_1_, *t*_2_ ∼ O[*ζ*].

The first equilibrium (eq. 8) arises when the combination of selection and inbreeding is approximately of greater magnitude than the effective paramutation rate.


$${\hat{\;p}}_{a}\;\approx \;\frac{u}{1-fw_{\mathrm{aa}}-(1-f)w_{\mathrm{Aa}}}+O[\zeta^{2}]$$

$${\hat{\;p}}_{B}\;\approx \;\frac{t_{1}}{1-fw_{B,B}-(1-f)(w_{A,B}-m(w_{A,B}-2w_{B,B}))}+O[\zeta^{2}]$$

(8) $${\hat{\;p}}_{A}\;\approx \;1-\frac{u}{1-fw_{\mathrm{aa}}-(1-f)w_{\mathrm{Aa}}}-\frac{t_{1}}{1-fw_{B,B}-(1-f)(w_{A,B}-m(w_{A,B}-2w_{B,B}))}+O[\zeta^{2}]$$

where $w_{\mathrm{aa}}=1-s,\;w_{\mathrm{Aa}}=1-\mathrm{hs},\;w_{\mathrm{AB}}=1-h_{2}s_{2},\;\;w_{\mathrm{BB}}=1-s_{2}$.

The key distinction relative to when *f* = 0 is the twofold effect of inbreeding, increasing the efficiency of selection through increased homozygosity and reducing the effective paramutation rate. Overall, at this equilibrium, inbreeding decreases the amount of segregating deleterious genetic and epigenetic variation through more effective selection and lowering the effective paramutation rate.

A second equilibrium occurs when the paramutation rate is high enough to outweigh the combined effects of selection and inbreeding, causing the epiallele to reach a high frequency. The equilibrium approximated to the first order is


$${\hat{\;p}}_{a}\;\approx \;\frac{u^{\prime }w_{\mathrm{BB}}}{w_{B,B}-w_{B,a}+f(w_{B,a}-w_{a,a})}\;+O[\zeta^{2}]$$

$${\hat{\;p}}_{B}\;\approx \;1-w_{\mathrm{BB}}\left(\frac{t_{2}}{w_{\mathrm{BB}}-f-(1-f)(1-m)w_{\mathrm{AB}}}+\frac{u^{\prime }}{w_{B,B}-w_{B,a}+f(w_{B,a}-w_{a,a})}\right)+O[\zeta^{2}]$$

(9) $${\hat{\;p}}_{A}\;\approx \;\frac{t_{2}w_{\mathrm{BB}}}{w_{\mathrm{BB}}-f-(1-f)(1-m)w_{\mathrm{AB}}}\;+O[\zeta^{2}]$$

where


$$w_{\mathrm{aa}}=1-s,\;w_{\mathrm{Aa}}=1-\mathrm{hs},\;w_{\mathrm{AB}}=1-h_{2}s_{2},\;\;w_{\mathrm{BB}}=1-s_{2},\;w_{\mathrm{Ba}}=1-hs-h_{2}s_{2}+s_{3}.$$

Two constraints must be met for the equilibrium to arise in a biologically valid form. One constraint that must hold is $w_{\mathrm{BB}}>w_{\mathrm{Ba}}+f(w_{\mathrm{aa}}-w_{\mathrm{Ba}})$. This constraint will always hold if $w_{\mathrm{BB}}$ > $w_{\mathrm{aa}}$, $w_{\mathrm{Ba}}$. Interestingly, when $w_{\mathrm{aa}}$ > $w_{\mathrm{Ba}}$, inbreeding *increases*  ${\hat{\;p}}_{a}$ and vice versa when $w_{\mathrm{Ba}}$ > $w_{\mathrm{aa}}$. Therefore, inbreeding can either increase or decrease the amount of deleterious genetic variation at this equilibrium whereby ${\hat{\;p}}_{a}$ may be higher or lower relative to when *f* = 0. The second constraint $w_{\mathrm{BB}}>f+(1-f)(1-m)w_{\mathrm{AB}}\;$ can never hold if $w_{\mathrm{BB}}\;$ <f. Intuitively, this implies high levels of inbreeding can prevent the epiallele from being at the highest frequency. When this equilibrium (eq. 9) is biologically valid, inbreeding always causes a net reduction in the amount of segregating deleterious epigenetic variation relative to when *f* = 0.

Based on numerical assessments, another equilibrium occurs with the parameter scenario: (*f* − *w*_BB_ − (1 − *f*) *w*_AB_)/*w*_AB_ > *m* (1 − *f*) (2*w*_BB_ − *w*_AB_) > (*w*_AA_ − *fw*_BB_ – (1 − *f*)*w*_AB_) and *w*_BB_ > *w*_aa_, *w*_Ba_. The wild-type allele and epiallele were at moderate frequencies and the deleterious allele at low frequency. The key result is that inbreeding reduces the effective paramutation rate which decreases the epiallele frequency at equilibrium and increases the wild-type allele (see Supplementary File S2, pg. 39, figure D1).

**Fig. 5.**
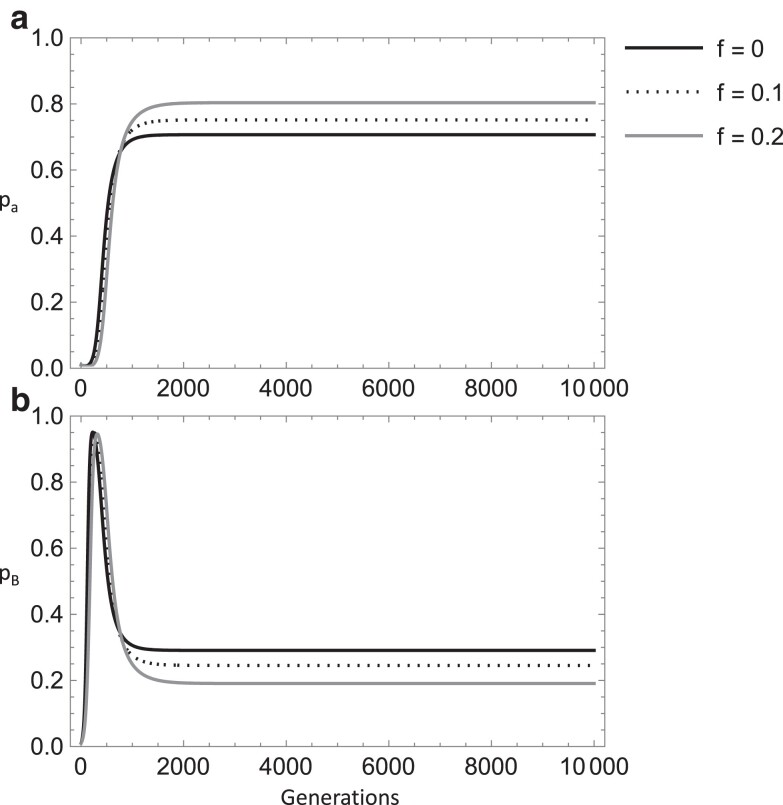
Plots of numerical simulations of the recursion equations for the deleterious allele frequency (top panel a) and epiallele frequency (bottom panel b) across time. The inbreeding coefficient increases going from the solid line (*f* = 0), to the dotted line (*f* = 0.1), to the dashed line (*f* = 0.2). Other parameters were held constant: *s* = 0.01, *s*_2_ = 0.02, *s*_3_ = 0.012, *t*_1_ = 10^−5^, *t*_2_ = 10^−4^, *u* = 10^−9^, *z* = 10^−9^, *u*′ = 10^−9^, *h* = 0.5, *h*_2_ = 0.5, *m* = 0.05.

A separate equilibrium arises under the mutually exclusive fitness constraints *w*_Ba_ > *w*_aa_, *w*_BB_. As the case with random mating (case C), a prerequisite is that there is a compensatory fitness interaction between the epiallele and the deleterious allele in the same (epi)genotype. With the fitness condition: *w*_Ba_ > *w*_aa_ > *w*_BB_, for the parameter values examined, inbreeding *increases* the deleterious allele equilibrium frequency and decreases that of the epiallele (Fig. 5).

Alternatively, when *w*_Ba_ > *w*_BB_ > *w*_aa_, inbreeding *increases* the epiallele frequency at equilibrium and decreases that of the deleterious allele (data not shown). Analogous to the random mating case, paramutation appears to maintain the wild-type allele at a low frequency, and this allows for an equilibrium to unfold based on the relative fitness of the epiallele and deleterious allele homozygotes. As inbreeding increases, the B/a (epi)genotype is reduced with corresponding increases in homozygosity. In turn, the difference in the (epi)homozygote relative fitnesses plays a larger role in determining the equilibrium frequencies. Of note, if inbreeding levels are sufficiently high, this equilibrium no longer occurs, most likely due to a reduction in the effective paramutation rate allowing for selection to cause the wild-type allele to reach a high frequency (i.e. eq. 8).

**Fig. 6.**
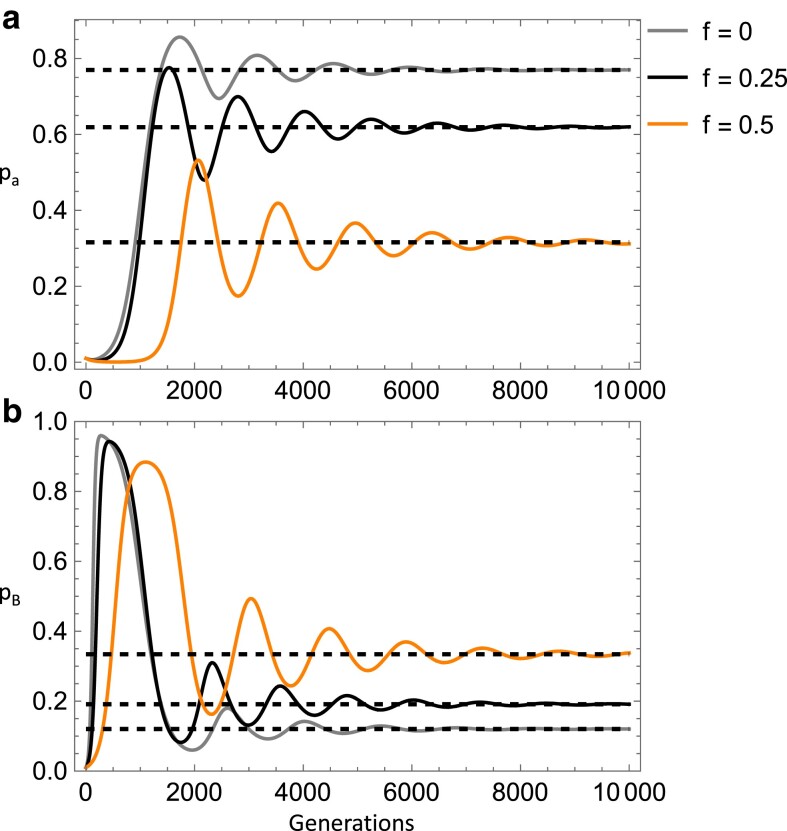
Plots of numerical simulations of the recursion equations for the deleterious allele frequency (top panel a) and the epiallele frequency (bottom panel b) across time, demonstrating cycling dynamics. The inbreeding coefficient increases going from the gray line (*f* = 0), to the black line (*f* = 0.25), to the orange line (*f* = 0.5), with equilibria indicated by the dashed lines. Other parameters were held constant: *s* = 0.01, *s*_2_ = 0.02, *s*_3_ = 0.001, *t*_1_ = 10^−5^, *t*_2_ = 10^−3^, *u* = 10^−9^, *z* = 10^−9^, *u*′ = 10^−9^, *h* = 0.5, *h*_2_ = 0.5, *m* = 0.05. Note, the orange line asymptotes at the lowest frequency in panel a) and highest frequency in panel b).

Analogous to the previous case (without inbreeding, *f* = 0), cyclical behavior of *p*_a_ and *p*_B_ occurs under the fitness condition: *w*_aa_ > *w*_Ba_ > *w*_BB_ and when paramutation was stronger than selection (*m* > *s*, *s*_2_). We see the impacts of inbreeding on this dynamic in Fig. 6. Inbreeding lowers the deleterious allele equilibrium frequency and *increases* that of the epiallele and also increases the length of time that cycling occurs. Importantly, once inbreeding levels are adequately high, the efficiency of selection outweighs the effective paramutation rate and the wild-type allele reaches a high frequency with more marginal amounts of the deleterious variants (as observed in eq. 8).

Relaxing the assumption of a small reverse spontaneous epimutation rate (i.e. *t_2_ >> ζ*), an equilibrium arises (eq. 10), where the wild-type allele is at a high frequency.


$${\hat{\;p}}_{a}\;\approx \;\frac{u}{1-fw_{\mathrm{aa}}-(1-f)w_{\mathrm{Aa}}}+O[\zeta^{2}]$$

$${\hat{\;p}}_{B}\;\approx \;\frac{t_{1}}{1-(1-t_{2})(\;fw_{BB}+(1-f)(w_{AB}-m(w_{AB}-2w_{BB})))}+O[\zeta^{2}]$$

(10) $${\hat{\;p}}_{A}\;\approx \;1-\frac{u}{1-fw_{\mathrm{aa}}-(1-f)w_{\mathrm{Aa}}}-\frac{t_{1}}{1-(1-t_{2})(\;fw_{BB}+(1-f)(w_{AB}-m(w_{AB}-2w_{BB})))}+O[\zeta^{2}]$$

The key distinction from eq. 8 is the presence of $t_{2}\;$ in the denominators of ${\hat{\;p}}_{B}$ and ${\hat{\;p}}_{A}$. Interestingly, as the epiallele reversion rate increases, both inbreeding and paramutation have less of an impact on the equilibrium frequencies of the wild-type allele and epiallele.

**Fig. 7.**
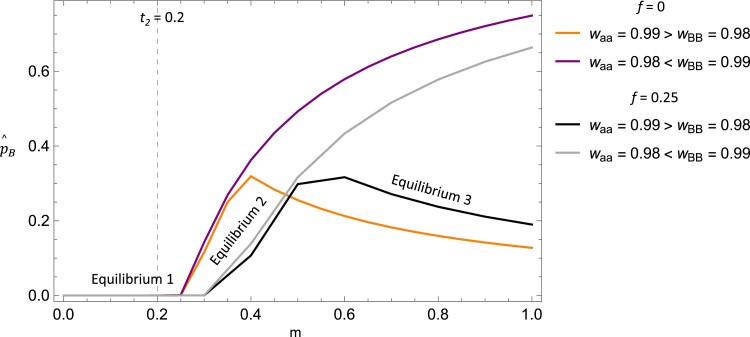
Plot of the epiallele equilibrium frequency against the paramutation rate (*m*) with inbreeding and with different relative fitness relationships of the deleterious allele and epiallele. This figure shows how inbreeding reduces the effective paramutation rate and decreases the parameter space where the epiallele is at an appreciable frequency. This is observed by comparing the *f* = 0 cases to the *f* = 0.25 cases. The dashed line indicates the value of the epiallele reversion rate (*t*_2_) showing that the effective paramutation rate must be greater than this rate for two of the equilibria to arise. Other parameters were held constant: *w*_AA_ = 1, *w*_Aa_ = 0.995 (gray line), *w*_Aa_ = 0.99 (black line), *w*_AB_ = 0.99 (gray line), *w*_AB_ = 0.995 (black line), *w*_Ba_ = 0.985, *t*_1_ = 10^−5^, *t*_2_ = 0.2, *u* = 10^−9^, *z* = 10^−9^, *u*′ = 10^−9^.

As a compliment to the analytical results, we plot the equilibrium frequencies of the epiallele as the paramutation rate varies and for different relationships of *w*_BB_ and *w*_aa_ (see Fig. 7). Overall, the results are very similar to case C (when *t_2_ >> ζ*, Fig. 4). Three equilibria arise, depending on the strength of *m* relative to *t_2_* and the relative magnitudes of *w*_BB_ and *w*_aa_, and the general patterns mirror that of the random mating context (as shown in Fig. 7). The key distinction is that with inbreeding incorporated, there is increased parameter space where the equilibrium arises with the wild-type allele at a high frequency (eq. 10) and a corresponding decrease in the parameter space for the equilibria where the epiallele is at a moderate or high frequency (as demonstrated in Fig. 7). This is a direct result of inbreeding decreasing the effective paramutation rate requiring *m* to be larger in magnitude for these equilibria to arise.

Separately, with complete dominance in fitness, the analytical equilibria results generally mirror the incomplete dominance context in terms of equilibria constraints and properties. One key exception is that only four equilibria arise biologically instead of five. Specifically, the equilibrium dependent on the fitness constraint *w*_Ba_ > *w*_BB_, *w*_aa_ cannot arise with complete dominance in fitness (see Supplementary File S3, case D, for the analytical and numerical results).

#### Case E: the deleterious allele is paramutagenic

Following work by Geoghegan and Spencer (2013c), we now allow for a second paramutation rate caused by the presence of the deleterious allele. This property of paramutation has been observed in mice (Rassoulzadegan *et al*. 2006). Specifically, genotype A/a is now converted to (epi)genotype B/a at rate *m*_2_.


$$A/a\overset{m_{2}}{\to }\;B/a$$

We label the previous paramutation rate *m*_1_, and otherwise, the previous assumptions and parameters are kept the same as case D. An equilibrium arises (eq. 11), and overall, this equilibrium has similar properties as eq. 8 (case D) with some key distinctions (see Table 8 for a summary of the case E equilibria).


$${\hat{\;p}}_{a}\;\approx \;\frac{u}{1-fw_{a,a}-(1-f)(w_{A,a}-m_{2}(w_{A,a}-w_{B,a}))}+O[\zeta^{2}]$$

$${\hat{\;p}}_{B}\;\approx \;\frac{t_{1}}{1-fw_{B,B}-(1-f)(w_{A,B}-m_{1}(w_{A,B}-2w_{B,B}))}+\frac{(1-f)um_{2}w_{B,a}}{\left(1-fw_{a,a}-(1-f)(w_{A,a}-m_{2}(w_{A,a}-w_{B,a})))(1-fw_{B,B}-(1-f)(w_{A,B}-m_{1}(w_{A,B}-2w_{B,B}))\right)})+O[\zeta^{2}]$$

(11) $${\hat{\;p}}_{A}\;\approx \;1-\frac{u}{1-fw_{a,a}-(1-f)(w_{A,a}-m_{2}(w_{A,a}-w_{B,a}))}-\frac{t_{1}}{1-fw_{B,B}-(1-f)(w_{A,B}-m_{1}(w_{A,B}-2w_{B,B}))}-\frac{(1-f)um_{2}w_{B,a}}{\left(1-fw_{a,a}-(1-f)(w_{A,a}-m_{2}(w_{A,a}-w_{B,a})))(1-fw_{B,B}-(1-f)(w_{A,B}-m_{1}(w_{A,B}-2w_{B,B}))\right)})+O[\zeta^{2}]$$

**Table 8.** Summary of case E equilibria [with paramutation (*m*_1_, *m*_2_ > 0) and inbreeding (*f* > 0)].

Equilibria conditions	${\hat{p}}_{a}$	${\hat{p}}_{B}$	${\hat{p}}_{A}$	Eq. #
*w*_AA_ > *w*_Aa_ > *w*_aa_, *w*_AA_ > *w*_AB_ > *w*_BB_, *u* << 1 − *fw*_aa_ − (1 − *f*)(*w*_Aa_ − m_2_(*w*_Aa_ − *w*_Ba_)), *t*_1_ << 1 − *fw*_BB_ − (1 − *f*)(*w*_AB_ − m_1_(*w*_AB_ − 2*w*_BB_)), *m*_1_(1 − *f*) (2*w*_BB_ − *w*_AB_) < (*w*_AA_ − *fw*_BB_ − (1 − *f*)*w*_AB_) (equilibrium 1)	very low	low	high	11
*w*_AA_ > *w*_Aa_, *w*_AB_ > *w*_BB_ > *w*_aa_, *w*_Ba_, *u*′ << *w*_BB_ − *w*_Ba_ − *f*(*w*_aa_ − *w*_Ba_), *t*_2_ << *w*_BB_ − *f* − (1 − *f*)(1 − *m*_1_) *w*_AB_, *m*_1_(1 − *f*) > (*f* + (1 − *f*) *w*_AB_ − *w*_BB_)/*w*_AB_ (equilibrium 2)	very low–low	high	low	Identical to case D, eq. 9
*w*_AA_ > *w*_Aa_, *w*_AB_ > *w*_aa_ > *w*_BB,_ *w*_Ba_ *z* << *w*_aa_ − *f* − (1 − *f*)(1 − *m*_2_)*w*_Aa_, *z* << *w*_aa_ − (1 − *f*)*w*_Ba_ − *fw*_BB_, *m*_2_ (1 − *f*) > (*f* + (1 − *f*) *w*_Aa_ − *w*_aa_)/*w*_Aa_ (equilibrium 3)	very high	very low	very low	12

AN, analyzed numerically, approximation assumptions: *u*, *u*′, *z*, *t*_1_, *t*_2_ ∼ O[*ζ*].

The deleterious allele equilibrium frequency decreases relative to when *m_2_* = 0. This is caused by a reduction in the marginal fitness of the deleterious allele due to the additional paramutation rate converting (epi)genotype A/a to B/a (since by assumption *w*_Aa_ > *w*_Ba_). Next, focusing on the epiallele equilibrium frequency, the first term is the same result as the equilibrium when *m_2_* = 0 (case D, eq. 8) and therefore has the same interpretation. The second term is new and expected to be positive when the equilibrium is biologically valid and therefore the epiallele equilibrium increases with the additional paramutation rate (*m_2_*). The increase is expected to be very small, due to being dependent on the mutation rate (*u*) in the numerator. However, this term does imply that the epiallele can be maintained at a nonzero frequency even if forward spontaneous is completely absent *t*_1_ = 0. This is in contrast to cases A–D for the equilibria where the wild-type is at a high frequency; the epiallele would not be present at equilibrium if *t*_1_ = 0.

A second equilibrium occurs where the epiallele is at high frequency (not shown) and is identical to that of case D (eq. 9). The additional source of paramutation does not impact the epiallele (or the alleles) at this equilibrium. This is likely because both the wild-type and the deleterious allele are at a low equilibrium frequency and the A/a genotype (where m_2_ is relevant) has a negligible impact to the degree of approximation.

Interestingly, a third analytically tractable equilibrium occurs where the deleterious allele is at a high frequency (eq. 12):


$$\begin{array}{r} {\hat{\;p}}_{a}\;\approx \;1-\frac{zw_{a,a}(w_{a,a}-(1-f)(1-m_{2})w_{B,a}-fw_{B,B})}{\left(w_{a,a}-f-(1-f)(1-m_{2})w_{A,a})(w_{a,a}-(1-f)w_{B,a}-fw_{B,B}\right)}+O[\zeta^{2}] \end{array}$$

$$\;\begin{array}{r} {\hat{\;p}}_{B}\;\approx \;\frac{(1-f)zm_{2}w_{a,a}w_{B,a}}{\left(w_{a,a}-f-(1-f)(1-m_{2})w_{A,a})(w_{a,a}-(1-f)w_{B,a}-fw_{B,B}\right)}+O[\zeta^{2}] \end{array}$$

(12) $${\hat{\;p}}_{A}\approx \frac{zw_{a,a}}{\left(w_{a,a}-f-(1-f)(1-m_{2})w_{A,a}\right)}\;+O[\zeta^{2}]$$

It is clear that *m*_2_ allows for this equilibrium to occur in a biologically valid form by reducing the fitness contribution of the A/a heterozygote, which is observed through the term (1 − *m*_2_)*w*_Aa_. Without *m*_2_, this equilibrium is never biologically valid, since by assumption of the model, *w*_Aa_ > *w*_aa_ always holds. In addition, there are constraints on the fitness of the deleterious allele homozygote. One minimum constraint that must hold is *w*_aa_ > *f*. Also, the equilibrium is never biologically valid if *w*_aa_ < *w*_Ba_, *w*_BB_, which is intuitive for an equilibrium where the deleterious allele is at a very high frequency.

Interestingly, inbreeding will always *increase* the epiallele at this equilibrium when *w*_BB_ > *w*_Ba_ as the marginal fitness of the epiallele increases from less pairings with the deleterious allele and more B/B homozygotes. In contrast, the deleterious allele will always decrease due to inbreeding under the same parameter constraints, whereas inbreeding always increases the wild-type allele at this equilibrium, consistent with classic expectations.

In contrast to previous cases, the constraints on this equilibrium are not mutually exclusive with that of eq. 11 where the wild type is at a high frequency. Therefore, when there is an overlap in parameter space where they are both valid, the equilibrium that arises will depend on the initial frequencies.

As a reminder, case D had three equilibria which were assessed numerically, with interesting properties such as inbreeding causing an increase in the deleterious allele (Fig. 5) and cycling of the (epi)allele frequencies (Fig. 6 and see Table 7 for a summary). As assessed numerically, three equilibria with very similar properties arise for case E (data not shown). Also, when the assumption of a small epiallele reversion rate is relaxed for this case (i.e. *t_2_ >> ζ*), we note similar properties at equilibria to previous cases such that the impacts of the other parameters that affect the epiallele are reduced. The net effect is generally a reduction in the epiallele frequency at equilibrium with a corresponding increase in the wild-type allele (data not shown).

## Discussion

### Overview of results

This paper has presented fundamental theory on deleterious epimutation–selection balance. The equilibrium properties with epimutation can be similar to a three-allele genetic model (discussed below), but unique aspects of epigenetic processes can give rise to conditions when it does not. In particular, when paramutation is occurring and when it is near or stronger than natural selection, unexpected equilibria arise and there are nonintuitive effects of certain parameters. Specifically, we find the deleterious allele or the epiallele can reach high frequencies under the condition of a high paramutation rate and inbreeding can increase deleterious genetic or epigenetic variation. Furthermore, the work gives rise to straightforward directions for empirical research, be it characterizing or accounting for deleterious epimutation or examining uncertainties that limit theoretical and therefore conceptual/general understanding of epigenetics.

### Comparison of results to previous population genetics models

A previous paper considered models of deleterious mutation–selection balance with multiple deleterious genetic alleles and incomplete dominance (Clark 1998). Cases A and B (which lack paramutation) could be viewed as a three-allele model with two deleterious alleles, as opposed to one deleterious allele and one deleterious epiallele. However, the distinct biology of epialleles does not allow for some of the assumptions used to obtain analytical results of Clark (1998). Specifically, the assumptions of a single mutation rate of the wild-type allele to distinct deleterious alleles and that the reversion rate is negligible are not generally valid with epialleles. These assumptions were relaxed in Stenøien and Pedersen (2005) which account for the distinct biology of epialleles compared to genetic alleles (see next section for details).

There are similar population genetics contexts to cases C, D, and E (which include paramutation). From a theoretical standpoint, paramutation is analogous to gene conversion (Geoghegan and Spencer 2013c). A previous study looked at the effects of mating systems on gene conversion (Glémin 2010). Inbreeding decreases heterozygous pairings of alleles and epialleles, respectively, and therefore is expected to be of significant importance for both gene conversion and paramutation. These conceptual expectations were confirmed analytically for gene conversion in Glémin (2010) and for paramutation in the current paper. Glémin (2010) highlights how what was previously assumed to be genomic signatures of selection could in fact be gene conversion, such that it represents an additional evolutionary force with its importance underestimated. Paramutation could also be an unaccounted force in shaping epigenetic and genetic variation (Geoghegan and Spencer 2013a).

Although gene conversion and paramutation have similar properties and can have similar outcomes, there are important differences. One finding by Glémin (2010) was an increase in deleterious allele frequencies at equilibrium as a result of gene conversion. We find a similar possibility with the current model. However, in Glémin (2010), gene conversion can cause a direct increase in the deleterious allele, whereas in the case of paramutation the cause is indirect and depends on specific fitness relationships, as a point of distinction. In addition, with paramutation, only one of the alleles may be paramutable and the other is not (Springer and McGinnis 2015). This can put limitations on the ability for paramutation to increase the amount of a deleterious epialleles in comparison with gene conversion increasing the amount of a deleterious allele. Furthermore and interestingly, gene conversion has particularly significant consequences in recombination hotspots, indicating genomic context can be a significant factor (Glémin 2010). In a similar framework, paramutation can depend on the genomic sequence context to occur, but this pattern does not always hold (Springer and McGinnis 2015).

As a final point, our understanding is the deleterious allele with gene conversion is more stable than a deleterious epiallele maintained by paramutation. This distinction arises because of a high epiallele reversion rate. A high epiallele reversion rate can partially or completely mitigate the effects of paramutation (for example see eqs. 7 and 10), something that is unlikely to occur with gene conversion. This suggests the evolution of epigenetic resetting mechanisms can protect against the deleterious potential of paramutation, whereas there may not be a parallel possibility to protecting against the potential accumulation of deleterious alleles due to gene conversion.

### Comparison of results to previous theoretical epigenetic models

A previous study derived analytical equilibrium results for deleterious epimutation–selection balance with incomplete dominance in haploids and diploids (Stenøien and Pedersen 2005). Their results for diploids were similar to our case A results in the context where the epiallele reversion rate was not small in magnitude (i.e. *t_2_ >> ζ*, see eq. 2). The frequency of the deleterious allele was identical to ours, which was the same in form as classic results of two-allele deleterious mutation–selection balance with incomplete dominance. Also, very similar to our case A (eq. 2) results, the epiallele equilibrium frequency was determined by a balance of selection and the epiallele reversion rate acting against it and the forward epimutation rate maintaining it at a nonzero frequency. In a similar modeling framework, Webster and Phillips (2024) developed a model of mutation/spontaneous epimutation–selection balance, but allowing epialleles to also be adaptive. Interestingly, they found that when an epiallele is adaptive it can compensate for a deleterious allele, which causes an increase in the frequency of the deleterious allele from classic mutation–selection balance. They also highlight that the deleterious allele can reach a high equilibrium frequency with the addition of epigenetic variation, but this occurs in a limited/rare area of a parameter space and requires that the epiallele is adaptive, as well as a very low reverse epimutation rate. In our study, the epiallele is always comparatively deleterious to the wild-type allele, yet the deleterious genetic allele can also reach moderate to very high frequencies through a different mechanism, namely as a result of paramutation. We also find the opposite can occur whereby the deleterious allele is below classic mutation–selection balance (e.g. case E, eq. 11). Generally, we have broadened these modeling frameworks by incorporating inbreeding (cases B, D, E) and paramutation (cases C–E).

There are also theoretical studies that directly relate to cases C–E (with paramutation). Spencer and Geoghegan have made seminal contributions to the field of population epigenetics including a series of papers analyzing environmentally influenced epiallele stability and epigenetic contributions to phenotypic variation alongside genetic variation (Geoghegan and Spencer 2012, 2013a, 2013b). The study most similar to the current paper was Geoghegan and Spencer (2013c). This was the first thorough theoretical treatment of paramutation. Similar to our work, their model has two alleles and an epiallele; however, Geoghegan and Spencer (2013c) do not restrict the fitness assumptions, such that one allele and the epiallele are not always assumed deleterious. They identified parameter conditions of equilibria, including fixation of either allele, a polymorphic equilibrium with a high frequency of the epiallele, as well as a polymorphic equilibrium with appreciable genetic and epigenetic variation. Due to the incorporation of mutation, fixation of an allele was not possible for our model; nevertheless, we observe similar results for the equilibria where one of the variants is at a very high frequency. With regard to polymorphic equilibria, as assessed numerically we also identify parameter conditions where all three variants are at appreciable frequencies. Another finding from Geoghegan and Spencer (2013c) was that increases in the epiallele reversion rate increase the possibility of a unique equilibrium arising. Similarly, we find as the epiallele reversion rate increases to a sufficiently high rate we find only one equilibrium is possible because paramutation becomes negligible.

In contrast to the previous work, our current study does not allow for the possibility that the epiallele is adaptive in addition to being deleterious, and so, we have restricted our analysis to deleterious epimutation–selection balance. However, in our model genetic variation is regeneratable by mutation, as well as epigenetic variation by spontaneous forward epimutation. Furthermore, we studied inbreeding to allow for basic insights into the impacts of breeding systems on deleterious epimutation–selection balance and its effects on the phenomenon of paramutation.

Geoghegan and Spencer (2013c) found the epiallele reversion rate can reduce the number of locally stable equilibria by mitigating the effects of paramutation, which was required for some equilibria to arise. We find inbreeding acts as another factor that can restrict the number of locally stable equilibria, by reducing heterozygosity (which is a prerequisite for paramutation), such that equilibria which depend on a high paramutation rate are unlikely to arise. Our analysis allows for both genetic variation and epigenetic variation to be present at all equilibria due to the addition of mutation and forward spontaneous epimutation rates. Specifically, forward spontaneous epimutation can be an important consideration if epialleles are able to form independent of the (epi)genotype (i.e. regardless of paramutation). However, if spontaneous forward spontaneous epimutation is not occurring (i.e. t_1_ = 0), we show (in case E, eqs. 11 and 12) the epiallele can be maintained at equilibrium independent of the forward spontaneous epimutation rate. It is maintained by paramutation caused by the deleterious allele and mutation, which highlights an important contribution of incorporating mutation into the model. Also, of note, we allowed for the epiallele to have a distinct mutation rate from the wild-type allele and this can have nontrivial effects on the equilibrium frequency of the deleterious allele, as epialleles can differ by orders of magnitude in their mutation rates (Yi and Goodisman 2021). Following this, the epiallele could have important effects on the amount of segregating deleterious genetic variation even if the epiallele has nearly neutral deleterious effects.

### The fitness interaction of the epiallele and deleterious allele is a key biological factor for equilibrium outcomes

Epimutations can be deleterious through changes to gene expression that are random with respect to adaptation. In contrast, mutations can be deleterious either through changes to gene expression or protein sequence that are random with respect to adaptation. Therefore, one possibility is that when both an epimutation and a mutation result in a change in gene expression, there could be a compensatory fitness interaction of the deleterious variants based on how each has modified gene expression. In contrast, this could be less likely when there is a deleterious mutational change in protein sequence combined with a deleterious epimutational change in gene expression. Therefore, the type of mutation (i.e. regulatory vs protein) that occurs could be a relevant factor in nature when epimutation is also occurring.

In line with this discussion, in the parameter scenario where paramutation is occurring and is of sufficient magnitude, we find that the relative fitnesses of the a/a, B/B, and B/a (epi)genotypes are a major determining factor in which equilibrium arises. Three distinct and mutually exclusive (i.e. not simultaneously locally stable) equilibria can occur based on the relative magnitudes of *w*_aa_, *w*_Ba,_ and *w*_BB_. If there is a compensatory fitness interaction of the deleterious allele and the epiallele, this allows for the equilibrium where either variant can be at high frequency or both can be at intermediate equilibrium frequencies based on the relative magnitudes of *w*_aa_ and *w*_BB_. Alternatively, if when paired together they are more deleterious (*w*_aa_*, w*_BB_  *> w*_Ba_), then the deleterious allele can reach high frequencies when *w*_aa_ > *w*_BB_ and this is also when cycling occurs as the equilibrium is approached. Alternatively, if *w*_aa_ < *w*_BB_, this allows for the equilibrium where the epiallele is at a high frequency, essentially taking the place of the wild-type allele in the population (see eqs. 6 and 9). Overall, whether a given mutation or epimutation at a genetic locus is more deleterious and depending on their fitness interaction, this can result in quite different equilibrium outcomes and distinct paths to reaching equilibria.

### Epimutation and mating systems

Based on the demonstrated results on the importance of inbreeding effects on deleterious epigenetic variation, there may be patterns in nature reflecting the coevolution of epialleles and mating systems. Comparisons could be performed on sister species (such as *Arabidopsis* species), for epigenetic modifications such as methylation, and molecular signatures of paramutation. One would expect when comparing highly inbreeding vs randomly mating sister species there would be significantly different epiallele frequencies. Populations with high epimutation rates such as through the process of paramutation may result in near fixation of epialleles in a random mating species, whereas highly inbred sister species would be much more protected from high epimutation rates, reducing the frequency of deleterious epialleles at equilibria. However, we note parameter scenarios where there is a more unexpected effect of the degree of inbreeding. For example, when the epiallele is very deleterious (*w*_BB_ < 0.5*w*_AB_, eg. see eq. 5), paramutation reduces its frequency at equilibrium and this purging effect of paramutation is stronger with random mating. Also, unexpectedly, we find inbreeding can increase the equilibrium frequencies of the deleterious allele or the epiallele under some conditions. This is in direct contrast to the expectations of the increased efficiency of selection against deleterious variation as inbreeding increases within the classic modeling framework. The more unexpected impacts of inbreeding occur when it is at low to moderate levels and when paramutation is stronger than selection. When inbreeding reaches high levels (such that paramutation has a relatively minimal effect), both deleterious genetic variation and epigenetic variation are simultaneously decreased by inbreeding, in line with classic expectations. Overall, these results suggest more nuanced relationships of breeding systems and segregating deleterious variation than anticipated.

### General implications of deleterious epimutation–selection balance

Epimutation is expected to have neutral or deleterious effects when impacting phenotypically relevant genetic loci, analogous to classical understanding of mutation (Bonduriansky and Day 2018). As shown, deleterious epimutations may cause significant additional segregating deleterious variation. However, in order for these effects to impact populations, epimutations and the corresponding epialleles must be heritable, variable among individuals, and have nonnegligible effects on fitness (Bonduriansky and Day 2018).

It is possible that as an overall pattern, when epimutation is viewed as a largely deleterious process (when nonneutral, like mutation), but its causal mechanisms are nonetheless invaluable for certain biological roles in many clades, such as transposon methylation and phenotypic plasticity, natural selection minimizes its deleterious consequences through different paths such as generating resetting mechanisms (Charlesworth *et al*. 2017), increasing reversion rates over forward (van der Graaf *et al*. 2015), and possibly even reorganizing genomes (Choi and Yuh 2020). Alternatively, for vulnerable populations that possess evolutionarily relevant epigenetic variation, there may be a significant additional risk to their survival, and the co-evolution of epimutation with mating systems will be of prime importance.

## Supplementary Material

iyae080_Supplementary_Data

## Data Availability

The authors affirm that all data necessary for confirming the conclusions of the article are present within the article, figures, tables, and Supplementary material. Supplementary File S1 goes through the detailed analytical derivation of the recursion equations used for the model. Supplementary File S2 contains a detailed calculations and analysis corresponding to the main text. Supplementary File S3 demonstrates the results of the complete dominance context with detailed calculations and analysis with supporting figures.

## Supplemental Material

Supplemental material available at GENETICS online.

## Appendix

### A1: determining equilibria

Represented as general functions, the systems of recursion equations for the deleterious allele and epiallele are $p_{a}^{\prime }=f_{1}(\;p_{a},\;p_{B};\;n_{1,1},\;n_{1,2},\;\ldots ,\;n_{1,x})$ and $p_{B}^{\prime }=f_{2}(\;p_{a},\;p_{B};\;n_{2,1},n_{2,2},\;\ldots ,n_{2,y})$, such that $n_{i}$ is a general symbol for a parameter. “*x*” and “*y*” represent the number of parameters present in the recursion equations for the deleterious allele and epiallele, respectively. The multivariate equilibrium condition is

$${\hat{\;p}}_{a}=f_{1}({\hat{\;p}}_{a},{\hat{\;p}}_{B};n_{1,1},n_{1,2},\ldots ,n_{1,x})$$

$${\hat{\;p}}_{B}=f_{2}({\hat{\;p}}_{a},{\hat{\;p}}_{B};n_{2,1},n_{2,2},\ldots ,n_{2,y})$$

For each fitness and inbreeding case, a particular set of parameters were assumed to be of order (ζ), such that any parameter assumed to be of order (ζ) for a given case is transformed: $n_{i}$ −> $\tilde{n_{i}}\;\zeta \;$ (Otto and Day 2007). Furthermore, the equilibria (${\hat{\;p}}_{a}$, ${\hat{\;p}}_{B}$), are assumed to be perturbations from equilibria. Define ($p_{a,0}$, $\;p_{B,0}$) to be zeroth-order equilibria and ($p_{a,1}\;$, $p_{B,1}$) to be first-order perturbations. Accordingly, the following substitutions are made into equations for the equilibrium frequencies: ${\hat{\;p}}_{a}\;$ = $p_{a,0}$ + $p_{a,1}\zeta \;$ +… and $\hat{\;p_{B}}\;$ = $p_{B,0}$ + $p_{B,1}\zeta \;$ +…resulting in $f_{1}(\zeta )=p_{a,0}$ + $p_{a,1}\zeta \;$ + …, $f_{2}(\zeta )=p_{B,0}$ + $p_{B,1}\zeta$ +…. $f_{1}(\zeta )$ and $f_{2}(\zeta )$ are expressions for the recursion equations after the above substitutions for the variables and parameters of order (ζ) have been made. The first-order Taylor's approximations of $f_{1}(\zeta )$ and $f_{2}(\zeta )$ about ζ and evaluated at ζ = 0 result in the following system of equations:

$$(\;f_{1}(\zeta )-p_{a,0}){|}_{\zeta =0}+\left(\frac{df_{1}(\zeta )}{d\zeta }-p_{a,1}\right){|}_{\zeta =0}(\zeta )+O[\zeta^{2}]=0$$

$$(\;f_{2}(\zeta )-p_{B,0}){|}_{\zeta =0}+\left(\frac{df_{2}(\zeta )}{d\zeta }-p_{B,1}\right){|}_{\zeta =0}(\zeta )+O[\zeta^{2}]=0$$

The above system is satisfied if the terms $(\;f_{i}(\zeta )-p_{\;j,k}){|}_{\zeta =0}$ and $\left(\frac{df_{i}(\zeta )}{d\zeta }-p_{\;j,l}\right){|}_{\zeta =0}$ equal zero such that the zeroth-order terms satisfy:

$$0=(\;f_{1}(\zeta )-p_{a,0}){|}_{\zeta =0}$$

$$0=(\;f_{2}(\zeta )-p_{B,0}){|}_{\zeta =0}.$$

Solving for $p_{a,0}$ and $p_{B,0}$ simultaneously gives all zeroth-order equilibria for the deleterious allele and epiallele frequencies. The system for the first-order terms is

$$0=\left(\frac{df_{1}(\zeta )}{d\zeta }-p_{a,1}\right){|}_{\zeta =0}$$

$$0=\left(\frac{df_{2}(\zeta )}{d\zeta }-p_{B,1}\right){|}_{\zeta =0}$$

Letting $\left(\;p_{a,0,i}\;,\;p_{B,0,i}\right)$ represent an arbitrary equilibrium for the zeroth-order terms, substituting this into the above equations, and solving for $p_{a,1}\;\mathrm{and}\;p_{B,1}$ simultaneously gives the first-order terms that satisfy the multivariate equilibrium condition. Therefore, a first-order approximation is

$${\hat{\;p}}_{a,i\;}\approx \;p_{a,0,i}+p_{a,1,i}\zeta +O[\zeta^{2}],$$

$${\hat{\;p}}_{B,i}\;\approx \;p_{B,0,i}+p_{B,1,i}\zeta +O[\zeta^{2}],$$

$${\hat{\;p}}_{A,i}\;\approx \;1-{\hat{\;p}}_{a,i}-{\hat{\;p}}_{B,i}+O[\zeta^{2}].$$

Original parameters are then substituted back that were assumed to be of order *ζ*, $\tilde{n_{i}}\zeta$ = $n_{i}$, to obtain the first-order equilibrium approximation.

### A2: local stability analysis

Recursion equations for the deleterious allele and epiallele frequencies are

$$p_{a}^{\prime }=f_{1}(\;p_{a},p_{B})$$

$$p_{B}^{\prime }=f_{2}(\;p_{a},p_{B}).$$

The Jacobian matrix for the recursion equations is

$$\left(\begin{array}{cc} \frac{\partial (\;f_{1}(\;p_{a},p_{B}))}{\partial p_{a}} & \frac{\partial (\;f_{1}(\;p_{a},p_{B}))}{\partial p_{B}}\\ \frac{\partial (\;f_{2}(\;p_{a},p_{B}))}{\partial p_{a}} & \frac{\partial (\;f_{2}(\;p_{a},p_{B}))}{\partial p_{B}} \end{array}\right)$$

The eigenvalues of this Jacobian matrix evaluated at an equilibrium indicate its local stability (Otto and Day 2007). Let an eigenvalue be approximated as $\lambda \;\approx \;\lambda_{0}\;$+$\;\lambda_{1}\zeta$ + $\;\lambda_{2}\zeta^{2}$ …Then, the characteristic equation g(ζ) is

$$g(\zeta )=\det\left({\left(\begin{array}{cc} \frac{\partial (\;f_{1}(\;p_{a},p_{B}))}{\partial p_{a}} & \frac{\partial (\;f_{1}(\;p_{a},p_{B}))}{\partial p_{B}}\\ \frac{\partial (\;f_{2}(\;p_{a},p_{B}))}{\partial p_{a}} & \frac{\partial (\;f_{2}(\;p_{a},p_{B}))}{\partial p_{B}} \end{array}\right)}_{\left|(\;p_{a}\;=\;p_{a,0}+p_{a,1}\zeta ,p_{B\;}=\;p_{B,0}+p_{B,1}\zeta ,n_{i}\;=\;\;\tilde{n_{i}}\;\zeta \;,\ldots \right)}-\left(\begin{array}{cc} \;\lambda_{0}\;+\;\lambda_{1}\zeta \;+\;\lambda_{2}\zeta^{2}\ldots & 0\\ 0 & \;\lambda_{0}\;+\;\lambda_{1}\zeta \;+\;\lambda_{2}\zeta^{2}\ldots \end{array}\right)\right)$$

Performing a Taylor series with respect to *ζ* around the point (ζ=0) on the characteristic equation approximated to the first order is

$$g(\zeta ){|}_{\zeta =0}+\left(\frac{dg(\zeta )}{d\zeta }\right){|}_{\zeta =0}(\zeta )+O[\zeta^{2}]=0$$

The characteristic equation has the form a($\lambda_{0}$) + b($\lambda_{0}$, $\;\lambda_{1}$) ζ = 0. Solving the zeroth-order term [a($\lambda_{0}$)] for eigenvalues gives both zeroth-order eigenvalues $(\lambda_{0,1}$, $\lambda_{0,2})$. Separately substituting both these solutions into the first-order term and then solving for the first-order term give both first-order terms $(\lambda_{1,1}$, $\lambda_{1,2})$. This is followed by substituting in the original parameters that were assumed to be of order (*ζ*), $\;\tilde{n_{i}}\;\zeta$ = $n_{i}$, giving the approximated eigenvalues

$$\lambda_{\left(1\right)}\;\approx \;\lambda_{0,1}+\lambda_{1,1}+O[\zeta^{2}]$$

$$\lambda_{\left(2\right)}\;\approx \;\lambda_{0,2}+\lambda_{1,2}+O[\zeta^{2}]$$

The approximated eigenvalues can then be used to determine local stability such that $\left|\lambda_{\left(1\right)}\right|$ < 1 and $|\lambda_{\left(2\right)}|\;$ < 1 (Otto and Day 2007).

### A3: example of an equilibrium calculation and local stability analysis:

To demonstrate the analysis procedure, go through the above methods to determine the equilibria and perform a local stability analysis on case A as an example. First, following assumptions of this case, letting the paramutation rate equal zero (m = 0), and inputting the fitness assumptions of Table 1 into the original recursion equations give the recursion equations for the deleterious allele and epiallele, respectively (see Supplementary File S2, case A for full recursion equations):

$$p_{a}^{\prime }=f_{1}(\;p_{a},p_{B})$$

$$p_{B}^{\prime }=f_{2}(\;p_{a},p_{B})$$

Then, setting the equilibrium condition (see Supplementary File S2, case A for full equations),

$$f_{1}({\hat{\;p}}_{a},{\hat{\;p}}_{B})-{\hat{\;p}}_{a}\;=0$$

$$f_{1}({\hat{\;p}}_{a},{\hat{\;p}}_{B})-{\hat{\;p}}_{B}\;=0$$

It is assumed that spontaneous epimutation and mutation are of order zeta, such that $t_{1}=\tilde{t_{1}}\zeta ,\;t_{2}=\tilde{t_{2}}\zeta ,\;u=\tilde{u}\zeta$, $=\tilde{z}\zeta$, $\;c=\tilde{c}\zeta$ and equilibria are

$${\hat{\;p}}_{a}=p_{a,0}+p_{a,1}\zeta +\ldots$$

$${\hat{\;p}}_{B}=p_{B,0}+p_{B,1}\zeta +\ldots$$

Next, a Taylor series is performed with respect to *ζ* around the point (ζ=0) on both equations separately, and including up to the first-order terms. The equilibrium condition for the zeroth-order terms is

$$\left(-p_{a,0}+\frac{-((-1+s)p_{a,0}^{2})+(1-hs-h_{2}s_{2}+s_{3})p_{a,0}p_{B,0}+(-1+hs)p_{a,0}(-1+p_{a,0}+p_{B,0})}{1-2hsp_{a,0}-sp_{a,0}^{2}+2hsp_{a,0}^{2}-2h_{2}s_{2}p_{B,0}+2s_{3}p_{a,0}p_{B,0}-s_{2}p_{B,0}^{2}+2h_{2}s_{2}p_{B,0}^{2}}\right)=0$$

$$\left(-p_{B,0}\frac{-(\left(-1+h_{2}s_{2})p_{B,0}(-1+p_{a,0}+p_{B,0}\right))+p_{B,0}((-1+hs+h_{2}s_{2}-s_{3})p_{a,0}+(-1+s_{2})p_{B,0})}{-1+2hsp_{a,0}+sp_{a,0}^{2}-2hsp_{a,0}^{2}+2h_{2}s_{2}p_{B,0}-2s_{3}p_{a,0}p_{B,0}+s_{2}p_{B,0}^{2}-2h_{2}s_{2}p_{B,0}^{2}}\right)=0$$

Solving for $p_{a,0}$, and $p_{B,0}$ gives three sets of zeroth-order terms. However, after further analysis, only one leads to a biologically valid equilibrium, $\left(\;p_{a,0}=0,p_{B,0}=0\right)$. Substituting these values for the zeroth-order frequencies into the first-order terms (see Supplementary File S2, case A) for both equations gives the equilibrium condition for the first-order terms,

$$u-p_{a,1}+(1-hs)p_{a,1}=0$$

$$t_{1}-p_{B,1}-(-1+h_{2}s_{2})p_{B,1}=0$$

Solving for $p_{a,1}$ and $p_{B,1}$ and incorporating with the zeroth-order terms give the first-order approximation for the allele and epiallele frequencies:

$${\hat{\;p}}_{a}\;\approx \;\frac{\tilde{u}}{hs}\zeta +O[\zeta^{2}]$$

$${\hat{\;p}}_{B}\;\approx \;\frac{\tilde{t_{1}}}{h_{2}s_{2}}\zeta +O[\zeta^{2}]$$

$${\hat{\;p}}_{A}\;\approx \;1-\frac{\tilde{u}}{hs}\zeta -\frac{\tilde{t_{1}}}{h_{2}s_{2}}\zeta +O[\zeta^{2}]$$

Then, back substituting the original parameters of order (ζ), $\;(\;\tilde{t_{1}}\zeta =t_{1}\;,\;\tilde{u}\zeta =\;u)$, gives the first-order approximation.

Next, a local stability analysis is performed for this equilibrium. The Jacobian matrix is calculated and expressions for the approximated equilibrium terms and the mutation and spontaneous epimutation rates are substituted and assumed to be of order (ζ), such that $p_{B}=p_{B,0}+p_{B,1}\zeta \;,p_{a}=p_{a,0}+p_{a,1}\zeta ,t_{1}=\tilde{t_{1}}\zeta ,\;t_{2}=\tilde{t_{2}}\zeta ,\;u=\tilde{u}\zeta$, $z=\tilde{z}\zeta$, $\;c=\tilde{c}\zeta$. After the substitutions, the characteristic equation was calculated, and then, its first-order Taylor's approximation with respect to *ζ* around the point (ζ = 0) was evaluated. Next, substituting in explicit equilibrium terms for this equilibrium $\left(\;p_{a,0}=0,\;p_{B,0}=0,\;p_{a,1}=\frac{u}{hs},\;p_{B,1}=\frac{t_{1}}{h_{2}s_{2}}\right)$ results in the first-order approximation for the characteristic equation. Solving

$$(1-hs-\lambda_{0})(1-h_{2}s_{2}-\lambda_{0})=0$$

for the zeroth-order terms gives

$$\lambda_{0,1}=1-hs,\;\;\lambda_{0,2}=1-h_{2}s_{2}.$$

Substituting $\lambda_{0,1}=1-hs$ into the first-order term above and solving for $\lambda_{1,1}$ gives

$$\lambda_{1,1}=\left(5-\frac{2}{h}-3hs\right)u+(-1+hs)z+\left(1-2hs+\frac{s_{3}}{h_{2}s_{2}}\right)t_{1}$$

Then, making the substitution $\lambda_{0,2}=1-h_{2}s_{2}$ into the first-order term and solving for $\lambda_{1,2}$, results in

$$\lambda_{1,2}\;=\;-c+u+\frac{us_{3}}{hs}+5t_{1}-\frac{2t_{1}}{h_{2}}-t_{2}+h_{2}s_{2}(c-2u-3t_{1}+t_{2})+O[\zeta^{2}]$$

Bringing the zero- and first-order terms together and simplifying give the first-order approximation for the eigenvalues

$$\lambda_{\left(1\right)}\;\approx \;1-hs+\left(5-\frac{2}{h}-3hs\right)u+(-1+hs)z+\left(1-2hs+\frac{s_{3}}{h_{2}s_{2}}\right)t_{1}+O[\zeta^{2}]$$

$$\lambda_{\left(2\right)}\;\approx 1-h_{2}s_{2}-c+u+\frac{us_{3}}{hs}+5t_{1}-\frac{2t_{1}}{h_{2}}-t_{2}+h_{2}s_{2}(c-2u-3t_{1}+t_{2})+O[\zeta^{2}]$$

Note: All analysis was performed using Wolfram Mathematica, version 12.3.1.0.
