# Supplementary material for: Deleterious mutation/epimutation–selection balance with and without inbreeding: a population (epi)genetics model
Source: Genetics. 2024 May 11;227(3):iyae080. doi: 10.1093/genetics/iyae080 (PMC11228854; doi:10.1093/genetics/iyae080)
Supplement: iyae080_Supplementary_Data [file iyae080_supplementary_data.zip › File_S1_GENETICS-2024-306923.pdf]

# Supplement Overview

## Table of Contents


### File S1 - Derivation of Recursion Equations for Model

### File S2 - Incomplete Dominance: Analysis of equilibria and local stability
Introduction
Case A: Random Mating and without Paramutation
Case B: With Inbreeding and without Paramutation
Case C: Random Mating and with Paramutation
Case D: With Inbreeding and with Paramutation
Case E: With Inbreeding and the Deleterious Allele Causes Paramutation

### File S3 - Complete Dominance: Analysis of equilibria and local stability
Introduction
Case A: Random Mating and without Paramutation
Case B: With Inbreeding and without Paramutation
Case C: Random Mating and with Paramutation
Case D: With Inbreeding and with Paramutation


## Derivation of the recursion equations for the allele/epiallele frequencies

The assumed sequence of events, is the pairing of gametes with inbreeding, followed by paramutation occurring in zygotes. Natural selection then occurs based on the (epi)genotypes, followed by spontaneous epimutation of the next generation of gametes. Then mutation of the gametes occurs as the final event in the life cycle. $p_A$, $p_B$, and $p_a$ are frequencies of the wild-type allele (A), epiallele (B), and deleterious allele (a) respectively. (Epi)genotype frequencies are A/A → $p_{AA}$, A/*B* → $p_{AB}$, A/a → $p_{Aa}$, *B*/*B* → $p_{BB}$, *B*/a → $p_{Ba}$, a/a → $p_{aa}$.

Genotype frequencies after gametes pair with inbreeding$^{(1)}$:

$p_{AA}^{(1)} = p_A\, p_A\, (1 - f) + p_A\, f$

$p_{AB}^{(1)} = 2\, p_A\, p_B\, (1 - f)$

$p_{Aa}^{(1)} = 2\, p_A\, p_a\, (1 - f)$

$p_{BB}^{(1)} = p_B\, p_B\, (1 - f) + p_B\, f$

$p_{Ba}{}^{(1)} = 2\ p_B\, p_a\, (1 - f)$

$p_{aa}{}^{(1)} = p_a\, p_a\, (1 - f) + p_a\, f$

After paramutation in zygotes$^{(2)}$:

$p_{AA}{}^{(2)} = p_{AA}{}^{(1)}$

$p_{AB}{}^{(2)} = p_{AB}{}^{(1)}\, (1 - m)$

$p_{Aa}{}^{(2)} = p_{Aa}{}^{(1)}$

$p_{BB}{}^{(2)} = p_{BB}{}^{(1)} + \left(p_{AB}{}^{(1)}\right) m$

$p_{Ba}{}^{(2)} = p_{Ba}{}^{(1)}$

$p_{aa}{}^{(2)} = p_{aa}{}^{(1)}$

Then natural selection on (epi) genotypes occurs, normalized by mean fitness$^{(3)}$:

$$p_{AA}{}^{(3)} = \frac{w_1 \left(p_{AA}{}^{(2)}\right)}{\overline{w}}$$

$$p_{AB}{}^{(3)} = \frac{w_2 \left(p_{AB}{}^{(2)}\right)}{\overline{w}}$$

$$p_{Aa}{}^{(3)} = \frac{w_3 \left(p_{Aa}{}^{(2)}\right)}{\overline{w}}$$

$$p_{BB}{}^{(3)} = \frac{w_4 \left(p_{BB}{}^{(2)}\right)}{\overline{w}}$$

$$p_{Ba}{}^{(3)} = \frac{w_5 \left(p_{Ba}{}^{(2)}\right)}{\overline{w}}$$

$$p_{aa}{}^{(3)} = \frac{w_6 \left(p_{aa}{}^{(2)}\right)}{\overline{w}}$$

where,

Mean Fitness $= \overline{w} = w_1 \left(p_{AA}{}^{(2)}\right) + w_2 \left(p_{AB}{}^{(2)}\right) + w_3 \left(p_{Aa}{}^{(2)}\right) + w_4 \left(p_{BB}{}^{(2)}\right) + w_5 \left(p_{Ba}{}^{(2)}\right) + w_6 \left(p_{aa}{}^{(2)}\right)$, and

$$p_A{}^{(3)} = p_{AA}{}^{(3)} + \frac{1}{2} p_{AB}{}^{(3)} + \frac{1}{2} p_{Aa}{}^{(3)}$$

$$p_B{}^{(3)} = p_{BB}{}^{(3)} + \frac{1}{2}\, p_{AB}{}^{(3)} + \frac{1}{2}\, p_{Ba}{}^{(3)}$$

$$p_a{}^{(3)} = p_{aa}{}^{(3)} + \frac{1}{2}\, p_{Aa}{}^{(3)} + \frac{1}{2}\, p_{Ba}{}^{(3)}$$

(Epi)allele frequencies after forward and reverse spontaneous epimutation of gametes$^{(4)}$:

$$p_A{}^{(4)} = p_A{}^{(3)}\,(1 - t_1) + t_2\, p_B{}^{(3)}$$

$$p_B{}^{(4)} = p_B{}^{(3)}\,(1 - t_2) + t_1\, p_A{}^{(3)}$$

$$p_a{}^{(4)} = p_a{}^{(3)}$$

Followed by forward and reverse mutation of gametes$^{(5)}$

$$p_A{}^{(5)} = p_A{}^{(4)}\,(1 - u) + p_a{}^{(4)}\, z$$

$$p_B{}^{(5)} = p_B{}^{(4)}\,(1 - c)$$

$$p_a{}^{(5)} = p_a{}^{(4)}\,(1 - z) + c\, p_B{}^{(4)} + u\, p_A{}^{(4)}$$

Note, in the main text: $c = u'$.

Inputting each event into the consecutive event to derive the recursion equations, and inputting the expression for mean fitness, then after simplification the recursion equations for the wild-type allele, epiallele, and deleterious allele respectively are

$$\begin{aligned} p_A{}' = \big(&-\big((-1+f)\,(-1+u)\,p_A^2\,(-1+t_1)\,w_1\big) + (-1+f)\,(-1+u)\,p_B^2\,t_2\,w_4 + \\ &p_A\,(f\,(-1+u)\,(-1+t_1)\,w_1 + (-1+f)\,(p_a\,(-1+u-z-(-1+u)\,t_1)\,w_3 + (-1+u)\,p_B\,((-1+m)\,(-1+t_1-t_2)\,w_2 + 2\,m\,t_2\,w_4))) + \\ &p_B\,(-((-1+f)\,z\,p_a\,w_5) - (-1+u)\,t_2\,(f\,w_4 - (-1+f)\,p_a\,w_5)) + z\,p_a\,(f-(-1+f)\,p_a)\,w_6\big) \big/ \\ \big(&-\big((-1+f)\,p_A^2\,w_1\big) - (-1+f)\,p_B^2\,w_4 + p_A\,(f\,w_1 + 2\,(-1+f)\,(-p_a\,w_3 + p_B\,((-1+m)\,w_2 - m\,w_4))) + \\ &p_B\,(f\,w_4 - 2\,(-1+f)\,p_a\,w_5) + p_a\,(f-(-1+f)\,p_a)\,w_6\big) \end{aligned}$$

$$\begin{aligned} p_B{}' = -\Big(&\big((-1+c)\,\big((-1+f)\,p_A^2\,t_1\,w_1 - p_A\,(t_1\,(f\,w_1 + (-1+f)\,((-1+m)\,p_B\,w_2 - p_a\,w_3)) - (-1+f)\,p_B\,(-1+t_2)\,((-1+m)\,w_2 - 2\,m\,w_4)) - \\ &p_B\,(-1+t_2)\,((-f+(-1+f)\,p_B)\,w_4 + (-1+f)\,p_a\,w_5)\big)\big) \big/ \big((-1+f)\,p_A^2\,w_1 + (-1+f)\,p_B^2\,w_4 - \\ &p_A\,(f\,w_1 + 2\,(-1+f)\,(-p_a\,w_3 + p_B\,((-1+m)\,w_2 - m\,w_4))) - p_B\,(f\,w_4 - 2\,(-1+f)\,p_a\,w_5) + p_a\,(-f+(-1+f)\,p_a)\,w_6\big)\Big) \end{aligned}$$

$$\begin{aligned} p_a{}' = \big(&(-1+f)\,p_A^2\,(-u+(-c+u)\,t_1)\,w_1 + (-1+f)\,p_B^2\,(-c+(c-u)\,t_2)\,w_4 + p_A\,(f\,(u+(c-u)\,t_1)\,w_1 + \\ &(-1+f)\,(p_a\,(-1-u+z+(-c+u)\,t_1)\,w_3 + p_B\,((-1+m)\,(c+u+(c-u)\,t_1+(-c+u)\,t_2)\,w_2 + 2\,m\,(-c+(c-u)\,t_2)\,w_4))) + \\ &p_B\,(f\,(c+(-c+u)\,t_2)\,w_4 + (-1+f)\,p_a\,(-1-c+z+(c-u)\,t_2)\,w_5) + (-1+z)\,p_a\,(-f+(-1+f)\,p_a)\,w_6\big) \big/ \\ \big(&-\big((-1+f)\,p_A^2\,w_1\big) - (-1+f)\,p_B^2\,w_4 + p_A\,(f\,w_1 + 2\,(-1+f)\,(-p_a\,w_3 + p_B\,((-1+m)\,w_2 - m\,w_4))) + \\ &p_B\,(f\,w_4 - 2\,(-1+f)\,p_a\,w_5) + p_a\,(f-(-1+f)\,p_a)\,w_6\big) \end{aligned}$$

(Eq. 1)

Next, due to the constraint: $p_A + p_B + p_a = 1$, the dimensions can be reduced from three to two. Following classic theory we choose to explicitly track the deleterious allele and epiallele frequencies (where $p_A = 1 - p_a - p_B$ ). After substitution, the recursion equations are

$$p_B' = -\Big(\big((-1+c)\big((-1+f)(-1+p_a+p_B)^2 t_1 w_1 - (1-p_a-p_B)(t_1(f w_1 + (-1+f)((-1+m) p_B w_2 - p_a w_3)) - (-1+f) p_B (-1+t_2)((-1+m) w_2 - 2 m w_4)) - p_B(-1+t_2)((-f+(-1+f)p_B) w_4 + (-1+f) p_a w_5)\big)\big) \Big/ \big((-1+f)(-1+p_a+p_B)^2 w_1 + (-1+f) p_B^2 w_4 - (1-p_a-p_B)(f w_1 + 2(-1+f)(-p_a w_3 + p_B((-1+m) w_2 - m w_4))) - p_B(f w_4 - 2(-1+f) p_a w_5) + p_a(-f+(-1+f)p_a) w_6\big)\Big)$$

$$p_a' = \big((-1+f)(-1+p_a+p_B)^2(-u+(-c+u)t_1) w_1 + (-1+f) p_B^2 (-c+(c-u)t_2) w_4 + (1-p_a-p_B)(f(u+(c-u)t_1) w_1 + (-1+f)(p_a(-1-u+z+(-c+u)t_1) w_3 + p_B((-1+m)(c+u+(c-u)t_1+(-c+u)t_2) w_2 + 2 m(-c+(c-u)t_2) w_4))) + p_B(f(c+(-c+u)t_2) w_4 + (-1+f) p_a(-1-c+z+(c-u)t_2) w_5) + (-1+z) p_a(-f+(-1+f)p_a) w_6\big) \Big/ \big(-\big((-1+f)(-1+p_a+p_B)^2 w_1\big) - (-1+f) p_B^2 w_4 + (1-p_a-p_B)(f w_1 + 2(-1+f)(-p_a w_3 + p_B((-1+m) w_2 - m w_4))) + p_B(f w_4 - 2(-1+f) p_a w_5) + p_a(f-(-1+f)p_a) w_6\big)$$

(Eq. 2)

In order to perform numerical simulations of the above recursion equations, a few substitutions were made: $p_B' = pB[t+1]$, $p_B = pB[t]$, $p_a' = pa[t+1]$, and $p_a = pa[t]$. Additionally, fitness terms $w_i$ were substituted to correspond to the incomplete dominance context of the main text (table 3) and the spontaneous epimutation rate symbols were changed ( $t_1 = r1$ and $t_2 = r2$ ) since ‘t’ now represents time (in generations). Following these changes, the coding below was used to generate figures 2, 3 and 5 of the main text:

```
pa[1] := 0.01;
pB[1] := 0.01;
s =;
s2 =;
s3 =;
r1 =;
r2 =;
u =;
z =;
c =;
f =;
h =;
h2 =;
m =;
```

```
tb1 = {{t, pa[t]}};

tb2 = {{t, pB[t]}};

tb3 = {{pa[t], pB[t]}};

For[t = 1, t ≤ 10000, t++,

 pB[t + 1] =
  -(((-1 + c) ((-1 + f) r1 (-1 + pa[t] + pB[t])^2 - (-1 + r2) pB[t] ((-1 + f) (1 - h s - h2
                s2 + s3) pa[t] + (1 - s2) (-f + (-1 + f) pB[t])) - (1 - pa[t] - pB[t])
          (-((-1 + f) (-1 + r2) (-2 m (1 - s2) + (-1 + m) (1 - h2 s2)) pB[t]) +
            r1 (f + (-1 + f) (-((1 - h s) pa[t]) + (-1 + m) (1 - h2 s2) pB[t]))))) /
     ((1 - s) pa[t] (-f + (-1 + f) pa[t]) - (f (1 - s2) - 2 (-1 + f) (1 - h s - h2 s2 + s3) pa[t])
        pB[t] + (-1 + f) (1 - s2) pB[t]^2 + (-1 + f) (-1 + pa[t] + pB[t])^2 - (1 - pa[t] - pB[t])
        (f + 2 (-1 + f) (-((1 - h s) pa[t]) + (-m (1 - s2) + (-1 + m) (1 - h2 s2)) pB[t]))));

 pa[t + 1] = ((1 - s) (-1 + z) pa[t] (-f + (-1 + f) pa[t]) +
     (f (1 - s2) (c + r2 (-c + u)) + (-1 + f) (1 - h s - h2 s2 + s3) (-1 - c + r2 (c - u) + z) pa[t])
      pB[t] + (-1 + f) (1 - s2) (-c + r2 (c - u)) pB[t]^2 +
     (-1 + f) (-u + r1 (-c + u)) (-1 + pa[t] + pB[t])^2 + (1 - pa[t] - pB[t]) (f (r1 (c - u) + u) +
        (-1 + f) ((1 - h s) (-1 - u + r1 (-c + u) + z) pa[t] + (2 m (1 - s2) (-c + r2 (c - u)) +
              (-1 + m) (1 - h2 s2) (c + r1 (c - u) + u + r2 (-c + u))) pB[t]))) /
    ((1 - s) pa[t] (f - (-1 + f) pa[t]) + (f (1 - s2) - 2 (-1 + f) (1 - h s - h2 s2 + s3) pa[t])
      pB[t] - (-1 + f) (1 - s2) pB[t]^2 - (-1 + f) (-1 + pa[t] + pB[t])^2 + (1 - pa[t] - pB[t])
      (f + 2 (-1 + f) (-((1 - h s) pa[t]) + (-m (1 - s2) + (-1 + m) (1 - h2 s2)) pB[t])));

 tb1 = Append[tb1, {t, pa[t + 1]}];

 tb2 = Append[tb2, {t, pB[t + 1]}];

 tb3 = Append[tb3, {pa[t], pB[t]}];

]
```

A similar approach was used for numerical evaluations in the complete dominance case, except using the corresponding system of recursion equations:

```
pa[1] := 0.01;
pB[1] := 0.01;
s =;
s2 =;
r1 =;
r2 =;
u =;
z =;
c =;

tb300 = {{t, pa[t]}};

tb301 = {{t, pB[t]}};

tb302 = {{pa[t], pB[t]}};

For[t = 1, t ≤ 10000, t++,

 pB[t + 1] =
  - (((-1 + c) (-r1 (-1 + pa[t] + pB[t])^2 - (-1 + r2) pB[t] (- ((1 - s2) pa[t]) - (1 - s2)
          pB[t]) - (1 - pa[t] - pB[t]) (- ((-1 + r2) pB[t]) + r1 (pa[t] + pB[t])))) /
     (- ((1 - s) pa[t]^2) + 2 (-pa[t] - pB[t]) (1 - pa[t] - pB[t]) -
       2 (1 - s2) pa[t] × pB[t] - (1 - s2) pB[t]^2 - (-1 + pa[t] + pB[t])^2));

 pa[t + 1] =
  (- ((1 - s) (-1 + z) pa[t]^2) - (1 - s2) (-1 - c + r2 (c - u) + z) pa[t] × pB[t] - (1 - s2)
      (-c + r2 (c - u)) pB[t]^2 - (-u + r1 (-c + u)) (-1 + pa[t] + pB[t])^2 + (1 - pa[t] - pB[t])
      (- ((-1 - u + r1 (-c + u) + z) pa[t]) - (-c - r1 (c - u) - u - r2 (-c + u)) pB[t])) /
   ((1 - s) pa[t]^2 - 2 (-pa[t] - pB[t]) (1 - pa[t] - pB[t]) + 2 (1 - s2) pa[t] × pB[t] +
     (1 - s2) pB[t]^2 + (-1 + pa[t] + pB[t])^2);

 tb300 = Append[tb300, {t, pa[t + 1]}];

 tb301 = Append[tb301, {t, pB[t + 1]}];

 tb302 = Append[tb302, {pa[t], pB[t]}];

]
```
