# Supplementary material for: Deleterious mutation/epimutation–selection balance with and without inbreeding: a population (epi)genetics model
Source: Genetics. 2024 May 11;227(3):iyae080. doi: 10.1093/genetics/iyae080 (PMC11228854; doi:10.1093/genetics/iyae080)
Supplement: iyae080_Supplementary_Data [file iyae080_supplementary_data.zip › File_S2_GENETICS-2024-306923.pdf]

# S2: Incomplete Dominance

## Introduction

For all cases (A-E), incomplete dominance is assumed. Following the appendices (A1 and A2), we calculate equilibria and perform a local stability analysis for each case. As a complement to the analytical results, numerical results are included. Parameter values are considered that allow for consistent biologically valid equilibria such that all (epi)allele frequencies are between zero and one ($0 \lesssim \hat{p}_a \lesssim 1$, $0 \lesssim \hat{p}_B \lesssim 1$, $0 \lesssim \hat{p}_A \lesssim 1$). Also, parameter values were used that meet the assumptions of the approximation conditions and are within empirically reasonable ranges. The spontaneous epimutation rates ($t_i$), inbreeding coefficient (f), selection coefficients, and the paramutation rate (m), were varied across an empirically reasonable range. Specifically, the forward spontaneous epimutation rate was allowed to vary from (~ $10^{-5}$ - $10^{-3}$) (van der Graaf et al. 2015), and the inbreeding coefficient was varied from random mating to very high levels (~ 0 - 0.95). As paramutation is currently a rarely studied phenomenon, its lower bound rate is not well established, whereas in some empirical cases it occurs at a rate of nearly 100%, and therefore its chosen rate is from the upper end of the forward spontaneous epimutation rate to near 100% (~ $10^{-3}$ - 0.90) (Hollick 2017). Numerical methods are used: to demonstrate the level of accuracy of the analytical approximations, obtain equilibria when the approximate analytical methods did not provide biologically valid solutions, and to help justify statements of local stability conditions when eigenvalue approximations were too complicated for straightforward analytical interpretations. Note: for all tables in File S2 and S3, numerical equilibrium values and eigenvalues were rounded to $10^{-7}$ and the percentage differences of numerical equilibria were rounded to $10^{-2}$.

## Case A: Random Mating (*f* = 0) and without Paramutation (m = 0)

Here we demonstrate the calculations for determining equilibria and performing local stability analysis for Case A of the main text, with complimentary numerical methods. The same methods were used in all cases for the incomplete dominance (this file) and complete dominance (File S3) contexts.

### Determining equilibria (following Appendix section A1 )

Assuming there is no inbreeding or paramutation (f = 0, m = 0), the recursion equations for the deleterious allele and epiallele

respectively are

$$p_B\,' = -\Big(\big((-1+c)\big(-(-1+p_a+p_B)^2\,t_1-(1-p_a-p_B)\,(((1-h\,s)\,p_a+p_B\,(1-h_2\,s_2))\,t_1+p_B\,(-1+h_2\,s_2)\,(-1+t_2))- p_B\,(-p_B\,(1-s_2)-p_a\,(1-h\,s-h_2\,s_2+s_3))\,(-1+t_2)\big)\big)\Big/ \Big(-\big((1-s)\,p_a^2\big)-(-1+p_a+p_B)^2-p_B^2\,(1-s_2)+2\,(1-p_a-p_B)\,(-((1-h\,s)\,p_a)+p_B\,(-1+h_2\,s_2))-2\,p_a\,p_B\,(1-h\,s-h_2\,s_2+s_3)\Big)\Big)$$

$$p_a\,' = \Big(-\big((1-s)\,(-1+z)\,p_a^2\big)-(-1+p_a+p_B)^2\,(-u+(-c+u)\,t_1)-p_B^2\,(1-s_2)\,(-c+(c-u)\,t_2)-p_a\,p_B\,(1-h\,s-h_2\,s_2+s_3)\,(-1-c+z+(c-u)\,t_2)+ (1-p_a-p_B)\,(-((1-h\,s)\,p_a\,(-1-u+z+(-c+u)\,t_1))+p_B\,(1-h_2\,s_2)\,(c+u+(c-u)\,t_1+(-c+u)\,t_2))\Big)\Big/ \Big((1-s)\,p_a^2+(-1+p_a+p_B)^2+p_B^2\,(1-s_2)-2\,(1-p_a-p_B)\,(-((1-h\,s)\,p_a)+p_B\,(-1+h_2\,s_2))+2\,p_a\,p_B\,(1-h\,s-h_2\,s_2+s_3)\Big)$$

Then making substitutions assuming mutation and spontaneous epimutation are of order $\zeta$, and substituting in the equilibrium terms up to the order of approximation (see Appendix section A1 for details),

$$\Big\{0 == -\Big(\big((-1+c)\big(-(-1+p_a+p_B)^2\,t_1-(1-p_a-p_B)\,(((1-h\,s)\,p_a+p_B\,(1-h_2\,s_2))\,t_1+p_B\,(-1+h_2\,s_2)\,(-1+t_2))- p_B\,(-p_B\,(1-s_2)-p_a\,(1-h\,s-h_2\,s_2+s_3))\,(-1+t_2)\big)\big)\Big/\Big(-\big((1-s)\,p_a^2\big)-(-1+p_a+p_B)^2- p_B^2\,(1-s_2)+2\,(1-p_a-p_B)\,(-((1-h\,s)\,p_a)+p_B\,(-1+h_2\,s_2))-2\,p_a\,p_B\,(1-h\,s-h_2\,s_2+s_3)\Big)\Big)-p_B,$$
$$0 == \Big(-\big((1-s)\,(-1+z)\,p_a^2\big)-(-1+p_a+p_B)^2\,(-u+(-c+u)\,t_1)-p_B^2\,(1-s_2)\,(-c+(c-u)\,t_2)-p_a\,p_B\,(1-h\,s-h_2\,s_2+s_3) (-1-c+z+(c-u)\,t_2)+(1-p_a-p_B)\,(-((1-h\,s)\,p_a\,(-1-u+z+(-c+u)\,t_1))+p_B\,(1-h_2\,s_2)\,(c+u+(c-u)\,t_1+(-c+u)\,t_2))\Big)\Big/ \Big((1-s)\,p_a^2+(-1+p_a+p_B)^2+p_B^2\,(1-s_2)-2\,(1-p_a-p_B)\,(-((1-h\,s)\,p_a)+p_B\,(-1+h_2\,s_2))+2\,p_a\,p_B\,(1-h\,s-h_2\,s_2+s_3)\Big)-p_a\Big\}\;/.$$
$$\{p_B\to(p_{B,0}+p_{B,1}\,\zeta),\,p_a\to(p_{a,0}+p_{a,1}\,\zeta),\,t_1\to t_1\,\zeta,\,t_2\to t_2\,\zeta,\;u\to u\,\zeta,\;z\to z\,\zeta,\,c\to c\,\zeta\}$$

Using a Taylor's approximation, with respect to $\zeta$ around the point $\zeta = 0$, including up to first order terms, and then solving for equilibrium terms.

Series[% == 0, {ζ, 0, 1}]

In[ ]:=

$$\text{Solve}\Big[\Big\{\Big(-p_{B,0}+\frac{-((-1+h_2\,s_2)\,p_{B,0}\,(-1+p_{a,0}+p_{B,0}))+p_{B,0}\,((-1+h\,s+h_2\,s_2-s_3)\,p_{a,0}+(-1+s_2)\,p_{B,0})}{-1+2\,h\,s\,p_{a,0}+s\,p_{a,0}^2-2\,h\,s\,p_{a,0}^2+2\,h_2\,s_2\,p_{B,0}-2\,s_3\,p_{a,0}\,p_{B,0}+s_2\,p_{B,0}^2-2\,h_2\,s_2\,p_{B,0}^2}\Big)==0,$$
$$\Big(-p_{a,0}+\frac{-\big((-1+s)\,p_{a,0}^2\big)+(1-h\,s-h_2\,s_2+s_3)\,p_{a,0}\,p_{B,0}+(-1+h\,s)\,p_{a,0}\,(-1+p_{a,0}+p_{B,0})}{1-2\,h\,s\,p_{a,0}-s\,p_{a,0}^2+2\,h\,s\,p_{a,0}^2-2\,h_2\,s_2\,p_{B,0}+2\,s_3\,p_{a,0}\,p_{B,0}-s_2\,p_{B,0}^2+2\,h_2\,s_2\,p_{B,0}^2}\Big)==0\Big\},\{p_{B,0},\,p_{a,0}\}\Big];$$

In[∘]:= $\Big\{\Big(-p_{B,1}+\big(-t_1(-1+p_{a,0}+p_{B,0})^2-(1-p_{a,0}-p_{B,0})(t_1((1-h\,s)\,p_{a,0}+(1-h_2\,s_2)\,p_{B,0})+(-1+h_2\,s_2)(t_2\,p_{B,0}-p_{B,1}))+(-1+h_2\,s_2)\,p_{B,0}(-p_{a,1}-p_{B,1})-((-1+h\,s+h_2\,s_2-s_3)\,p_{a,0}+(-1+s_2)\,p_{B,0})(t_2\,p_{B,0}-p_{B,1})+p_{B,0}((-1+h\,s+h_2\,s_2-s_3)\,p_{a,1}+(-1+s_2)\,p_{B,1})\big)\big/\big(-1+2\,h\,s\,p_{a,0}+s\,p_{a,0}^2-2\,h\,s\,p_{a,0}^2+2\,h_2\,s_2\,p_{B,0}-2\,s_3\,p_{a,0}\,p_{B,0}+s_2\,p_{B,0}^2-2\,h_2\,s_2\,p_{B,0}^2\big)-(-((-1+h_2\,s_2)\,p_{B,0}(-1+p_{a,0}+p_{B,0}))+p_{B,0}((-1+h\,s+h_2\,s_2-s_3)\,p_{a,0}+(-1+s_2)\,p_{B,0}))\Big(\frac{c}{-1+2\,h\,s\,p_{a,0}+s\,p_{a,0}^2-2\,h\,s\,p_{a,0}^2+2\,h_2\,s_2\,p_{B,0}-2\,s_3\,p_{a,0}\,p_{B,0}+s_2\,p_{B,0}^2-2\,h_2\,s_2\,p_{B,0}^2}-(2(-h\,s\,p_{a,1}-s\,p_{a,0}\,p_{a,1}+2\,h\,s\,p_{a,0}\,p_{a,1}+s_3\,p_{a,1}\,p_{B,0}-h_2\,s_2\,p_{B,1}+s_3\,p_{a,0}\,p_{B,1}-s_2\,p_{B,0}\,p_{B,1}+2\,h_2\,s_2\,p_{B,0}\,p_{B,1}))\big/\big(-1+2\,h\,s\,p_{a,0}+s\,p_{a,0}^2-2\,h\,s\,p_{a,0}^2+2\,h_2\,s_2\,p_{B,0}-2\,s_3\,p_{a,0}\,p_{B,0}+s_2\,p_{B,0}^2-2\,h_2\,s_2\,p_{B,0}^2\big)^2\Big)\Big)==0,$

$\big(-p_{a,1}-\big(2\big(-((-1+s)\,p_{a,0}^2)+(1-h\,s-h_2\,s_2+s_3)\,p_{a,0}\,p_{B,0}+(-1+h\,s)\,p_{a,0}(-1+p_{a,0}+p_{B,0})\big)(-h\,s\,p_{a,1}-s\,p_{a,0}\,p_{a,1}+2\,h\,s\,p_{a,0}\,p_{a,1}+s_3\,p_{a,1}\,p_{B,0}-h_2\,s_2\,p_{B,1}+s_3\,p_{a,0}\,p_{B,1}-s_2\,p_{B,0}\,p_{B,1}+2\,h_2\,s_2\,p_{B,0}\,p_{B,1})\big)\big/\big(1-2\,h\,s\,p_{a,0}-s\,p_{a,0}^2+2\,h\,s\,p_{a,0}^2-2\,h_2\,s_2\,p_{B,0}+2\,s_3\,p_{a,0}\,p_{B,0}-s_2\,p_{B,0}^2+2\,h_2\,s_2\,p_{B,0}^2\big)^2+\big((-1+s)\big(z\,p_{a,0}^2-2\,p_{a,0}\,p_{a,1}\big)-c(-1+s_2)\,p_{B,0}^2+u(-1+p_{a,0}+p_{B,0})^2+(1-p_{a,0}-p_{B,0})((1-h\,s)(-((-u+z)\,p_{a,0})+p_{a,1})+(c+u)(1-h_2\,s_2)\,p_{B,0})+(1-h\,s)\,p_{a,0}(-p_{a,1}-p_{B,1})+(1-h\,s-h_2\,s_2+s_3)(-(((-c+z)\,p_{a,0}-p_{a,1})\,p_{B,0})+p_{a,0}\,p_{B,1})\big)\big/\big(1-2\,h\,s\,p_{a,0}-s\,p_{a,0}^2+2\,h\,s\,p_{a,0}^2-2\,h_2\,s_2\,p_{B,0}+2\,s_3\,p_{a,0}\,p_{B,0}-s_2\,p_{B,0}^2+2\,h_2\,s_2\,p_{B,0}^2\big)\big)==0\Big\}\;/.\;\{p_{B,0}\to 0,\,p_{a,0}\to 0\};$

In[∘]:= Solve[%, $\{p_{B,1}, p_{a,1}\}$];

Incorporating zeroth order and first order terms together, and then substituting in the original parameters ($t_1\,\zeta$- > $t_1$, $u\,\zeta$ -> u ...),

the first order approximation is that of eq. 1 of the main text:

$$\hat{p}_a \sim \frac{u}{h\,s}+O[\zeta^2]$$

$$\hat{p}_B \sim \frac{t_1}{h_2\,s_2}+O[\zeta^2]$$

$$\hat{p}_A \sim 1-\frac{u}{h\,s}-\frac{t_1}{h_2\,s_2}+O[\zeta^2]$$

Next evaluating the accuracy of the analytical approximation in comparison to numerical equilibria solutions (Table A1 and A2).

Table A1: Evaluating the accuracy of the equilibrium approximation for the deleterious allele as the mutation rate of the wild-type allele increases from a lower to upper limit

| u | ~ $\hat{p}_a$ Analytical | $\hat{p}_a$ Numerical | Percentage Difference |
|---|---|---|---|
| $1. \times 10^{-9}$ | $1. \times 10^{-7}$ | $1. \times 10^{-7}$ | 0 |
| $1.1 \times 10^{-8}$ | $1.1 \times 10^{-6}$ | $1.1 \times 10^{-6}$ | 0 |
| $2.1 \times 10^{-8}$ | $2.1 \times 10^{-6}$ | $2.1 \times 10^{-6}$ | 0 |
| $3.1 \times 10^{-8}$ | $3.1 \times 10^{-6}$ | $3.1 \times 10^{-6}$ | 0 |
| $4.1 \times 10^{-8}$ | $4.1 \times 10^{-6}$ | $4.1 \times 10^{-6}$ | 0 |
| $5.1 \times 10^{-8}$ | $5.1 \times 10^{-6}$ | $5.1 \times 10^{-6}$ | 0 |
| $6.1 \times 10^{-8}$ | $6.1 \times 10^{-6}$ | $6.1 \times 10^{-6}$ | 0 |
| $7.1 \times 10^{-8}$ | $7.1 \times 10^{-6}$ | $7.1 \times 10^{-6}$ | 0 |
| $8.1 \times 10^{-8}$ | $8.1 \times 10^{-6}$ | $8.1 \times 10^{-6}$ | 0 |
| $9.1 \times 10^{-8}$ | $9.1 \times 10^{-6}$ | $9.1 \times 10^{-6}$ | 0 |

Parameter values: s = 0.02, $s_2$ = 0.01, $s_3$ = 0.001, h = 0.5, $h_2$ = 0.5,
$t_1$ = 10 ^ −5, $t_2$ = 10 ^ −4, c = 10 ^ −9, z = 10 ^ −9

Table A2: Evaluating the accuracy of the equilibrium approximation for the epiallele as the forward spontaneous epimutation rate increases from a lower to upper limit

| $t_1$ | ~ $\hat{p}_B$ Analytical | $\hat{p}_B$ Numerical | Percentage Difference |
|---|---|---|---|
| 0.00001 | 0.0004 | 0.000398 | 0.4 |
| 0.00011 | 0.0044 | 0.004382 | 0.4 |
| 0.00021 | 0.0084 | 0.008365 | 0.41 |
| 0.00031 | 0.0124 | 0.012347 | 0.42 |
| 0.00041 | 0.0164 | 0.016329 | 0.44 |
| 0.00051 | 0.0204 | 0.020309 | 0.45 |
| 0.00061 | 0.0244 | 0.024288 | 0.46 |
| 0.00071 | 0.0284 | 0.028267 | 0.47 |
| 0.00081 | 0.0324 | 0.032244 | 0.48 |
| 0.00091 | 0.0364 | 0.036221 | 0.49 |
| 0.00101 | 0.0404 | 0.040196 | 0.5 |

Parameter values: s = 0.02, $s_2$ = 0.05, $s_3$ = 0.001, h = 0.5, $h_2$ = 0.5,
u = 10 ^ −9, $t_2$ = 10 ^ −4, c = 10 ^ −9, z = 10 ^ −9

Evaluating the accuracy of the analytical approximation (eq. 2 of the main text) in comparison to numerical equilibria solutions (Tables A3 and A4):

Table A3: Evaluating the accuracy of the equilibrium approximation for the deleterious allele as the mutation rate of the wild-type allele increases from a lower to upper limit

| u | ~ $\hat{p}_a$ Analytical | $\hat{p}_a$ Numerical | Percentage Difference |
|---|---|---|---|
| $1. \times 10^{-9}$ | $1. \times 10^{-7}$ | $1. \times 10^{-7}$ | 0 |
| $1.1 \times 10^{-8}$ | $1.1 \times 10^{-6}$ | $1.1 \times 10^{-6}$ | 0 |
| $2.1 \times 10^{-8}$ | $2.1 \times 10^{-6}$ | $2.1 \times 10^{-6}$ | 0 |
| $3.1 \times 10^{-8}$ | $3.1 \times 10^{-6}$ | $3.1 \times 10^{-6}$ | 0 |
| $4.1 \times 10^{-8}$ | $4.1 \times 10^{-6}$ | $4.1 \times 10^{-6}$ | 0 |
| $5.1 \times 10^{-8}$ | $5.1 \times 10^{-6}$ | $5.1 \times 10^{-6}$ | 0 |
| $6.1 \times 10^{-8}$ | $6.1 \times 10^{-6}$ | $6.1 \times 10^{-6}$ | 0 |
| $7.1 \times 10^{-8}$ | $7.1 \times 10^{-6}$ | $7.1 \times 10^{-6}$ | 0 |
| $8.1 \times 10^{-8}$ | $8.1 \times 10^{-6}$ | $8.1 \times 10^{-6}$ | 0 |
| $9.1 \times 10^{-8}$ | $9.1 \times 10^{-6}$ | $9.1 \times 10^{-6}$ | 0 |

Parameter values: $s = 0.02$, $s_2 = 0.01$, $s_3 = 0.001$, $h = 0.5$, $h_2 = 0.5$, $t_1 = 10^{-5}$, $t_2 = 0.2$, $c = 10^{-9}$, $z = 10^{-9}$

Table A4: Evaluating the accuracy of the equilibrium approximation for the epiallele as the forward spontaneous epimutation rate increases from a lower to upper limit

| $t_1$ | ~ $\hat{p}_B$ Analytical | $\hat{p}_B$ Numerical | Percentage Difference |
|---|---|---|---|
| 0.00001 | 0.000045 | 0.000045 | 0. |
| 0.00011 | 0.0005 | 0.0005 | 0. |
| 0.00021 | 0.000955 | 0.000954 | 0.1 |
| 0.00031 | 0.001409 | 0.001407 | 0.14 |
| 0.00041 | 0.001864 | 0.001861 | 0.16 |
| 0.00051 | 0.002318 | 0.002313 | 0.22 |
| 0.00061 | 0.002773 | 0.002766 | 0.25 |
| 0.00071 | 0.003227 | 0.003218 | 0.28 |
| 0.00081 | 0.003682 | 0.00367 | 0.33 |
| 0.00091 | 0.004136 | 0.004121 | 0.36 |
| 0.00101 | 0.004591 | 0.004572 | 0.41 |

Parameter values: $s = 0.02$, $s_2 = 0.05$, $s_3 = 0.001$, $h = 0.5$, $h_2 = 0.5$, $u = 10^{-9}$, $t_2 = 0.2$, $u = 10^{-9}$, $c = 10^{-9}$, $z = 10^{-9}$

## Local Stability analysis (following Appendix Section A2 )

Calculating the Jacobian matrix for the recursion equations and then obtaining the approximated characteristic equation. This is followed by substituting in explicit equilibrium terms into the characteristic equation

$(p_{a,0} \to 0,\ p_{B,0} \to 0,\ p_{a,1} \to \frac{u}{h\,s},\ p_{B,1} \to \frac{t_1}{h_2\,s_2})$, and solving for $\lambda_0$:

In[ ]:= Solve[$(1 - h\,s - \lambda_0)\,(1 - h_2\,s_2 - \lambda_0) == 0,\ \lambda_0$]

Out[ ]=

$\{\{\lambda_0 \to 1 - h\,s\},\ \{\lambda_0 \to 1 - h_2\,s_2\}\}$

Using the $\lambda_0$ terms to solve for the corresponding $\lambda_1$ terms, and then back substituting the original parameters and simplifying.

The approximated eigenvalues are

$$\lambda_{(1)} \sim 1 - h\,s + \left(5 - \frac{2}{h} - 3\,h\,s\right) u + (-1 + h\,s)\,z + \left(1 - 2\,h\,s + \frac{s_3}{h_2\,s_2}\right) t_1 + O\left[\zeta^2\right]$$

$$\lambda_{(2)} \sim 1 - h_2\,s_2 - c + u + \frac{u\,s_3}{h\,s} + 5\,t_1 - \frac{2\,t_1}{h_2} - t_2 + h_2\,s_2\,(c - 2\,u - 3\,t_1 + t_2) + O\left[\zeta^2\right]$$

Numerically evaluating approximated eigenvalues for local stability such that $|\lambda_{(1)}| < 1$ and $|\lambda_{(2)}| < 1$, where key parameters

were varied across an empirically reasonable range:

Table A5: Numerically evaluating the approximated eigenvalues to determine local stability as the mutation rate of the wild-type allele varies across a given range

| u | $\sim\lambda_{(1)}$ | $\sim\lambda_{(2)}$ | Local Stability |
|---|---|---|---|
| $1. \times 10^{-9}$ | 0.990012 | 0.99491 | Stable |
| $1.1 \times 10^{-8}$ | 0.990012 | 0.99491 | Stable |
| $2.1 \times 10^{-8}$ | 0.990012 | 0.99491 | Stable |
| $3.1 \times 10^{-8}$ | 0.990012 | 0.99491 | Stable |
| $4.1 \times 10^{-8}$ | 0.990012 | 0.99491 | Stable |
| $5.1 \times 10^{-8}$ | 0.990012 | 0.99491 | Stable |
| $6.1 \times 10^{-8}$ | 0.990012 | 0.99491 | Stable |
| $7.1 \times 10^{-8}$ | 0.990012 | 0.99491 | Stable |
| $8.1 \times 10^{-8}$ | 0.990012 | 0.99491 | Stable |
| $9.1 \times 10^{-8}$ | 0.990012 | 0.99491 | Stable |

Parameter values: $s = 0.02$, $s_2 = 0.01$, $s_3 = 0.001$,
$h = 0.5$, $h_2 = 0.5$, $t_1 = 10$ ^ −5, $t_2 = 10$ ^ −4,
c = 10 ^ −9, z = 10 ^ −9

Table A6: Numerically evaluating the approximated eigenvalues to determine local stability as the forward spontaneous epimutation rate varies across a given range

| $t_1$ | $\sim\lambda_{(1)}$ | $\sim\lambda_{(2)}$ | Local Stability |
|---|---|---|---|
| 0.00001 | 0.99001 | 0.974912 | Stable |
| 0.00011 | 0.990112 | 0.975004 | Stable |
| 0.00021 | 0.990214 | 0.975097 | Stable |
| 0.00031 | 0.990316 | 0.975189 | Stable |
| 0.00041 | 0.990418 | 0.975282 | Stable |
| 0.00051 | 0.99052 | 0.975374 | Stable |
| 0.00061 | 0.990622 | 0.975467 | Stable |
| 0.00071 | 0.990724 | 0.975559 | Stable |
| 0.00081 | 0.990826 | 0.975652 | Stable |
| 0.00091 | 0.990928 | 0.975744 | Stable |
| 0.00101 | 0.99103 | 0.975837 | Stable |

Parameter values: $s = 0.02$, $s_2 = 0.05$, $s_3 = 0.001$,
$h = 0.5$, $h_2 = 0.5$, u = 10 ^ −9, $t_2 = 10$ ^ −4,
c = 10 ^ −9, z = 10 ^ −9

Evaluation of local stability through numerical simulations of the recursion equations (Figure A1). Evidence for local stability was supported through simulations of the recursion equations beginning above (gray) and below (black line) the equilibrium (dashed

line).

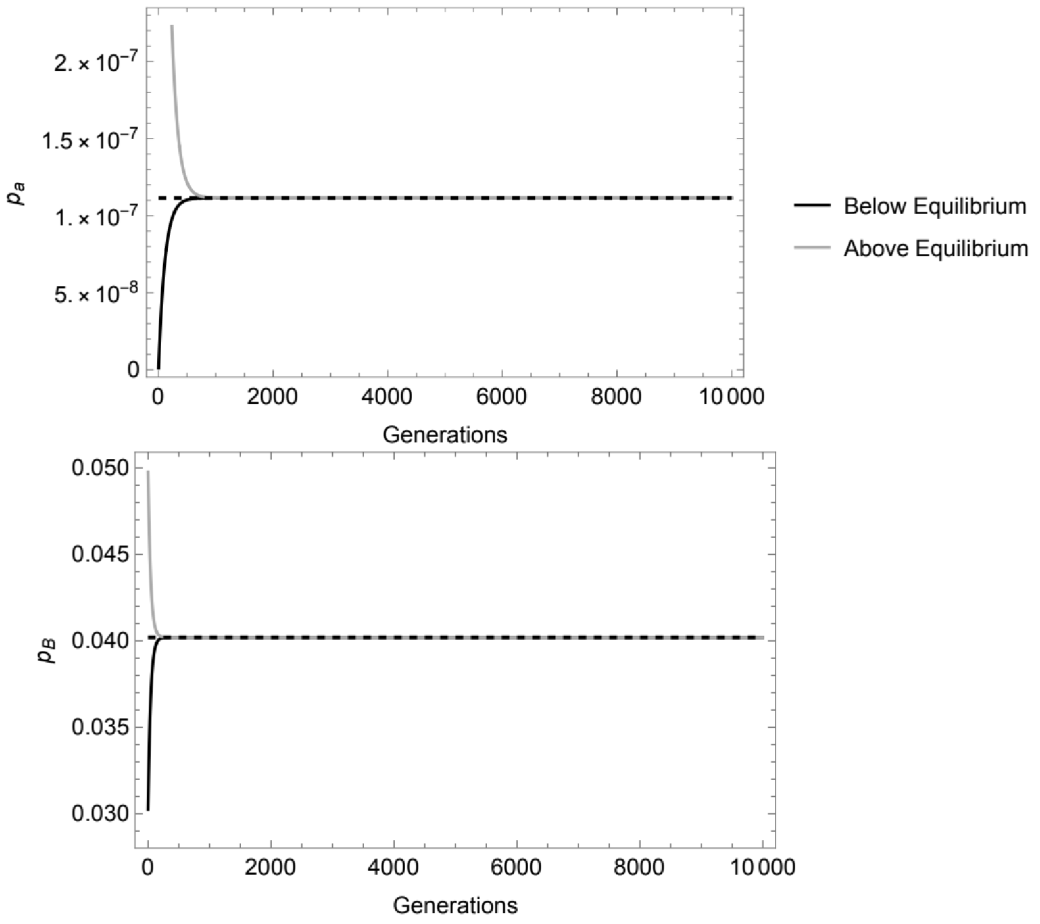


Figure A1: Plots of the deleterious allele frequency (top) and the epiallele frequency (bottom) across time in generations. The numerical parameter values are $s = 0.02$, $s_2 = 0.05$, $s_3 = 0.001$, $h = 0.5$, $h_2 = 0.5$, $u = 10^{-9}$, $t_1 = 0.00101$, $u \to 10^{-9}$, $t_2 = 10^{-4}$, $c = 10^{-9}$, $z = 10^{-9}$

The approximated eigenvalues corresponding for eq. 2 of the main text are

$$\lambda_{(1)} \sim 1 - h\,s + \frac{1}{h\,(h_2\,s_2\,(-1+t_2)-t_2)} \left(-h\,s_3\,t_1 + h_2\,s_2\left(h\,(-1+2\,h\,s)\,t_1 - \left(\left(2-5\,h+3\,h^2\,s\right)u + h\,(1-h\,s)\,z\right)(-1+t_2)\right) + \left(\left(2-5\,h+3\,h^2\,s\right)u + h\,(1-h\,s)\,z\right)t_2\right) + O\left[\zeta^2\right]$$

$$\lambda_{(2)} \sim (-1+h_2\,s_2)\,(-1+t_2) + \frac{1}{h\,s\,(h_2\,s_2\,(-1+t_2)-t_2)} \left(-2\,h\,s\,s_2\,t_1\,(-1+t_2) - h\,s\,h_2^2\,s_2^2\,(3\,t_1+(c-2\,u)\,(-1+t_2))\,(-1+t_2) + (h\,s\,(t_1-(c-u)\,(-1+t_2)) + u\,s_3\,(-1+t_2))\,t_2 + h_2\,s_2\left(-u\,s_3\,(-1+t_2)^2 + h\,s\,(t_1\,(-5+4\,t_2) + (-1+t_2)\,(-c+u+(2\,c-3\,u)\,t_2))\right)\right) + O\left[\zeta^2\right]$$

Numerically evaluating the approximated eigenvalues for local stability such that $|\lambda_{(1)}| < 1$ and $|\lambda_{(2)}| < 1$ (Tables A7 and A8):

Table A7: Numerically evaluating the approximated eigenvalues to determine local stability as the mutation rate of the wild-type allele varies across a given range

| u | $\sim\lambda_{(1)}$ | $\sim\lambda_{(2)}$ | Local Stability |
|---|---|---|---|
| $1. \times 10^{-9}$ | 0.99 | 0.79599 | Stable |
| $1.1 \times 10^{-8}$ | 0.99 | 0.79599 | Stable |
| $2.1 \times 10^{-8}$ | 0.99 | 0.79599 | Stable |
| $3.1 \times 10^{-8}$ | 0.99 | 0.79599 | Stable |
| $4.1 \times 10^{-8}$ | 0.99 | 0.79599 | Stable |
| $5.1 \times 10^{-8}$ | 0.99 | 0.79599 | Stable |
| $6.1 \times 10^{-8}$ | 0.99 | 0.79599 | Stable |
| $7.1 \times 10^{-8}$ | 0.99 | 0.79599 | Stable |
| $8.1 \times 10^{-8}$ | 0.99 | 0.795991 | Stable |
| $9.1 \times 10^{-8}$ | 0.99 | 0.795991 | Stable |

Parameter values: $s = 0.02$, $s_2 = 0.01$, $s_3 = 0.001$,
$h = 0.5$, $h_2 = 0.5$, $t_1 = 10^{\wedge}-5$, $t_2 = 0.2$, $c = 10^{\wedge}-9$,
$z = 10^{\wedge}-9$

Table A8: Numerically evaluating the approximated eigenvalues to determine local stability as the forward spontaneous epimutation rate varies across a given range

| $t_1$ | $\sim\lambda_{(1)}$ | $\sim\lambda_{(2)}$ | Local Stability |
|---|---|---|---|
| 0.00001 | 0.990001 | 0.779992 | Stable |
| 0.00011 | 0.990013 | 0.779912 | Stable |
| 0.00021 | 0.990024 | 0.779832 | Stable |
| 0.00031 | 0.990036 | 0.779751 | Stable |
| 0.00041 | 0.990048 | 0.779671 | Stable |
| 0.00051 | 0.990059 | 0.779591 | Stable |
| 0.00061 | 0.990071 | 0.779511 | Stable |
| 0.00071 | 0.990082 | 0.77943 | Stable |
| 0.00081 | 0.990094 | 0.77935 | Stable |
| 0.00091 | 0.990105 | 0.77927 | Stable |
| 0.00101 | 0.990117 | 0.77919 | Stable |

Parameter values: $s = 0.02$, $s_2 = 0.05$, $s_3 = 0.001$,
$h = 0.5$, $h_2 = 0.5$, $u = 10^{\wedge}-9$, $t_2 = 0.2$, $u = 10^{\wedge}-9$,
$c = 10^{\wedge}-9$, $z = 10^{\wedge}-9$

Evaluation of local stability through numerical simulations of the recursion equations (Figure A2). Evidence for local stability was supported through simulations of the recursion equations beginning above (gray line) and below (black line) the equilibrium (dashed line).

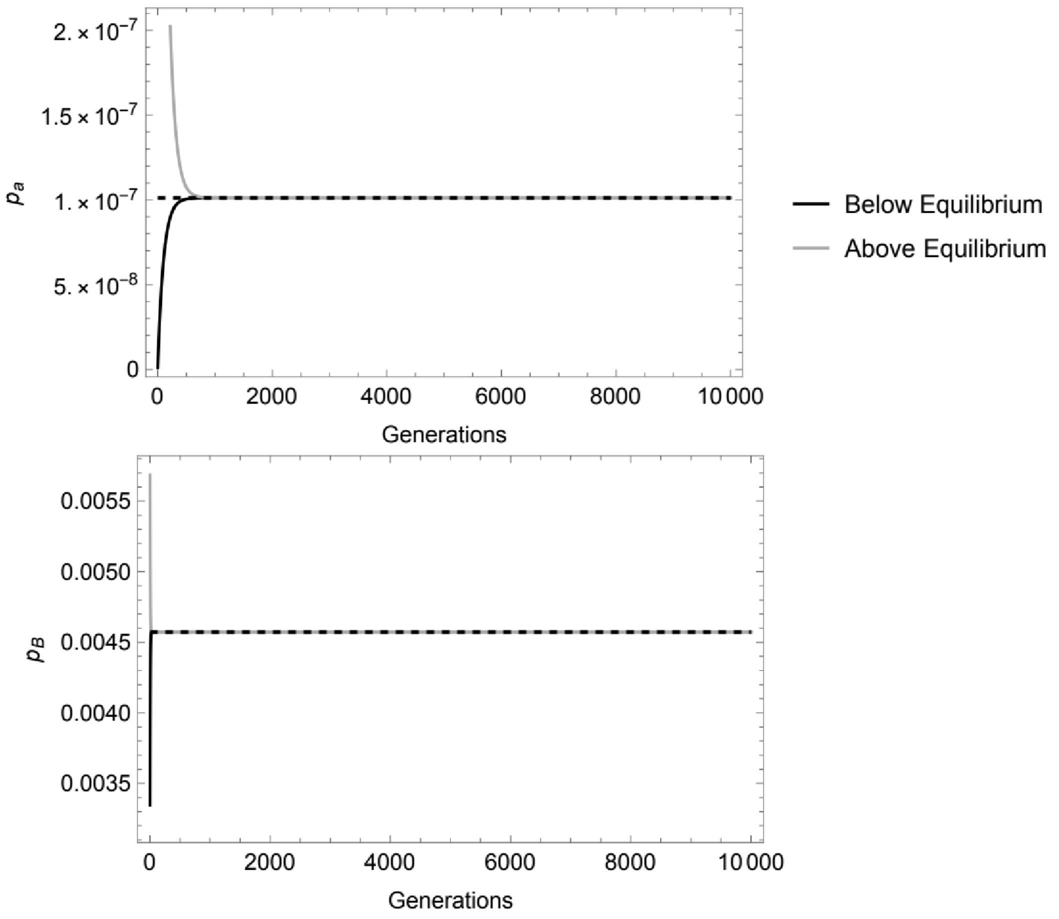


Figure A2: Plots of the deleterious allele frequency (top) and the epiallele frequency (bottom) across time in generations. The numerical parameter values are
$s = 0.02$, $s_2 = 0.05$, $s_3 = 0.001$, $h = 0.5$, $h_2 = 0.5$, $u = 10^{-9}$, $t_1 = 0.00101$, $t_2 = 0.2$, $c = 10^{-9}$, $z = 10^{-9}$

# Case B: With Inbreeding ($f > 0$) and without Paramutation ($m = 0$)

Evaluating the accuracy of the analytical approximation (eq. 3 of the main text) in comparison to numerical equilibria solutions (Tables B1-B4):

Table B1: Evaluating the accuracy of the equilibrium approximation for the deleterious allele as the mutation rate of the wild-type allele increases from a lower to upper limit

| $u$ | $\sim \hat{p}_a$ Analytical | $\hat{p}_a$ Numerical | Percentage Difference |
|---|---|---|---|
| $1.\times 10^{-9}$ | $1.\times 10^{-7}$ | $1.\times 10^{-7}$ | 0 |
| $1.1\times 10^{-8}$ | $7.\times 10^{-7}$ | $7.\times 10^{-7}$ | 0 |
| $2.1\times 10^{-8}$ | $1.4\times 10^{-6}$ | $1.4\times 10^{-6}$ | 0 |
| $3.1\times 10^{-8}$ | $2.1\times 10^{-6}$ | $2.1\times 10^{-6}$ | 0 |
| $4.1\times 10^{-8}$ | $2.7\times 10^{-6}$ | $2.7\times 10^{-6}$ | 0 |
| $5.1\times 10^{-8}$ | $3.4\times 10^{-6}$ | $3.4\times 10^{-6}$ | 0 |
| $6.1\times 10^{-8}$ | $4.1\times 10^{-6}$ | $4.1\times 10^{-6}$ | 0 |
| $7.1\times 10^{-8}$ | $4.7\times 10^{-6}$ | $4.7\times 10^{-6}$ | 0 |
| $8.1\times 10^{-8}$ | $5.4\times 10^{-6}$ | $5.4\times 10^{-6}$ | 0 |
| $9.1\times 10^{-8}$ | $6.1\times 10^{-6}$ | $6.1\times 10^{-6}$ | 0 |

Parameter values: $f = 0.5$, $s = 0.02$, $s_2 = 0.01$, $s_3 = 0.001$, $h = 0.5$, $h_2 = 0.5$, $t_1 = 10^{-5}$, $t_2 = 10^{-4}$, $c = 10^{-9}$, $z = 10^{-9}$

Table B2: Evaluating the accuracy of the equilibrium approximation for the epiallele as the forward spontaneous epimutation rate increases from a lower to upper limit

| $t_1$ | $\sim \hat{p}_B$ Analytical | $\hat{p}_B$ Numerical | Percentage Difference |
|---|---|---|---|
| 0.00001 | 0.0013333 | 0.0013159 | 1.31 |
| 0.00011 | 0.0146667 | 0.0144718 | 1.33 |
| 0.00021 | 0.028 | 0.0276222 | 1.35 |
| 0.00031 | 0.0413333 | 0.0407668 | 1.37 |
| 0.00041 | 0.0546667 | 0.0539054 | 1.39 |
| 0.00051 | 0.068 | 0.0670379 | 1.41 |
| 0.00061 | 0.0813333 | 0.080164 | 1.44 |
| 0.00071 | 0.0946667 | 0.0932835 | 1.46 |
| 0.00081 | 0.108 | 0.106396 | 1.49 |
| 0.00091 | 0.121333 | 0.119502 | 1.51 |
| 0.00101 | 0.134667 | 0.1326 | 1.53 |

Parameter values: $f = 0.5$, $s = 0.02$, $s_2 = 0.01$, $s_3 = 0.001$, $h = 0.5$, $h_2 = 0.5$, $u = 10^{-9}$, $t_2 = 10^{-4}$, $c = 10^{-9}$, $z = 10^{-9}$

Table B3: Evaluating the accuracy of the equilibrium approximation for the deleterious allele as the inbreeding coefficient varies from a lower to upper limit

| $f$ | $\sim \hat{p}_a$ Analytical | $\hat{p}_a$ Numerical | Percentage Difference |
|---|---|---|---|
| 0.01 | $1.\times 10^{-7}$ | $1.\times 10^{-7}$ | 0 |
| 0.11 | $1.\times 10^{-7}$ | $1.\times 10^{-7}$ | 0 |
| 0.21 | $1.\times 10^{-7}$ | $1.\times 10^{-7}$ | 0 |
| 0.31 | $1.\times 10^{-7}$ | $1.\times 10^{-7}$ | 0 |
| 0.41 | $1.\times 10^{-7}$ | $1.\times 10^{-7}$ | 0 |
| 0.51 | $1.\times 10^{-7}$ | $1.\times 10^{-7}$ | 0 |
| 0.61 | $1.\times 10^{-7}$ | $1.\times 10^{-7}$ | 0 |
| 0.71 | $1.\times 10^{-7}$ | $1.\times 10^{-7}$ | 0 |
| 0.81 | $1.\times 10^{-7}$ | $1.\times 10^{-7}$ | 0 |
| 0.91 | $1.\times 10^{-7}$ | $1.\times 10^{-7}$ | 0 |

Parameter values: $s = 0.02$, $s_2 = 0.01$, $s_3 = 0.001$, $h = 0.5$, $h_2 = 0.5$, $u = 10^{-9}$, $t_1 = 10^{-5}$, $t_2 = 10^{-4}$, $c = 10^{-9}$, $z = 10^{-9}$

Table B4: Evaluating the accuracy of the equilibrium approximation for the epiallele as the inbreeding coefficient varies from a lower to upper limit

| f | ~ $\hat{p}_B$ Analytical | $\hat{p}_B$ Numerical | Percentage Difference |
|---|---|---|---|
| 0.01 | 0.0019802 | 0.0019418 | 1.94 |
| 0.11 | 0.0018018 | 0.00177 | 1.76 |
| 0.21 | 0.0016529 | 0.0016261 | 1.62 |
| 0.31 | 0.0015267 | 0.0015039 | 1.49 |
| 0.41 | 0.0014184 | 0.0013987 | 1.39 |
| 0.51 | 0.0013245 | 0.0013073 | 1.3 |
| 0.61 | 0.0012422 | 0.0012271 | 1.22 |
| 0.71 | 0.0011696 | 0.0011562 | 1.15 |
| 0.81 | 0.001105 | 0.001093 | 1.09 |
| 0.91 | 0.0010471 | 0.0010364 | 1.02 |

Parameter values: $s = 0.02$, $s_2 = 0.01$, $s_3 = 0.001$, $h = 0.5$, $h_2 = 0.5$,
$u = 10^{-9}$, $t_1 = 10^{-5}$, $t_2 = 10^{-4}$, $c = 10^{-9}$,
$z = 10^{-9}$

Evaluating the accuracy of the analytical approximation (eq. 4 of the main text) in comparison to numerical equilibria solutions

(Tables B5-B8):

Table B5: Evaluating the accuracy of the equilibrium approximation for the deleterious allele as the mutation rate of the wild-type allele increases from a lower to upper limit

| u | ~ $\hat{p}_a$ Analytical | $\hat{p}_a$ Numerical | Percentage Difference |
|---|---|---|---|
| $1. \times 10^{-9}$ | $1. \times 10^{-7}$ | $1. \times 10^{-7}$ | 0 |
| $1.1 \times 10^{-8}$ | $7. \times 10^{-7}$ | $7. \times 10^{-7}$ | 0 |
| $2.1 \times 10^{-8}$ | $1.4 \times 10^{-6}$ | $1.4 \times 10^{-6}$ | 0 |
| $3.1 \times 10^{-8}$ | $2.1 \times 10^{-6}$ | $2.1 \times 10^{-6}$ | 0 |
| $4.1 \times 10^{-8}$ | $2.7 \times 10^{-6}$ | $2.7 \times 10^{-6}$ | 0 |
| $5.1 \times 10^{-8}$ | $3.4 \times 10^{-6}$ | $3.4 \times 10^{-6}$ | 0 |
| $6.1 \times 10^{-8}$ | $4.1 \times 10^{-6}$ | $4.1 \times 10^{-6}$ | 0 |
| $7.1 \times 10^{-8}$ | $4.7 \times 10^{-6}$ | $4.7 \times 10^{-6}$ | 0 |
| $8.1 \times 10^{-8}$ | $5.4 \times 10^{-6}$ | $5.4 \times 10^{-6}$ | 0 |
| $9.1 \times 10^{-8}$ | $6.1 \times 10^{-6}$ | $6.1 \times 10^{-6}$ | 0 |

Parameter values: $f = 0.5$, $s = 0.02$, $s_2 = 0.01$, $s_3 = 0.001$, $h = 0.5$,
$h_2 = 0.5$, $t_1 = 10^{-5}$, $t_2 = 0.2$, $c = 10^{-9}$, $z = 10^{-9}$

Table B6: Evaluating the accuracy of the equilibrium approximation for the epiallele as the forward spontaneous epimutation rate increases from a lower to upper limit

| $t_1$ | ~ $\hat{p}_B$ Analytical | $\hat{p}_B$ Numerical | Percentage Difference |
|---|---|---|---|
| 0.00001 | 0.0000485 | 0.0000485 | 0. |
| 0.00011 | 0.000534 | 0.0005337 | 0.06 |
| 0.00021 | 0.0010194 | 0.0010184 | 0.1 |
| 0.00031 | 0.0015049 | 0.0015027 | 0.15 |
| 0.00041 | 0.0019903 | 0.0019865 | 0.19 |
| 0.00051 | 0.0024757 | 0.0024698 | 0.24 |
| 0.00061 | 0.0029612 | 0.0029527 | 0.29 |
| 0.00071 | 0.0034466 | 0.0034352 | 0.33 |
| 0.00081 | 0.003932 | 0.0039172 | 0.38 |
| 0.00091 | 0.0044175 | 0.0043987 | 0.43 |
| 0.00101 | 0.0049029 | 0.0048799 | 0.47 |

Parameter values: f = 0.5, s = 0.02, $s_2$ = 0.01, $s_3$ = 0.001, h = 0.5,
$h_2$ = 0.5, u = 10 ^ −9, $t_2$ = 0.2, c = 10 ^ −9, z = 10 ^ −9

Table B7: Evaluating the accuracy of the equilibrium approximation for the deleterious allele as the inbreeding coefficient varies from a lower to upper limit

| f | ~ $\hat{p}_a$ Analytical | $\hat{p}_a$ Numerical | Percentage Difference |
|---|---|---|---|
| 0.01 | $1. \times 10^{-7}$ | $1. \times 10^{-7}$ | 0 |
| 0.11 | $1. \times 10^{-7}$ | $1. \times 10^{-7}$ | 0 |
| 0.21 | $1. \times 10^{-7}$ | $1. \times 10^{-7}$ | 0 |
| 0.31 | $1. \times 10^{-7}$ | $1. \times 10^{-7}$ | 0 |
| 0.41 | $1. \times 10^{-7}$ | $1. \times 10^{-7}$ | 0 |
| 0.51 | $1. \times 10^{-7}$ | $1. \times 10^{-7}$ | 0 |
| 0.61 | $1. \times 10^{-7}$ | $1. \times 10^{-7}$ | 0 |
| 0.71 | $1. \times 10^{-7}$ | $1. \times 10^{-7}$ | 0 |
| 0.81 | $1. \times 10^{-7}$ | $1. \times 10^{-7}$ | 0 |
| 0.91 | $1. \times 10^{-7}$ | $1. \times 10^{-7}$ | 0 |

Parameter values: s = 0.02, $s_2$ = 0.01, $s_3$ = 0.001, h = 0.5,
$h_2$ = 0.5, $t_1$ = 10 ^ −5, $t_2$ = 0.2, u = 10 ^ −9,
c = 10 ^ −9, z = 10 ^ −9

Table B8: Evaluating the accuracy of the equilibrium approximation for the epiallele as the inbreeding coefficient varies from a lower to upper limit

| f | ~ $\hat{p}_B$ Analytical | $\hat{p}_B$ Numerical | Percentage Difference |
|---|---|---|---|
| 0.01 | 0.000049 | 0.000049 | 0 |
| 0.11 | 0.0000489 | 0.0000489 | 0 |
| 0.21 | 0.0000488 | 0.0000488 | 0 |
| 0.31 | 0.0000487 | 0.0000487 | 0 |
| 0.41 | 0.0000486 | 0.0000486 | 0 |
| 0.51 | 0.0000485 | 0.0000485 | 0 |
| 0.61 | 0.0000484 | 0.0000484 | 0 |
| 0.71 | 0.0000483 | 0.0000483 | 0 |
| 0.81 | 0.0000483 | 0.0000483 | 0 |
| 0.91 | 0.0000482 | 0.0000482 | 0 |

Parameter values: s = 0.02, $s_2$ = 0.01, $s_3$ = 0.001, h = 0.5, $h_2$ = 0.5,
$t_1$ = 10 ^ −5, $t_2$ = 0.2, u = 10 ^ −9, c = 10 ^ −9,
z = 10 ^ −9

## Local Stability analysis (following Appendix Section A2 )

The approximated eigenvalues corresponding with eq. 3 of the main text are

$$\lambda_{(1)} \sim 1 - f\,s - h\,s + f\,h\,s + \Big((-1+f)\,(f\,(-1+h)-h)\,s_3\,t_1 + s_2\,\big((-1+f)\,h_2\,\big(\big(2-5\,h+3\,h^2\,s+f^2\,\big(1-4\,h+3\,h^2\big)\,s+f\,\big(-3+5\,h+4\,h\,s-6\,h^2\,s\big)\big)\,u-(f\,(-1+h)-h)\,(1+f\,(-1+h)\,s-h\,s)\,z+(f\,(-1+h)-h)\,(1+2\,f\,(-1+h)\,s-2\,h\,s)\,t_1\big) - f\,\big(\big(2-5\,h+3\,h^2\,s+f^2\,\big(1-4\,h+3\,h^2\big)\,s+f\,\big(-3+5\,h+4\,h\,s-6\,h^2\,s\big)\big)\,u-(f\,(-1+h)-h)\,(1+f\,(-1+h)\,s-h\,s)\,z+(f\,(-1+h)-h)\,(1+f\,(-1+h)\,s-h\,s)\,t_1\big)\big)\Big)\Big/\big((f\,(-1+h)-h)\,(-f+(-1+f)\,h_2)\,s_2\big)+O\big[\zeta^2\big]$$

$$\lambda_{(2)} \sim 1 - f\,s_2 - h_2\,s_2 + f\,h_2\,s_2 + \Big(-c\,f^2\,s-c\,f\,h\,s+c\,f^2\,h\,s+f^2\,s\,u+f\,h\,s\,u-f^2\,h\,s\,u+f\,u\,s_3-f^2\,u\,s_3-2\,f\,s\,t_1+3\,f^2\,s\,t_1-2\,h\,s\,t_1+5\,f\,h\,s\,t_1-3\,f^2\,h\,s\,t_1-f^2\,s\,t_2-f\,h\,s\,t_2+f^2\,h\,s\,t_2-(-1+f)^2\,s\,h_2^2\,s_2\,(-c\,f-c\,h+c\,f\,h+f\,u+2\,h\,u-2\,f\,h\,u+3\,(f+h-f\,h)\,t_1+(f\,(-1+h)-h)\,t_2)+f^2\,s\,s_2\,(c\,f+c\,h-c\,f\,h-f\,u-2\,h\,u+2\,f\,h\,u+(f\,(-1+h)-h)\,t_1+(f+h-f\,h)\,t_2)+(-1+f)\,h_2\,((-1+f)\,u\,s_3-(f\,(-1+h)-h)\,s\,(c-u-5\,t_1+t_2)+2\,f\,s\,s_2\,(-c\,f-c\,h+c\,f\,h+f\,u+2\,h\,u-2\,f\,h\,u+2\,(f+h-f\,h)\,t_1+(f\,(-1+h)-h)\,t_2))\Big)\Big/\big((f\,(-1+h)-h)\,s\,(-f+(-1+f)\,h_2)\big)+O\big[\zeta^2\big]$$

Numerically evaluating the approximated eigenvalues for local stability such that $|\lambda_{(1)}| < 1$ and $|\lambda_{(2)}| < 1$ (Tables B9-B11):

Table B9: Numerically evaluating the approximated eigenvalues to determine local stability as the mutation rate of the wild-type allele varies across a given range

| u | $\sim\lambda_{(1)}$ | $\sim\lambda_{(2)}$ | Local Stability |
|---|---|---|---|
| $1.\times10^{-9}$ | 0.98501 | 0.992411 | Stable |
| $1.1\times10^{-8}$ | 0.98501 | 0.992411 | Stable |
| $2.1\times10^{-8}$ | 0.98501 | 0.992411 | Stable |
| $3.1\times10^{-8}$ | 0.98501 | 0.992411 | Stable |
| $4.1\times10^{-8}$ | 0.985011 | 0.992411 | Stable |
| $5.1\times10^{-8}$ | 0.985011 | 0.992411 | Stable |
| $6.1\times10^{-8}$ | 0.985011 | 0.992411 | Stable |
| $7.1\times10^{-8}$ | 0.985011 | 0.992411 | Stable |
| $8.1\times10^{-8}$ | 0.985011 | 0.992411 | Stable |
| $9.1\times10^{-8}$ | 0.985011 | 0.992411 | Stable |

Parameter values: $f = 0.5$, $s = 0.02$, $s_2 = 0.01$,
$s_3 = 0.001$, $h = 0.5$, $h_2 = 0.5$, $t_1 = 10$^−5, $t_2 = 10$^−4,
$c = 10$^−9, $z = 10$^−9

Table B10: Numerically evaluating the approximated eigenvalues to determine local stability as the forward spontaneous epimutation rate varies across a given range

| $t_1$ | $\sim\lambda_{(1)}$ | $\sim\lambda_{(2)}$ | Local Stability |
|---|---|---|---|
| 0.00001 | 0.98501 | 0.992411 | Stable |
| 0.00011 | 0.985115 | 0.992509 | Stable |
| 0.00021 | 0.98522 | 0.992608 | Stable |
| 0.00031 | 0.985324 | 0.992707 | Stable |
| 0.00041 | 0.985429 | 0.992806 | Stable |
| 0.00051 | 0.985534 | 0.992904 | Stable |
| 0.00061 | 0.985638 | 0.993003 | Stable |
| 0.00071 | 0.985743 | 0.993102 | Stable |
| 0.00081 | 0.985848 | 0.993201 | Stable |
| 0.00091 | 0.985952 | 0.993299 | Stable |
| 0.00101 | 0.986057 | 0.993398 | Stable |

Parameter values: f = 0.5, s = 0.02, $s_2$ = 0.01, $s_3$ = 0.001, h = 0.5, $h_2$ = 0.5, u = 10 ^ –9, $t_2$ = 10 ^ –4, c = 10 ^ –9, z = 10 ^ –9

Table B11: Numerically evaluating the approximated eigenvalues to determine local stability as the inbreeding coefficient varies across a given range

| f | $\sim\lambda_{(1)}$ | $\sim\lambda_{(2)}$ | Local Stability |
|---|---|---|---|
| 0.01 | 0.989912 | 0.99486 | Stable |
| 0.11 | 0.988911 | 0.99436 | Stable |
| 0.21 | 0.987911 | 0.99386 | Stable |
| 0.31 | 0.986911 | 0.993361 | Stable |
| 0.41 | 0.985911 | 0.992861 | Stable |
| 0.51 | 0.98491 | 0.992361 | Stable |
| 0.61 | 0.98391 | 0.991861 | Stable |
| 0.71 | 0.98291 | 0.991361 | Stable |
| 0.81 | 0.98191 | 0.990861 | Stable |
| 0.91 | 0.98091 | 0.990361 | Stable |

Parameter values: s = 0.02, $s_2$ = 0.01, $s_3$ = 0.001, h = 0.5, $h_2$ = 0.5, u = 10 ^ –9, $t_1$ = 10 ^ –5, $t_2$ = 10 ^ –4, c = 10 ^ –9, z = 10 ^ –9

Evaluation of local stability through numerical simulations of the recursion equations (Figure B1). Evidence for local stability was supported through simulations of the recursion equations beginning above (gray line) and below (black line) the equilibrium (dashed line).

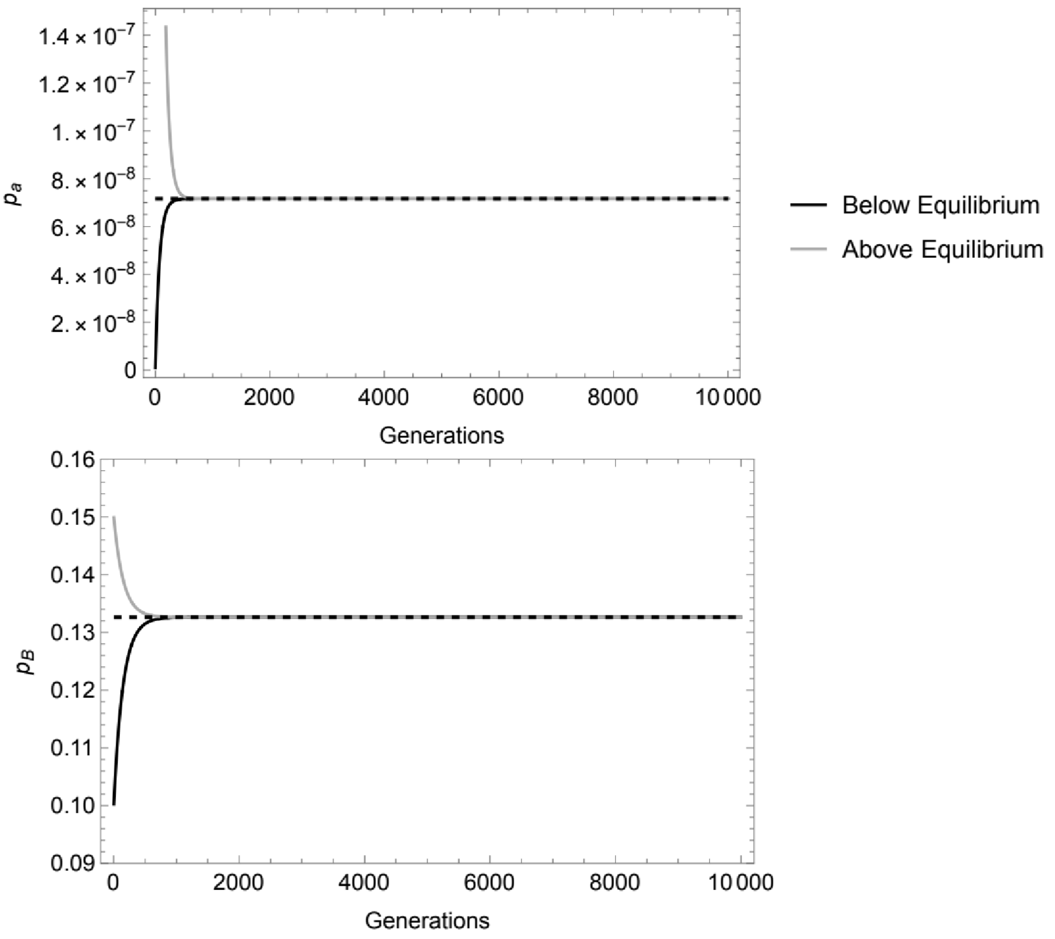


Figure B1: Plots of the deleterious allele frequency (top) and the epiallele frequency (bottom) across time in generations. The numerical parameter values are
$f = 0.5$, $s = 0.02$, $s_2 = 0.01$, $s_3 = 0.001$, $h = 0.5$, $h_2 = 0.5$, $u = 10\wedge{-9}$, $t_2 = 10\wedge{-4}$, $c = 10\wedge{-9}$, $z = 10\wedge{-9}$, $t_1 = 0.00101$.

The approximated eigenvalues corresponding with eq. 4 of the main text are

$$\begin{gathered}
\lambda_{(1)} \sim 1 - f s - h s + f h s + \big(-((-1+f)(f(-1+h)-h) s_3 t_1) + \\
s_2 \big(f \big((f(-1+h)-h)(1+f(-1+h) s - h s) t_1 - \big(\big(2 - 5h + 3h^2 s + f^2 \big(1 - 4h + 3h^2\big) s + f\big(-3 + 5h + 4 h s - 6 h^2 s\big)\big) u - \\
(f(-1+h)-h)(1+f(-1+h) s - h s) z\big)(-1+t_2)\big) + (-1+f) h_2 \big(-((f(-1+h)-h)(1+2f(-1+h) s - 2 h s) t_1) + \\
\big(\big(2 - 5h + 3h^2 s + f^2 \big(1 - 4h + 3h^2\big) s + f\big(-3 + 5h + 4 h s - 6 h^2 s\big)\big) u - (f(-1+h)-h)(1+f(-1+h) s - h s) z\big)(-1+t_2)\big)\big) + \\
\big(\big(2 - 5h + 3h^2 s + f^2 \big(1 - 4h + 3h^2\big) s + f\big(-3 + 5h + 4 h s - 6 h^2 s\big)\big) u - (f(-1+h)-h)(1+f(-1+h) s - h s) z\big) t_2\big) / \\
((f(-1+h)-h)((-f+(-1+f) h_2) s_2 (-1+t_2) + t_2)) \\
+ O\big[\zeta^2\big]
\end{gathered}$$

$$\begin{gathered}
\lambda_{(2)} \sim -((1 - f s_2 - h_2 s_2 + f h_2 s_2)(-1+t_2)) + \big(s(-f+(-1+f) h_2) s_2^2 (f((f+h-fh) t_1 - (c f(-1+h) - c h + (f + 2h - 2fh) u)(-1+t_2)) + \\
(-1+f) h_2 (3(f(-1+h)-h) t_1 + (c f(-1+h) - c h + (f+2h-2fh) u)(-1+t_2)))(-1+t_2) + \\
((f(-1+h)-h) s(-t_1 + (c-u)(-1+t_2)) - (-1+f) u s_3 (-1+t_2)) t_2 + \\
s_2 \big(c f^2 s + c f h s - c f^2 h s - f^2 s u - f h s u + f^2 h s u - f u s_3 + f^2 u s_3 + 2 f s t_1 - 3 f^2 s t_1 + 2 h s t_1 - 5 f h s t_1 + 3 f^2 h s t_1 - 3 c f^2 s t_2 - \\
3 c f h s t_2 + 3 c f^2 h s t_2 + 3 f^2 s u t_2 + 4 f h s u t_2 - 4 f^2 h s u t_2 + 2 f u s_3 t_2 - 2 f^2 u s_3 t_2 - 2 f s t_1 t_2 + 2 f^2 s t_1 t_2 - 2 h s t_1 t_2 +
\end{gathered}$$

$$4 f h s t_1 t_2 - 2 f^2 h s t_1 t_2 + 2 c f^2 s t_2^2 + 2 c f h s t_2^2 - 2 c f^2 h s t_2^2 - 2 f^2 s u t_2^2 - 3 f h s u t_2^2 + 3 f^2 h s u t_2^2 - f u s_3 t_2^2 + f^2 u s_3 t_2^2 + (-1+f) h_2 \left(-\left((-1+f) u s_3 (-1+t_2)^2\right) + (f(-1+h)-h) s t_1 (-5+4 t_2) + s(-1+t_2)(-((f(-1+h)-h)(c-u)) + (2 c f(-1+h) - 2 c h + f(2-3 h) u + 3 h u) t_2))\right)\Big/((f(-1+h)-h) s((-f+(-1+f) h_2) s_2 (-1+t_2)+t_2))$$

$$+O[\zeta^2]$$

Numerically evaluating the approximated eigenvalues for local stability such that $|\lambda_{(1)}| < 1$ and $|\lambda_{(2)}| < 1$ (Tables B12-B14):

Table B12: Numerically evaluating the approximated eigenvalues to determine local stability as the mutation rate of the wild-type allele varies across a given range

| u | $\sim\lambda_{(1)}$ | $\sim\lambda_{(2)}$ | Local Stability |
|---|---|---|---|
| $1.\times 10^{-9}$ | 0.985 | 0.793991 | Stable |
| $1.1\times 10^{-8}$ | 0.985 | 0.793991 | Stable |
| $2.1\times 10^{-8}$ | 0.985 | 0.793991 | Stable |
| $3.1\times 10^{-8}$ | 0.985 | 0.793991 | Stable |
| $4.1\times 10^{-8}$ | 0.985 | 0.793991 | Stable |
| $5.1\times 10^{-8}$ | 0.985 | 0.793991 | Stable |
| $6.1\times 10^{-8}$ | 0.985 | 0.793991 | Stable |
| $7.1\times 10^{-8}$ | 0.985 | 0.793991 | Stable |
| $8.1\times 10^{-8}$ | 0.985 | 0.793991 | Stable |
| $9.1\times 10^{-8}$ | 0.985 | 0.793991 | Stable |

Parameter values: $f = 0.5$, $s = 0.02$, $s_2 = 0.01$,
$s_3 = 0.001$, $h = 0.5$, $h_2 = 0.5$, $t_1 = 10\verb|^|-5$, $t_2 = 0.2$,
$c = 10\verb|^|-9$, $z = 10\verb|^|-9$

Table B13: Numerically evaluating the approximated eigenvalues to determine local stability as the forward spontaneous epimutation rate varies across a given range

| $t_1$ | $\sim\lambda_{(1)}$ | $\sim\lambda_{(2)}$ | Local Stability |
|---|---|---|---|
| 0.00001 | 0.985 | 0.793991 | Stable |
| 0.00011 | 0.985004 | 0.793897 | Stable |
| 0.00021 | 0.985008 | 0.793804 | Stable |
| 0.00031 | 0.985012 | 0.79371 | Stable |
| 0.00041 | 0.985016 | 0.793617 | Stable |
| 0.00051 | 0.985019 | 0.793523 | Stable |
| 0.00061 | 0.985023 | 0.79343 | Stable |
| 0.00071 | 0.985027 | 0.793336 | Stable |
| 0.00081 | 0.985031 | 0.793243 | Stable |
| 0.00091 | 0.985035 | 0.793149 | Stable |
| 0.00101 | 0.985038 | 0.793056 | Stable |

Parameter values: $f = 0.5$, $s = 0.02$, $s_2 = 0.01$,
$s_3 = 0.001$, $h = 0.5$, $h_2 = 0.5$, $u = 10\verb|^|-9$, $t_2 = 0.2$,
$c = 10\verb|^|-9$, $z = 10\verb|^|-9$

Table B14: Numerically evaluating the approximated eigenvalues to determine local stability as the inbreeding coefficient varies across a given range

| f | ~ $\lambda_{(1)}$ | ~ $\lambda_{(2)}$ | Local Stability |
|---|---|---|---|
| 0.01 | 0.9899 | 0.79595 | Stable |
| 0.11 | 0.9889 | 0.79555 | Stable |
| 0.21 | 0.9879 | 0.795151 | Stable |
| 0.31 | 0.9869 | 0.794751 | Stable |
| 0.41 | 0.9859 | 0.794351 | Stable |
| 0.51 | 0.9849 | 0.793951 | Stable |
| 0.61 | 0.9839 | 0.793551 | Stable |
| 0.71 | 0.9829 | 0.793151 | Stable |
| 0.81 | 0.9819 | 0.792751 | Stable |
| 0.91 | 0.9809 | 0.792351 | Stable |

Parameter values: $s = 0.02$, $s_2 = 0.01$, $s_3 = 0.001$,
$h = 0.5$, $h_2 = 0.5$, $t_1 = 10$ ^ –5, $t_2 = 0.2$, $u = 10$ ^ –9,
$c = 10$ ^ –9, $z = 10$ ^ –9

Evaluation of local stability through numerical simulations of the recursion equations (Figure B2). Evidence for local stability was supported through simulations of the recursion equations beginning above (gray line) and below (black line) the equilibrium (dashed line).

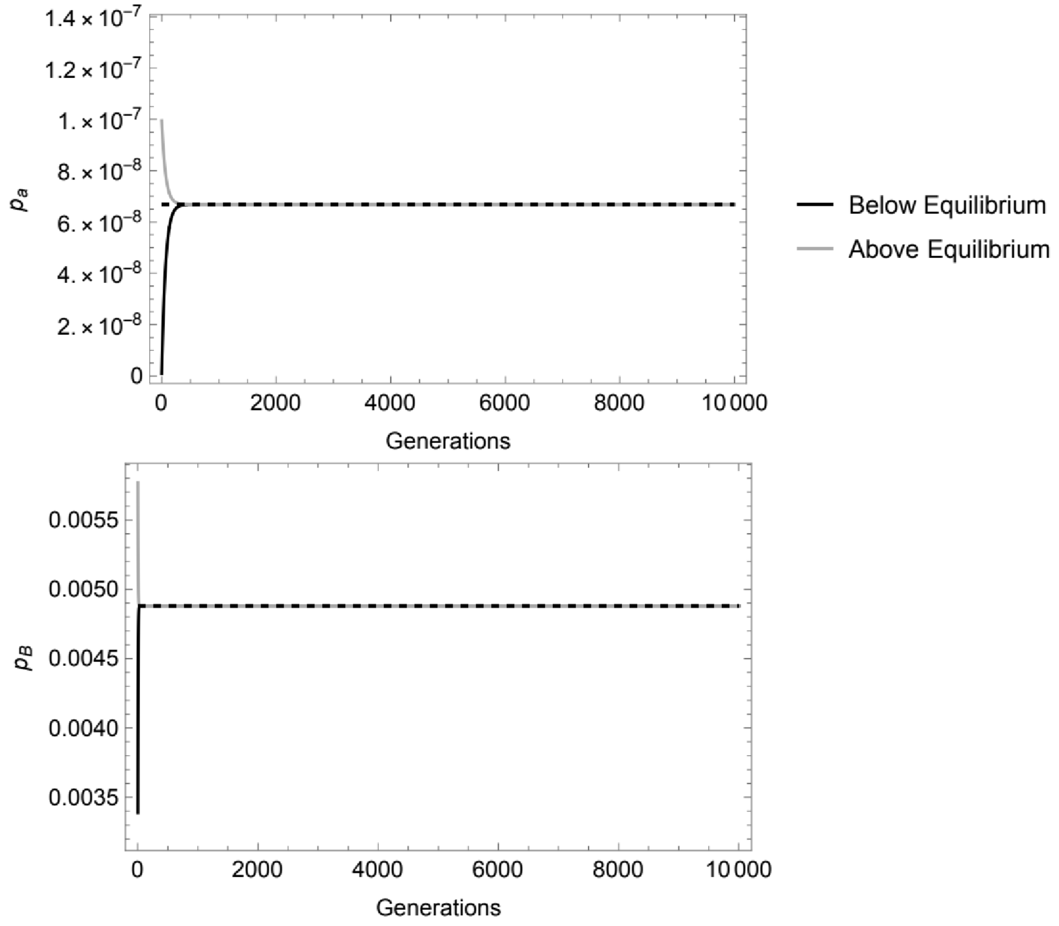

Figure B2: Plots of the deleterious allele frequency (top) and the epiallele frequency (bottom) across time in generations. The numerical parameter values are f = 0.5, s = 0.02, $s_2$ = 0.01, $s_3$ = 0.001, h = 0.5, $h_2$ = 0.5, u = 10 ^ −9, $t_2$ = 0.2, c = 10 ^ −9, z = 10 ^ −9, $t_1$ = 0.00101

# Case C: Random Mating (*f* = 0) and with Paramutation (m > 0)

Evaluating the accuracy of the analytical approximation (eq. 5 of the main text) in comparison to numerical equilibria solutions (Tables C1-C4):

Table C1: Evaluating the accuracy of the equilibrium approximation for the deleterious allele as the paramutation rate increases from a lower to upper limit such that the equilibrium is biologically valid

| m | ~ $\hat{p}_a$ Analytical | $\hat{p}_a$ Numerical | Percentage Difference |
|---|---|---|---|
| 0.001 | $1.\times 10^{-7}$ | $1.\times 10^{-7}$ | 0 |
| 0.0015 | $1.\times 10^{-7}$ | $1.\times 10^{-7}$ | 0 |
| 0.002 | $1.\times 10^{-7}$ | $1.\times 10^{-7}$ | 0 |
| 0.0025 | $1.\times 10^{-7}$ | $1.\times 10^{-7}$ | 0 |
| 0.003 | $1.\times 10^{-7}$ | $1.\times 10^{-7}$ | 0 |
| 0.0035 | $1.\times 10^{-7}$ | $1.\times 10^{-7}$ | 0 |
| 0.004 | $1.\times 10^{-7}$ | $1.\times 10^{-7}$ | 0 |
| 0.0045 | $1.\times 10^{-7}$ | $1.\times 10^{-7}$ | 0 |
| 0.005 | $1.\times 10^{-7}$ | $1.\times 10^{-7}$ | 0 |

Parameter values: s = 0.02, $s_2$ = 0.01, $s_3$ = 0.001, h = 0.5, $h_2$ = 0.5,
$t_1$ = 10 ^ −5, $t_2$ = 10 ^ −4, u = 10 ^ −9, c = 10 ^ −9,
z = 10 ^ −9

Table C2: Evaluating the accuracy of the equilibrium approximation for the epiallele as the paramutation rate increases from a lower to upper limit such that the equilibrium is biologically valid

| m | ~ $\hat{p}_B$ Analytical | $\hat{p}_B$ Numerical | Percentage Difference |
|---|---|---|---|
| 0.001 | 0.0024907 | 0.0024302 | 2.43 |
| 0.0015 | 0.0028389 | 0.0027606 | 2.76 |
| 0.002 | 0.0033003 | 0.0031949 | 3.19 |
| 0.0025 | 0.0039409 | 0.0037913 | 3.8 |
| 0.003 | 0.00489 | 0.0046616 | 4.67 |
| 0.0035 | 0.0064412 | 0.0060503 | 6.07 |
| 0.004 | 0.009434 | 0.008617 | 8.66 |
| 0.0045 | 0.0176211 | 0.0149618 | 15.09 |
| 0.005 | 0.133333 | 0.0561252 | 57.91 |

Parameter values: s = 0.02, $s_2$ = 0.01, $s_3$ = 0.001, h = 0.5, $h_2$ = 0.5,
$t_1$ = 10 ^ −5, $t_2$ = 10 ^ −4, u = 10 ^ −9, c = 10 ^ −9,
z = 10 ^ −9

Table C3: Evaluating the accuracy of the equilibrium approximation for the deleterious allele as the forward spontaneous epimutation rate increases from a lower to upper limit

| $t_1$ | ~ $\hat{p}_a$ Analytical | $\hat{p}_a$ Numerical | Percentage Difference |
|---|---|---|---|
| 0.00001 | $1.\times10^{-7}$ | $1.\times10^{-7}$ | 0. |
| 0.00011 | $1.\times10^{-7}$ | $1.\times10^{-7}$ | 0. |
| 0.00021 | $1.\times10^{-7}$ | $1.\times10^{-7}$ | 0. |
| 0.00031 | $1.\times10^{-7}$ | $1.\times10^{-7}$ | 0. |
| 0.00041 | $1.\times10^{-7}$ | $1.\times10^{-7}$ | 0. |
| 0.00051 | $1.\times10^{-7}$ | $1.\times10^{-7}$ | 0. |
| 0.00061 | $1.\times10^{-7}$ | $1.\times10^{-7}$ | 0. |
| 0.00071 | $1.\times10^{-7}$ | $1.\times10^{-7}$ | 0. |
| 0.00081 | $1.\times10^{-7}$ | $1.\times10^{-7}$ | 0. |
| 0.00091 | $1.\times10^{-7}$ | $1.\times10^{-7}$ | 0. |
| 0.00101 | $1.\times10^{-7}$ | $1.\times10^{-7}$ | 0. |

Parameter values: $m = 0.001$, $s = 0.02$, $s_2 = 0.01$, $s_3 = 0.001$, $h = 0.5$,
$h_2 = 0.5$, $u = 10^{-9}$, $t_2 = 10^{-4}$, $u = 10^{-9}$,
$c = 10^{-9}$, $z = 10^{-9}$

Table C4: Evaluating the accuracy of the equilibrium approximation for the epiallele as the forward spontaneous epimutation rate increases from a lower to upper limit

| $t_1$ | ~ $\hat{p}_B$ Analytical | $\hat{p}_B$ Numerical | Percentage Difference |
|---|---|---|---|
| 0.00001 | 0.0024907 | 0.0024302 | 2.43 |
| 0.00011 | 0.0273973 | 0.0267139 | 2.49 |
| 0.00021 | 0.0523039 | 0.0509627 | 2.56 |
| 0.00031 | 0.0772105 | 0.0751742 | 2.64 |
| 0.00041 | 0.102117 | 0.0993462 | 2.71 |
| 0.00051 | 0.127024 | 0.123476 | 2.79 |
| 0.00061 | 0.15193 | 0.14756 | 2.88 |
| 0.00071 | 0.176837 | 0.171595 | 2.96 |
| 0.00081 | 0.201744 | 0.195578 | 3.06 |
| 0.00091 | 0.22665 | 0.219504 | 3.15 |
| 0.00101 | 0.251557 | 0.24337 | 3.25 |

Parameter values: $m = 0.001$, $s = 0.02$, $s_2 = 0.01$, $s_3 = 0.001$, $h = 0.5$,
$h_2 = 0.5$, $u = 10^{-9}$, $t_2 = 10^{-4}$, $u = 10^{-9}$,
$c = 10^{-9}$, $z = 10^{-9}$

Evaluating the accuracy of the analytical approximation (eq. 6 of the main text) in comparison to numerical equilibria solutions (Tables C5-C8):

Table C5: Evaluating the accuracy of the equilibrium approximation for the deleterious allele as the paramutation rate increases from a lower to upper limit such that the equilibrium is biologically valid

| m | ~ $\hat{p}_a$ Analytical | $\hat{p}_a$ Numerical | Percentage Difference |
|---|---|---|---|
| 0.01 | $2.\times 10^{-7}$ | $2.\times 10^{-7}$ | 0. |
| 0.06 | $2.\times 10^{-7}$ | $2.\times 10^{-7}$ | 0. |
| 0.11 | $2.\times 10^{-7}$ | $2.\times 10^{-7}$ | 0. |
| 0.16 | $2.\times 10^{-7}$ | $2.\times 10^{-7}$ | 0. |
| 0.21 | $2.\times 10^{-7}$ | $2.\times 10^{-7}$ | 0. |
| 0.26 | $2.\times 10^{-7}$ | $2.\times 10^{-7}$ | 0. |
| 0.31 | $2.\times 10^{-7}$ | $2.\times 10^{-7}$ | 0. |
| 0.36 | $2.\times 10^{-7}$ | $2.\times 10^{-7}$ | 0. |
| 0.41 | $2.\times 10^{-7}$ | $2.\times 10^{-7}$ | 0. |
| 0.46 | $2.\times 10^{-7}$ | $2.\times 10^{-7}$ | 0. |
| 0.51 | $2.\times 10^{-7}$ | $2.\times 10^{-7}$ | 0. |
| 0.56 | $2.\times 10^{-7}$ | $2.\times 10^{-7}$ | 0. |
| 0.61 | $2.\times 10^{-7}$ | $2.\times 10^{-7}$ | 0. |
| 0.66 | $2.\times 10^{-7}$ | $2.\times 10^{-7}$ | 0. |
| 0.71 | $2.\times 10^{-7}$ | $2.\times 10^{-7}$ | 0. |
| 0.76 | $2.\times 10^{-7}$ | $2.\times 10^{-7}$ | 0. |
| 0.81 | $2.\times 10^{-7}$ | $2.\times 10^{-7}$ | 0. |
| 0.86 | $2.\times 10^{-7}$ | $2.\times 10^{-7}$ | 0. |
| 0.91 | $2.\times 10^{-7}$ | $2.\times 10^{-7}$ | 0. |

Parameter values: $s = 0.02$, $s_2 = 0.01$, $s_3 = 0.001$, $h = 0.5$, $h_2 = 0.5$,
$t_1 = 10\text{^}-5$, $t_2 = 10\text{^}-4$, $u = 10\text{^}-9$, $c = 10\text{^}-9$, $z = 10\text{^}-9$

Table C6: Evaluating the accuracy of the equilibrium approximation for the epiallele as the paramutation rate increases from a lower to upper limit such that the equilibrium is biologically valid

| m | ~ $\hat{p}_B$ Analytical | $\hat{p}_B$ Numerical | Percentage Difference |
|---|---|---|---|
| 0.01 | 0.980026 | 0.98 | 0. |
| 0.06 | 0.99819 | 0.99819 | 0. |
| 0.11 | 0.999052 | 0.999052 | 0. |
| 0.16 | 0.999358 | 0.999358 | 0. |
| 0.21 | 0.999514 | 0.999514 | 0. |
| 0.26 | 0.99961 | 0.99961 | 0. |
| 0.31 | 0.999674 | 0.999674 | 0. |
| 0.36 | 0.999719 | 0.99972 | 0. |
| 0.41 | 0.999754 | 0.999754 | 0. |
| 0.46 | 0.999781 | 0.999781 | 0. |
| 0.51 | 0.999803 | 0.999803 | 0. |
| 0.56 | 0.999821 | 0.999821 | 0. |
| 0.61 | 0.999835 | 0.999835 | 0. |
| 0.66 | 0.999848 | 0.999848 | 0. |
| 0.71 | 0.999859 | 0.999859 | 0. |
| 0.76 | 0.999868 | 0.999868 | 0. |
| 0.81 | 0.999876 | 0.999876 | 0. |
| 0.86 | 0.999883 | 0.999883 | 0. |
| 0.91 | 0.99989 | 0.99989 | 0. |

Parameter values: $s = 0.02$, $s_2 = 0.01$, $s_3 = 0.001$, $h = 0.5$, $h_2 = 0.5$,
$t_1 = 10\text{^}-5$, $t_2 = 10\text{^}-4$, $u = 10\text{^}-9$, $c = 10\text{^}-9$, $z = 10\text{^}-9$

Table C7: Evaluating the accuracy of the equilibrium approximation for the deleterious allele as the forward spontaneous epimutation rate increases from a lower to upper limit

| $t_1$ | ~ $\hat{p}_a$ Analytical | $\hat{p}_a$ Numerical | Percentage Difference |
|---|---|---|---|
| 0.00001 | $2.\times 10^{-7}$ | $2.\times 10^{-7}$ | 0. |
| 0.00011 | $2.\times 10^{-7}$ | $2.\times 10^{-7}$ | 0. |
| 0.00021 | $2.\times 10^{-7}$ | $2.\times 10^{-7}$ | 0. |
| 0.00031 | $2.\times 10^{-7}$ | $2.\times 10^{-7}$ | 0. |
| 0.00041 | $2.\times 10^{-7}$ | $2.\times 10^{-7}$ | 0. |
| 0.00051 | $2.\times 10^{-7}$ | $2.\times 10^{-7}$ | 0. |
| 0.00061 | $2.\times 10^{-7}$ | $2.\times 10^{-7}$ | 0. |
| 0.00071 | $2.\times 10^{-7}$ | $2.\times 10^{-7}$ | 0. |
| 0.00081 | $2.\times 10^{-7}$ | $2.\times 10^{-7}$ | 0. |
| 0.00091 | $2.\times 10^{-7}$ | $2.\times 10^{-7}$ | 0. |
| 0.00101 | $2.\times 10^{-7}$ | $2.\times 10^{-7}$ | 0. |

Parameter values: m = 0.1, s = 0.02, $s_2$ = 0.01, $s_3$ = 0.001, h = 0.5,
$h_2$ = 0.5, u = 10 ^ −9, $t_2$ = 10 ^ −4, u = 10 ^ −9,
c = 10 ^ −9, z = 10 ^ −9

Table C8: Evaluating the accuracy of the equilibrium approximation for the epiallele as the forward spontaneous epimutation rate increases from a lower to upper limit

| $t_1$ | ~ $\hat{p}_B$ Analytical | $\hat{p}_B$ Numerical | Percentage Difference |
|---|---|---|---|
| 0.00001 | 0.998952 | 0.998952 | 0. |
| 0.00011 | 0.998953 | 0.998952 | 0. |
| 0.00021 | 0.998954 | 0.998952 | 0. |
| 0.00031 | 0.998955 | 0.998952 | 0. |
| 0.00041 | 0.998956 | 0.998952 | 0. |
| 0.00051 | 0.998957 | 0.998952 | 0. |
| 0.00061 | 0.998958 | 0.998952 | 0. |
| 0.00071 | 0.998959 | 0.998952 | 0. |
| 0.00081 | 0.99896 | 0.998952 | 0. |
| 0.00091 | 0.998961 | 0.998952 | 0. |
| 0.00101 | 0.998962 | 0.998952 | 0. |

Parameter values: m = 0.1, s = 0.02, $s_2$ = 0.01, $s_3$ = 0.001, h = 0.5,
$h_2$ = 0.5, u = 10 ^ −9, $t_2$ = 10 ^ −4, u = 10 ^ −9,
c = 10 ^ −9, z = 10 ^ −9

Evaluating the accuracy of the analytical approximation (eq. 7 of the main text) in comparison to numerical equilibria solutions

(Tables C9-C12):

Table C9: Evaluating the accuracy of the equilibrium approximation for the deleterious allele as the paramutation rate increases from a lower to upper limit such that the equilibrium is biologically valid

| m | ~ $\hat{p}_a$ Analytical | $\hat{p}_a$ Numerical | Percentage Difference |
|---|---|---|---|
| 0.005 | $1.\times 10^{-7}$ | $1.\times 10^{-7}$ | 0. |
| 0.025 | $1.\times 10^{-7}$ | $1.\times 10^{-7}$ | 0. |
| 0.045 | $1.\times 10^{-7}$ | $1.\times 10^{-7}$ | 0. |
| 0.065 | $1.\times 10^{-7}$ | $1.\times 10^{-7}$ | 0. |
| 0.085 | $1.\times 10^{-7}$ | $1.\times 10^{-7}$ | 0. |
| 0.105 | $1.\times 10^{-7}$ | $1.\times 10^{-7}$ | 0. |
| 0.125 | $1.\times 10^{-7}$ | $1.\times 10^{-7}$ | 0. |
| 0.145 | $1.\times 10^{-7}$ | $1.\times 10^{-7}$ | 0. |
| 0.165 | $1.\times 10^{-7}$ | $1.\times 10^{-7}$ | 0. |
| 0.185 | $1.\times 10^{-7}$ | $1.\times 10^{-7}$ | 0. |
| 0.205 | $1.\times 10^{-7}$ | $1.\times 10^{-7}$ | 0. |

Parameter values: $s = 0.02$, $s_2 = 0.01$, $s_3 = 0.001$, $h = 0.5$,
$h_2 = 0.5$, $t_1 = 10$ ^ −5, $t_2 = 0.2$, $u = 10$ ^ −9, $c = 10$ ^ −9,
$z = 10$ ^ −9

Table C10: Evaluating the accuracy of the equilibrium approximation for the epiallele as the paramutation rate increases from a lower to upper limit such that the equilibrium is biologically valid

| m | ~ $\hat{p}_B$ Analytical | $\hat{p}_B$ Numerical | Percentage Difference |
|---|---|---|---|
| 0.005 | 0.00005 | 0.00005 | 0. |
| 0.025 | 0.0000543 | 0.0000543 | 0. |
| 0.045 | 0.0000593 | 0.0000593 | 0. |
| 0.065 | 0.0000655 | 0.0000654 | 0.15 |
| 0.085 | 0.000073 | 0.000073 | 0. |
| 0.105 | 0.0000825 | 0.0000825 | 0. |
| 0.125 | 0.0000948 | 0.0000948 | 0. |
| 0.145 | 0.0001114 | 0.0001114 | 0. |
| 0.165 | 0.0001352 | 0.0001351 | 0.07 |
| 0.185 | 0.0001718 | 0.0001717 | 0.06 |
| 0.205 | 0.0002355 | 0.0002352 | 0.13 |

Parameter values: $s = 0.02$, $s_2 = 0.01$, $s_3 = 0.001$, $h = 0.5$,
$h_2 = 0.5$, $t_1 = 10$ ^ −5, $t_2 = 0.2$, $u = 10$ ^ −9, $c = 10$ ^ −9, $z = 10$ ^ −9

Table C11: Evaluating the accuracy of the equilibrium approximation for the deleterious allele as the forward spontaneous epimutation rate increases from a lower to upper limit

| $t_1$ | ~ $\hat{p}_a$ Analytical | $\hat{p}_a$ Numerical | Percentage Difference |
|---|---|---|---|
| 0.00001 | $1.\times 10^{-7}$ | $1.\times 10^{-7}$ | 0. |
| 0.00011 | $1.\times 10^{-7}$ | $1.\times 10^{-7}$ | 0. |
| 0.00021 | $1.\times 10^{-7}$ | $1.\times 10^{-7}$ | 0. |
| 0.00031 | $1.\times 10^{-7}$ | $1.\times 10^{-7}$ | 0. |
| 0.00041 | $1.\times 10^{-7}$ | $1.\times 10^{-7}$ | 0. |
| 0.00051 | $1.\times 10^{-7}$ | $1.\times 10^{-7}$ | 0. |
| 0.00061 | $1.\times 10^{-7}$ | $1.\times 10^{-7}$ | 0. |
| 0.00071 | $1.\times 10^{-7}$ | $1.\times 10^{-7}$ | 0. |
| 0.00081 | $1.\times 10^{-7}$ | $1.\times 10^{-7}$ | 0. |
| 0.00091 | $1.\times 10^{-7}$ | $1.\times 10^{-7}$ | 0. |
| 0.00101 | $1.\times 10^{-7}$ | $1.\times 10^{-7}$ | 0. |

Parameter values: m = 0.1, s = 0.02, $s_2$ = 0.01, $s_3$ = 0.001, h = 0.5,
$h_2$ = 0.5, u = 10 ^ −9, $t_2$ = 0.2, u = 10 ^ −9,
c = 10 ^ −9, z = 10 ^ −9

Table C12: Evaluating the accuracy of the equilibrium approximation for the epiallele as the forward spontaneous epimutation rate increases from a lower to upper limit

| $t_1$ | ~ $\hat{p}_B$ Analytical | $\hat{p}_B$ Numerical | Percentage Difference |
|---|---|---|---|
| 0.00001 | 0.0000799 | 0.0000799 | 0. |
| 0.00011 | 0.0008786 | 0.0008773 | 0.15 |
| 0.00021 | 0.0016773 | 0.0016726 | 0.28 |
| 0.00031 | 0.002476 | 0.0024658 | 0.41 |
| 0.00041 | 0.0032748 | 0.0032569 | 0.55 |
| 0.00051 | 0.0040735 | 0.0040459 | 0.68 |
| 0.00061 | 0.0048722 | 0.0048328 | 0.81 |
| 0.00071 | 0.0056709 | 0.0056177 | 0.94 |
| 0.00081 | 0.0064696 | 0.0064004 | 1.07 |
| 0.00091 | 0.0072684 | 0.0071812 | 1.2 |
| 0.00101 | 0.0080671 | 0.0079599 | 1.33 |

Parameter values: m = 0.1, s = 0.02, $s_2$ = 0.01, $s_3$ = 0.001, h = 0.5,
$h_2$ = 0.5, u = 10 ^ −9, $t_2$ = 0.2, u = 10 ^ −9,
c = 10 ^ −9, z = 10 ^ −9

Following the main text (see case C results and table 6 equilibrium 2), an equilibrium arises where the epiallele and wild-type allele are at moderate to high frequencies and the deleterious allele is at a low frequency. Paramutation increases the epiallele at equilibrium (see figure C1) and decreases the wild-type allele (not shown) and has marginal effects on the deleterious allele (not shown).

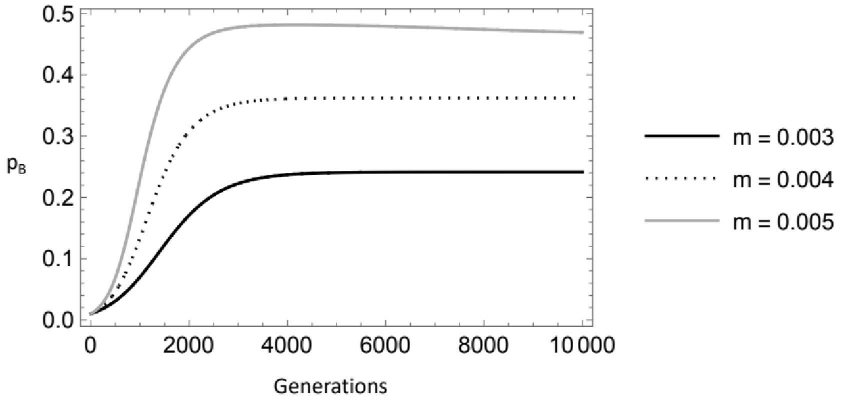


Figure C1: A plot of numerical simulations showing the epiallele frequency across time. The epiallele increases with the paramutation rate going from the black line to the dotted line to the gray line. Other parameter values were held constant:
s = 0.02, $s_2$ = 0.01, $s_3$ = 0.0005, h = 0.1, $h_2$ = 0.1, $t_1$ = 10 ^ −5, $t_2$ = 5 x 10 ^ −5, u = 10 ^ −9, c = 10 ^ −9, z = 10 ^ −9

## Local Stability Analysis (following Appendix Section A2 )

The approximated eigenvalues corresponding with eq. 5 of the main text are

$$\lambda_{(1)} \sim 1 - h\,s + \left(m\left(-2 + 5\,h - 3\,h^2\,s\right)u + h\,m\,(-1 + h\,s)\,z - h\,s_3\,t_1 + s_2\left(2\,m\left(\left(2 - 5\,h + 3\,h^2\,s\right)u + h\,(1 - h\,s)\,z + h\,(-1 + h\,s)\,t_1\right) + h_2\left(-\left((-1 + m)\left(\left(2 - 5\,h + 3\,h^2\,s\right)u + h\,(1 - h\,s)\,z\right)\right) - h\,(1 - 2\,h\,s + 2\,m\,(-1 + h\,s))\,t_1\right)\right)\right) / (h\,(m + (-2\,m + (-1 + m)\,h_2)\,s_2)) + O[\zeta^2]$$

$$\lambda_{(2)} \sim 1 + m - 2\,m\,s_2 - h_2\,s_2 + m\,h_2\,s_2 + \left(-m\,(c\,h\,s + c\,h\,m\,s + m\,u - h\,s\,u - 2\,h\,m\,s\,u - u\,s_3 + h\,(-1 + m)\,s\,t_1 + h\,s\,t_2 + h\,m\,s\,t_2) - (-2\,m + (-1 + m)\,h_2)\,s_2^2\,(-2\,m\,(c\,h\,s + u - 2\,h\,s\,u - h\,s\,t_1 + h\,s\,t_2) + h_2\,(-c\,h\,s + c\,h\,m\,s + m\,u + 2\,h\,s\,u - 2\,h\,m\,s\,u - 3\,h\,(-1 + m)\,s\,t_1 + h\,(-1 + m)\,s\,t_2)) + s_2\left(h_2\left(c\,h\,s + c\,h\,m\,s - 2\,c\,h\,m^2\,s + m\,u - 2\,m^2\,u - h\,s\,u - 3\,h\,m\,s\,u + 4\,h\,m^2\,s\,u + (-1 + m)\,u\,s_3 + h\left(-5 + 3\,m + 2\,m^2\right)s\,t_1 + h\,s\,t_2 + h\,m\,s\,t_2 - 2\,h\,m^2\,s\,t_2\right) + 2\,(-m\,u\,s_3 + h\,(1 - 3\,m)\,s\,t_1 + m\,(c\,h\,(1 + 2\,m)\,s - (-2\,m + h\,s + 4\,h\,m\,s)\,u + h\,(1 + 2\,m)\,s\,t_2))\right)\right) / (h\,s\,(m + (-2\,m + (-1 + m)\,h_2)\,s_2)) + O[\zeta^2]$$

Numerically evaluating the approximated eigenvalues for local stability such that $|\lambda_{(1)}| < 1$ and $|\lambda_{(2)}| < 1$ (Tables C13 and C14):

Table C13: Numerically evaluating the approximated eigenvalues to determine local stability as the paramutation rate varies across a given range

| m | $\sim\lambda_{(1)}$ | $\sim\lambda_{(2)}$ | Local Stability |
|---|---|---|---|
| 0.001 | 0.990015 | 0.995895 | Stable |
| 0.0015 | 0.990017 | 0.996388 | Stable |
| 0.002 | 0.99002 | 0.99688 | Stable |
| 0.0025 | 0.990023 | 0.997373 | Stable |
| 0.003 | 0.990029 | 0.997865 | Stable |
| 0.0035 | 0.990038 | 0.998358 | Stable |
| 0.004 | 0.990056 | 0.998851 | Stable |
| 0.0045 | 0.990105 | 0.999344 | Stable |
| 0.005 | 0.990793 | 0.999848 | Stable |

Parameter values: $s = 0.02$, $s_2 = 0.01$, $s_3 = 0.001$, $h = 0.5$, $h_2 = 0.5$, $t_1 = 10$ ^ −5,
$t_2 = 10$ ^ −4, $u = 10$ ^ −9, $c = 10$ ^ −9, $z = 10$ ^ −9

Table C14: Numerically evaluating the approximated eigenvalues to determine local stability as the forward spontaneous epimutation rate varies across a given range

| $t_1$ | $\sim\lambda_{(1)}$ | $\sim\lambda_{(2)}$ | Local Stability |
|---|---|---|---|
| 0.00001 | 0.990015 | 0.995895 | Stable |
| 0.00011 | 0.990162 | 0.995994 | Stable |
| 0.00021 | 0.990309 | 0.996093 | Stable |
| 0.00031 | 0.990456 | 0.996192 | Stable |
| 0.00041 | 0.990604 | 0.996291 | Stable |
| 0.00051 | 0.990751 | 0.99639 | Stable |
| 0.00061 | 0.990898 | 0.996489 | Stable |
| 0.00071 | 0.991045 | 0.996588 | Stable |
| 0.00081 | 0.991192 | 0.996686 | Stable |
| 0.00091 | 0.991339 | 0.996785 | Stable |
| 0.00101 | 0.991487 | 0.996884 | Stable |

Parameter values: $m = 0.001$, $s = 0.02$, $s_2 = 0.01$, $s_3 = 0.001$, $h = 0.5$, $h_2 = 0.5$, $u = 10$ ^ −9,
$t_2 = 10$ ^ −4, $u = 10$ ^ −9, $c = 10$ ^ −9, $z = 10$ ^ −9

Evaluation of local stability through numerical simulations of the recursion equations (Figure C2). Evidence for local stability was

supported through simulations of the recursion equations beginning above (gray line) and below (black line) the equilibrium (dashed line).

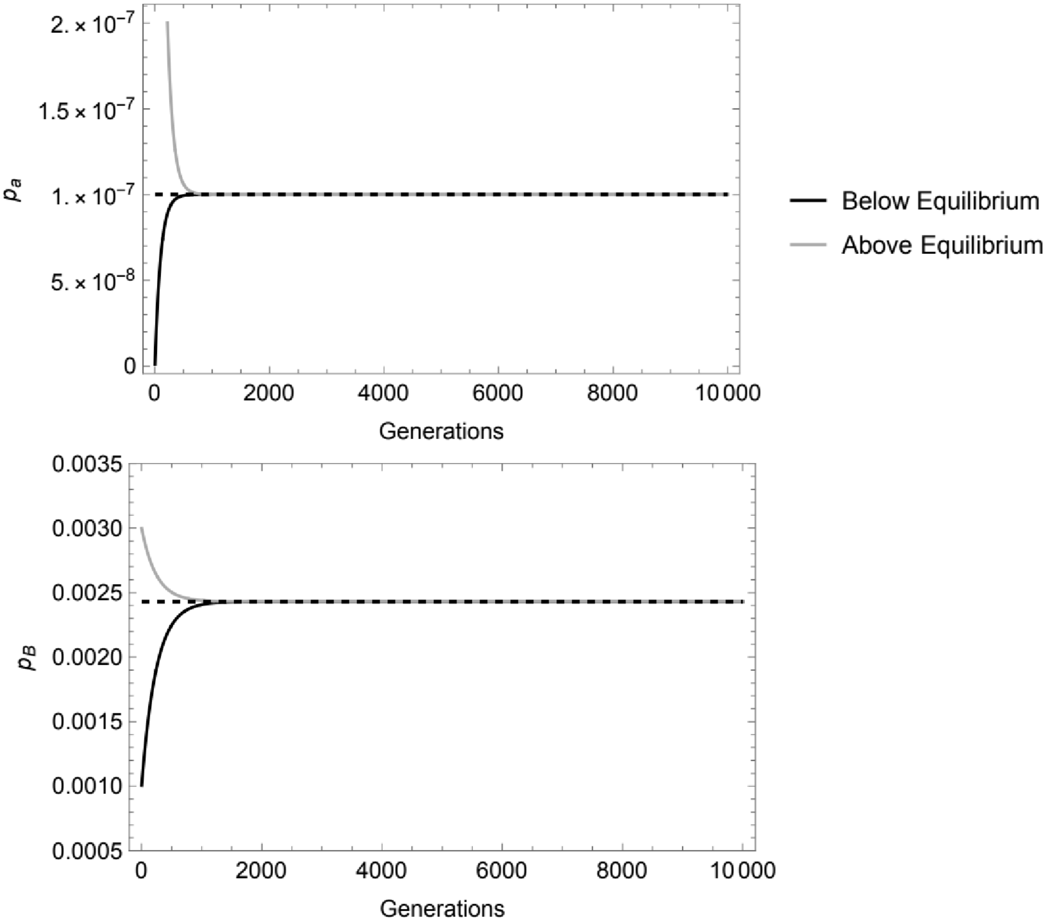


Figure C2: Plots of the deleterious allele frequency (top) and the epiallele frequency (bottom) across time in generations. The numerical parameter values are
$s = 0.02$, $s_2 = 0.01$, $s_3 = 0.001$, $h = 0.5$, $h_2 = 0.5$, $t_1 = 10\wedge{-5}$, $t_2 = 10\wedge{-4}$, $u = 10\wedge{-9}$, $c = 10\wedge{-9}$, $z = 10\wedge{-9}$, $m = 0.001$

The approximated eigenvalues corresponding with eq. 6 of the main text are

$$
\begin{gathered}
\lambda_{(1)} \sim -\frac{(-1+m)(-1+h_2 s_2)}{-1+s_2} + \\
(-((-1+m) h_2 s_2^3 (-c+u+t_1-t_2+4 m t_2+(-1+m) h_2^2 (2 c-u-t_1+3 t_2)+h_2 (3 c-c m-2 u+m u+(-2+m) t_1+(4-7 m) t_2)))+ \\
m (c m+c h s-2 c h m s-h s u+h m s u+h (-1+m) s t_1+h s t_2+h m s t_2+s_3 ((-1+m) (2 c-u)-(-1+m) t_1-(1+m) t_2))+ \\
s_2 (-3 c m+c m^2-c h s+3 c h m s+m u-m^2 u+h s u-h m s u+m t_1-m^2 t_1+h s t_1-h m s t_1-m t_2- \\
m^2 t_2-3 h s t_2+5 h m s t_2-4 h m^2 s t_2+s_3 (-((-1+m) (2 c-u))+(-1+m) t_1+(3-5 m+4 m^2) t_2)+ \\
h_2 (4 c m-4 c m^2+c h s-5 c h m s+4 c h m^2 s-m u+m^2 u-h s u+3 h m s u-2 h m^2 s u-(-1+m) (-h s+m (-1+2 h s)) \\
t_1+m t_2+m^2 t_2+5 h s t_2-7 h m s t_2+2 h m^2 s t_2-(-1+m) s_3 ((-1+2 m) (2 c-u)+(1-2 m) t_1+(-5+2 m) t_2)))-
\end{gathered}
$$

$$s_2^2\,(-2\,c+c\,m+c\,h\,s+u-m\,u+t_1-m\,t_1-3\,t_2+5\,m\,t_2-4\,m^2\,t_2+(-1+m)\,h_2^2\,(3\,c-5\,c\,m-2\,c\,h\,s+2\,c\,h\,m\,s-u+2\,m\,u+h\,s\,u-h\,m\,s\,u+(-1+2\,m+h\,s-h\,m\,s)\,t_1+5\,t_2-2\,m\,t_2-3\,h\,s\,t_2+3\,h\,m\,s\,t_2-(-1+m)\,s_3\,(2\,c-u-t_1+3\,t_2))+h_2\,(5\,c-7\,c\,m+2\,c\,m^2-3\,c\,h\,s+3\,c\,h\,m\,s-2\,u+4\,m\,u-2\,m^2\,u+h\,s\,u-h\,m\,s\,u-(-1+m)\,(-2+2\,m+h\,s)\,t_1+8\,t_2-12\,m\,t_2+6\,m^2\,t_2-h\,s\,t_2+5\,h\,m\,s\,t_2-4\,h\,m^2\,s\,t_2+(-1+m)\,s_3\,(-2\,c+u+t_1+(-1+4\,m)\,t_2))))\Big/$$
$$((-1+s_2)\,(-m+(1+(-1+m)\,h_2)\,s_2)\,(h\,s+(-1+h_2)\,s_2-s_3))$$
$$+O[\zeta^2]$$

$$\lambda_{(2)} \sim \frac{-1+h\,s+h_2\,s_2-s_3}{-1+s_2}+\big(m\,s\,\big(c\,\big(-2+5\,h-3\,h^2\,s\big)+h\,(-1+h\,s)\,z\big)+s_3^2\,(m\,(-3\,c+z)+t_2)+s_3\,(m\,(c\,(-5+6\,h\,s)+z-2\,h\,s\,z)-h\,s\,t_2)+h_2\,s_2^3\,\big(-c+z+(-1+2\,m)\,t_2+(-1+m)\,h_2^2\,(3\,c-z+2\,t_2)+h_2\,(-c\,(-4+m)+(-2+m)\,z+(3-4\,m)\,t_2)\big)+s_2^2\,\big(3\,c-2\,c\,s-c\,h\,s-z+h\,s\,z+c\,s_3-z\,s_3+2\,t_2-2\,m\,t_2-2\,h\,s\,t_2+2\,h\,m\,s\,t_2+s_3\,t_2-2\,m\,s_3\,t_2+h_2^2\,(5\,c-8\,c\,m-6\,c\,h\,s+6\,c\,h\,m\,s-z+2\,m\,z+2\,h\,s\,z-2\,h\,m\,s\,z+(3-2\,m-4\,h\,s+4\,h\,m\,s)\,t_2-2\,(-1+m)\,s_3\,(3\,c-z+2\,t_2))+h_2\,(-8\,c+4\,c\,m+2\,c\,s+7\,c\,h\,s-2\,c\,m\,s-c\,h\,m\,s+2\,z-2\,m\,z-3\,h\,s\,z+h\,m\,s\,z+(-5+5\,h\,s+m\,(4-6\,h\,s))\,t_2+s_3\,(c\,(-7+m)-(-3+m)\,z+(-4+6\,m)\,t_2))\big)+s_2\,\big(-3\,c\,m+2\,c\,s-5\,c\,h\,s+2\,c\,m\,s+c\,h\,m\,s+3\,c\,h^2\,s^2+m\,z+h\,s\,z-h\,m\,s\,z-h^2\,s^2\,z-2\,h\,s\,t_2+2\,h\,m\,s\,t_2+2\,h^2\,s^2\,t_2-2\,h^2\,m\,s^2\,t_2+s_3^2\,(3\,c-z+(1-2\,m)\,t_2)-s_3\,(c\,(-5+m+6\,h\,s)-(-1+m+2\,h\,s)\,z+(-3+3\,h\,s+m\,(2-4\,h\,s))\,t_2)+h_2\,\big(5\,c\,m-2\,c\,s+5\,c\,h\,s+2\,c\,m\,s-11\,c\,h\,m\,s-3\,c\,h^2\,s^2+3\,c\,h^2\,m\,s^2-m\,z-h\,s\,z+3\,h\,m\,s\,z+h^2\,s^2\,z-h^2\,m\,s^2\,z+h\,s\,(3-2\,h\,s+2\,m\,(-1+h\,s))\,t_2+(-1+m)\,s_3^2\,(3\,c-z+2\,t_2)+s_3\,(c\,(-5+11\,m+6\,h\,s-6\,h\,m\,s)+(1-3\,m-2\,h\,s+2\,h\,m\,s)\,z+(-4+4\,h\,s+m\,(2-4\,h\,s))\,t_2)\big)\big)\big)\Big/$$
$$((-1+s_2)\,(-m+(1+(-1+m)\,h_2)\,s_2)\,(h\,s+(-1+h_2)\,s_2-s_3))$$
$$+O[\zeta^2]$$

Numerically evaluating the approximated eigenvalues for local stability such that $|\lambda_{(1)}| < 1$ and $|\lambda_{(2)}| < 1$ (Tables C15 and C16):

Table C15: Numerically evaluating the approximated eigenvalues to determine local stability as the paramutation rate varies across a given range

| m | $\sim\lambda_{(1)}$ | $\sim\lambda_{(2)}$ | Local Stability |
|---|---|---|---|
| 0.01 | 0.995097 | 0.995841 | Stable |
| 0.06 | 0.944848 | 0.99595 | Stable |
| 0.11 | 0.8946 | 0.995955 | Stable |
| 0.16 | 0.844353 | 0.995957 | Stable |
| 0.21 | 0.794106 | 0.995958 | Stable |
| 0.26 | 0.743859 | 0.995958 | Stable |
| 0.31 | 0.693612 | 0.995959 | Stable |
| 0.36 | 0.643365 | 0.995959 | Stable |
| 0.41 | 0.593118 | 0.995959 | Stable |
| 0.46 | 0.542871 | 0.995959 | Stable |
| 0.51 | 0.492624 | 0.995959 | Stable |
| 0.56 | 0.442377 | 0.99596 | Stable |
| 0.61 | 0.39213 | 0.99596 | Stable |
| 0.66 | 0.341882 | 0.99596 | Stable |
| 0.71 | 0.291635 | 0.99596 | Stable |
| 0.76 | 0.241388 | 0.99596 | Stable |
| 0.81 | 0.191141 | 0.99596 | Stable |
| 0.86 | 0.140894 | 0.99596 | Stable |
| 0.91 | 0.090647 | 0.99596 | Stable |

Parameter values : $s = 0.02$, $s_2 = 0.01$, $s_3 = 0.001$, $h = 0.5$, $h_2 = 0.5$,
$t_1 = 10$ ^ −5, $t_2 = 10$ ^ −4, $u = 10$ ^ −9, $c = 10$ ^ −9, $z = 10$ ^ −9

Table C16: Numerically evaluating the approximated eigenvalues to determine local stability as the forward spontaneous epimutation rate varies across a given range

| $t_1$ | $\sim\lambda_{(1)}$ | $\sim\lambda_{(2)}$ | Local Stability |
|---|---|---|---|
| 0.00001 | 0.90465 | 0.995954 | Stable |
| 0.00011 | 0.904559 | 0.995954 | Stable |
| 0.00021 | 0.904469 | 0.995954 | Stable |
| 0.00031 | 0.904379 | 0.995954 | Stable |
| 0.00041 | 0.904288 | 0.995954 | Stable |
| 0.00051 | 0.904198 | 0.995954 | Stable |
| 0.00061 | 0.904107 | 0.995954 | Stable |
| 0.00071 | 0.904017 | 0.995954 | Stable |
| 0.00081 | 0.903926 | 0.995954 | Stable |
| 0.00091 | 0.903836 | 0.995954 | Stable |
| 0.00101 | 0.903745 | 0.995954 | Stable |

Parameter values: $m = 0.1$, $s = 0.02$, $s_2 = 0.01$, $s_3 = 0.001$, $h = 0.5$, $h_2 = 0.5$,
$u = 10$ ^ −9, $t_2 = 10$ ^ −4, $u = 10$ ^ −9, $c = 10$ ^ −9, $z = 10$ ^ −9

Evaluation of local stability through numerical simulations of the recursion equations (Figure C3). Evidence for local stability was supported through simulations of the recursion equations beginning above (gray line) and below (black line) the equilibrium (dashed line).

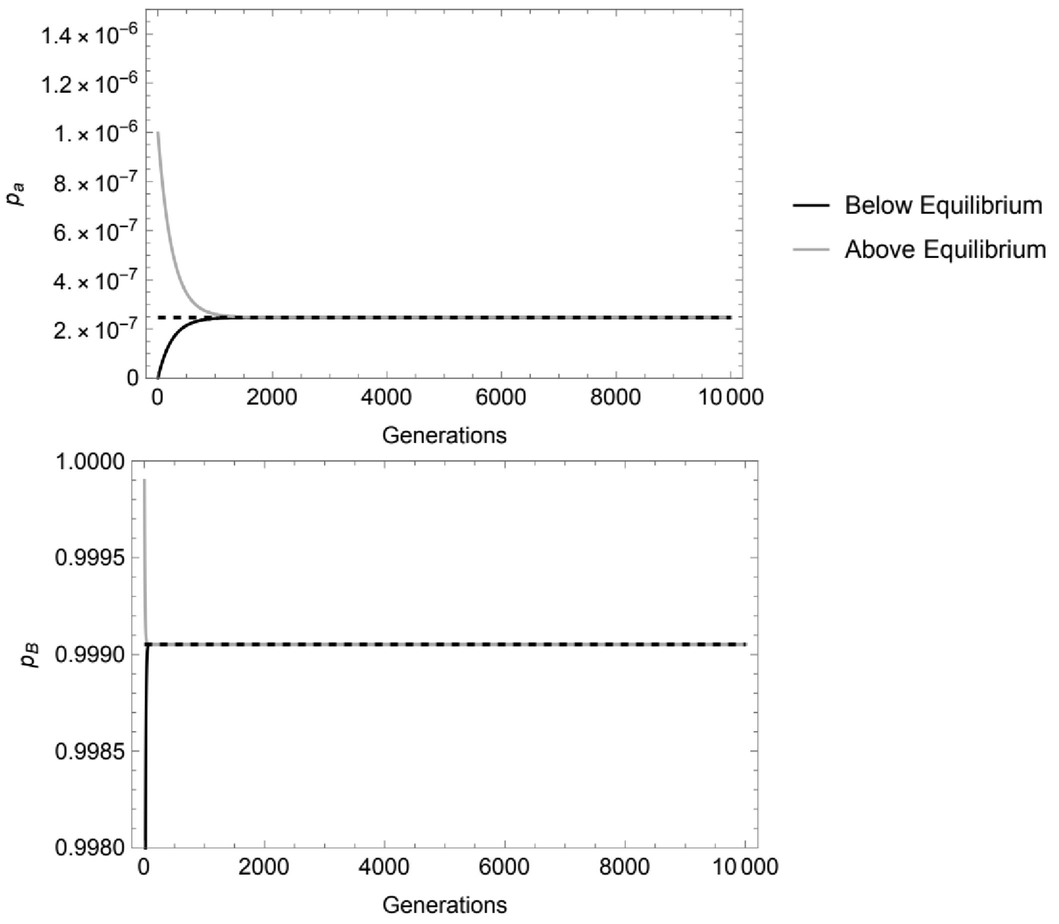


Figure C3: Plots of the deleterious allele frequency (top) and the epiallele frequency (bottom) across time in generations. The numerical parameter values are
$s = 0.02$, $s_2 = 0.01$, $s_3 = 0.001$, $h = 0.5$, $h_2 = 0.5$, $t_1 = 10^{-5}$, $t_2 = 10^{-4}$, $u = 10^{-9}$, $c = 10^{-9}$, $z = 10^{-9}$, $m = 0.11$

The approximated eigenvalues corresponding with eq. 7 of the main text are

$$\lambda_{(1)} \sim 1 - h\,s + \Big(h\,s_3\,t_1 + s_2\,\big(2\,m\,\big((h - h^2\,s)\,t_1 + \big((2 - 5\,h + 3\,h^2\,s)\,u + h\,(1 - h\,s)\,z\big)\,(-1 + t_2)\big) + h_2\,\big(h\,(1 - 2\,h\,s + 2\,m\,(-1 + h\,s))\,t_1 - (-1 + m)\,\big((2 - 5\,h + 3\,h^2\,s)\,u + h\,(1 - h\,s)\,z\big)\,(-1 + t_2)\big)\big) - \big((2 - 5\,h + 3\,h^2\,s)\,u + h\,(1 - h\,s)\,z\big)\,(-m + (1 + m)\,t_2)\Big) \Big/ (h\,(-m + (-2\,m + (-1 + m)\,h_2)\,s_2\,(-1 + t_2) + (1 + m)\,t_2)) + O[\zeta^2]$$

$$\lambda_{(2)} \sim -((1 + m - 2\,m\,s_2 - h_2\,s_2 + m\,h_2\,s_2)\,(-1 + t_2)) + \Big((1 - h\,s + (1 + m + (-2\,m + (-1 + m)\,h_2)\,s_2)\,(-1 + t_2))$$

$$\left(2\,t_1-(-1+m)\,(-1+h_2\,s_2)\,t_1-c\,(1+m+(-2\,m+(-1+m)\,h_2)\,s_2)\,(-1+t_2)+\frac{2\,(-m+(-1+m)\,h_2)\,s_2\,t_1}{-m+(-2\,m+(-1+m)\,h_2)\,s_2\,(-1+t_2)+(1+m)\,t_2}-\frac{(-1+s_2)\,t_1\,(-1+t_2)}{-m+(-2\,m+(-1+m)\,h_2)\,s_2\,(-1+t_2)+(1+m)\,t_2}-\frac{(1+m+(-2\,m+(-1+m)\,h_2)\,s_2)\,t_1\,(-1+t_2)}{-m+(-2\,m+(-1+m)\,h_2)\,s_2\,(-1+t_2)+(1+m)\,t_2}-(-1-m+(2\,m-(-1+m)\,h_2)\,s_2)\,(-1+t_2)\left(-\frac{u}{h\,s}-\frac{t_1}{-m+(-2\,m+(-1+m)\,h_2)\,s_2\,(-1+t_2)+(1+m)\,t_2}\right)+(-1+t_2)\left(-\frac{u\,(-1+h\,s+h_2\,s_2-s_3)}{h\,s}-\frac{(-1+s_2)\,t_1}{-m+(-2\,m+(-1+m)\,h_2)\,s_2\,(-1+t_2)+(1+m)\,t_2}\right)+(2\,(1+m+(-2\,m+(-1+m)\,h_2)\,s_2)\,(-1+t_2)\,(u\,(-m+(1+m)\,t_2)+s_2\,(m\,(t_1-2\,u\,(-1+t_2))-(-1+m)\,h_2\,(u+t_1-u\,t_2))))/(-m+(-2\,m+(-1+m)\,h_2)\,s_2\,(-1+t_2)+(1+m)\,t_2)\right)\Big/(-1+h\,s-(1+m+(-2\,m+(-1+m)\,h_2)\,s_2)\,(-1+t_2))+O[\zeta^2]$$

Numerically evaluating the approximated eigenvalues for local stability such that $|\lambda_{(1)}| < 1$ and $|\lambda_{(2)}| < 1$ (Tables C17 and C18):

Table C17: Numerically evaluating the approximated eigenvalues to determine local stability as the paramutation rate varies across a given range

| m | $\sim\lambda_{(1)}$ | $\sim\lambda_{(2)}$ | Local Stability |
|---|---|---|---|
| 0.005 | 0.99 | 0.79993 | Stable |
| 0.025 | 0.99 | 0.815688 | Stable |
| 0.045 | 0.99 | 0.831446 | Stable |
| 0.065 | 0.99 | 0.847203 | Stable |
| 0.085 | 0.99 | 0.86296 | Stable |
| 0.105 | 0.990001 | 0.878716 | Stable |
| 0.125 | 0.990001 | 0.894471 | Stable |
| 0.145 | 0.990001 | 0.910225 | Stable |
| 0.165 | 0.990001 | 0.925975 | Stable |
| 0.185 | 0.990001 | 0.941721 | Stable |
| 0.205 | 0.990002 | 0.957455 | Stable |

Parameter values: $s = 0.02$, $s_2 = 0.01$, $s_3 = 0.001$, $h = 0.5$,
$h_2 = 0.5$, $t_1 = 10^{\wedge}-5$, $t_2 = 0.2$, $u = 10^{\wedge}-9$, $c = 10^{\wedge}-9$, $z = 10^{\wedge}-9$

Table C18: Numerically evaluating the approximated eigenvalues to determine local stability as the forward spontaneous epimutation rate varies across a given range

| $t_1$ | $\sim\lambda_{(1)}$ | $\sim\lambda_{(2)}$ | Local Stability |
|---|---|---|---|
| 0.00001 | 0.990001 | 0.874777 | Stable |
| 0.00011 | 0.990006 | 0.874551 | Stable |
| 0.00021 | 0.990012 | 0.874325 | Stable |
| 0.00031 | 0.990017 | 0.874099 | Stable |
| 0.00041 | 0.990023 | 0.873872 | Stable |
| 0.00051 | 0.990028 | 0.873646 | Stable |
| 0.00061 | 0.990034 | 0.87342 | Stable |
| 0.00071 | 0.990039 | 0.873194 | Stable |
| 0.00081 | 0.990045 | 0.872967 | Stable |
| 0.00091 | 0.99005 | 0.872741 | Stable |
| 0.00101 | 0.990056 | 0.872515 | Stable |

Parameter values: m = 0.1, s = 0.02, $s_2$ = 0.01, $s_3$ = 0.001, h = 0.5,
$h_2$ = 0.5, u = 10 ^ −9, $t_2$ = 0.2, u = 10 ^ −9, c = 10 ^ −9,
z = 10 ^ −9

Evaluation of local stability through numerical simulations of the recursion equations (Figure C4). Evidence for local stability was supported through simulations of the recursion equations beginning above (gray line) and below (black line) the equilibrium (dashed line).

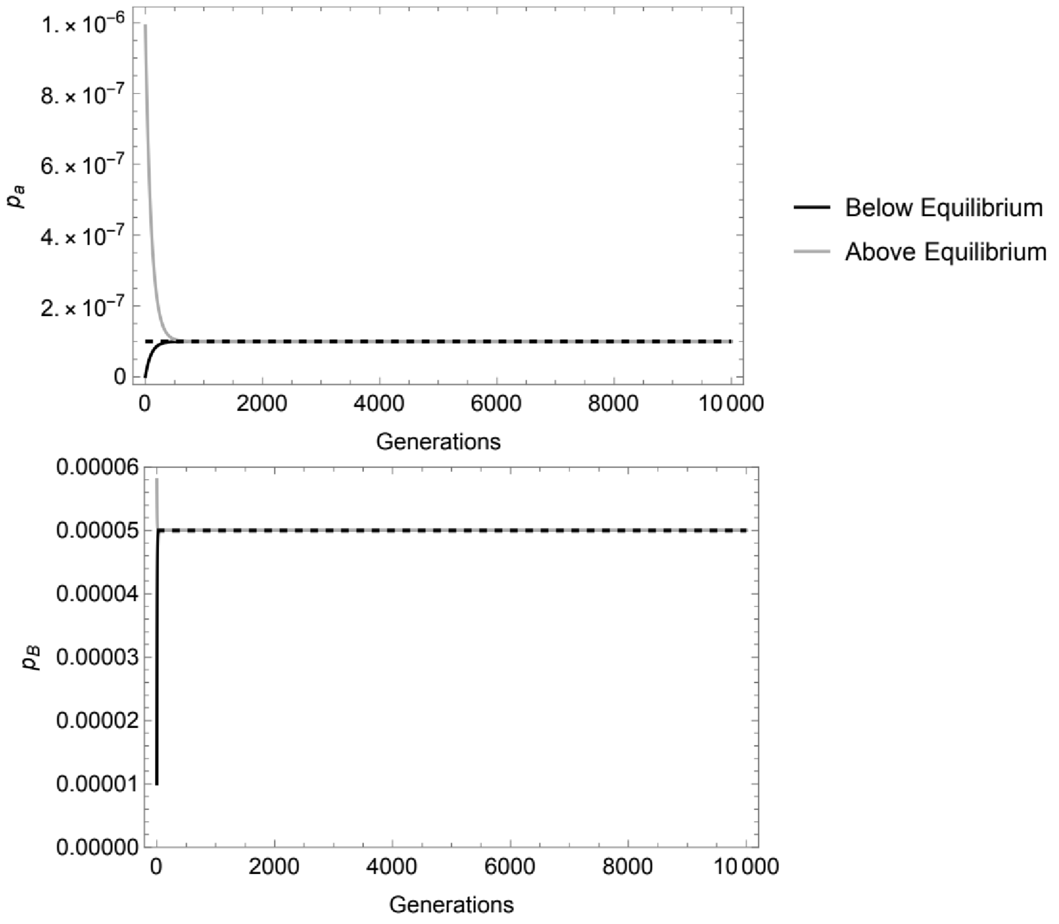


Figure C4: Plots of the deleterious allele frequency (top) and the epiallele frequency (bottom) across time in generations. The numerical parameter values are
s = 0.02, $s_2$ = 0.01, $s_3$ = 0.001, h = 0.5, $h_2$ = 0.5, $t_1$ = 10 ^ −5, $t_2$ = 0.2, u = 10 ^ −9, c = 10 ^ −9, z = 10 ^ −9, m = 0.005

# Case D: With Inbreeding (*f* > 0) and with Paramutation (m > 0)

Evaluating the accuracy of the analytical approximation (eq. 8 of the main text) in comparison to numerical equilibria solutions (Tables D1-D6):

Table D1: Evaluating the accuracy of the equilibrium approximation for the deleterious allele as the paramutation rate increases from a lower to upper limit such that the equilibrium is biologically valid

| m | ~ $\hat{p}_a$ Analytical | $\hat{p}_a$ Numerical | Percentage Difference |
|---|---|---|---|
| 0.001 | $1.\times10^{-7}$ | $1.\times10^{-7}$ | 0 |
| 0.0015 | $1.\times10^{-7}$ | $1.\times10^{-7}$ | 0 |
| 0.002 | $1.\times10^{-7}$ | $1.\times10^{-7}$ | 0 |
| 0.0025 | $1.\times10^{-7}$ | $1.\times10^{-7}$ | 0 |
| 0.003 | $1.\times10^{-7}$ | $1.\times10^{-7}$ | 0 |
| 0.0035 | $1.\times10^{-7}$ | $1.\times10^{-7}$ | 0 |
| 0.004 | $1.\times10^{-7}$ | $1.\times10^{-7}$ | 0 |
| 0.0045 | $1.\times10^{-7}$ | $1.\times10^{-7}$ | 0 |
| 0.005 | $1.\times10^{-7}$ | $1.\times10^{-7}$ | 0 |

Parameter values: $f = 0.5$, $s = 0.02$, $s_2 = 0.01$, $s_3 = 0.001$, $h = 0.5$, $h_2 = 0.5$,
$t_1 = 10^{\wedge}-5$, $t_2 = 10^{\wedge}-4$, $u = 10^{\wedge}-9$, $c = 10^{\wedge}-9$, $z = 10^{\wedge}-9$

Table D2: Evaluating the accuracy of the equilibrium approximation for the epiallele as the paramutation rate increases from a lower to upper limit such that the equilibrium is biologically valid

| m | ~ $\hat{p}_B$ Analytical | $\hat{p}_B$ Numerical | Percentage Difference |
|---|---|---|---|
| 0.001 | 0.0014071 | 0.001427 | 1.39 |
| 0.0015 | 0.0014576 | 0.001479 | 1.45 |
| 0.002 | 0.0015118 | 0.0015349 | 1.5 |
| 0.0025 | 0.0015703 | 0.0015952 | 1.56 |
| 0.003 | 0.0016334 | 0.0016604 | 1.63 |
| 0.0035 | 0.0017019 | 0.0017312 | 1.69 |
| 0.004 | 0.0017763 | 0.0018083 | 1.77 |
| 0.0045 | 0.0018576 | 0.0018926 | 1.85 |
| 0.005 | 0.0019466 | 0.0019851 | 1.94 |

Parameter values: $f = 0.5$, $s = 0.02$, $s_2 = 0.01$, $s_3 = 0.001$, $h = 0.5$, $h_2 = 0.5$,
$t_1 = 10^{\wedge}-5$, $t_2 = 10^{\wedge}-4$, $u = 10^{\wedge}-9$, $c = 10^{\wedge}-9$, $z = 10^{\wedge}-9$

Table D3: Evaluating the accuracy of the equilibrium approximation for the deleterious allele as the forward spontaneous epimutation rate increases from a lower to upper limit

| $t_1$ | ~ $\hat{p}_a$ Analytical | $\hat{p}_a$ Numerical | Percentage Difference |
|---|---|---|---|
| 0.00001 | $1.\times10^{-7}$ | $1.\times10^{-7}$ | 0. |
| 0.00011 | $1.\times10^{-7}$ | $1.\times10^{-7}$ | 0. |
| 0.00021 | $1.\times10^{-7}$ | $1.\times10^{-7}$ | 0. |
| 0.00031 | $1.\times10^{-7}$ | $1.\times10^{-7}$ | 0. |
| 0.00041 | $1.\times10^{-7}$ | $1.\times10^{-7}$ | 0. |
| 0.00051 | $1.\times10^{-7}$ | $1.\times10^{-7}$ | 0. |
| 0.00061 | $1.\times10^{-7}$ | $1.\times10^{-7}$ | 0. |
| 0.00071 | $1.\times10^{-7}$ | $1.\times10^{-7}$ | 0. |
| 0.00081 | $1.\times10^{-7}$ | $1.\times10^{-7}$ | 0. |
| 0.00091 | $1.\times10^{-7}$ | $1.\times10^{-7}$ | 0. |
| 0.00101 | $1.\times10^{-7}$ | $1.\times10^{-7}$ | 0. |

Parameter values: $m = 0.001$, $f = 0.5$, $s = 0.02$, $s_2 = 0.01$, $s_3 = 0.001$, $h = 0.5$,
$h_2 = 0.5$, $u = 10^{\wedge}-9$, $t_2 = 10^{\wedge}-4$, $u = 10^{\wedge}-9$, $c = 10^{\wedge}-9$, $z = 10^{\wedge}-9$

Table D4: Evaluating the accuracy of the equilibrium approximation for the epiallele as the forward spontaneous epimutation rate increases from a lower to upper limit

| $t_1$ | ~ $\hat{p}_B$ Analytical | $\hat{p}_B$ Numerical | Percentage Difference |
|---|---|---|---|
| 0.00001 | 0.0014071 | 0.001427 | 1.39 |
| 0.00011 | 0.0154742 | 0.0156975 | 1.42 |
| 0.00021 | 0.0295346 | 0.0299679 | 1.45 |
| 0.00031 | 0.0435882 | 0.0442383 | 1.47 |
| 0.00041 | 0.0576345 | 0.0585087 | 1.49 |
| 0.00051 | 0.0716734 | 0.0727792 | 1.52 |
| 0.00061 | 0.0857045 | 0.0870496 | 1.55 |
| 0.00071 | 0.0997276 | 0.10132 | 1.57 |
| 0.00081 | 0.113742 | 0.11559 | 1.6 |
| 0.00091 | 0.127748 | 0.129861 | 1.63 |
| 0.00101 | 0.141745 | 0.144131 | 1.66 |

Parameter values: m = 0.001, f = 0.5, s = 0.02, $s_2$ = 0.01, $s_3$ = 0.001, h = 0.5,
$h_2$ = 0.5, u = 10^−9, $t_2$ = 10^−4, u = 10^−9, c = 10^−9, z = 10^−9

Table D5: Evaluating the accuracy of the equilibrium approximation for the deleterious allele as the inbreeding coefficient varies from a lower to upper limit such that the equilibrium is biologically valid

| f | ~ $\hat{p}_a$ Analytical | $\hat{p}_a$ Numerical | Percentage Difference |
|---|---|---|---|
| 0.4 | $1.\times 10^{-7}$ | $1.\times 10^{-7}$ | 0. |
| 0.45 | $1.\times 10^{-7}$ | $1.\times 10^{-7}$ | 0. |
| 0.5 | $1.\times 10^{-7}$ | $1.\times 10^{-7}$ | 0. |
| 0.55 | $1.\times 10^{-7}$ | $1.\times 10^{-7}$ | 0. |
| 0.6 | $1.\times 10^{-7}$ | $1.\times 10^{-7}$ | 0. |
| 0.65 | $1.\times 10^{-7}$ | $1.\times 10^{-7}$ | 0. |
| 0.7 | $1.\times 10^{-7}$ | $1.\times 10^{-7}$ | 0. |
| 0.75 | $1.\times 10^{-7}$ | $1.\times 10^{-7}$ | 0. |
| 0.8 | $1.\times 10^{-7}$ | $1.\times 10^{-7}$ | 0. |
| 0.85 | $1.\times 10^{-7}$ | $1.\times 10^{-7}$ | 0. |
| 0.9 | $1.\times 10^{-7}$ | $1.\times 10^{-7}$ | 0. |

Parameter values: m = 0.01, s = 0.02, $s_2$ = 0.01, $s_3$ = 0.001, h = 0.5, $h_2$ = 0.5,
u = 10^−9, $t_1$ = 10^−5, $t_2$ = 10^−4, c = 10^−9, z = 10^−9

Table D6: Evaluating the accuracy of the equilibrium approximation for the epiallele as the inbreeding coefficient varies from a lower to upper limit such that the equilibrium is biologically valid

| f | ~ $\hat{p}_B$ Analytical | $\hat{p}_B$ Numerical | Percentage Difference |
|---|---|---|---|
| 0.4 | 0.0091743 | 0.0084011 | 8.43 |
| 0.45 | 0.005457 | 0.0051743 | 5.18 |
| 0.5 | 0.0038835 | 0.0037383 | 3.74 |
| 0.55 | 0.0030143 | 0.0029262 | 2.92 |
| 0.6 | 0.0024631 | 0.002404 | 2.4 |
| 0.65 | 0.0020822 | 0.0020399 | 2.03 |
| 0.7 | 0.0018034 | 0.0017716 | 1.76 |
| 0.75 | 0.0015905 | 0.0015657 | 1.56 |
| 0.8 | 0.0014225 | 0.0014026 | 1.4 |
| 0.85 | 0.0012866 | 0.0012703 | 1.27 |
| 0.9 | 0.0011744 | 0.0011609 | 1.15 |

Parameter values: m = 0.01, s = 0.02, $s_2$ = 0.01, $s_3$ = 0.001, h = 0.5, $h_2$ = 0.5,
u = 10 ^ −9, $t_1$ = 10 ^ −5, $t_2$ = 10 ^ −4, c = 10 ^ −9, z = 10 ^ −9

Evaluating the accuracy of the analytical approximation (eq. 9 of the main text) in comparison to numerical equilibria solutions (Tables D7-D12):

Table D7: Evaluating the accuracy of the equilibrium approximation for the deleterious allele as the paramutation rate increases from a lower to upper limit such that the equilibrium is biologically valid

| m | ~ $\hat{p}_a$ Analytical | $\hat{p}_a$ Numerical | Percentage Difference |
|---|---|---|---|
| 0.01 | $2.\times 10^{-7}$ | $2.\times 10^{-7}$ | 0. |
| 0.06 | $2.\times 10^{-7}$ | $2.\times 10^{-7}$ | 0. |
| 0.11 | $2.\times 10^{-7}$ | $2.\times 10^{-7}$ | 0. |
| 0.16 | $2.\times 10^{-7}$ | $2.\times 10^{-7}$ | 0. |
| 0.21 | $2.\times 10^{-7}$ | $2.\times 10^{-7}$ | 0. |
| 0.26 | $2.\times 10^{-7}$ | $2.\times 10^{-7}$ | 0. |
| 0.31 | $2.\times 10^{-7}$ | $2.\times 10^{-7}$ | 0. |
| 0.36 | $2.\times 10^{-7}$ | $2.\times 10^{-7}$ | 0. |
| 0.41 | $2.\times 10^{-7}$ | $2.\times 10^{-7}$ | 0. |
| 0.46 | $2.\times 10^{-7}$ | $2.\times 10^{-7}$ | 0. |
| 0.51 | $2.\times 10^{-7}$ | $2.\times 10^{-7}$ | 0. |
| 0.56 | $2.\times 10^{-7}$ | $2.\times 10^{-7}$ | 0. |
| 0.61 | $2.\times 10^{-7}$ | $2.\times 10^{-7}$ | 0. |
| 0.66 | $2.\times 10^{-7}$ | $2.\times 10^{-7}$ | 0. |
| 0.71 | $2.\times 10^{-7}$ | $2.\times 10^{-7}$ | 0. |
| 0.76 | $2.\times 10^{-7}$ | $2.\times 10^{-7}$ | 0. |
| 0.81 | $2.\times 10^{-7}$ | $2.\times 10^{-7}$ | 0. |
| 0.86 | $2.\times 10^{-7}$ | $2.\times 10^{-7}$ | 0. |
| 0.91 | $2.\times 10^{-7}$ | $2.\times 10^{-7}$ | 0. |

Parameter values : f = 0.1, s = 0.02, $s_2$ = 0.01, $s_3$ = 0.001, h = 0.5 $h_2$ = 0.5,
$t_1$ = 10 ^ −5, $t_2$ = 10 ^ −4, u = 10 ^ −9, c = 10 ^ −9, z = 10 ^ −9

Table D8: Evaluating the accuracy of the equilibrium approximation for the epiallele as the paramutation rate increases from a lower to upper limit such that the equilibrium is biologically valid

| m | ~ $\hat{p}_B$ Analytical | $\hat{p}_B$ Numerical | Percentage Difference |
|---|---|---|---|
| 0.01 | 0.971397 | 0.971346 | 0.01 |
| 0.06 | 0.997947 | 0.997947 | 0. |
| 0.11 | 0.998935 | 0.998935 | 0. |
| 0.16 | 0.999281 | 0.999281 | 0. |
| 0.21 | 0.999457 | 0.999458 | 0. |
| 0.26 | 0.999564 | 0.999564 | 0. |
| 0.31 | 0.999636 | 0.999636 | 0. |
| 0.36 | 0.999687 | 0.999687 | 0. |
| 0.41 | 0.999726 | 0.999726 | 0. |
| 0.46 | 0.999756 | 0.999756 | 0. |
| 0.51 | 0.99978 | 0.99978 | 0. |
| 0.56 | 0.9998 | 0.9998 | 0. |
| 0.61 | 0.999817 | 0.999817 | 0. |
| 0.66 | 0.999831 | 0.999831 | 0. |
| 0.71 | 0.999843 | 0.999843 | 0. |
| 0.76 | 0.999853 | 0.999853 | 0. |
| 0.81 | 0.999862 | 0.999862 | 0. |
| 0.86 | 0.99987 | 0.99987 | 0. |
| 0.91 | 0.999878 | 0.999878 | 0. |

Parameter values: f = 0.1, s = 0.02, $s_2$ = 0.01, $s_3$ = 0.001, h = 0.5, $h_2$ = 0.5,
$t_1$ = 10 ^ −5, $t_2$ = 10 ^ −4, u = 10 ^ −9, c = 10 ^ −9, z = 10 ^ −9

Table D9: Evaluating the accuracy of the equilibrium approximation for the deleterious allele as the forward spontaneous epimutation rate increases from a lower to upper limit

| $t_1$ | ~ $\hat{p}_a$ Analytical | $\hat{p}_a$ Numerical | Percentage Difference |
|---|---|---|---|
| 0.00001 | $2. \times 10^{-7}$ | $2. \times 10^{-7}$ | 0. |
| 0.00011 | $2. \times 10^{-7}$ | $2. \times 10^{-7}$ | 0. |
| 0.00021 | $2. \times 10^{-7}$ | $2. \times 10^{-7}$ | 0. |
| 0.00031 | $2. \times 10^{-7}$ | $2. \times 10^{-7}$ | 0. |
| 0.00041 | $2. \times 10^{-7}$ | $2. \times 10^{-7}$ | 0. |
| 0.00051 | $2. \times 10^{-7}$ | $2. \times 10^{-7}$ | 0. |
| 0.00061 | $2. \times 10^{-7}$ | $2. \times 10^{-7}$ | 0. |
| 0.00071 | $2. \times 10^{-7}$ | $2. \times 10^{-7}$ | 0. |
| 0.00081 | $2. \times 10^{-7}$ | $2. \times 10^{-7}$ | 0. |
| 0.00091 | $2. \times 10^{-7}$ | $2. \times 10^{-7}$ | 0. |
| 0.00101 | $2. \times 10^{-7}$ | $2. \times 10^{-7}$ | 0. |

Parameter values: f = 0.1, m = 0.1, s = 0.02, $s_2$ = 0.01, $s_3$ = 0.001, h = 0.5,
$h_2$ = 0.5, u = 10 ^ −9, $t_2$ = 10 ^ −4, u = 10 ^ −9, c = 10 ^ −9,
z = 10 ^ −9

Table D10: Evaluating the accuracy of the equilibrium approximation for the epiallele as the forward spontaneous epimutation rate increases from a lower to upper limit

| $t_1$ | ~ $\hat{p}_B$ Analytical | $\hat{p}_B$ Numerical | Percentage Difference |
|---|---|---|---|
| 0.00001 | 0.998822 | 0.998822 | 0. |
| 0.00011 | 0.998823 | 0.998822 | 0. |
| 0.00021 | 0.998824 | 0.998822 | 0. |
| 0.00031 | 0.998826 | 0.998822 | 0. |
| 0.00041 | 0.998827 | 0.998822 | 0. |
| 0.00051 | 0.998828 | 0.998822 | 0. |
| 0.00061 | 0.99883 | 0.998822 | 0. |
| 0.00071 | 0.998831 | 0.998822 | 0. |
| 0.00081 | 0.998832 | 0.998822 | 0. |
| 0.00091 | 0.998833 | 0.998822 | 0. |
| 0.00101 | 0.998835 | 0.998822 | 0. |

Parameter values: f = 0.1, m = 0.1, s = 0.02, $s_2$ = 0.01, $s_3$ = 0.001, h = 0.5,
$h_2$ = 0.5, u = 10 ^ −9, $t_2$ = 10 ^ −4, u = 10 ^ −9, c = 10 ^ −9,
z = 10 ^ −9

Table D11: Evaluating the accuracy of the equilibrium approximation for the deleterious allele as the inbreeding coefficient varies from a lower to upper limit such that the equilibrium is biologically valid

| f | ~ $\hat{p}_a$ Analytical | $\hat{p}_a$ Numerical | Percentage Difference |
|---|---|---|---|
| 0.01 | $2.\times 10^{-7}$ | $2.\times 10^{-7}$ | 0. |
| 0.03 | $2.\times 10^{-7}$ | $2.\times 10^{-7}$ | 0. |
| 0.05 | $2.\times 10^{-7}$ | $2.\times 10^{-7}$ | 0. |
| 0.07 | $2.\times 10^{-7}$ | $2.\times 10^{-7}$ | 0. |
| 0.09 | $2.\times 10^{-7}$ | $2.\times 10^{-7}$ | 0. |
| 0.11 | $2.\times 10^{-7}$ | $2.\times 10^{-7}$ | 0. |
| 0.13 | $2.\times 10^{-7}$ | $2.\times 10^{-7}$ | 0. |
| 0.15 | $2.\times 10^{-7}$ | $2.\times 10^{-7}$ | 0. |
| 0.17 | $2.\times 10^{-7}$ | $2.\times 10^{-7}$ | 0. |
| 0.19 | $2.\times 10^{-7}$ | $2.\times 10^{-7}$ | 0. |
| 0.21 | $2.\times 10^{-7}$ | $2.\times 10^{-7}$ | 0. |
| 0.23 | $2.\times 10^{-7}$ | $2.\times 10^{-7}$ | 0. |
| 0.25 | $2.\times 10^{-7}$ | $2.\times 10^{-7}$ | 0. |
| 0.27 | $2.\times 10^{-7}$ | $2.\times 10^{-7}$ | 0. |
| 0.29 | $2.\times 10^{-7}$ | $2.\times 10^{-7}$ | 0. |

Parameter values : m = 0.1, s = 0.02, $s_2$ = 0.01, $s_3$ = 0.001, h = 0.5, $h_2$ = 0.5,
u = 10 ^ −9, $t_1$ = 10 ^ −5, $t_2$ = 10 ^ −4, u = 10 ^ −9, c = 10 ^ −9, z = 10 ^ −9

Table D12: Evaluating the accuracy of the equilibrium approximation for the epiallele as the inbreeding coefficient varies from a lower to upper limit

| f | ~ $\hat{p}_B$ Analytical | $\hat{p}_B$ Numerical | Percentage Difference |
|---|---|---|---|
| 0.01 | 0.99894 | 0.99894 | 0. |
| 0.03 | 0.998916 | 0.998916 | 0. |
| 0.05 | 0.998891 | 0.998891 | 0. |
| 0.07 | 0.998864 | 0.998864 | 0. |
| 0.09 | 0.998836 | 0.998836 | 0. |
| 0.11 | 0.998807 | 0.998807 | 0. |
| 0.13 | 0.998776 | 0.998776 | 0. |
| 0.15 | 0.998744 | 0.998744 | 0. |
| 0.17 | 0.99871 | 0.99871 | 0. |
| 0.19 | 0.998674 | 0.998674 | 0. |
| 0.21 | 0.998635 | 0.998635 | 0. |
| 0.23 | 0.998595 | 0.998595 | 0. |
| 0.25 | 0.998552 | 0.998552 | 0. |
| 0.27 | 0.998506 | 0.998506 | 0. |
| 0.29 | 0.998458 | 0.998458 | 0. |

Parameter values: $m = 0.1$, $s = 0.02$, $s_2 = 0.01$, $s_3 = 0.001$, $h = 0.5$,
$h_2 = 0.5$, $u = 10$ ^ −9, $t_1 = 10$ ^ −5, $t_2 = 10$ ^ −4, $u = 10$ ^ −9, $c = 10$ ^ −9, $z = 10$ ^ −9

Evaluating the accuracy of the analytical approximation (eq. 10 of the main text) in comparison to numerical equilibria solutions (Tables D13-D18):

Table D13: Evaluating the accuracy of the equilibrium approximation for the deleterious allele as the paramutation rate increases from a lower to upper limit such that the equilibrium is biologically valid

| m | ~ $\hat{p}_a$ Analytical | $\hat{p}_a$ Numerical | Percentage Difference |
|---|---|---|---|
| 0.005 | $1. \times 10^{-7}$ | $1. \times 10^{-7}$ | 0. |
| 0.025 | $1. \times 10^{-7}$ | $1. \times 10^{-7}$ | 0. |
| 0.045 | $1. \times 10^{-7}$ | $1. \times 10^{-7}$ | 0. |
| 0.065 | $1. \times 10^{-7}$ | $1. \times 10^{-7}$ | 0. |
| 0.085 | $1. \times 10^{-7}$ | $1. \times 10^{-7}$ | 0. |
| 0.105 | $1. \times 10^{-7}$ | $1. \times 10^{-7}$ | 0. |
| 0.125 | $1. \times 10^{-7}$ | $1. \times 10^{-7}$ | 0. |
| 0.145 | $1. \times 10^{-7}$ | $1. \times 10^{-7}$ | 0. |
| 0.165 | $1. \times 10^{-7}$ | $1. \times 10^{-7}$ | 0. |
| 0.185 | $1. \times 10^{-7}$ | $1. \times 10^{-7}$ | 0. |
| 0.205 | $1. \times 10^{-7}$ | $1. \times 10^{-7}$ | 0. |

Parameter values: $f = 0.1$, $s = 0.02$, $s_2 = 0.01$, $s_3 = 0.001$, $h = 0.5$, $h_2 = 0.5$,
$u = 10$ ^ −9, $t_1 = 10$ ^ −5, $t_2 = 0.2$, $u = 10$ ^ −9, $c = 10$ ^ −9, $z = 10$ ^ −9

Table D14: Evaluating the accuracy of the equilibrium approximation for the epiallele as the paramutation rate increases from a lower to upper limit such that the equilibrium is biologically valid

| m | ~ $\hat{p}_B$ Analytical | $\hat{p}_B$ Numerical | Percentage Difference |
|---|---|---|---|
| 0.005 | 0.0000498 | 0.0000498 | 0. |
| 0.025 | 0.0000536 | 0.0000536 | 0. |
| 0.045 | 0.000058 | 0.000058 | 0. |
| 0.065 | 0.0000632 | 0.0000632 | 0. |
| 0.085 | 0.0000694 | 0.0000694 | 0. |
| 0.105 | 0.000077 | 0.000077 | 0. |
| 0.125 | 0.0000864 | 0.0000864 | 0. |
| 0.145 | 0.0000984 | 0.0000985 | 0.1 |
| 0.165 | 0.0001144 | 0.0001144 | 0. |
| 0.185 | 0.0001366 | 0.0001366 | 0. |
| 0.205 | 0.0001694 | 0.0001695 | 0.06 |

Parameter values: f = 0.1, s = 0.02, $s_2$ = 0.01, $s_3$ = 0.001, h = 0.5, $h_2$ = 0.5,
u = 10 ^ −9, $t_1$ = 10 ^ −5, $t_2$ = 0.2, u = 10 ^ −9, c = 10 ^ −9, z = 10 ^ −9

Table D15: Evaluating the accuracy of the equilibrium approximation for the deleterious allele as the forward spontaneous epimutation rate increases from a lower to upper limit

| $t_1$ | ~ $\hat{p}_a$ Analytical | $\hat{p}_a$ Numerical | Percentage Difference |
|---|---|---|---|
| 0.00001 | $1.\times 10^{-7}$ | $1.\times 10^{-7}$ | 0. |
| 0.00011 | $1.\times 10^{-7}$ | $1.\times 10^{-7}$ | 0. |
| 0.00021 | $1.\times 10^{-7}$ | $1.\times 10^{-7}$ | 0. |
| 0.00031 | $1.\times 10^{-7}$ | $1.\times 10^{-7}$ | 0. |
| 0.00041 | $1.\times 10^{-7}$ | $1.\times 10^{-7}$ | 0. |
| 0.00051 | $1.\times 10^{-7}$ | $1.\times 10^{-7}$ | 0. |
| 0.00061 | $1.\times 10^{-7}$ | $1.\times 10^{-7}$ | 0. |
| 0.00071 | $1.\times 10^{-7}$ | $1.\times 10^{-7}$ | 0. |
| 0.00081 | $1.\times 10^{-7}$ | $1.\times 10^{-7}$ | 0. |
| 0.00091 | $1.\times 10^{-7}$ | $1.\times 10^{-7}$ | 0. |
| 0.00101 | $1.\times 10^{-7}$ | $1.\times 10^{-7}$ | 0. |

Parameter values: m = 0.1, f = 0.1, s = 0.02, $s_2$ = 0.01, $s_3$ = 0.001, h = 0.5,
$h_2$ = 0.5, u = 10 ^ −9, $t_2$ = 0.2, u = 10 ^ −9, c = 10 ^ −9,
z = 10 ^ −9

Table D16: Evaluating the accuracy of the equilibrium approximation for the epiallele as the forward spontaneous epimutation rate increases from a lower to upper limit

| $t_1$ | ~ $\hat{p}_B$ Analytical | $\hat{p}_B$ Numerical | Percentage Difference |
|---|---|---|---|
| 0.00001 | 0.0000749 | 0.0000749 | 0. |
| 0.00011 | 0.0008241 | 0.000823 | 0.13 |
| 0.00021 | 0.0015733 | 0.0015694 | 0.25 |
| 0.00031 | 0.0023224 | 0.002314 | 0.36 |
| 0.00041 | 0.0030716 | 0.0030569 | 0.48 |
| 0.00051 | 0.0038208 | 0.003798 | 0.6 |
| 0.00061 | 0.00457 | 0.0045375 | 0.71 |
| 0.00071 | 0.0053191 | 0.0052752 | 0.83 |
| 0.00081 | 0.0060683 | 0.0060112 | 0.94 |
| 0.00091 | 0.0068175 | 0.0067455 | 1.06 |
| 0.00101 | 0.0075667 | 0.0074782 | 1.17 |

Parameter values: m = 0.1, f = 0.1, s = 0.02, $s_2$ = 0.01, $s_3$ = 0.001, h = 0.5,
$h_2$ = 0.5, u = 10 ^ −9, $t_2$ = 0.2, u = 10 ^ −9, c = 10 ^ −9,
z = 10 ^ −9

Table D17: Evaluating the accuracy of the equilibrium approximation for the deleterious allele as the inbreeding coefficient varies from a lower to upper limit such that the equilibrium is biologically valid

| f | ~ $\hat{p}_a$ Analytical | $\hat{p}_a$ Numerical | Percentage Difference |
|---|---|---|---|
| 0.01 | $1. \times 10^{-7}$ | $1. \times 10^{-7}$ | 0. |
| 0.06 | $1. \times 10^{-7}$ | $1. \times 10^{-7}$ | 0. |
| 0.11 | $1. \times 10^{-7}$ | $1. \times 10^{-7}$ | 0. |
| 0.16 | $1. \times 10^{-7}$ | $1. \times 10^{-7}$ | 0. |
| 0.21 | $1. \times 10^{-7}$ | $1. \times 10^{-7}$ | 0. |
| 0.26 | $1. \times 10^{-7}$ | $1. \times 10^{-7}$ | 0. |
| 0.31 | $1. \times 10^{-7}$ | $1. \times 10^{-7}$ | 0. |
| 0.36 | $1. \times 10^{-7}$ | $1. \times 10^{-7}$ | 0. |
| 0.41 | $1. \times 10^{-7}$ | $1. \times 10^{-7}$ | 0. |
| 0.46 | $1. \times 10^{-7}$ | $1. \times 10^{-7}$ | 0. |

Parameter values: $m = 0.1$, $s = 0.02$, $s_2 = 0.01$, $s_3 = 0.001$, $h = 0.5$, $h_2 = 0.5$,
$u = 10\,\hat{}\,{-9}$, $t_1 = 10\,\hat{}\,{-5}$, $t_2 = 0.2$, $u = 10\,\hat{}\,{-9}$, $c = 10\,\hat{}\,{-9}$, $z = 10\,\hat{}\,{-9}$

Table D18: Evaluating the accuracy of the equilibrium approximation for the epiallele as the inbreeding coefficient varies from a lower to upper limit such that the equilibrium is biologically valid

| f | ~ $\hat{p}_B$ Analytical | $\hat{p}_B$ Numerical | Percentage Difference |
|---|---|---|---|
| 0.01 | 0.0000793 | 0.0000793 | 0. |
| 0.06 | 0.0000768 | 0.0000768 | 0. |
| 0.11 | 0.0000745 | 0.0000744 | 0.13 |
| 0.16 | 0.0000722 | 0.0000722 | 0. |
| 0.21 | 0.0000701 | 0.0000701 | 0. |
| 0.26 | 0.0000682 | 0.0000681 | 0.15 |
| 0.31 | 0.0000663 | 0.0000663 | 0. |
| 0.36 | 0.0000645 | 0.0000645 | 0. |
| 0.41 | 0.0000628 | 0.0000628 | 0. |
| 0.46 | 0.0000612 | 0.0000612 | 0. |

Parameter values: $m = 0.1$, $s = 0.02$, $s_2 = 0.01$, $s_3 = 0.001$, $h = 0.5$, $h_2 = 0.5$,
$u = 10\,\hat{}\,{-9}$, $t_1 = 10\,\hat{}\,{-5}$, $t_2 = 0.2$, $u = 10\,\hat{}\,{-9}$, $c = 10\,\hat{}\,{-9}$, $z = 10\,\hat{}\,{-9}$

Following the main text (see case D results and table 7, equilibrium 2), an equilibrium arises mirroring case C where the epiallele and wild-type allele are at moderate to high frequencies and the deleterious allele is at a low frequency. The key effect of inbreeding is to decrease the epiallele at equilibrium by reducing the effective paramutation rate (See figure D1) and increases the wild-type allele (not shown).

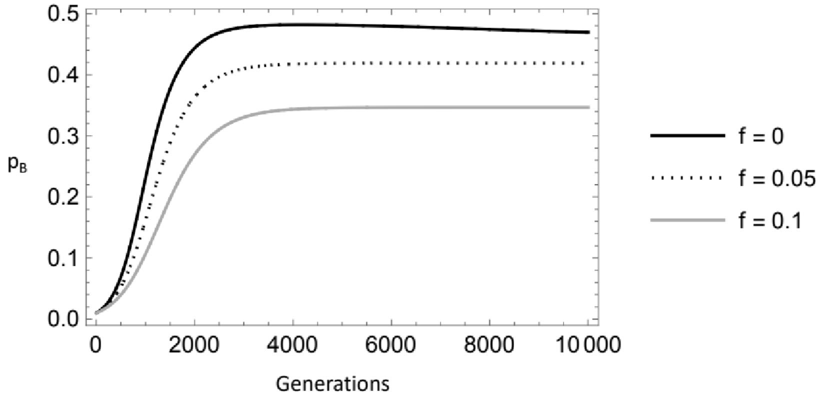


Figure D1: A plot of numerical simulations showing the epiallele frequency across time. The epiallele decreases with increased inbreeding going from the black line to the dotted line to the gray line. Other parameter values were held constant:
s = 0.02, $s_2$ = 0.01, $s_3$ = 0.0005, h = 0.1, $h_2$ = 0.1, $t_1$ = 10 ^ –5, $t_2$ = 5 x 10 ^ –5, u = 10 ^ –9, c = 10 ^ –9, z = 10 ^ –9, m = 0.005

## Local Stability analysis (following Appendix Section A2 )

The approximated eigenvalues corresponding with eq. 8 of the main text are

$$\begin{gathered}
\lambda_{(1)} \sim 1-f s-h s+f h s+ \\
\left((-1+f)\left(m\left(2-5 h+3 h^2 s+f^2\left(1-4 h+3 h^2\right) s+f\left(-3-6 h^2 s+h(5+4 s)\right)\right) u-(f(-1+h)-h) m(1+f(-1+h) s-h s) z+(f+h-f h) s_3 t_1\right)+ \\
s_2\left(-\left((-2 m+f(-1+2 m))\left(\left(2-5 h+3 h^2 s+f^2\left(1-4 h+3 h^2\right) s+f\left(-3+5 h+4 h s-6 h^2 s\right)\right) u-\right.\right.\right. \\
\left.\left.(f(-1+h)-h)(1+f(-1+h) s-h s) z+(f(-1+h)-h)(1+f(-1+h) s-h s) t_1\right)\right)+(-1+f) h_2 \\
\left((-1+m)\left(\left(2-5 h+3 h^2 s+f^2\left(1-4 h+3 h^2\right) s+f\left(-3+5 h+4 h s-6 h^2 s\right)\right) u-(f(-1+h)-h)(1+f(-1+h) s-h s) z\right)+\right. \\
\left.\left.\left.(f(-1+h)-h)(-1-2 f(-1+h) s+2 h s+2 m(1+f(-1+h) s-h s)) t_1\right)\right)\right) / \\
\left((f(-1+h)-h)\left((-1+f) m+\left(f+2 m-2 f m+(-1+f)(-1+m) h_2\right) s_2\right)\right)+ \\
O\left[\zeta^2\right]
\end{gathered}$$

$$\begin{gathered}
\lambda_{(2)} \sim 1+m-f m-f s_2-2 m s_2+2 f m s_2-h_2 s_2+f h_2 s_2+m h_2 s_2-f m h_2 s_2+ \\
\left((-1+f) m\left(c f s+c h s-c f h s+c f m s-c f^2 m s+c h m s-2 c f h m s+c f^2 h m s+m u-\right.\right. \\
f m u-f s u-h s u+f h s u-f m s u+f^2 m s u-2 h m s u+4 f h m s u-2 f^2 h m s u+(-1+f) u s_3+ \\
\left.(f(-1+h)-h)(1+(-1+f) m) s t_1+f s t_2+h s t_2-f h s t_2+f m s t_2-f^2 m s t_2+h m s t_2-2 f h m s t_2+f^2 h m s t_2\right)+ \\
\left(f+2 m-2 f m+(-1+f)(-1+m) h_2\right) s_2^2\left(-c f^2 s-c f h s+c f^2 h s-2 c f m s+2 c f^2 m s-2 c h m s+4 c f h m s-\right. \\
2 c f^2 h m s-2 m u+2 f m u+f^2 s u+2 f h s u-2 f^2 h s u+2 f m s u-2 f^2 m s u+4 h m s u-8 f h m s u+ \\
4 f^2 h m s u+(f(-1+h)-h)(-2 m+f(-1+2 m)) s t_1-f^2 s t_2-f h s t_2+f^2 h s t_2-2 f m s t_2+2 f^2 m s t_2-2 h m s t_2+ \\
4 f h m s t_2-2 f^2 h m s t_2+(-1+f) h_2(c f s+c h s-c f h s-c f m s-c h m s+c f h m s-m u-f s u-2 h s u+ \\
\left.2 f h s u+f m s u+2 h m s u-2 f h m s u-3(f(-1+h)-h)(-1+m) s t_1+(f(-1+h)-h)(-1+m) s t_2)\right)+ \\
s_2\left(c f^2 s+c f h s-c f^2 h s+2 c f m s-2 c f^3 m s+2 c h m s-2 c f h m s-2 c f^2 h m s+2 c f^3 h m s+4 c f m^2 s-\right. \\
8 c f^2 m^2 s+4 c f^3 m^2 s+4 c h m^2 s-12 c f h m^2 s+12 c f^2 h m^2 s-4 c f^3 h m^2 s+f m u-f^2 m u+4 m^2 u-8 f m^2 u+ \\
4 f^2 m^2 u-f^2 s u-f h s u+f^2 h s u-2 f m s u+2 f^3 m s u-2 h m s u+6 f^2 h m s u-4 f^3 h m s u-4 f m^2 s u+ \\
8 f^2 m^2 s u-4 f^3 m^2 s u-8 h m^2 s u+24 f h m^2 s u-24 f^2 h m^2 s u+8 f^3 h m^2 s u-f u s_3+f^2 u s_3-2 m u s_3+ \\
4 f m u s_3-2 f^2 m u s_3+2 f s t_1-3 f^2 s t_1+2 h s t_1-5 f h s t_1+3 f^2 h s t_1-6 f m s t_1+6 f^2 m s t_1-6 h m s t_1+ \\
12 f h m s t_1-6 f^2 h m s t_1+f^2 s t_2+f h s t_2-f^2 h s t_2+2 f m s t_2-2 f^3 m s t_2+2 h m s t_2-2 f h m s t_2-2 f^2 h m s t_2+
\end{gathered}$$

$$2 f^3 h m s t_2 + 4 f m^2 s t_2 - 8 f^2 m^2 s t_2 + 4 f^3 m^2 s t_2 + 4 h m^2 s t_2 - 12 f h m^2 s t_2 + 12 f^2 h m^2 s t_2 - 4 f^3 h m^2 s t_2 +$$
$$(-1+f) h_2 (-c f s - c h s + c f h s - c f m s + 2 c f^2 m s - c h m s + 3 c f h m s - 2 c f^2 h m s + 2 c f m^2 s - 2 c f^2 m^2 s +$$
$$2 c h m^2 s - 4 c f h m^2 s + 2 c f^2 h m^2 s - m u + f m u + 2 m^2 u - 2 f m^2 u + f s u + h s u - f h s u + f m s u -$$
$$2 f^2 m s u + 3 h m s u - 7 f h m s u + 4 f^2 h m s u - 2 f m^2 s u + 2 f^2 m^2 s u - 4 h m^2 s u + 8 f h m^2 s u - 4 f^2 h m^2 s u +$$
$$(-1+f)(-1+m) u s_3 - (f(-1+h) - h)(-1+m)(-5 + 2(-1+f) m) s t_1 - f s t_2 - h s t_2 + f h s t_2 - f m s t_2 + 2 f^2 m s t_2 -$$
$$h m s t_2 + 3 f h m s t_2 - 2 f^2 h m s t_2 + 2 f m^2 s t_2 - 2 f^2 m^2 s t_2 + 2 h m^2 s t_2 - 4 f h m^2 s t_2 + 2 f^2 h m^2 s t_2))) /$$
$$((f(-1+h) - h) s ((-1+f) m + (f + 2 m - 2 f m + (-1+f)(-1+m) h_2) s_2))$$
$$+O[\zeta^2]$$

Numerically evaluating the approximated eigenvalues for local stability such that $|\lambda_{(1)}| < 1$ and $|\lambda_{(2)}| < 1$ (Tables D19-D21):

Table D19: Numerically evaluating the approximated eigenvalues to determine local stability as the paramutation rate varies across a given range

| m | $\sim\lambda_{(1)}$ | $\sim\lambda_{(2)}$ | Local Stability |
|---|---|---|---|
| 0.001 | 0.985011 | 0.992903 | Stable |
| 0.0015 | 0.985012 | 0.993149 | Stable |
| 0.002 | 0.985012 | 0.993396 | Stable |
| 0.0025 | 0.985013 | 0.993642 | Stable |
| 0.003 | 0.985013 | 0.993888 | Stable |
| 0.0035 | 0.985014 | 0.994134 | Stable |
| 0.004 | 0.985014 | 0.99438 | Stable |
| 0.0045 | 0.985015 | 0.994627 | Stable |
| 0.005 | 0.985016 | 0.994873 | Stable |

Parameter values: f = 0.5, s = 0.02, $s_2$ = 0.01, $s_3$ = 0.001, h = 0.5, $h_2$ = 0.5,
$t_1$ = 10 ^ −5, $t_2$ = 10 ^ −4, u = 10 ^ −9, c = 10 ^ −9, z = 10 ^ −9

Table D20: Numerically evaluating the approximated eigenvalues to determine local stability as the forward spontaneous epimutation rate varies across a given range

| $t_1$ | $\sim\lambda_{(1)}$ | $\sim\lambda_{(2)}$ | Local Stability |
|---|---|---|---|
| 0.00001 | 0.985011 | 0.992903 | Stable |
| 0.00011 | 0.985123 | 0.993002 | Stable |
| 0.00021 | 0.985235 | 0.993101 | Stable |
| 0.00031 | 0.985347 | 0.9932 | Stable |
| 0.00041 | 0.98546 | 0.993298 | Stable |
| 0.00051 | 0.985572 | 0.993397 | Stable |
| 0.00061 | 0.985684 | 0.993496 | Stable |
| 0.00071 | 0.985796 | 0.993595 | Stable |
| 0.00081 | 0.985908 | 0.993694 | Stable |
| 0.00091 | 0.98602 | 0.993793 | Stable |
| 0.00101 | 0.986132 | 0.993892 | Stable |

Parameter values: m = 0.001, f = 0.5, s = 0.02, $s_2$ = 0.01, $s_3$ = 0.001,
h = 0.5, $h_2$ = 0.5, u = 10 ^ −9, $t_2$ = 10 ^ −4, u = 10 ^ −9,
c = 10 ^ −9, z = 10 ^ −9

Table D21: Numerically evaluating the approximated eigenvalues to determine local stability as the inbreeding coefficient varies across a given range

| f | $\sim\lambda_{(1)}$ | $\sim\lambda_{(2)}$ | Local Stability |
|---|---|---|---|
| 0.4 | 0.986069 | 0.998821 | Stable |
| 0.45 | 0.985542 | 0.998078 | Stable |
| 0.5 | 0.985031 | 0.997335 | Stable |
| 0.55 | 0.984524 | 0.996593 | Stable |
| 0.6 | 0.98402 | 0.99585 | Stable |
| 0.65 | 0.983518 | 0.995108 | Stable |
| 0.7 | 0.983016 | 0.994366 | Stable |
| 0.75 | 0.982514 | 0.993623 | Stable |
| 0.8 | 0.982013 | 0.992881 | Stable |
| 0.85 | 0.981512 | 0.992138 | Stable |
| 0.9 | 0.981011 | 0.991396 | Stable |

Parameter values: m = 0.01, s = 0.02, $s_2$ = 0.01, $s_3$ = 0.001, h = 0.5,
$h_2$ = 0.5, u = 10^−9, $t_1$ = 10^−5, $t_2$ = 10^−4, c = 10^−9, z = 10^−9

Evaluation of local stability through numerical simulations of the recursion equations (Figure D2). Evidence for local stability was supported through simulations of the recursion equations beginning above (dotted line) and below (solid line) the equilibrium (dashed line).

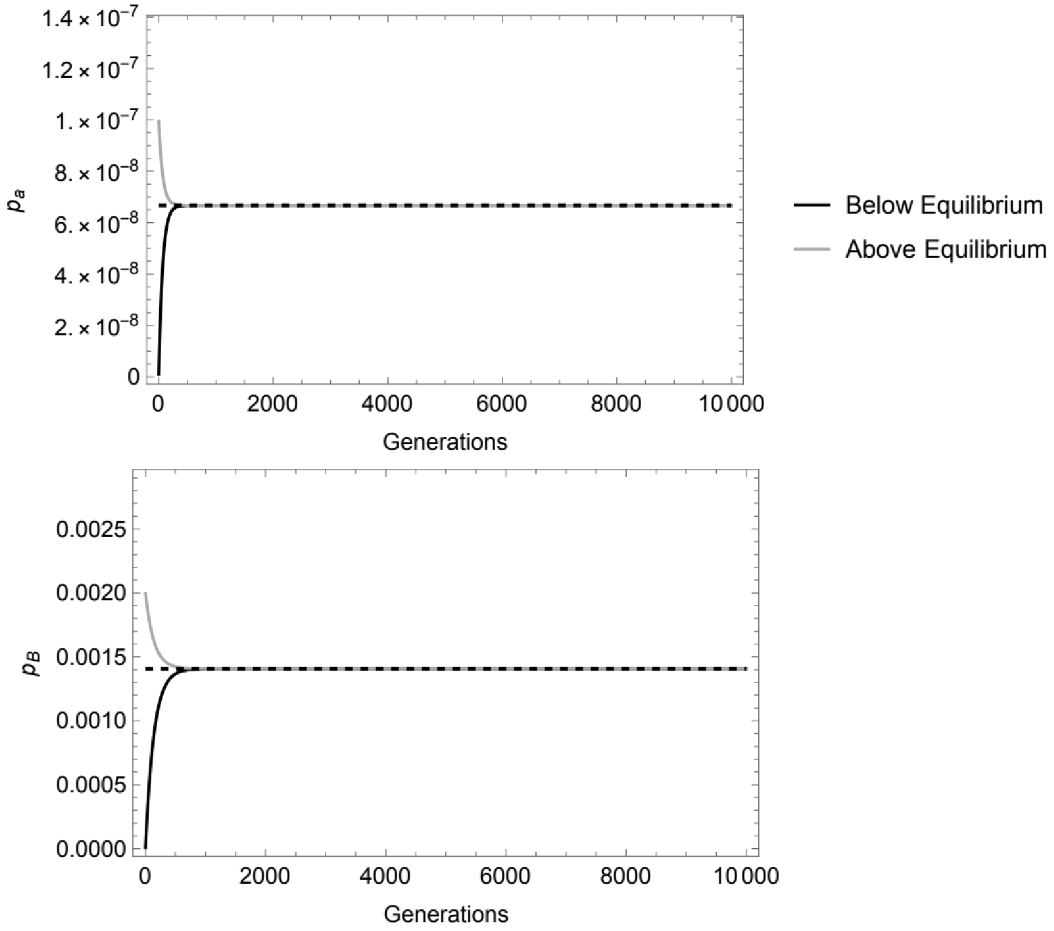


Figure D2: Plots of the deleterious allele frequency (top) and the epiallele frequency (bottom) across time in generations. The

numerical parameter values are

$f = 0.5$, $s = 0.02$, $s_2 = 0.01$, $s_3 = 0.001$, $h = 0.5$, $h_2 = 0.5$, $t_1 = 10^{\wedge}-5$, $t_2 = 10^{\wedge}-4$, $u = 10^{\wedge}-9$, $c = 10^{\wedge}-9$, $z = 10^{\wedge}-9$, $m \to 0.001$

The approximated eigenvalues corresponding with eq. 9 of the main text are

$$\begin{gathered}
\lambda_{(1)} \sim \frac{-1+m-fm+(-1+f)(-1+m)h_2 s_2}{-1+s_2} + \\
\Big((-1+f)m\big(-cm+cfm-cfs-chs+cfhs+cfms-cf^2ms+2chms-4cfhms+2cf^2hms+fsu+hsu-fhsu-fmsu+ \\
f^2msu-hmsu+2fhmsu-f^2hmsu-(f(-1+h)-h)(1+(-1+f)m)st_1-fst_2-hst_2+fhst_2-fmst_2+ \\
f^2mst_2-hmst_2+2fhmst_2-f^2hmst_2-(-1+f)s_3((1+(-1+f)m)(2c-u)+(-1+m-fm)t_1+(1+m-fm)t_2)\big)+ \\
(-1+f)(-1+m)h_2 s_2^3\big(-c+cf+u+t_1-t_2+2ft_2+4mt_2-4fmt_2+(-1+f)^2(-1+m)h_2^2(2c-u-t_1+3t_2)- \\
(-1+f)h_2(3c-cf-cm+cfm-2u+mu+(-2+m)t_1+(4-2f-7m+4fm)t_2)\big)- \\
s_2\big(3cm-4cfm+cf^2m-cm^2+3cfm^2-3cf^2m^2+cf^3m^2+cfs+chs-cfhs-cfms+cf^2ms-3chms+ \\
6cfhms-3cf^2hms-mu+fmu+m^2u-2fm^2u+f^2m^2u-fsu-hsu+fhsu+fmsu-f^2msu+ \\
hmsu-2fhmsu+f^2hmsu-mt_1+fmt_1+m^2t_1-2fm^2t_1+f^2m^2t_1-fst_1-hst_1+fhst_1+fmst_1- \\
f^2mst_1+hmst_1-2fhmst_1+f^2hmst_1+mt_2-fmt_2+m^2t_2-2fm^2t_2+f^2m^2t_2+3fst_2-2f^2st_2+ \\
3hst_2-5fhst_2+2f^2hst_2-5fmst_2+7f^2mst_2-2f^3mst_2-5hmst_2+12fhmst_2-9f^2hmst_2+ \\
2f^3hmst_2+4fm^2st_2-8f^2m^2st_2+4f^3m^2st_2+4hm^2st_2-12fhm^2st_2+12f^2hm^2st_2-4f^3hm^2st_2+ \\
(-1+f)s_3\big((1+(-1+f)m)(2c-u)+(-1+m-fm)t_1+\big(3-5m+4m^2+2f^2m(-1+2m)+f\big(-2+7m-8m^2\big)\big)t_2\big)+ \\
(-1+f)h_2\big(4cm-4cfm-4cm^2+6cfm^2-2cf^2m^2+cfs+chs-cfhs-3cfms+2cf^2ms- \\
5chms+9cfhms-4cf^2hms+2cfm^2s-2cf^2m^2s+4chm^2s-8cfhm^2s+4cf^2hm^2s- \\
mu+fmu+m^2u-2fm^2u+f^2m^2u-fsu-hsu+fhsu+3fmsu-2f^2msu+3hmsu- \\
5fhmsu+2f^2hmsu-2fm^2su+2f^2m^2su-2hm^2su+4fhm^2su-2f^2hm^2su+ \\
\big((f(-1+h)-h)s+m\big(-1+f+f(3-5h)s+2f^2(-1+h)s+3hs\big)-(-1+f)m^2(1-2hs+f(-1+2(-1+h)s))\big)t_1+ \\
mt_2-fmt_2+m^2t_2-2fm^2t_2+f^2m^2t_2+5fst_2+5hst_2-5fhst_2-7fmst_2+2f^2mst_2-7hmst_2+ \\
9fhmst_2-2f^2hmst_2+2fm^2st_2-2f^2m^2st_2+2hm^2st_2-4fhm^2st_2+2f^2hm^2st_2- \\
(-1+f)(-1+m)s_3((1+2(-1+f)m)(2c-u)+(-1-2(-1+f)m)t_1+(5+2(-1+f)m)t_2)\big)\big)+ \\
s_2^2\big(2c-cf-cm+2cfm-cf^2m-chs+cfhs-u+mu-fmu-t_1+mt_1-fmt_1+3t_2-2ft_2- \\
5mt_2+7fmt_2-2f^2mt_2+4m^2t_2-8fm^2t_2+4f^2m^2t_2+ \\
(-1+f)^2(-1+m)h_2^2(-3c+5cm-4cfm+cfs+2chs-2cfhs-cfms-2chms+2cfhms+u-2mu+2fmu- \\
fsu-hsu+fhsu+fmsu+hmsu-fhmsu+(1+f(-1+h)s-hs+m(-2+hs+f(2+s-hs)))t_1-5t_2+2mt_2- \\
2fmt_2+3fst_2+3hst_2-3fhst_2-3fmst_2-3hmst_2+3fhmst_2-(-1+f)(-1+m)s_3(2c-u-t_1+3t_2))+ \\
(-1+f)h_2\big(5c-cf-7cm+7cfm-2cf^2m+2cm^2-4cfm^2+2cf^2m^2-cfs-3chs+3cfhs+ \\
cfms+3chms-3cfhms-2u+4mu-3fmu-2m^2u+2fm^2u+fsu+hsu-fhsu-fmsu- \\
hmsu+fhmsu+\big(-2+2(-1+f)m^2+hs+f(s-hs)+m(4-hs+f(-3+(-1+h)s))\big)t_1+8t_2- \\
2ft_2-12mt_2+9fmt_2-2f^2mt_2+6m^2t_2-10fm^2t_2+4f^2m^2t_2-fst_2+2f^2st_2-hst_2+3fhst_2- \\
2f^2hst_2+5fmst_2-6f^2mst_2+5hmst_2-11fhmst_2+6f^2hmst_2-4fm^2st_2+4f^2m^2st_2- \\
4hm^2st_2+8fhm^2st_2-4f^2hm^2st_2+(-1+f)(-1+m)s_3(2c-u-t_1+(1-2f-4m+4fm)t_2)\big)\big)\Big)\Big/ \\
((-1+s_2)(m-fm+(-1+(-1+f)(-1+m)h_2)s_2)(f(-1+h)s-hs+s_2+(-1+f)h_2s_2-(-1+f)s_3))+ \\
O[\zeta^2]
\end{gathered}$$

$$\begin{gathered}
\lambda_{(2)} \sim \frac{-1+fs+hs-fhs-(-1+f)h_2s_2+(-1+f)s_3}{-1+s_2} + \\
-\Big(\Big((-1+f)h_2 s_2^3\big(-c+2cf+z-t_2+ft_2+2mt_2-2fmt_2+(-1+f)^2(-1+m)h_2^2(3c-z+2t_2)- \\
(-1+f)h_2(c(4+2f(-1+m)-m)+(-2+m)z+(3-4m+f(-1+2m))t_2)\big)-
\end{gathered}$$

$$
\begin{array}{c}
(-1+f)\,(m\,s\,(c\,(2-5\,h+3\,h^2\,s+f^2\,(1-4\,h+3\,h^2)\,s+f\,(-3+5\,h+4\,h\,s-6\,h^2\,s))-(f\,(-1+h)-h)\,(1+f\,(-1+h)\,s-h\,s)\,z)+ \\
(-1+f)\,s_3^2\,((-1+f)\,m\,(3\,c-z)+t_2)+ \\
s_3\,(-((-1+f)\,m\,(c\,(5-6\,h\,s+2\,f\,(-2+3\,h)\,s)+(-1-2\,f\,(-1+h)\,s+2\,h\,s)\,z))+(f+h-f\,h)\,s\,t_2))+ \\
s_2^2\,(-3\,c+2\,c\,f+2\,c\,s+c\,f\,s-2\,c\,f^2\,s+c\,h\,s-3\,c\,f\,h\,s+2\,c\,f^2\,h\,s+z-f\,s\,z-h\,s\,z+f\,h\,s\,z-c\,s_3+3\,c\,f\,s_3- \\
2\,c\,f^2\,s_3+z\,s_3-f\,z\,s_3-2\,t_2+f\,t_2+2\,m\,t_2-2\,f\,m\,t_2+2\,f\,s\,t_2-f^2\,s\,t_2+2\,h\,s\,t_2-3\,f\,h\,s\,t_2+f^2\,h\,s\,t_2- \\
2\,f\,m\,s\,t_2+2\,f^2\,m\,s\,t_2-2\,h\,m\,s\,t_2+4\,f\,h\,m\,s\,t_2-2\,f^2\,h\,m\,s\,t_2-s_3\,t_2+2\,f\,s_3\,t_2-f^2\,s_3\,t_2+2\,m\,s_3\,t_2- \\
4\,f\,m\,s_3\,t_2+2\,f^2\,m\,s_3\,t_2+(-1+f)^2\,h_2^2\,(-5\,c+8\,c\,m-3\,c\,f\,m+4\,c\,f\,s+6\,c\,h\,s-6\,c\,f\,h\,s-4\,c\,f\,m\,s- \\
6\,c\,h\,m\,s+6\,c\,f\,h\,m\,s+z-2\,m\,z+f\,m\,z-2\,f\,s\,z-2\,h\,s\,z+2\,f\,h\,s\,z+2\,f\,m\,s\,z+2\,h\,m\,s\,z-2\,f\,h\,m\,s\,z+ \\
(-3-4\,f\,(-1+h)\,s+4\,h\,s+m\,(2+4\,f\,(-1+h)\,s-4\,h\,s))\,t_2-2\,(-1+f)\,(-1+m)\,s_3\,(3\,c-z+2\,t_2))- \\
(-1+f)\,h_2\,(8\,c-2\,c\,f-4\,c\,m+5\,c\,f\,m-2\,c\,f^2\,m-2\,c\,s-5\,c\,f\,s+2\,c\,f^2\,s-7\,c\,h\,s+9\,c\,f\,h\,s-2\,c\,f^2\,h\,s+2\,c\,m\,s+c\,f\,m\,s- \\
2\,c\,f^2\,m\,s+c\,h\,m\,s-3\,c\,f\,h\,m\,s+2\,c\,f^2\,h\,m\,s-2\,z+2\,m\,z-f\,m\,z+3\,f\,s\,z+3\,h\,s\,z-3\,f\,h\,s\,z-f\,m\,s\,z-h\,m\,s\,z+ \\
f\,h\,m\,s\,z+(5-5\,h\,s+2\,f^2\,(-1+h)\,(-1+2\,m)\,s+m\,(-4+6\,h\,s)+f\,(-1-5\,s+7\,h\,s+m\,(2+6\,s-10\,h\,s)))\,t_2- \\
(-1+f)\,s_3\,(c\,(7+2\,f\,(-1+m)-m)+(-3+m)\,z+(4-6\,m+f\,(-2+4\,m))\,t_2)))+ \\
s_2\,(3\,c\,m-5\,c\,f\,m+2\,c\,f^2\,m-2\,c\,s+3\,c\,f\,s+5\,c\,h\,s-5\,c\,f\,h\,s-2\,c\,m\,s+c\,f\,m\,s+3\,c\,f^2\,m\,s-2\,c\,f^3\,m\,s-c\,h\,m\,s+ \\
4\,c\,f\,h\,m\,s-5\,c\,f^2\,h\,m\,s+2\,c\,f^3\,h\,m\,s-c\,f^2\,s^2-4\,c\,f\,h\,s^2+4\,c\,f^2\,h\,s^2-3\,c\,h^2\,s^2+6\,c\,f\,h^2\,s^2-3\,c\,f^2\,h^2\,s^2-m\,z+ \\
f\,m\,z-f\,s\,z-h\,s\,z+f\,h\,s\,z+f\,m\,s\,z-f^2\,m\,s\,z+h\,m\,s\,z-2\,f\,h\,m\,s\,z+f^2\,h\,m\,s\,z+f^2\,s^2\,z+2\,f\,h\,s^2\,z-2\,f^2\,h\,s^2\,z+ \\
h^2\,s^2\,z-2\,f\,h^2\,s^2\,z+f^2\,h^2\,s^2\,z-5\,c\,s_3+5\,c\,f\,s_3+c\,m\,s_3-4\,c\,f\,m\,s_3+5\,c\,f^2\,m\,s_3-2\,c\,f^3\,m\,s_3+4\,c\,f\,s\,s_3- \\
4\,c\,f^2\,s\,s_3+6\,c\,h\,s\,s_3-12\,c\,f\,h\,s\,s_3+6\,c\,f^2\,h\,s\,s_3+z\,s_3-f\,z\,s_3-m\,z\,s_3+2\,f\,m\,z\,s_3-f^2\,m\,z\,s_3-2\,f\,s\,z\,s_3+ \\
2\,f^2\,s\,z\,s_3-2\,h\,s\,z\,s_3+4\,f\,h\,s\,z\,s_3-2\,f^2\,h\,s\,z\,s_3-3\,c\,s_3^2+6\,c\,f\,s_3^2-3\,c\,f^2\,s_3^2+z\,s_3^2-2\,f\,z\,s_3^2+f^2\,z\,s_3^2+2\,f\,s\,t_2-f^2\,s\,t_2+ \\
2\,h\,s\,t_2-3\,f\,h\,s\,t_2+f^2\,h\,s\,t_2-2\,f\,m\,s\,t_2+2\,f^2\,m\,s\,t_2-2\,h\,m\,s\,t_2+4\,f\,h\,m\,s\,t_2-2\,f^2\,h\,m\,s\,t_2-2\,f^2\,s^2\,t_2+f^3\,s^2\,t_2- \\
4\,f\,h\,s^2\,t_2+6\,f^2\,h\,s^2\,t_2-2\,f^3\,h\,s^2\,t_2-2\,h^2\,s^2\,t_2+5\,f\,h^2\,s^2\,t_2-4\,f^2\,h^2\,s^2\,t_2+f^3\,h^2\,s^2\,t_2+2\,f^2\,m\,s^2\,t_2-2\,f^3\,m\,s^2\,t_2+ \\
4\,f\,h\,m\,s^2\,t_2-8\,f^2\,h\,m\,s^2\,t_2+4\,f^3\,h\,m\,s^2\,t_2+2\,h^2\,m\,s^2\,t_2-6\,f\,h^2\,m\,s^2\,t_2+6\,f^2\,h^2\,m\,s^2\,t_2-2\,f^3\,h^2\,m\,s^2\,t_2- \\
3\,s_3\,t_2+4\,f\,s_3\,t_2-f^2\,s_3\,t_2+2\,m\,s_3\,t_2-4\,f\,m\,s_3\,t_2+2\,f^2\,m\,s_3\,t_2+3\,f\,s\,s_3\,t_2-5\,f^2\,s\,s_3\,t_2+2\,f^3\,s\,s_3\,t_2+3\,h\,s\,s_3\,t_2- \\
8\,f\,h\,s\,s_3\,t_2+7\,f^2\,h\,s\,s_3\,t_2-2\,f^3\,h\,s\,s_3\,t_2-4\,f\,m\,s\,s_3\,t_2+8\,f^2\,m\,s\,s_3\,t_2-4\,f^3\,m\,s\,s_3\,t_2-4\,h\,m\,s\,s_3\,t_2+12\,f\,h\,m\,s\,s_3\,t_2- \\
12\,f^2\,h\,m\,s\,s_3\,t_2+4\,f^3\,h\,m\,s\,s_3\,t_2-s_3^2\,t_2+3\,f\,s_3^2\,t_2-3\,f^2\,s_3^2\,t_2+f^3\,s_3^2\,t_2+2\,m\,s_3^2\,t_2-6\,f\,m\,s_3^2\,t_2+6\,f^2\,m\,s_3^2\,t_2- \\
2\,f^3\,m\,s_3^2\,t_2+(-1+f)\,h_2\,(5\,c\,m-5\,c\,f\,m-2\,c\,s+3\,c\,f\,s+5\,c\,h\,s-5\,c\,f\,h\,s+2\,c\,m\,s-7\,c\,f\,m\,s+4\,c\,f^2\,m\,s-11\,c\,h\,m\,s+ \\
17\,c\,f\,h\,m\,s-6\,c\,f^2\,h\,m\,s-c\,f^2\,s^2-4\,c\,f\,h\,s^2+4\,c\,f^2\,h\,s^2-3\,c\,h^2\,s^2+6\,c\,f\,h^2\,s^2-3\,c\,f^2\,h^2\,s^2+c\,f^2\,m\,s^2+ \\
4\,c\,f\,h\,m\,s^2-4\,c\,f^2\,h\,m\,s^2+3\,c\,h^2\,m\,s^2-6\,c\,f\,h^2\,m\,s^2+3\,c\,f^2\,h^2\,m\,s^2-m\,z+f\,m\,z-f\,s\,z-h\,s\,z+f\,h\,s\,z+ \\
3\,f\,m\,s\,z-2\,f^2\,m\,s\,z+3\,h\,m\,s\,z-5\,f\,h\,m\,s\,z+2\,f^2\,h\,m\,s\,z+f^2\,s^2\,z+2\,f\,h\,s^2\,z-2\,f^2\,h\,s^2\,z+h^2\,s^2\,z- \\
2\,f\,h^2\,s^2\,z+f^2\,h^2\,s^2\,z-f^2\,m\,s^2\,z-2\,f\,h\,m\,s^2\,z+2\,f^2\,h\,m\,s^2\,z-h^2\,m\,s^2\,z+2\,f\,h^2\,m\,s^2\,z-f^2\,h^2\,m\,s^2\,z+ \\
(f\,(-1+h)-h)\,s\,(-3-2\,f\,(-1+h)\,s+2\,h\,s+2\,m\,(1+f\,(-1+h)\,s-h\,s))\,t_2+(-1+f)^2\,(-1+m)\,s_3^2\,(3\,c-z+2\,t_2)- \\
(-1+f)\,s_3\,(c\,(-5+4\,f\,s+6\,h\,s-6\,f\,h\,s+m\,(11-6\,h\,s+f\,(-6-4\,s+6\,h\,s)))+(1+2\,f\,(-1+h)\,s-2\,h\,s+m\,(-3+2\,h \\
s+f\,(2+2\,s-2\,h\,s)))\,z+2\,(-2+m-2\,f\,(-1+h)\,s+2\,h\,s+2\,f\,(-1+h)\,m\,s-2\,h\,m\,s)\,t_2))))\,/ \\
((-1+s_2)\,(m-f\,m+(-1+(-1+f)\,(-1+m)\,h_2)\,s_2)\,(f\,(-1+h)\,s-h\,s+s_2+(-1+f)\,h_2\,s_2-(-1+f)\,s_3))) \\
+O[\zeta^2]
\end{array}
$$

Numerically evaluating the approximated eigenvalues for local stability such that $|\lambda_{(1)}| < 1$ and $|\lambda_{(2)}| < 1$ (Tables D22-D24):

Table D22: Numerically evaluating the approximated eigenvalues to determine local stability as the paramutation rate varies across a given range

| m | $\sim\lambda_{(1)}$ | $\sim\lambda_{(2)}$ | Local Stability |
|---|---|---|---|
| 0.01 | 0.996608 | 0.995172 | Stable |
| 0.06 | 0.951382 | 0.995341 | Stable |
| 0.11 | 0.90616 | 0.995348 | Stable |
| 0.16 | 0.860937 | 0.99535 | Stable |
| 0.21 | 0.815715 | 0.995351 | Stable |
| 0.26 | 0.770493 | 0.995352 | Stable |
| 0.31 | 0.72527 | 0.995352 | Stable |
| 0.36 | 0.680048 | 0.995353 | Stable |
| 0.41 | 0.634825 | 0.995353 | Stable |
| 0.46 | 0.589603 | 0.995353 | Stable |
| 0.51 | 0.544381 | 0.995353 | Stable |
| 0.56 | 0.499158 | 0.995353 | Stable |
| 0.61 | 0.453936 | 0.995353 | Stable |
| 0.66 | 0.408714 | 0.995353 | Stable |
| 0.71 | 0.363491 | 0.995354 | Stable |
| 0.76 | 0.318269 | 0.995354 | Stable |
| 0.81 | 0.273046 | 0.995354 | Stable |
| 0.86 | 0.227824 | 0.995354 | Stable |
| 0.91 | 0.182602 | 0.995354 | Stable |

Parameter values : $f = 0.1$, $s = 0.02$, $s_2 = 0.01$, $s_3 = 0.001$, $h = 0.5$,
$h_2 = 0.5$, $t_1 = 10^{-5}$, $t_2 = 10^{-4}$, $u = 10^{-9}$, $c = 10^{-9}$, $z = 10^{-9}$

Table D23: Numerically evaluating the approximated eigenvalues to determine local stability as the forward spontaneous epimutation rate varies across a given range

| $t_1$ | $\sim\lambda_{(1)}$ | $\sim\lambda_{(2)}$ | Local Stability |
|---|---|---|---|
| 0.00001 | 0.915204 | 0.995347 | Stable |
| 0.00011 | 0.915113 | 0.995347 | Stable |
| 0.00021 | 0.915021 | 0.995347 | Stable |
| 0.00031 | 0.91493 | 0.995347 | Stable |
| 0.00041 | 0.914838 | 0.995347 | Stable |
| 0.00051 | 0.914747 | 0.995347 | Stable |
| 0.00061 | 0.914655 | 0.995347 | Stable |
| 0.00071 | 0.914564 | 0.995347 | Stable |
| 0.00081 | 0.914472 | 0.995347 | Stable |
| 0.00091 | 0.914381 | 0.995347 | Stable |
| 0.00101 | 0.914289 | 0.995347 | Stable |

Parameter values: $f = 0.1$, $m = 0.1$, $s = 0.02$, $s_2 = 0.01$, $s_3 = 0.001$, $h = 0.5$,
$h_2 = 0.5$, $u = 10^{-9}$, $t_2 = 10^{-4}$, $u = 10^{-9}$, $c = 10^{-9}$, $z = 10^{-9}$

Table D24: Numerically evaluating the approximated eigenvalues to determine local stability as the inbreeding coefficient varies across a given range

| f | $\sim\lambda_{(1)}$ | $\sim\lambda_{(2)}$ | Local Stability |
|---|---|---|---|
| 0.01 | 0.905705 | 0.995894 | Stable |
| 0.03 | 0.907816 | 0.995772 | Stable |
| 0.05 | 0.909927 | 0.995651 | Stable |
| 0.07 | 0.912038 | 0.995529 | Stable |
| 0.09 | 0.914149 | 0.995408 | Stable |
| 0.11 | 0.91626 | 0.995286 | Stable |
| 0.13 | 0.918371 | 0.995165 | Stable |
| 0.15 | 0.920482 | 0.995043 | Stable |
| 0.17 | 0.922592 | 0.994922 | Stable |
| 0.19 | 0.924703 | 0.9948 | Stable |
| 0.21 | 0.926814 | 0.994679 | Stable |
| 0.23 | 0.928925 | 0.994557 | Stable |
| 0.25 | 0.931036 | 0.994435 | Stable |
| 0.27 | 0.933147 | 0.994314 | Stable |
| 0.29 | 0.935258 | 0.994192 | Stable |

Parameter values: $m = 0.1$, $s = 0.02$, $s_2 = 0.01$, $s_3 = 0.001$, $h = 0.5$, $h_2 = 0.5$,
$u = 10^{-9}$, $t_1 = 10^{-5}$, $t_2 = 10^{-4}$, $u = 10^{-9}$, $c = 10^{-9}$, $z = 10^{-9}$

Evaluation of local stability through numerical simulations of the recursion equations (Figure D3). Evidence for local stability was supported through simulations of the recursion equations beginning above (gray line) and below (black line) the equilibrium (dashed line).

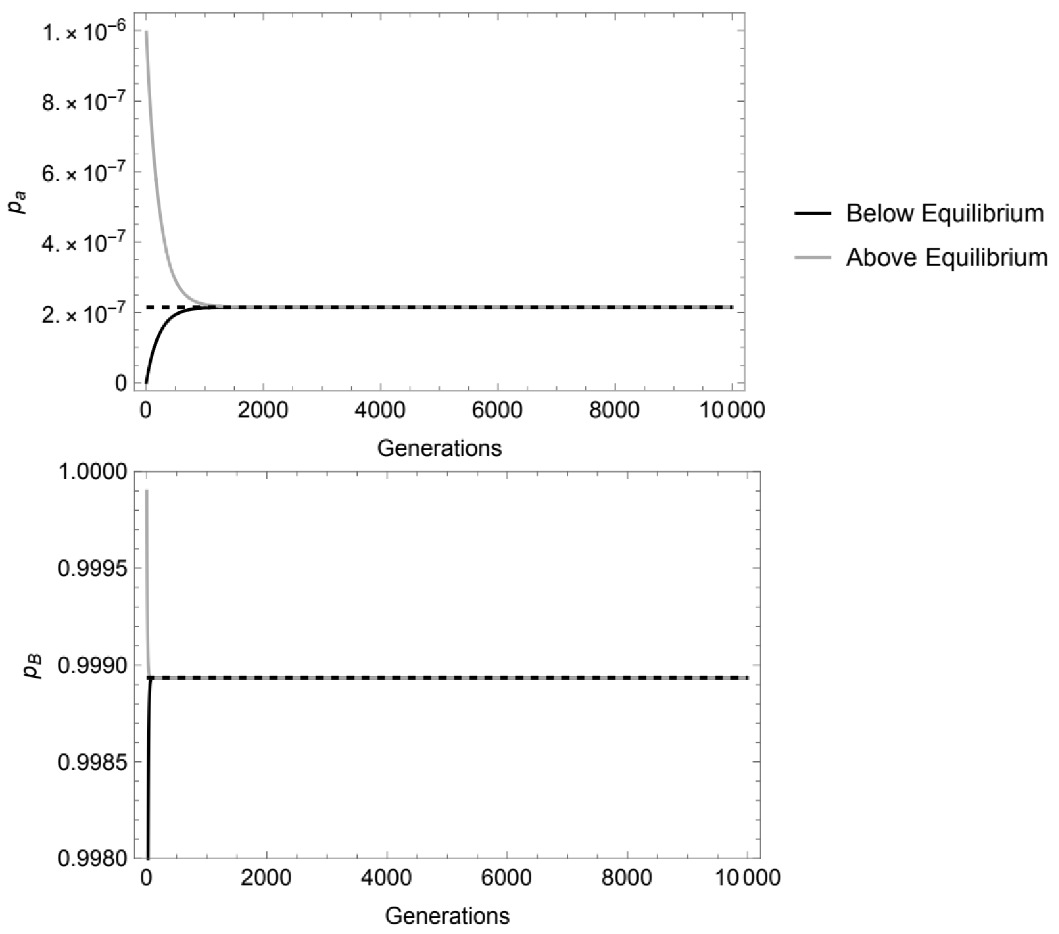


Figure D3: Plots of the deleterious allele frequency (top) and the epiallele frequency (bottom) across time in generations. The numerical parameter values are

$f = 0.1, s = 0.02, s_2 = 0.01, s_3 = 0.001, h = 0.5, h_2 = 0.5, t_1 = 10^{-5}, t_2 = 10^{-4}, u = 10^{-9}, c = 10^{-9}, z = 10^{-9}, m \to 0.11$

The approximated eigenvalues corresponding with eq. 10 of the main text are

$$\lambda_{(1)} \sim 1 - f s - h s + f h s + \Big((-1+f)(f(-1+h)-h) s_3 t_1 + s_2 \Big(-((-2m + f(-1+2m))\Big(-((f(-1+h)-h)(1+f(-1+h)s - h s) t_1) + \Big(\big(2 - 5h + 3h^2 s + f^2 (1 - 4h + 3h^2) s + f\big(-3 + 5h + 4hs - 6h^2 s\big)\big) u - (f(-1+h)-h)(1 + f(-1+h)s - hs) z\Big)(-1+t_2)\Big)\Big) + (-1+f) h_2 \Big(-((f(-1+h)-h)(-1 - 2f(-1+h)s + 2hs + 2m(1 + f(-1+h)s - hs)) t_1) + (-1+m)\Big(\big(2 - 5h + 3h^2 s + f^2(1 - 4h + 3h^2) s + f\big(-3 + 5h + 4hs - 6h^2 s\big)\big) u - (f(-1+h)-h)(1 + f(-1+h)s - hs) z\Big)(-1+t_2)\Big)\Big) + \Big(\big(2 - 5h + 3h^2 s + f^2(1 - 4h + 3h^2) s + f\big(-3 + 5h + 4hs - 6h^2 s\big)\big) u - (f(-1+h)-h)(1 + f(-1+h)s - hs) z\Big) (m - f m + (-1 + (-1+f) m) t_2)\Big) \Big/ ((f(-1+h)-h)(m - f m + (f + 2m - 2fm + (-1+f)(-1+m) h_2) s_2 (-1+t_2) + (-1 + (-1+f) m) t_2)) + O[\zeta^2]$$

$$\lambda_{(2)} \sim (-1 - m + f m + f s_2 + 2 m s_2 - 2 f m s_2 + h_2 s_2 - f h_2 s_2 - m h_2 s_2 + f m h_2 s_2)(-1 + t_2) -$$

$$
\Bigg(\Bigg(2(-1+f)\,t_1\,(f-f\,s+(-1+f)(-1+h\,s)-(-1+(-1+f)\,m+(f+2\,m-2\,f\,m+(-1+f)(-1+m)\,h_2)\,s_2)(-1+t_2))-
f\,t_1\,(f-f\,s+(-1+f)(-1+h\,s)-(-1+(-1+f)\,m+(f+2\,m-2\,f\,m+(-1+f)(-1+m)\,h_2)\,s_2)(-1+t_2))+
(-1+f)(-1+m)(1-h_2\,s_2)\,t_1\,(f-f\,s+(-1+f)(-1+h\,s)-(-1+(-1+f)\,m+(f+2\,m-2\,f\,m+(-1+f)(-1+m)\,h_2)\,s_2)(-1+t_2))+
c\,(1+m-f\,m-(f+2\,m-2\,f\,m+(-1+f)(-1+m)\,h_2)\,s_2)
(f-f\,s+(-1+f)(-1+h\,s)-(-1+(-1+f)\,m+(f+2\,m-2\,f\,m+(-1+f)(-1+m)\,h_2)\,s_2)(-1+t_2))(-1+t_2)+(-1+f)
(1+m+(-2\,m+(-1+m)\,h_2)\,s_2)\left(-\frac{u}{(f+h-f\,h)\,s}+\frac{t_1}{-1+f(-1+s_2)(-1+t_2)+(-1+f)(1+m+(-2\,m+(-1+m)\,h_2)\,s_2)(-1+t_2)}\right)
(f-f\,s+(-1+f)(-1+h\,s)-(-1+(-1+f)\,m+(f+2\,m-2\,f\,m+(-1+f)(-1+m)\,h_2)\,s_2)(-1+t_2))(-1+t_2)-
(-1+f)\left(\frac{u\,(1-h\,s-h_2\,s_2+s_3)}{(f(-1+h)-h)\,s}-\frac{(-1+s_2)\,t_1}{-1+f(-1+s_2)(-1+t_2)+(-1+f)(1+m+(-2\,m+(-1+m)\,h_2)\,s_2)(-1+t_2)}\right)
(f-f\,s+(-1+f)(-1+h\,s)-(-1+(-1+f)\,m+(f+2\,m-2\,f\,m+(-1+f)(-1+m)\,h_2)\,s_2)(-1+t_2))(-1+t_2)+
((f+2\,m-2\,f\,m+2(-1+f)(-1+m)\,h_2)\,s_2\,t_1\,(m-f\,m+f\,s+h\,s-f\,h\,s+(f+2\,m-2\,f\,m+(-1+f)(-1+m)\,h_2)\,s_2\,(-1+t_2)+
(-1+(-1+f)\,m)\,t_2))/(m-f\,m+(f+2\,m-2\,f\,m+(-1+f)(-1+m)\,h_2)\,s_2\,(-1+t_2)+(-1+(-1+f)\,m)\,t_2)-
((-1+f)(-1+s_2)\,t_1\,(-1+t_2)(m-f\,m+f\,s+h\,s-f\,h\,s+(f+2\,m-2\,f\,m+(-1+f)(-1+m)\,h_2)\,s_2\,(-1+t_2)+(-1+(-1+f)\,m)\,t_2))/
(m-f\,m+(f+2\,m-2\,f\,m+(-1+f)(-1+m)\,h_2)\,s_2\,(-1+t_2)+(-1+(-1+f)\,m)\,t_2)-((-1+f)(1+m+(-2\,m+(-1+m)\,h_2)\,s_2)
t_1\,(-1+t_2)(m-f\,m+f\,s+h\,s-f\,h\,s+(f+2\,m-2\,f\,m+(-1+f)(-1+m)\,h_2)\,s_2\,(-1+t_2)+(-1+(-1+f)\,m)\,t_2))/
(m-f\,m+(f+2\,m-2\,f\,m+(-1+f)(-1+m)\,h_2)\,s_2\,(-1+t_2)+(-1+(-1+f)\,m)\,t_2)+
((f(-1+s_2)+(-1+f)(1+m+(-2\,m+(-1+m)\,h_2)\,s_2))
(f-f\,s+(-1+f)(-1+h\,s)-(-1+(-1+f)\,m+(f+2\,m-2\,f\,m+(-1+f)(-1+m)\,h_2)\,s_2)(-1+t_2))(-1+t_2)
(s_2\,(-((-2\,m+f(-1+2\,m))((f+h-f\,h)\,t_1+(-2\,h+f(-1+2\,h))\,u\,(-1+t_2)))+(-1+f)(-1+m)\,h_2
(2(f+h-f\,h)\,t_1+(-2\,h+f(-1+2\,h))\,u\,(-1+t_2)))+(-2\,h+f(-1+2\,h))\,u\,(m-f\,m+(-1+(-1+f)\,m)\,t_2)))/
((f(-1+h)-h)(m-f\,m+(f+2\,m-2\,f\,m+(-1+f)(-1+m)\,h_2)\,s_2\,(-1+t_2)+(-1+(-1+f)\,m)\,t_2))\Bigg)\Big/
(m-f\,m+f\,s+h\,s-f\,h\,s+(f+2\,m-2\,f\,m+(-1+f)(-1+m)\,h_2)\,s_2\,(-1+t_2)+(-1+(-1+f)\,m)\,t_2)\Bigg)+O[\zeta^2]
$$

Numerically evaluating the approximated eigenvalues for local stability such that $|\lambda_{(1)}| < 1$ and $|\lambda_{(2)}| < 1$ (Tables D25-D27):

Table D25: Numerically evaluating the approximated eigenvalues to determine local stability as the paramutation rate varies across a given range

| m | $\sim\lambda_{(1)}$ | $\sim\lambda_{(2)}$ | Local Stability |
|---|---|---|---|
| 0.005 | 0.989 | 0.799136 | Stable |
| 0.025 | 0.989 | 0.813318 | Stable |
| 0.045 | 0.989 | 0.827501 | Stable |
| 0.065 | 0.989 | 0.841682 | Stable |
| 0.085 | 0.989 | 0.855864 | Stable |
| 0.105 | 0.989001 | 0.870045 | Stable |
| 0.125 | 0.989001 | 0.884225 | Stable |
| 0.145 | 0.989001 | 0.898404 | Stable |
| 0.165 | 0.989001 | 0.912581 | Stable |
| 0.185 | 0.989001 | 0.926757 | Stable |
| 0.205 | 0.989001 | 0.940928 | Stable |

Parameter values: $f = 0.1$, $s = 0.02$, $s_2 = 0.01$, $s_3 = 0.001$,
$h = 0.5$, $h_2 = 0.5$, $u = 10^{-9}$, $t_1 = 10^{-5}$, $t_2 = 0.2$, $u = 10^{-9}$,
$c = 10^{-9}$, $z = 10^{-9}$

Table D26: Numerically evaluating the approximated eigenvalues to determine local stability as the forward spontaneous epimutation rate varies across a given range

| $t_1$ | $\sim\lambda_{(1)}$ | $\sim\lambda_{(2)}$ | Local Stability |
|---|---|---|---|
| 0.00001 | 0.989001 | 0.866499 | Stable |
| 0.00011 | 0.989006 | 0.866294 | Stable |
| 0.00021 | 0.989011 | 0.866088 | Stable |
| 0.00031 | 0.989017 | 0.865882 | Stable |
| 0.00041 | 0.989022 | 0.865676 | Stable |
| 0.00051 | 0.989027 | 0.86547 | Stable |
| 0.00061 | 0.989033 | 0.865264 | Stable |
| 0.00071 | 0.989038 | 0.865059 | Stable |
| 0.00081 | 0.989044 | 0.864853 | Stable |
| 0.00091 | 0.989049 | 0.864647 | Stable |
| 0.00101 | 0.989054 | 0.864441 | Stable |

Parameter values: m = 0.1, f = 0.1, s = 0.02, $s_2$ = 0.01,
$s_3$ = 0.001, h = 0.5, $h_2$ = 0.5, u = 10 ^ −9,
$t_2$ = 0.2, u = 10 ^ −9, c = 10 ^ −9, z = 10 ^ −9

Table D27: Numerically evaluating the approximated eigenvalues to determine local stability as the inbreeding coefficient varies across a given range

| f | $\sim\lambda_{(1)}$ | $\sim\lambda_{(2)}$ | Local Stability |
|---|---|---|---|
| 0.01 | 0.989901 | 0.87395 | Stable |
| 0.06 | 0.989401 | 0.869811 | Stable |
| 0.11 | 0.988901 | 0.865672 | Stable |
| 0.16 | 0.988401 | 0.861533 | Stable |
| 0.21 | 0.987901 | 0.857393 | Stable |
| 0.26 | 0.987401 | 0.853254 | Stable |
| 0.31 | 0.986901 | 0.849115 | Stable |
| 0.36 | 0.986401 | 0.844976 | Stable |
| 0.41 | 0.985901 | 0.840836 | Stable |
| 0.46 | 0.985401 | 0.836697 | Stable |

Parameter values : m = 0.1, s = 0.02, $s_2$ = 0.01, $s_3$ = 0.001,
h = 0.5, $h_2$ = 0.5, u = 10 ^ −9, $t_1$ = 10 ^ −5, $t_2$ = 0.2, u = 10 ^ −9,
c = 10 ^ −9, z = 10 ^ −9

Evaluation of local stability through numerical simulations of the recursion equations (Figure D4). Evidence for local stability was supported through simulations of the recursion equations beginning above (dotted line) and below (solid line) the equilibrium (dashed line).

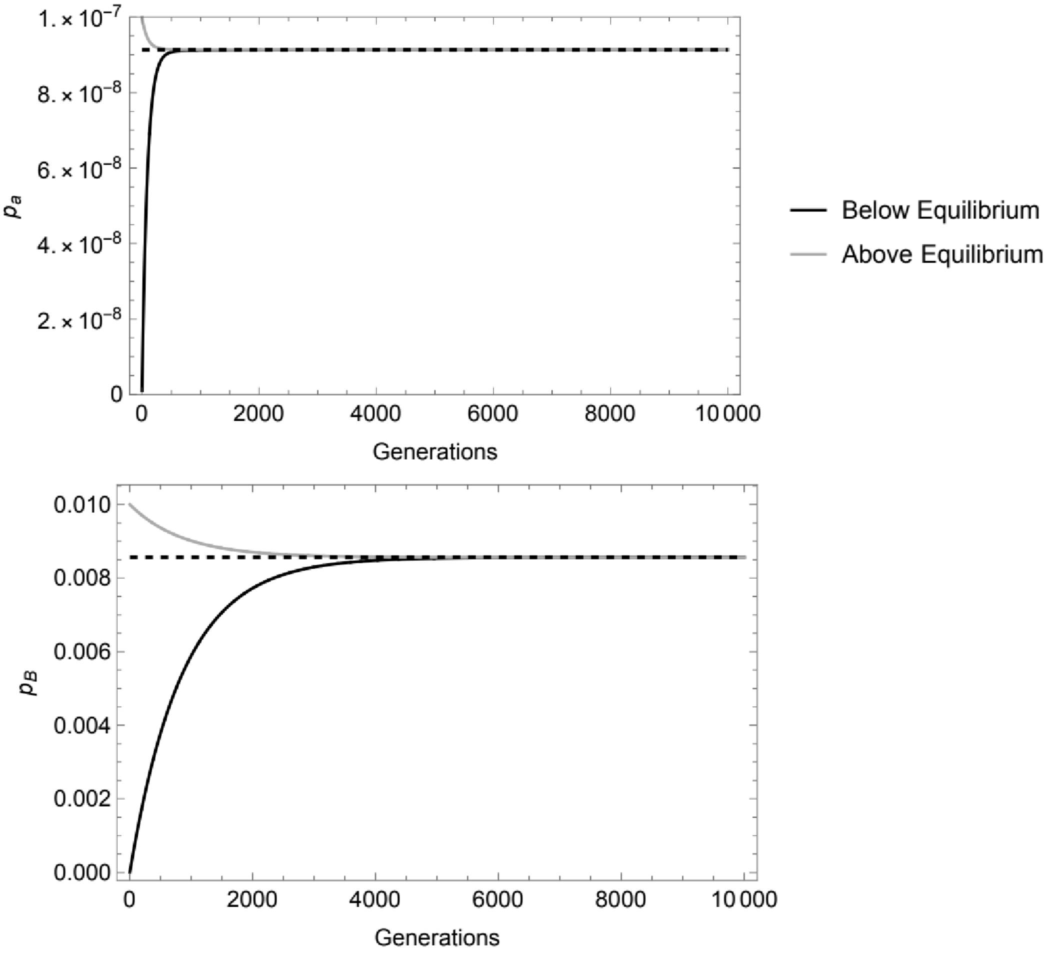


Figure D4: Plots of the deleterious allele frequency (top) and the epiallele frequency (bottom) across time in generations. The numerical parameter values are f = 0.1, s = 0.02, $s_2$ = 0.01, $s_3$ = 0.001, h = 0.5, $h_2$ = 0.5, u = 10^−9, $t_1$ = 10^−5, $t_2$ = 0.2, u = 10^−9, c = 10^−9, z = 10^−9, m = 0.005

# Case E: The Deleterious Allele is Paramutagenic (Aa -> Ba at rate $m_2$)

The recursion equations for this case are

$$p_B' = \big((-1+c)\big((-1+f)(-1+p_a+p_B)^2 t_1 w_{A,A} - p_B(-1+t_2)((-1+f)p_a w_{B,a} + (-f+(-1+f)p_B) w_{B,B}) - (1-p_a-p_B)(t_1(f w_{A,A} + (-1+f)((-1+m_2)p_a w_{A,a} + (-1+m_1)p_B w_{A,B})) - (-1+f)(-1+t_2)(-m_2 p_a w_{B,a} + p_B((-1+m_1)w_{A,B} - 2 m_1 w_{B,B}))))\big)\big/ \big(p_a(-f+(-1+f)p_a)w_{a,a} + (-1+f)(-1+p_a+p_B)^2 w_{A,A} + (-1+f)p_B^2 w_{B,B} - p_B(-2(-1+f)p_a w_{B,a} + f w_{B,B}) - (1-p_a-p_B)(f w_{A,A} + 2(-1+f)(p_a((-1+m_2)w_{A,a} - m_2 w_{B,a}) + p_B((-1+m_1)w_{A,B} - m_1 w_{B,B})))\big),$$

$$p_a' = \big((-1+z)p_a(-f+(-1+f)p_a)w_{a,a} + (-1+f)(-1+p_a+p_B)^2(-u+(-c+u)t_1)w_{A,A} + (-1+f)p_B^2(-c+(c-u)t_2)w_{B,B} + p_B((-1+f)p_a(-1-c+z+(c-u)t_2)w_{B,a} + f(c+(-c+u)t_2)w_{B,B}) + (1-p_a-p_B)(f(u+(c-u)t_1)w_{A,A} + (-1+f)(-p_a((-1+m_2)(-1-u+z+(-c+u)t_1)w_{A,a} + m_2(1+c-z+(-c+u)t_2)w_{B,a}) + p_B((-1+m_1)(c+u+(c-u)t_1+(-c+u)t_2)w_{A,B} + 2m_1(-c+(c-u)t_2)w_{B,B}))))\big)\big/ \big(p_a(f-(-1+f)p_a)w_{a,a} - (-1+f)(-1+p_a+p_B)^2 w_{A,A} - (-1+f)p_B^2 w_{B,B} + p_B(-2(-1+f)p_a w_{B,a} + f w_{B,B}) + (1-p_a-p_B)(f w_{A,A} + 2(-1+f)(p_a((-1+m_2)w_{A,a} - m_2 w_{B,a}) + p_B((-1+m_1)w_{A,B} - m_1 w_{B,B})))\big)$$

Evaluating the accuracy of the analytical approximation (eq. 11 of the main text) in comparison to numerical equilibria solutions (Tables E1-E4):

Table E1: Evaluating the accuracy of the equilibrium approximation for the deleterious allele as the paramutation rate increases from a lower to upper limit such that the equilibrium is biologically valid

| $m_2$ | ~ $\hat{p}_a$ Analytical | $\hat{p}_a$ Numerical | Percentage Difference |
|---|---|---|---|
| 0.005 | $5.\times10^{-8}$ | $5.\times10^{-8}$ | 0. |
| 0.055 | $5.\times10^{-8}$ | $5.\times10^{-8}$ | 0. |
| 0.105 | $5.\times10^{-8}$ | $5.\times10^{-8}$ | 0. |
| 0.155 | $4.\times10^{-8}$ | $5.\times10^{-8}$ | 20. |
| 0.205 | $4.\times10^{-8}$ | $4.\times10^{-8}$ | 0. |
| 0.255 | $4.\times10^{-8}$ | $4.\times10^{-8}$ | 0. |
| 0.305 | $4.\times10^{-8}$ | $4.\times10^{-8}$ | 0. |
| 0.355 | $4.\times10^{-8}$ | $4.\times10^{-8}$ | 0. |
| 0.405 | $4.\times10^{-8}$ | $4.\times10^{-8}$ | 0. |
| 0.455 | $4.\times10^{-8}$ | $4.\times10^{-8}$ | 0. |
| 0.505 | $4.\times10^{-8}$ | $4.\times10^{-8}$ | 0. |
| 0.555 | $4.\times10^{-8}$ | $4.\times10^{-8}$ | 0. |
| 0.605 | $3.\times10^{-8}$ | $3.\times10^{-8}$ | 0. |
| 0.655 | $3.\times10^{-8}$ | $3.\times10^{-8}$ | 0. |
| 0.705 | $3.\times10^{-8}$ | $3.\times10^{-8}$ | 0. |
| 0.755 | $3.\times10^{-8}$ | $3.\times10^{-8}$ | 0. |
| 0.805 | $3.\times10^{-8}$ | $3.\times10^{-8}$ | 0. |
| 0.855 | $3.\times10^{-8}$ | $3.\times10^{-8}$ | 0. |

Parameter values: $w_{A,A} = 1$, $w_{A,a} = 0.99$, $w_{A,B} = 0.98$, $w_{B,B} = 0.97$, $w_{B,a} = 0.96$, $w_{a,a} = 0.97$, f = 0.5, m1 = 0.05, $t_1 = 10$^−5, $t_2 = 10$^−4, u = 10^−9, c = 10^−9, z = 10^−9

Table E2: Evaluating the accuracy of the equilibrium approximation for the epiallele as the paramutation rate increases from a lower to upper limit such that the equilibrium is biologically valid

| $m_2$ | ~ $\hat{p}_B$ Analytical | $\hat{p}_B$ Numerical | Percentage Difference |
|---|---|---|---|
| 0.005 | 0.0100001 | 0.00953541 | 4.65 |
| 0.055 | 0.0100013 | 0.00953657 | 4.65 |
| 0.105 | 0.0100023 | 0.00953765 | 4.65 |
| 0.155 | 0.0100033 | 0.00953866 | 4.65 |
| 0.205 | 0.0100043 | 0.00953961 | 4.64 |
| 0.255 | 0.0100051 | 0.00954049 | 4.64 |
| 0.305 | 0.010006 | 0.00954132 | 4.64 |
| 0.355 | 0.0100067 | 0.0095421 | 4.64 |
| 0.405 | 0.0100075 | 0.00954284 | 4.64 |
| 0.455 | 0.0100081 | 0.00954353 | 4.64 |
| 0.505 | 0.0100088 | 0.00954419 | 4.64 |
| 0.555 | 0.0100094 | 0.00954481 | 4.64 |
| 0.605 | 0.01001 | 0.0095454 | 4.64 |
| 0.655 | 0.0100105 | 0.00954596 | 4.64 |
| 0.705 | 0.0100111 | 0.0095465 | 4.64 |
| 0.755 | 0.0100116 | 0.009547 | 4.64 |
| 0.805 | 0.0100121 | 0.00954749 | 4.64 |
| 0.855 | 0.0100125 | 0.00954795 | 4.64 |

Parameter values: $w_{A,A} = 1$, $w_{A,a} = 0.99$, $w_{A,B} = 0.98$, $w_{B,B} = 0.97$, $w_{B,a} = 0.96$, $w_{a,a} = 0.97$, f = 0.5, m1 = 0.05, $t_1 = 10$ ^ −5, $t_2 = 10$ ^ −4, u = 10 ^ −9, c = 10 ^ −9, z = 10 ^ −9

Table E3: Evaluating the accuracy of the equilibrium approximation for the deleterious allele as the inbreeding coefficient varies from a lower to upper limit such that the equilibrium is biologically valid

| f | ~ $\hat{p}_a$ Analytical | $\hat{p}_a$ Numerical | Percentage Difference |
|---|---|---|---|
| 0.51 | $5. \times 10^{-8}$ | $5. \times 10^{-8}$ | 0. |
| 0.56 | $5. \times 10^{-8}$ | $5. \times 10^{-8}$ | 0. |
| 0.61 | $4. \times 10^{-8}$ | $4. \times 10^{-8}$ | 0. |
| 0.66 | $4. \times 10^{-8}$ | $4. \times 10^{-8}$ | 0. |
| 0.71 | $4. \times 10^{-8}$ | $4. \times 10^{-8}$ | 0. |
| 0.76 | $4. \times 10^{-8}$ | $4. \times 10^{-8}$ | 0. |
| 0.81 | $4. \times 10^{-8}$ | $4. \times 10^{-8}$ | 0. |
| 0.86 | $4. \times 10^{-8}$ | $4. \times 10^{-8}$ | 0. |
| 0.91 | $4. \times 10^{-8}$ | $4. \times 10^{-8}$ | 0. |
| 0.96 | $3. \times 10^{-8}$ | $3. \times 10^{-8}$ | 0. |

Parameter values: $w_{A,A} = 1$, $w_{A,a} = 0.99$, $w_{A,B} = 0.98$, $w_{B,B} = 0.97$, $w_{B,a} = 0.96$, $w_{a,a} = 0.97$, m2 = 0.05, m1 = 0.05, $t_1 = 10$ ^ −5, $t_2 = 10$ ^ −4, u = 10 ^ −9, c = 10 ^ −9, z = 10 ^ −9

Table E4: Evaluating the accuracy of the equilibrium approximation for the deleterious allele as the inbreeding coefficient varies from a lower to upper limit such that the equilibrium is biologically valid

| f | ~ $\hat{p}_B$ Analytical | $\hat{p}_B$ Numerical | Percentage Difference |
|---|---|---|---|
| 0.51 | 0.00632982 | 0.00606841 | 4.31 |
| 0.56 | 0.00223236 | 0.00218865 | 2. |
| 0.61 | 0.00135512 | 0.0013381 | 1.27 |
| 0.66 | 0.00097283 | 0.00096386 | 0.93 |
| 0.71 | 0.00075877 | 0.00075325 | 0.73 |
| 0.76 | 0.00062192 | 0.00061819 | 0.6 |
| 0.81 | 0.00052689 | 0.0005242 | 0.51 |
| 0.86 | 0.00045705 | 0.00045503 | 0.44 |
| 0.91 | 0.00040356 | 0.00040198 | 0.39 |
| 0.96 | 0.00036127 | 0.00036001 | 0.35 |

Parameter values: $w_{A,A}$ = 1, $w_{A,a}$ = 0.99, $w_{A,B}$ = 0.98, $w_{B,B}$ = 0.97, $w_{B,a}$ = 0.96, $w_{a,a}$ = 0.97, m2 = 0.05, m1 = 0.05, $t_1$ = 10 ^ −5, $t_2$ = 10 ^ −4, u = 10 ^ −9, c = 10 ^ −9, z = 10 ^ −9

Evaluating the accuracy of the analytical approximation (equivalent to eq. 9 (case D) of the main text) in comparison to numerical equilibria solutions (Tables E5-E8):

Table E5: Evaluating the accuracy of the equilibrium approximation for the deleterious allele as the paramutation rate increases from a lower to upper limit such that the equilibrium is biologically valid

| $m_2$ | ~ $\hat{p}_a$ Analytical | $\hat{p}_a$ Numerical | Percentage Difference |
|---|---|---|---|
| 0.005 | $5.\times 10^{-8}$ | $5.\times 10^{-8}$ | 0. |
| 0.055 | $5.\times 10^{-8}$ | $5.\times 10^{-8}$ | 0. |
| 0.105 | $5.\times 10^{-8}$ | $5.\times 10^{-8}$ | 0. |
| 0.155 | $5.\times 10^{-8}$ | $5.\times 10^{-8}$ | 0. |
| 0.205 | $5.\times 10^{-8}$ | $5.\times 10^{-8}$ | 0. |
| 0.255 | $5.\times 10^{-8}$ | $5.\times 10^{-8}$ | 0. |
| 0.305 | $5.\times 10^{-8}$ | $5.\times 10^{-8}$ | 0. |
| 0.355 | $5.\times 10^{-8}$ | $5.\times 10^{-8}$ | 0. |
| 0.405 | $5.\times 10^{-8}$ | $5.\times 10^{-8}$ | 0. |
| 0.455 | $5.\times 10^{-8}$ | $5.\times 10^{-8}$ | 0. |
| 0.505 | $5.\times 10^{-8}$ | $5.\times 10^{-8}$ | 0. |
| 0.555 | $5.\times 10^{-8}$ | $5.\times 10^{-8}$ | 0. |
| 0.605 | $5.\times 10^{-8}$ | $5.\times 10^{-8}$ | 0. |
| 0.655 | $5.\times 10^{-8}$ | $5.\times 10^{-8}$ | 0. |
| 0.705 | $5.\times 10^{-8}$ | $5.\times 10^{-8}$ | 0. |
| 0.755 | $5.\times 10^{-8}$ | $5.\times 10^{-8}$ | 0. |
| 0.805 | $5.\times 10^{-8}$ | $5.\times 10^{-8}$ | 0. |
| 0.855 | $5.\times 10^{-8}$ | $5.\times 10^{-8}$ | 0. |

Parameter values: $w_{A,A}$ = 1, $w_{A,a}$ = 0.99, $w_{A,B}$ = 0.985, $w_{B,B}$ = 0.98, $w_{B,a}$ = 0.96, $w_{a,a}$ = 0.97, f = 0.1, m1 = 0.1, $t_1$ = 10 ^ −5, $t_2$ = 10 ^ −4, u = 10 ^ −9, c = 10 ^ −9, z = 10 ^ −9

Table E6: Evaluating the accuracy of the equilibrium approximation for the epiallele as the paramutation rate increases from a lower to upper limit such that the equilibrium is biologically valid

| $m_2$ | ~ $\hat{p}_B$ Analytical | $\hat{p}_B$ Numerical | Percentage Difference |
|---|---|---|---|
| 0.005 | 0.998807 | 0.998807 | 0. |
| 0.055 | 0.998807 | 0.998807 | 0. |
| 0.105 | 0.998807 | 0.998807 | 0. |
| 0.155 | 0.998807 | 0.998807 | 0. |
| 0.205 | 0.998807 | 0.998807 | 0. |
| 0.255 | 0.998807 | 0.998807 | 0. |
| 0.305 | 0.998807 | 0.998807 | 0. |
| 0.355 | 0.998807 | 0.998807 | 0. |
| 0.405 | 0.998807 | 0.998807 | 0. |
| 0.455 | 0.998807 | 0.998807 | 0. |
| 0.505 | 0.998807 | 0.998807 | 0. |
| 0.555 | 0.998807 | 0.998807 | 0. |
| 0.605 | 0.998807 | 0.998807 | 0. |
| 0.655 | 0.998807 | 0.998807 | 0. |
| 0.705 | 0.998807 | 0.998807 | 0. |
| 0.755 | 0.998807 | 0.998807 | 0. |
| 0.805 | 0.998807 | 0.998807 | 0. |
| 0.855 | 0.998807 | 0.998807 | 0. |

Parameter values: $w_{A,A}$ = 1, $w_{A,a}$ = 0.99, $w_{A,B}$ = 0.985, $w_{B,B}$ = 0.98, $w_{B,a}$ = 0.96, $w_{a,a}$ = 0.97, f = 0.1, m1 = 0.1, $t_1$ = 10 ^ −5, $t_2$ = 10 ^ −4, u = 10 ^ −9, c = 10 ^ −9, z = 10 ^ −9

Table E7: Evaluating the accuracy of the equilibrium approximation for the deleterious allele as the inbreeding coefficient varies from a lower to upper limit such that the equilibrium is biologically valid

| f | ~ $\hat{p}_a$ Analytical | $\hat{p}_a$ Numerical | Percentage Difference |
|---|---|---|---|
| 0.01 | $5.\times 10^{-8}$ | $5.\times 10^{-8}$ | 0. |
| 0.06 | $5.\times 10^{-8}$ | $5.\times 10^{-8}$ | 0. |
| 0.11 | $5.\times 10^{-8}$ | $5.\times 10^{-8}$ | 0. |
| 0.16 | $5.\times 10^{-8}$ | $5.\times 10^{-8}$ | 0. |
| 0.21 | $5.\times 10^{-8}$ | $5.\times 10^{-8}$ | 0. |
| 0.26 | $6.\times 10^{-8}$ | $6.\times 10^{-8}$ | 0. |
| 0.31 | $6.\times 10^{-8}$ | $6.\times 10^{-8}$ | 0. |
| 0.36 | $6.\times 10^{-8}$ | $6.\times 10^{-8}$ | 0. |
| 0.41 | $6.\times 10^{-8}$ | $6.\times 10^{-8}$ | 0. |
| 0.46 | $6.\times 10^{-8}$ | $6.\times 10^{-8}$ | 0. |
| 0.51 | $7.\times 10^{-8}$ | $7.\times 10^{-8}$ | 0. |
| 0.56 | $7.\times 10^{-8}$ | $7.\times 10^{-8}$ | 0. |
| 0.61 | $7.\times 10^{-8}$ | $7.\times 10^{-8}$ | 0. |
| 0.66 | $7.\times 10^{-8}$ | $7.\times 10^{-8}$ | 0. |
| 0.71 | $8.\times 10^{-8}$ | $8.\times 10^{-8}$ | 0. |
| 0.76 | $8.\times 10^{-8}$ | $8.\times 10^{-8}$ | 0. |
| 0.81 | $8.\times 10^{-8}$ | $8.\times 10^{-8}$ | 0. |

Parameter values: $w_{A,A}$ = 1, $w_{A,a}$ = 0.99, $w_{A,B}$ = 0.985, $w_{B,B}$ = 0.98, $w_{B,a}$ = 0.96, $w_{a,a}$ = 0.97, m2 = 0.1, m1 = 0.1, $t_1$ = 10 ^ −5, $t_2$ = 10 ^ −4, u = 10 ^ −9, c = 10 ^ −9, z = 10 ^ −9

Table E8: Evaluating the accuracy of the equilibrium approximation for the epiallele as the inbreeding coefficient varies from a lower to upper limit such that the equilibrium is biologically valid

| f | ~ $\hat{p}_B$ Analytical | $\hat{p}_B$ Numerical | Percentage Difference |
|---|---|---|---|
| 0.01 | 0.998939 | 0.998939 | 0. |
| 0.06 | 0.998869 | 0.998869 | 0. |
| 0.11 | 0.99879 | 0.99879 | 0. |
| 0.16 | 0.998699 | 0.998699 | 0. |
| 0.21 | 0.998593 | 0.998593 | 0. |
| 0.26 | 0.998468 | 0.998468 | 0. |
| 0.31 | 0.998319 | 0.998319 | 0. |
| 0.36 | 0.998138 | 0.998138 | 0. |
| 0.41 | 0.997913 | 0.997913 | 0. |
| 0.46 | 0.997626 | 0.997626 | 0. |
| 0.51 | 0.997248 | 0.997247 | 0. |
| 0.56 | 0.996727 | 0.996726 | 0. |
| 0.61 | 0.995961 | 0.995959 | 0. |
| 0.66 | 0.994728 | 0.994724 | 0. |
| 0.71 | 0.992412 | 0.992402 | 0. |
| 0.76 | 0.986464 | 0.986411 | 0.01 |
| 0.81 | 0.93738 | 0.931487 | 0.63 |

Parameter values: $w_{A,A}$ = 1, $w_{A,a}$ = 0.99, $w_{A,B}$ = 0.985, $w_{B,B}$ = 0.98, $w_{B,a}$ = 0.96, $w_{a,a}$ = 0.97, m2 = 0.1, m1 = 0.1, $t_1$ = 10 ^ −5, $t_2$ = 10 ^ −4, u = 10 ^ −9, c = 10 ^ −9, z = 10 ^ −9

Evaluating the accuracy of the analytical approximation (eq. 12 of the main text) in comparison to numerical equilibria solutions (Tables E9-E12):

Table E9: Evaluating the accuracy of the equilibrium approximation for the deleterious allele as the paramutation rate increases from a lower to upper limit such that the equilibrium is biologically valid

| $m_2$ | ~ $\hat{p}_a$ Analytical | $\hat{p}_a$ Numerical | Percentage Difference |
|---|---|---|---|
| 0.055 | 0.9999998 | 0.9999998 | 0. |
| 0.105 | 0.9999998 | 0.9999998 | 0. |
| 0.155 | 0.9999999 | 0.9999999 | 0. |
| 0.205 | 0.9999999 | 0.9999999 | 0. |
| 0.255 | 0.9999999 | 0.9999999 | 0. |
| 0.305 | 0.9999999 | 0.9999999 | 0. |
| 0.355 | 0.9999999 | 0.9999999 | 0. |
| 0.405 | 0.9999999 | 0.9999999 | 0. |
| 0.455 | 0.9999999 | 0.9999999 | 0. |
| 0.505 | 0.9999999 | 0.9999999 | 0. |
| 0.555 | 0.9999999 | 0.9999999 | 0. |
| 0.605 | 0.9999999 | 0.9999999 | 0. |
| 0.655 | 0.9999999 | 0.9999999 | 0. |
| 0.705 | 0.9999999 | 0.9999999 | 0. |
| 0.755 | 0.9999999 | 0.9999999 | 0. |
| 0.805 | 0.9999999 | 0.9999999 | 0. |
| 0.855 | 0.9999999 | 0.9999999 | 0. |

Parameter values: $w_{A,A}$ = 1, $w_{A,a}$ = 0.99, $w_{A,B}$ = 0.985, $w_{B,B}$ = 0.98, $w_{B,a}$ = 0.96, $w_{a,a}$ = 0.97, f = 0.1, m1 = 0.1, $t_1$ = 10 ^ −5, $t_2$ = 10 ^ −4, u = 10 ^ −9, c = 10 ^ −9, z = 10 ^ −9

Table E10: Evaluating the accuracy of the equilibrium approximation for the epiallele as the paramutation rate increases from a lower to upper limit such that the equilibrium is biologically valid

| $m_2$ | ~ $\hat{p}_B$ Analytical | $\hat{p}_B$ Numerical | Percentage Difference |
|---|---|---|---|
| 0.055 | $2.\times10^{-7}$ | $2.\times10^{-7}$ | 0. |
| 0.105 | $2.\times10^{-7}$ | $2.\times10^{-7}$ | 0. |
| 0.155 | $1.\times10^{-7}$ | $1.\times10^{-7}$ | 0. |
| 0.205 | $1.\times10^{-7}$ | $1.\times10^{-7}$ | 0. |
| 0.255 | $1.\times10^{-7}$ | $1.\times10^{-7}$ | 0. |
| 0.305 | $1.\times10^{-7}$ | $1.\times10^{-7}$ | 0. |
| 0.355 | $1.\times10^{-7}$ | $1.\times10^{-7}$ | 0. |
| 0.405 | $1.\times10^{-7}$ | $1.\times10^{-7}$ | 0. |
| 0.455 | $1.\times10^{-7}$ | $1.\times10^{-7}$ | 0. |
| 0.505 | $1.\times10^{-7}$ | $1.\times10^{-7}$ | 0. |
| 0.555 | $1.\times10^{-7}$ | $1.\times10^{-7}$ | 0. |
| 0.605 | $1.\times10^{-7}$ | $1.\times10^{-7}$ | 0. |
| 0.655 | $1.\times10^{-7}$ | $1.\times10^{-7}$ | 0. |
| 0.705 | $1.\times10^{-7}$ | $1.\times10^{-7}$ | 0. |
| 0.755 | $1.\times10^{-7}$ | $1.\times10^{-7}$ | 0. |
| 0.805 | $1.\times10^{-7}$ | $1.\times10^{-7}$ | 0. |
| 0.855 | $1.\times10^{-7}$ | $1.\times10^{-7}$ | 0. |

Parameter values: $w_{A,A}$ = 1, $w_{A,a}$ = 0.99, $w_{A,B}$ = 0.985, $w_{B,B}$ = 0.98, $w_{B,a}$ = 0.96, $w_{a,a}$ = 0.97, f = 0.1, m1 = 0.1, $t_1$ = 10 ^ −5, $t_2$ = 10 ^ −4, u = 10 ^ −9, c = 10 ^ −9, z = 10 ^ −9

Table E11: Evaluating the accuracy of the equilibrium approximation for the deleterious allele as the inbreeding coefficient varies from a lower to upper limit such that the equilibrium is biologically valid

| f | ~ $\hat{p}_a$ Analytical | $\hat{p}_a$ Numerical | Percentage Difference |
|---|---|---|---|
| 0.01 | 0.9999999 | 0.9999999 | 0. |
| 0.06 | 0.9999999 | 0.9999999 | 0. |
| 0.11 | 0.9999999 | 0.9999999 | 0. |
| 0.16 | 0.9999999 | 0.9999999 | 0. |
| 0.21 | 0.9999999 | 0.9999999 | 0. |
| 0.26 | 0.9999999 | 0.9999999 | 0. |
| 0.31 | 0.9999999 | 0.9999999 | 0. |
| 0.36 | 0.9999999 | 0.9999999 | 0. |
| 0.41 | 0.9999999 | 0.9999999 | 0. |
| 0.46 | 0.9999999 | 0.9999999 | 0. |
| 0.51 | 0.9999999 | 0.9999999 | 0. |
| 0.56 | 0.9999999 | 0.9999999 | 0. |
| 0.61 | 0.9999998 | 0.9999998 | 0. |
| 0.66 | 0.9999998 | 0.9999998 | 0. |
| 0.71 | 0.9999997 | 0.9999997 | 0. |
| 0.76 | 0.9999995 | 0.9999995 | 0. |
| 0.81 | 0.9999965 | 0.9999957 | 0. |

Parameter values: $w_{A,A}$ = 1, $w_{A,a}$ = 0.99, $w_{A,B}$ = 0.975, $w_{B,B}$ = 0.97, $w_{B,a}$ = 0.96, $w_{a,a}$ = 0.98, m2 = 0.1, m1 = 0.1, $t_1$ = 10 ^ −5, $t_2$ = 10 ^ −4, u = 10 ^ −9, c = 10 ^ −9, z = 10 ^ −9

Table E12: Evaluating the accuracy of the equilibrium approximation for the epiallele as the inbreeding coefficient varies from a lower to upper limit such that the equilibrium is biologically valid

| f | ~ $\hat{p}_B$ Analytical | $\hat{p}_B$ Numerical | Percentage Difference |
|---|---|---|---|
| 0.01 | $1.\times 10^{-7}$ | $1.\times 10^{-7}$ | 0. |
| 0.06 | $1.\times 10^{-7}$ | $1.\times 10^{-7}$ | 0. |
| 0.11 | $1.\times 10^{-7}$ | $1.\times 10^{-7}$ | 0. |
| 0.16 | $1.\times 10^{-7}$ | $1.\times 10^{-7}$ | 0. |
| 0.21 | $1.\times 10^{-7}$ | $1.\times 10^{-7}$ | 0. |
| 0.26 | $1.\times 10^{-7}$ | $1.\times 10^{-7}$ | 0. |
| 0.31 | $1.\times 10^{-7}$ | $1.\times 10^{-7}$ | 0. |
| 0.36 | $1.\times 10^{-7}$ | $1.\times 10^{-7}$ | 0. |
| 0.41 | $1.\times 10^{-7}$ | $1.\times 10^{-7}$ | 0. |
| 0.46 | $1.\times 10^{-7}$ | $1.\times 10^{-7}$ | 0. |
| 0.51 | $1.\times 10^{-7}$ | $1.\times 10^{-7}$ | 0. |
| 0.56 | $1.\times 10^{-7}$ | $1.\times 10^{-7}$ | 0. |
| 0.61 | $1.\times 10^{-7}$ | $1.\times 10^{-7}$ | 0. |
| 0.66 | $1.\times 10^{-7}$ | $1.\times 10^{-7}$ | 0. |
| 0.71 | $2.\times 10^{-7}$ | $2.\times 10^{-7}$ | 0. |
| 0.76 | $3.\times 10^{-7}$ | $3.\times 10^{-7}$ | 0. |
| 0.81 | $2.1\times 10^{-6}$ | $2.6\times 10^{-6}$ | 19.23 |

Parameter values: $w_{A,A} = 1$, $w_{A,a} = 0.99$, $w_{A,B} = 0.975$, $w_{B,B} = 0.97$, $w_{B,a} = 0.96$, $w_{a,a} = 0.98$, m2 = 0.1, m1 = 0.1, $t_1$ = 10 ^ −5, $t_2$ = 10 ^ −4, u = 10 ^ −9, c = 10 ^ −9, z = 10 ^ −9

## Local Stability analysis (following Appendix Section A2 )

The approximated eigenvalues corresponding with eq. 11 of the main text are

$$
\begin{aligned}
&\lambda_{(1)} \sim \frac{f\, w_{a,a} + w_{A,a} - f\, w_{A,a} - m_2\, w_{A,a} + f\, m_2\, w_{A,a} + m_2\, w_{B,a} - f\, m_2\, w_{B,a}}{w_{A,A}} + \\
&\frac{u\,(f\, w_{a,a} + 2\,(-1+f)\,(-1+m_2)\, w_{A,a} - 2\, w_{A,A} + f\, w_{A,A} + 2\, m_2\, w_{B,a} - 2\, f\, m_2\, w_{B,a})}{f\, w_{a,a} + (-1+f)\,(-1+m_2)\, w_{A,a} - w_{A,A} + m_2\, w_{B,a} - f\, m_2\, w_{B,a}} - \\
&\Bigg((-1+f)\, m_2\, w_{B,a} \Bigg(2\,(-1+f)\, u - f\, u + \frac{(-1+f)\, u\,((-1+m_2)\, w_{A,a} - m_2\, w_{B,a})}{f\, w_{a,a} + (-1+f)\,(-1+m_2)\, w_{A,a} - w_{A,A} + m_2\, w_{B,a} - f\, m_2\, w_{B,a}} - \\
&\frac{(-1+f)\, u\, w_{B,a}}{-f\, w_{a,a} + (-1+f+m_2-f\, m_2)\, w_{A,a} + w_{A,A} - m_2\, w_{B,a} + f\, m_2\, w_{B,a}} + \frac{c\, f\, w_{B,B}}{w_{A,A}} + \\
&\frac{(-1+f)\,((c+u)\,(-1+m_1)\, w_{A,B} - 2\, c\, m_1\, w_{B,B})}{w_{A,A}} + \frac{u\,((-2+f)\, w_{A,A} + 2\,(-1+f)\,(-1+m_1)\, w_{A,B} + (f + 2\, m_1 - 2\, f\, m_1)\, w_{B,B})}{f\, w_{a,a} + (-1+f)\,(-1+m_2)\, w_{A,a} - w_{A,A} + m_2\, w_{B,a} - f\, m_2\, w_{B,a}}\Bigg)\Bigg) \Big/ \\
&(f\, w_{a,a} + (-1+f)\,(-1+m_2)\, w_{A,a} - w_{A,B} + f\, w_{A,B} + m_1\, w_{A,B} - f\, m_1\, w_{A,B} + m_2\, w_{B,a} - f\, m_2\, w_{B,a} - f\, w_{B,B} - 2\, m_1\, w_{B,B} + 2\, f\, m_1\, w_{B,B}) + \\
&((f\, w_{a,a} + (-1+f)\,((-1+m_2)\, w_{A,a} - m_2\, w_{B,a})) \\
&(2\, u\, w_{A,A}^2 - f\, u\, w_{A,A}^2 + 2\, t_1\, w_{A,A}^2 - f\, t_1\, w_{A,A}^2 - 2\, u\, w_{A,A}\, w_{A,B} + 3\, f\, u\, w_{A,A}\, w_{A,B} - f^2\, u\, w_{A,A}\, w_{A,B} + 2\, u\, m_1\, w_{A,A}\, w_{A,B} - 3\, f\, u\, m_1\, w_{A,A}\, w_{A,B} + \\
&f^2\, u\, m_1\, w_{A,A}\, w_{A,B} - 2\, t_1\, w_{A,A}\, w_{A,B} + 2\, f\, t_1\, w_{A,A}\, w_{A,B} + 2\, m_1\, t_1\, w_{A,A}\, w_{A,B} - 2\, f\, m_1\, t_1\, w_{A,A}\, w_{A,B} - f\, u\, m_2\, w_{A,A}\, w_{B,a} + \\
&f^2\, u\, m_2\, w_{A,A}\, w_{B,a} - 2\, m_2\, t_1\, w_{A,A}\, w_{B,a} + 3\, f\, m_2\, t_1\, w_{A,A}\, w_{B,a} - f^2\, m_2\, t_1\, w_{A,A}\, w_{B,a} + 2\, m_2\, t_1\, w_{A,B}\, w_{B,a} - 4\, f\, m_2\, t_1\, w_{A,B}\, w_{B,a} + \\
&2\, f^2\, m_2\, t_1\, w_{A,B}\, w_{B,a} - 2\, m_1\, m_2\, t_1\, w_{A,B}\, w_{B,a} + 4\, f\, m_1\, m_2\, t_1\, w_{A,B}\, w_{B,a} - 2\, f^2\, m_1\, m_2\, t_1\, w_{A,B}\, w_{B,a} - 2\, f\, u\, w_{A,A}\, w_{B,B} + f^2\, u\, w_{A,A}\, w_{B,B} - \\
&4\, u\, m_1\, w_{A,A}\, w_{B,B} + 6\, f\, u\, m_1\, w_{A,A}\, w_{B,B} - 2\, f^2\, u\, m_1\, w_{A,A}\, w_{B,B} - f\, t_1\, w_{A,A}\, w_{B,B} - 2\, m_1\, t_1\, w_{A,A}\, w_{B,B} + 2\, f\, m_1\, t_1\, w_{A,A}\, w_{B,B} + \\
&f\, u\, m_2\, w_{B,a}\, w_{B,B} - f^2\, u\, m_2\, w_{B,a}\, w_{B,B} + 2\, u\, m_1\, m_2\, w_{B,a}\, w_{B,B} - 4\, f\, u\, m_1\, m_2\, w_{B,a}\, w_{B,B} + 2\, f^2\, u\, m_1\, m_2\, w_{B,a}\, w_{B,B} + \\
&f\, m_2\, t_1\, w_{B,a}\, w_{B,B} - f^2\, m_2\, t_1\, w_{B,a}\, w_{B,B} + 2\, m_1\, m_2\, t_1\, w_{B,a}\, w_{B,B} - 4\, f\, m_1\, m_2\, t_1\, w_{B,a}\, w_{B,B} + 2\, f^2\, m_1\, m_2\, t_1\, w_{B,a}\, w_{B,B} + \\
&f\, w_{a,a}\,(-((u - (-2+f)\, t_1)\, w_{A,A}) + (-1+f)\,(-1+m_1)\,(u + 2\, t_1)\, w_{A,B} - (-2\, m_1 + f\,(-1 + 2\, m_1))\,(u + t_1)\, w_{B,B}) +
\end{aligned}
$$

$$(-1+f)\,(-1+m_2)\,w_{A,a}\,((-2\,u+(-2+f)\,t_1)\,w_{A,A}+2\,(-1+f)\,(-1+m_1)\,(u+t_1)\,w_{A,B}-(-2\,m_1+f\,(-1+2\,m_1))\,(2\,u+t_1)\,w_{B,B})))\Big/$$

$$(w_{A,A}\,(f\,w_{a,a}+(-1+f)\,(-1+m_2)\,w_{A,a}-w_{A,A}+m_2\,w_{B,a}-f\,m_2\,w_{B,a})\,(-w_{A,A}+(-1+f)\,(-1+m_1)\,w_{A,B}+(f+2\,m_1-2\,f\,m_1)\,w_{B,B}))-$$

$$\Bigg((-f\,w_{a,a}+(-1+f+m_2-f\,m_2)\,w_{A,a}+w_{A,B}-f\,w_{A,B}-m_1\,w_{A,B}+f\,m_1\,w_{A,B}-m_2\,w_{B,a}+f\,m_2\,w_{B,a}+f\,w_{B,B}+2\,m_1\,w_{B,B}-2\,f\,m_1\,w_{B,B})$$

$$\Bigg(2\,(-1+f)\,u\,w_{A,A}-f\,u\,w_{A,A}+\frac{(-1+f)\,u\,w_{a,a}\,w_{A,A}}{f\,w_{a,a}+(-1+f)\,(-1+m_2)\,w_{A,a}-w_{A,A}+m_2\,w_{B,a}-f\,m_2\,w_{B,a}}+$$

$$\frac{(-1+f)\,u\,w_{A,A}\,((-1+m_2)\,w_{A,a}-m_2\,w_{B,a})}{f\,w_{a,a}+(-1+f)\,(-1+m_2)\,w_{A,a}-w_{A,A}+m_2\,w_{B,a}-f\,m_2\,w_{B,a}}+$$

$$w_{a,a}\left(-f\,z+\frac{(-1+f)\,u\,w_{A,A}}{f\,w_{a,a}+(-1+f)\,(-1+m_2)\,w_{A,a}-w_{A,A}+m_2\,w_{B,a}-f\,m_2\,w_{B,a}}\right)+$$

$$((-1+f)\,w_{A,A}\,w_{B,a}\,((-1+f)\,u\,m_2\,w_{B,a}+t_1\,(f\,w_{a,a}+(-1+f)\,(-1+m_2)\,w_{A,a}-w_{A,A}+m_2\,w_{B,a}-f\,m_2\,w_{B,a})))\Big/$$

$$((f\,w_{a,a}+(-1+f)\,(-1+m_2)\,w_{A,a}-w_{A,A}+m_2\,w_{B,a}-f\,m_2\,w_{B,a})\,(-w_{A,A}+(-1+f)\,(-1+m_1)\,w_{A,B}+(f+2\,m_1-2\,f\,m_1)\,w_{B,B}))+$$

$$(-1+f)\,\big((u-z)\,(-1+m_2)\,w_{A,a}+(-c+z)\,m_2\,w_{B,a}+$$

$$(w_{A,A}\,((-1+m_2)\,w_{A,a}-m_2\,w_{B,a})\,(t_1\,(f\,w_{a,a}+(-1+f)\,(-1+m_2)\,w_{A,a}-w_{A,A}+m_2\,w_{B,a}-f\,m_2\,w_{B,a})+u\,(-w_{A,A}+$$

$$(-1+f)\,(-1+m_1)\,w_{A,B}-m_2\,w_{B,a}+f\,m_2\,w_{B,a}+f\,w_{B,B}+2\,m_1\,w_{B,B}-2\,f\,m_1\,w_{B,B})))\Big/((f\,w_{a,a}+(-1+f)$$

$$(-1+m_2)\,w_{A,a}-w_{A,A}+m_2\,w_{B,a}-f\,m_2\,w_{B,a})\,(-w_{A,A}+(-1+f)\,(-1+m_1)\,w_{A,B}+(f+2\,m_1-2\,f\,m_1)\,w_{B,B}))\big)\Bigg)\Bigg)\Big/$$

$$(w_{A,A}\,(f\,w_{a,a}+(-1+f)\,(-1+m_2)\,w_{A,a}-w_{A,B}+f\,w_{A,B}+m_1\,w_{A,B}-f\,m_1\,w_{A,B}+m_2\,w_{B,a}-f\,m_2\,w_{B,a}-f\,w_{B,B}-2\,m_1\,w_{B,B}+2\,f\,m_1\,w_{B,B}))+$$

$$O[\zeta^2]$$

$$\lambda_{(2)}\sim\frac{w_{A,B}-f\,w_{A,B}-m_1\,w_{A,B}+f\,m_1\,w_{A,B}+f\,w_{B,B}+2\,m_1\,w_{B,B}-2\,f\,m_1\,w_{B,B}}{w_{A,A}}+$$

$$\Bigg((w_{A,A}\,(-((-1+f)\,u\,m_2\,w_{B,a})+t_1\,(-f\,w_{a,a}+(-1+f+m_2-f\,m_2)\,w_{A,a}+w_{A,A}-m_2\,w_{B,a}+f\,m_2\,w_{B,a}))$$

$$(f\,w_{a,a}+(-1+f)\,(-1+m_2)\,w_{A,a}-w_{A,B}+f\,w_{A,B}+m_1\,w_{A,B}-f\,m_1\,w_{A,B}+m_2\,w_{B,a}-f\,m_2\,w_{B,a}-f\,w_{B,B}-2\,m_1\,w_{B,B}+2\,f\,m_1\,w_{B,B})$$

$$((-2+f)\,w_{A,A}+2\,(-1+f)\,(-1+m_1)\,w_{A,B}+(f+2\,m_1-2\,f\,m_1)\,w_{B,B}))\Big/$$

$$((f\,w_{a,a}+(-1+f)\,(-1+m_2)\,w_{A,a}-w_{A,A}+m_2\,w_{B,a}-f\,m_2\,w_{B,a})\,(-w_{A,A}+(-1+f)\,(-1+m_1)\,w_{A,B}+(f+2\,m_1-2\,f\,m_1)\,w_{B,B}))-$$

$$(-1+f)\,m_2\,w_{A,A}\,w_{B,a}\Bigg(2\,(-1+f)\,u-f\,u+\frac{(-1+f)\,u\,((-1+m_2)\,w_{A,a}-m_2\,w_{B,a})}{f\,w_{a,a}+(-1+f)\,(-1+m_2)\,w_{A,a}-w_{A,A}+m_2\,w_{B,a}-f\,m_2\,w_{B,a}}-$$

$$\frac{(-1+f)\,u\,w_{B,a}}{-f\,w_{a,a}+(-1+f+m_2-f\,m_2)\,w_{A,a}+w_{A,A}-m_2\,w_{B,a}+f\,m_2\,w_{B,a}}+\frac{c\,f\,w_{B,B}}{w_{A,A}}+$$

$$\frac{(-1+f)\,((c+u)\,(-1+m_1)\,w_{A,B}-2\,c\,m_1\,w_{B,B})}{w_{A,A}}+\frac{u\,((-2+f)\,w_{A,A}+2\,(-1+f)\,(-1+m_1)\,w_{A,B}+(f+2\,m_1-2\,f\,m_1)\,w_{B,B})}{f\,w_{a,a}+(-1+f)\,(-1+m_2)\,w_{A,a}-w_{A,A}+m_2\,w_{B,a}-f\,m_2\,w_{B,a}}\Bigg)+$$

$$\big((-f\,w_{a,a}+(-1+f+m_2-f\,m_2)\,w_{A,a}+w_{A,B}-f\,w_{A,B}-m_1\,w_{A,B}+f\,m_1\,w_{A,B}-m_2\,w_{B,a}+f\,m_2\,w_{B,a}+f\,w_{B,B}+2\,m_1\,w_{B,B}-2\,f\,m_1\,w_{B,B})$$

$$((-1+f)\,(-1+m_1)\,w_{A,B}+(f+2\,m_1-2\,f\,m_1)\,w_{B,B})\,\big(2\,u\,w_{A,A}^2-f\,u\,w_{A,A}^2+2\,t_1\,w_{A,A}^2-f\,t_1\,w_{A,A}^2-2\,u\,w_{A,A}\,w_{A,B}+$$

$$3\,f\,u\,w_{A,A}\,w_{A,B}-f^2\,u\,w_{A,A}\,w_{A,B}+2\,u\,m_1\,w_{A,A}\,w_{A,B}-3\,f\,u\,m_1\,w_{A,A}\,w_{A,B}+f^2\,u\,m_1\,w_{A,A}\,w_{A,B}-2\,t_1\,w_{A,A}\,w_{A,B}+$$

$$2\,f\,t_1\,w_{A,A}\,w_{A,B}+2\,m_1\,t_1\,w_{A,A}\,w_{A,B}-2\,f\,m_1\,t_1\,w_{A,A}\,w_{A,B}-f\,u\,m_2\,w_{A,A}\,w_{B,a}+f^2\,u\,m_2\,w_{A,A}\,w_{B,a}-2\,m_2\,t_1\,w_{A,A}\,w_{B,a}+$$

$$3\,f\,m_2\,t_1\,w_{A,A}\,w_{B,a}-f^2\,m_2\,t_1\,w_{A,A}\,w_{B,a}+2\,m_2\,t_1\,w_{A,B}\,w_{B,a}-4\,f\,m_2\,t_1\,w_{A,B}\,w_{B,a}+2\,f^2\,m_2\,t_1\,w_{A,B}\,w_{B,a}-$$

$$2\,m_1\,m_2\,t_1\,w_{A,B}\,w_{B,a}+4\,f\,m_1\,m_2\,t_1\,w_{A,B}\,w_{B,a}-2\,f^2\,m_1\,m_2\,t_1\,w_{A,B}\,w_{B,a}-2\,f\,u\,w_{A,A}\,w_{B,B}+f^2\,u\,w_{A,A}\,w_{B,B}-$$

$$4\,u\,m_1\,w_{A,A}\,w_{B,B}+6\,f\,u\,m_1\,w_{A,A}\,w_{B,B}-2\,f^2\,u\,m_1\,w_{A,A}\,w_{B,B}-f\,t_1\,w_{A,A}\,w_{B,B}-2\,m_1\,t_1\,w_{A,A}\,w_{B,B}+2\,f\,m_1\,t_1\,w_{A,A}\,w_{B,B}+$$

$$f\,u\,m_2\,w_{B,a}\,w_{B,B}-f^2\,u\,m_2\,w_{B,a}\,w_{B,B}+2\,u\,m_1\,m_2\,w_{B,a}\,w_{B,B}-4\,f\,u\,m_1\,m_2\,w_{B,a}\,w_{B,B}+2\,f^2\,u\,m_1\,m_2\,w_{B,a}\,w_{B,B}+$$

$$f\,m_2\,t_1\,w_{B,a}\,w_{B,B}-f^2\,m_2\,t_1\,w_{B,a}\,w_{B,B}+2\,m_1\,m_2\,t_1\,w_{B,a}\,w_{B,B}-4\,f\,m_1\,m_2\,t_1\,w_{B,a}\,w_{B,B}+2\,f^2\,m_1\,m_2\,t_1\,w_{B,a}\,w_{B,B}+$$

$$f\,w_{a,a}\,(-((u-(-2+f)\,t_1)\,w_{A,A})+(-1+f)\,(-1+m_1)\,(u+2\,t_1)\,w_{A,B}-(-2\,m_1+f\,(-1+2\,m_1))\,(u+t_1)\,w_{B,B})+(-1+f)$$

$$(-1+m_2)\,w_{A,a}\,((-2\,u+(-2+f)\,t_1)\,w_{A,A}+2\,(-1+f)\,(-1+m_1)\,(u+t_1)\,w_{A,B}-(-2\,m_1+f\,(-1+2\,m_1))\,(2\,u+t_1)\,w_{B,B})\big)\big)\Big/$$

$$((f\, w_{a,a} + (-1+f)(-1+m_2)\, w_{A,a} - w_{A,A} + m_2\, w_{B,a} - f\, m_2\, w_{B,a})\,(-w_{A,A} + (-1+f)(-1+m_1)\, w_{A,B} + (f+2\, m_1 - 2\, f\, m_1)\, w_{B,B})) -$$

$$(f\, w_{a,a} + (-1+f)(-1+m_2)\, w_{A,a} - w_{A,B} + f\, w_{A,B} + m_1\, w_{A,B} - f\, m_1\, w_{A,B} + m_2\, w_{B,a} - f\, m_2\, w_{B,a} - f\, w_{B,B} - 2\, m_1\, w_{B,B} + 2\, f\, m_1\, w_{B,B})$$

$$\Big(2\,(-1+f)\, t_1\, w_{A,A} - f\, t_1\, w_{A,A} + (-1+f)(-1+m_1)\, t_1\, w_{A,B} -$$

$$\frac{(-1+f)\, u\, w_{A,A}\, w_{B,a}}{-f\, w_{a,a} + (-1+f+m_2-f\, m_2)\, w_{A,a} + w_{A,A} - m_2\, w_{B,a} + f\, m_2\, w_{B,a}} - f\, t_2\, w_{B,B} - (-1+f)\, t_2\, ((-1+m_1)\, w_{A,B} - 2\, m_1\, w_{B,B}) +$$

$$(2\,(-1+f)\, w_{A,A}\, ((-1+f)\, u\, m_2\, w_{B,a} + t_1\, (f\, w_{a,a} + (-1+f)(-1+m_2)\, w_{A,a} - w_{A,A} + m_2\, w_{B,a} - f\, m_2\, w_{B,a}))\, w_{B,B}) \Big/$$

$$((f\, w_{a,a} + (-1+f)(-1+m_2)\, w_{A,a} - w_{A,A} + m_2\, w_{B,a} - f\, m_2\, w_{B,a})\,(-w_{A,A} + (-1+f)(-1+m_1)\, w_{A,B} + (f+2\, m_1 - 2\, f\, m_1)\, w_{B,B})) +$$

$$c\,((-1+f+m_1-f\, m_1)\, w_{A,B} + (-2\, m_1 + f\,(-1+2\, m_1))\, w_{B,B}) +$$

$$((-1+f)\, w_{A,A}\, ((-1+m_1)\, w_{A,B} - 2\, m_1\, w_{B,B})\,(t_1\, (f\, w_{a,a} + (-1+f)(-1+m_2)\, w_{A,a} - w_{A,A} + m_2\, w_{B,a} - f\, m_2\, w_{B,a}) +$$

$$u\,(-w_{A,A} + (-1+f)(-1+m_1)\, w_{A,B} - m_2\, w_{B,a} + f\, m_2\, w_{B,a} + f\, w_{B,B} + 2\, m_1\, w_{B,B} - 2\, f\, m_1\, w_{B,B}))) \Big/$$

$$((f\, w_{a,a} + (-1+f)(-1+m_2)\, w_{A,a} - w_{A,A} + m_2\, w_{B,a} - f\, m_2\, w_{B,a})\,(-w_{A,A} + (-1+f)(-1+m_1)\, w_{A,B} + (f+2\, m_1 - 2\, f\, m_1)\, w_{B,B})) -$$

$$(-1+f)\, w_{A,A} \Big(-\frac{u\, m_2\, w_{B,a}}{-f\, w_{a,a} + (-1+f+m_2-f\, m_2)\, w_{A,a} + w_{A,A} - m_2\, w_{B,a} + f\, m_2\, w_{B,a}} -$$

$$(((-1+f)\, u\, m_2\, w_{B,a} + t_1\, (f\, w_{a,a} + (-1+f)(-1+m_2)\, w_{A,a} - w_{A,A} + m_2\, w_{B,a} - f\, m_2\, w_{B,a}))\,((-1+m_1)\, w_{A,B} - 2\, m_1\, w_{B,B})) \Big/$$

$$((f\, w_{a,a} + (-1+f)(-1+m_2)\, w_{A,a} - w_{A,A} + m_2\, w_{B,a} - f\, m_2\, w_{B,a})$$

$$(-w_{A,A} + (-1+f)(-1+m_1)\, w_{A,B} + (f+2\, m_1 - 2\, f\, m_1)\, w_{B,B}))\Big)\Big)\Big) \Big/$$

$$(w_{A,A}\,(-f\, w_{a,a} + (-1+f+m_2-f\, m_2)\, w_{A,a} + w_{A,B} - f\, w_{A,B} - m_1\, w_{A,B} + f\, m_1\, w_{A,B} - m_2\, w_{B,a} + f\, m_2\, w_{B,a} + f\, w_{B,B} + 2\, m_1\, w_{B,B} - 2\, f\, m_1\, w_{B,B}))$$

$$+ O[\zeta^2]$$

Numerically evaluating the approximated eigenvalues for local stability such that $|\lambda_{(1)}| < 1$ and $|\lambda_{(2)}| < 1$ (Tables E13 and E14):

Table E13: Numerically evaluating the approximated eigenvalues to determine local stability as the paramutation rate varies across a given range

| $m_2$ | $\sim\lambda_{(1)}$ | $\sim\lambda_{(2)}$ | Local Stability |
|---|---|---|---|
| 0.005 | 0.980124 | 0.999019 | Stable |
| 0.055 | 0.979381 | 0.999019 | Stable |
| 0.105 | 0.978638 | 0.999019 | Stable |
| 0.155 | 0.977895 | 0.999019 | Stable |
| 0.205 | 0.977153 | 0.999019 | Stable |
| 0.255 | 0.97641 | 0.999019 | Stable |
| 0.305 | 0.975667 | 0.999019 | Stable |
| 0.355 | 0.974924 | 0.999019 | Stable |
| 0.405 | 0.974182 | 0.999019 | Stable |
| 0.455 | 0.973439 | 0.99902 | Stable |
| 0.505 | 0.972696 | 0.99902 | Stable |
| 0.555 | 0.971953 | 0.99902 | Stable |
| 0.605 | 0.971211 | 0.99902 | Stable |
| 0.655 | 0.970468 | 0.99902 | Stable |
| 0.705 | 0.969725 | 0.99902 | Stable |
| 0.755 | 0.968982 | 0.99902 | Stable |
| 0.805 | 0.96824 | 0.99902 | Stable |
| 0.855 | 0.967497 | 0.99902 | Stable |

Parameter values: $w_{A,A} = 1$, $w_{A,a} = 0.99$, $w_{A,B} = 0.98$, $w_{B,B} = 0.97$,
$w_{B,a} = 0.96$, $w_{a,a} = 0.97$, $f = 0.5$, m1 = 0.05, $t_1 = 10^{\wedge}-5$, $t_2 = 10^{\wedge}-4$,

u = 10 ^ −9, c = 10 ^ −9, z = 10 ^ −9

Table E14: Numerically evaluating the approximated eigenvalues to determine local stability as the inbreeding coefficient varies across a given range

| f | $\sim\lambda_{(1)}$ | $\sim\lambda_{(2)}$ | Local Stability |
|---|---|---|---|
| 0.51 | 0.979196 | 0.998398 | Stable |
| 0.56 | 0.978188 | 0.995451 | Stable |
| 0.61 | 0.977245 | 0.992542 | Stable |
| 0.66 | 0.976313 | 0.989638 | Stable |
| 0.71 | 0.975383 | 0.986736 | Stable |
| 0.76 | 0.974456 | 0.983834 | Stable |
| 0.81 | 0.973529 | 0.980934 | Stable |
| 0.86 | 0.972602 | 0.978033 | Stable |
| 0.91 | 0.971676 | 0.975133 | Stable |
| 0.96 | 0.97075 | 0.972233 | Stable |

Parameter values: $w_{A,A}$ = 1, $w_{A,a}$ = 0.99, $w_{A,B}$ = 0.98, $w_{B,B}$ = 0.97,
$w_{B,a}$ = 0.96, $w_{a,a}$ = 0.97, m2 = 0.05, m1 = 0.05, $t_1$ = 10 ^ −5, $t_2$ = 10 ^ −4,
u = 10 ^ −9, c = 10 ^ −9, z = 10 ^ −9

The approximated eigenvalues corresponding with a 2nd equilibrium (equivalent to eq. 9 of the main text ) are

$$\lambda_{(1)} \sim \frac{f\,w_{A,A}+w_{A,B}-f\,w_{A,B}-m_1\,w_{A,B}+f\,m_1\,w_{A,B}}{w_{B,B}}+$$
$$(-2 f^2 t_2 w_{A,A}^2 ((-1+f) w_{B,a}+w_{B,B})+f\,w_{a,a} (2 f^2 t_2 w_{A,A}^2+(-1+f)(-1+m_1) w_{A,B} ((c-u)(f+(-1+f)(-1+m_1) w_{A,B}-w_{B,B})+$$
$$t_1 (-f-(-1+f)(-1+m_1) w_{A,B}+w_{B,B})+t_2 (-f+(-1+f)(-3+3 m_1) w_{A,B}+(-1+2 f+4 m_1-4 f m_1) w_{B,B}))+$$
$$w_{A,A} (f (c-u)(f+(-1+f)(-1+m_1) w_{A,B}-w_{B,B})+f\,t_1 (-f-(-1+f)(-1+m_1) w_{A,B}+w_{B,B})+$$
$$t_2 (-f^2+(-1+f) f (-5+5 m_1) w_{A,B}-(2+f-4 f m_1+f^2 (-2+4 m_1)) w_{B,B})))+$$
$$w_{A,A} (-((-1+f) w_{B,a} (f (2 c-u)(f+(-1+f)(-1+m_1) w_{A,B}-w_{B,B})+f\,t_1 (-f-(-1+f)(-1+m_1) w_{A,B}+w_{B,B})+$$
$$t_2 (-f^2+(-1+f) f (-5+5 m_1) w_{A,B}-(2+f-4 f m_1+f^2 (-2+4 m_1)) w_{B,B})))+$$
$$w_{B,B} (f (c (-2+f)+u)(f+(-1+f)(-1+m_1) w_{A,B}-w_{B,B})+f\,t_1 (f+(-1+f)(-1+m_1) w_{A,B}-w_{B,B})+$$
$$t_2 (f^2-(-1+f) f (-5+5 m_1) w_{A,B}+(2+f-4 f m_1+f^2 (-2+4 m_1)) w_{B,B})))-$$
$$(-1+f)(c (-1+m_2) w_{A,a} (f-w_{B,B}) w_{B,B}+(-1+f)(-1+m_1) w_{A,B}^2 ((-1+f)(-2 c+u+t_1+m_1 (2 c-u-t_1-t_2)-3 t_2+4 m_1 t_2) w_{B,a}+$$
$$(-c+c f+u+t_1-3 t_2+4 m_1 t_2-m_1 (-c+c f+u+t_1+t_2)) w_{B,B})+$$
$$w_{A,B} (-((-1+f)(-1+m_1) w_{B,a} (-((2 c-u)(f-w_{B,B}))+t_1 (f-w_{B,B})+t_2 (f+(1-2 f-4 m_1+4 f m_1) w_{B,B})))+$$
$$w_{B,B} (-c f+c f^2+f u+c f m_1-c f^2 m_1-f u m_1-c\,w_{A,a}+c f\,w_{A,a}+c m_1 w_{A,a}-c f m_1 w_{A,a}+c m_2 w_{A,a}-$$
$$c f m_2 w_{A,a}-c m_1 m_2 w_{A,a}+c f m_1 m_2 w_{A,a}-(-1+m_1) t_1 (f-w_{B,B})+c\,w_{B,B}-c f\,w_{B,B}-u\,w_{B,B}-$$
$$c m_1 w_{B,B}+c f m_1 w_{B,B}+u m_1 w_{B,B}-(-1+m_1) t_2 (f+(1-2 f-4 m_1+4 f m_1) w_{B,B})))))\Big/$$
$$((f+(-1+f)(-1+m_1) w_{A,B}-w_{B,B})(f\,w_{a,a}-(-1+f) w_{B,a}-w_{B,B}) w_{B,B})+$$
$$O[\zeta^2]$$

$$\lambda_{(2)} \sim \frac{f\,w_{a,a}+w_{B,a}-f\,w_{B,a}}{w_{B,B}}+$$
$$(f^2 w_{a,a}^2 ((c-z)(f+(-1+f)(-1+m_1) w_{A,B}-w_{B,B})+t_2 (f\,w_{A,A}+2 (-1+f)(-1+m_1) w_{A,B}+(-2+f+2 m_1-2 f m_1) w_{B,B}))+$$
$$(-1+f)((-1+m_2) t_2 w_{A,a} w_{B,B}^2+(-1+f) w_{B,a}^2$$
$$((3 c-z)(f+(-1+f)(-1+m_1) w_{A,B}-w_{B,B})+t_2 (f\,w_{A,A}+2 (-1+f)(-1+m_1) w_{A,B}+(-1+f+2 m_1-2 f m_1-m_2) w_{B,B}))+$$

$$w_{B,a}\,w_{B,B}\,(-((c\,(-1+2\,f)+z)\,(f+(-1+f)\,(-1+m_1)\,w_{A,B}-w_{B,B}))+t_2\,((-1+f)\,(-1+m_2)\,w_{A,a}+f\,w_{A,A}+2\,w_{A,B}-2\,f\,w_{A,B}-2\,m_1\,w_{A,B}+2\,f\,m_1\,w_{A,B}-w_{B,B}+f\,w_{B,B}+2\,m_1\,w_{B,B}-2\,f\,m_1\,w_{B,B}-m_2\,w_{B,B})))-$$
$$w_{a,a}\left(w_{B,B}\left(-\left(\left(c\left(-2-f+2\,f^2\right)+f\,z\right)(f+(-1+f)\,(-1+m_1)\,w_{A,B}-w_{B,B})\right)+f\,t_2\,((-1+f)\,(-1+m_2)\,w_{A,a}+f\,w_{A,A}+2\,w_{A,B}-2\,f\,w_{A,B}-2\,m_1\,w_{A,B}+2\,f\,m_1\,w_{A,B}-2\,w_{B,B}+f\,w_{B,B}+2\,m_1\,w_{B,B}-2\,f\,m_1\,w_{B,B})\right)+(-1+f)\,f\,w_{B,a}\,(2\,(2\,c-z)\,(f+(-1+f)\,(-1+m_1)\,w_{A,B}-w_{B,B})+t_2\,(2\,f\,w_{A,A}+4\,(-1+f)\,(-1+m_1)\,w_{A,B}-(3-2\,f-4\,m_1+4\,f\,m_1+m_2)\,w_{B,B}))\right)\Big)\Big/$$
$$((f+(-1+f)\,(-1+m_1)\,w_{A,B}-w_{B,B})\,(f\,w_{a,a}-(-1+f)\,w_{B,a}-w_{B,B})\,w_{B,B})$$
$$+\,O\left[\zeta^2\right]$$

Numerically evaluating the approximated eigenvalues for local stability such that $|\lambda_{(1)}| < 1$ and $|\lambda_{(2)}| < 1$ (Tables E15 and E16):

Table E15: Numerically evaluating the approximated eigenvalues to determine local stability as the paramutation rate varies across a given range

| $m_2$ | $\sim\lambda_{(1)}$ | $\sim\lambda_{(2)}$ | Local Stability |
|---|---|---|---|
| 0.005 | 0.916299 | 0.980633 | Stable |
| 0.055 | 0.916299 | 0.980631 | Stable |
| 0.105 | 0.916299 | 0.98063 | Stable |
| 0.155 | 0.916299 | 0.980628 | Stable |
| 0.205 | 0.916299 | 0.980626 | Stable |
| 0.255 | 0.916299 | 0.980625 | Stable |
| 0.305 | 0.916299 | 0.980623 | Stable |
| 0.355 | 0.916299 | 0.980621 | Stable |
| 0.405 | 0.916299 | 0.98062 | Stable |
| 0.455 | 0.916299 | 0.980618 | Stable |
| 0.505 | 0.916299 | 0.980616 | Stable |
| 0.555 | 0.916299 | 0.980615 | Stable |
| 0.605 | 0.916299 | 0.980613 | Stable |
| 0.655 | 0.916299 | 0.980612 | Stable |
| 0.705 | 0.916299 | 0.98061 | Stable |
| 0.755 | 0.916299 | 0.980608 | Stable |
| 0.805 | 0.916299 | 0.980607 | Stable |
| 0.855 | 0.916299 | 0.980605 | Stable |

Parameter values: $w_{A,A}$ = 1, $w_{A,a}$ = 0.99, $w_{A,B}$ = 0.985, $w_{B,B}$ = 0.98, $w_{B,a}$ = 0.96, $w_{a,a}$ = 0.97, f = 0.1, m1 = 0.1, $t_1$ = 10 ^ −5, $t_2$ = 10 ^ −4, u = 10 ^ −9, c = 10 ^ −9, z = 10 ^ −9

Table E16: Numerically evaluating the approximated eigenvalues to determine local stability as the inbreeding coefficient varies across a given range

| f | $\sim\lambda_{(1)}$ | $\sim\lambda_{(2)}$ | Local Stability |
|---|---|---|---|
| 0.01 | 0.905876 | 0.979713 | Stable |
| 0.06 | 0.911666 | 0.980222 | Stable |
| 0.11 | 0.917457 | 0.980732 | Stable |
| 0.16 | 0.923248 | 0.981241 | Stable |
| 0.21 | 0.929038 | 0.981749 | Stable |
| 0.26 | 0.934829 | 0.982258 | Stable |
| 0.31 | 0.94062 | 0.982766 | Stable |
| 0.36 | 0.946411 | 0.983274 | Stable |
| 0.41 | 0.952202 | 0.983781 | Stable |
| 0.46 | 0.957994 | 0.984288 | Stable |
| 0.51 | 0.963785 | 0.984793 | Stable |
| 0.56 | 0.969578 | 0.985296 | Stable |
| 0.61 | 0.975371 | 0.985796 | Stable |
| 0.66 | 0.981166 | 0.98629 | Stable |
| 0.71 | 0.986966 | 0.986769 | Stable |
| 0.76 | 0.99278 | 0.9872 | Stable |
| 0.81 | 0.998764 | 0.987054 | Stable |

Parameter values: $w_{A,A} = 1$, $w_{A,a} = 0.99$, $w_{A,B} = 0.985$,
$w_{B,B} = 0.98$, $w_{B,a} = 0.96$, $w_{a,a} = 0.97$, m2 = 0.1, m1 = 0.1,
$t_1 = 10^{-5}$, $t_2 = 10^{-4}$, $u = 10^{-9}$, $c = 10^{-9}$, $z = 10^{-9}$

The approximated eigenvalues corresponding with eq. 12 of the main text are

$$
\begin{gathered}
\lambda_{(1)} \sim \frac{w_{A,a} - f\, w_{A,a} - m_2\, w_{A,a} + f\, m_2\, w_{A,a} + f\, w_{A,A}}{w_{a,a}} + \\
\Big((-1+f)^2\, z\, m_2^2\, w_{a,a}\, w_{B,a}^2\, (2\,(-1+f+m_2-f\,m_2)\, w_{A,a} - f\, w_{A,A} + 2\, w_{B,a} - 2\, f\, w_{B,a} - 2\, m_2\, w_{B,a} + 2\, f\, m_2\, w_{B,a} + f\, w_{B,B}) + \\
z\,(-((-2+f)\, w_{a,a}) - (-1+f)\,((-1+m_2)\, w_{A,a} - m_2\, w_{B,a}))\,((-1+f)\,(-1+m_2)\, w_{A,a} + f\, w_{A,A} - w_{B,a} + f\, w_{B,a} + m_2\, w_{B,a} - f\, m_2\, w_{B,a} - f\, w_{B,B}) \\
((1-f)\, m_2\, w_{B,a}\,(2\,(-1+f)\, w_{B,a} - f\, w_{B,B}) + (2-f)\, w_{a,a}\,(w_{a,a} + (-1+f+m_2-f\,m_2)\, w_{B,a} - f\, w_{B,B}) + \\
(f\, w_{A,A} + 2\,(-1+f)\,((-1+m_2)\, w_{A,a} - m_2\, w_{B,a}))\,(-w_{a,a} - (-1+f)\, w_{B,a} + f\, w_{B,B})) - \\
(-1+f)\, z\, m_2\, w_{B,a}\,((-1+f+m_2-f\,m_2)\, w_{B,a} - f\, w_{B,B})\,((1-f)\, m_2\, w_{B,a}\,(2\,(-1+f)\, w_{B,a} - f\, w_{B,B}) + (2-f)\, w_{a,a} \\
(w_{a,a} + (-1+f+m_2-f\,m_2)\, w_{B,a} - f\, w_{B,B}) + (f\, w_{A,A} + 2\,(-1+f)\,((-1+m_2)\, w_{A,a} - m_2\, w_{B,a}))\,(-w_{a,a} - (-1+f)\, w_{B,a} + f\, w_{B,B})) - \\
z\,(w_{a,a} - (-1+f)\, w_{a,a} - f\, w_{A,A} - 2\,(-1+f)\,((-1+m_2)\, w_{A,a} - m_2\, w_{B,a}))\,((-1+f)\,(-1+m_2)\, w_{A,a} + f\, w_{A,A} - w_{B,a} + \\
f\, w_{B,a} + m_2\, w_{B,a} - f\, m_2\, w_{B,a} - f\, w_{B,B})\,\Big((-3+f)\, w_{a,a}^2 + 2\,(-1+f)\,(-1+m_2)\, w_{A,a}\,((-1+f)\, w_{B,a} - f\, w_{B,B}) + \\
w_{a,a}\,\big(2\,(-1+f)\,(-1+m_2)\, w_{A,a} + f\, w_{A,A} + 3\, w_{B,a} - 4\, f\, w_{B,a} + f^2\, w_{B,a} - m_2\, w_{B,a} + 2\, f\, m_2\, w_{B,a} - f^2\, m_2\, w_{B,a} + 3\, f\, w_{B,B} - f^2\, w_{B,B}\big) + \\
f\,((-1+f)\, m_2\, w_{B,a}\, w_{B,B} + w_{A,A}\,((-1+f)\, w_{B,a} - f\, w_{B,B}))\Big) + \\
((-1+f)\,(-1+m_2)\, w_{A,a} + f\, w_{A,A} - w_{B,a} + f\, w_{B,a} + m_2\, w_{B,a} - f\, m_2\, w_{B,a} - f\, w_{B,B}) \\
\Big((-1+f)^2\, z\, m_2\, w_{a,a}\, w_{B,a}^2 - f\, u\, w_{A,A}\,(w_{a,a} + (-1+f+m_2-f\,m_2)\, w_{A,a} - f\, w_{A,A})\,(w_{a,a} + (-1+f)\, w_{B,a} - f\, w_{B,B}) + \\
(-1+f)\, z\, w_{a,a}\,((-1+m_2)\, w_{A,a} - m_2\, w_{B,a})\,(w_{a,a} + (-1+f)\, w_{B,a} - f\, w_{B,B}) - (1-f)\, z\, w_{a,a} \\
((w_{a,a} + (-1+f+m_2-f\,m_2)\, w_{A,a} - f\, w_{A,A})\,(w_{a,a} + (-1+f)\, w_{B,a} - f\, w_{B,B}) + w_{a,a}\,(w_{a,a} + (-1+f+m_2-f\,m_2)\, w_{B,a} - f\, w_{B,B})) - \\
z\, w_{a,a}\,((w_{a,a} + (-1+f+m_2-f\,m_2)\, w_{A,a} - f\, w_{A,A})\,(w_{a,a} + (-1+f)\, w_{B,a} - f\, w_{B,B}) + \\
(1-f)\, w_{a,a}\,(w_{a,a} + (-1+f+m_2-f\,m_2)\, w_{B,a} - f\, w_{B,B})) - \\
(1-f)\,((u-z)\,(1-m_2)\, w_{A,a}\,(w_{a,a} + (-1+f+m_2-f\,m_2)\, w_{A,a} - f\, w_{A,A})\,(w_{a,a} + (-1+f)\, w_{B,a} - f\, w_{B,B}) + \\
(c-z)\, m_2\,(w_{a,a} + (-1+f+m_2-f\,m_2)\, w_{A,a} - f\, w_{A,A})\, w_{B,a}\,(w_{a,a} + (-1+f)\, w_{B,a} - f\, w_{B,B}) + \\
z\, w_{a,a}\,((-1+m_2)\, w_{A,a} - m_2\, w_{B,a})\,(w_{a,a} + (-1+f+m_2-f\,m_2)\, w_{B,a} - f\, w_{B,B}))\Big) + \\
(-1+f)\, m_2\, w_{B,a}\,\big(2\,(-1+f)^2\, z\, m_2\, w_{a,a}\, w_{B,a}\, w_{B,B} - t_1\,(w_{a,a} + (-1+f+m_2-f\,m_2)\, w_{A,a} - f\, w_{A,A})
\end{gathered}
$$

$$((-1+f)(-1+m_2)\, w_{A,a}+f\, w_{A,A})\,(w_{a,a}+(-1+f)\, w_{B,a}-f\, w_{B,B})+$$
$$t_2\,(w_{a,a}+(-1+f+m_2-f\, m_2)\, w_{A,a}-f\, w_{A,A})\,((-1+f)\, w_{B,a}-f\, w_{B,B})\,(w_{a,a}+(-1+f)\, w_{B,a}-f\, w_{B,B})+$$
$$c\,(w_{a,a}+(-1+f+m_2-f\, m_2)\, w_{A,a}-f\, w_{A,A})\,(w_{a,a}+(-1+f)\, w_{B,a}-f\, w_{B,B})\,((-1+f+m_2-f\, m_2)\, w_{B,a}-f\, w_{B,B})-$$
$$(1-f)\, z\, w_{a,a}\, w_{B,a}\,(w_{a,a}+(-1+f+m_2-f\, m_2)\, w_{B,a}-f\, w_{B,B})-(-1+f)\, z\, w_{a,a}\,(-w_{a,a}-(-1+f)\, w_{B,a}+f\, w_{B,B})$$
$$((-1+m_1)\, w_{A,B}-2\, m_1\, w_{B,B})+(1-f)\, m_2\, w_{B,a}\,(t_2\,(w_{a,a}+(-1+f+m_2-f\, m_2)\, w_{A,a}-f\, w_{A,A})\,(w_{a,a}+(-1+f)\, w_{B,a}-f\, w_{B,B})+$$
$$z\, w_{a,a}\,(w_{a,a}+(-1+f+m_2-f\, m_2)\, w_{B,a}-f\, w_{B,B})+(1-f)\, z\, w_{a,a}\,((-1+m_1)\, w_{A,B}-2\, m_1\, w_{B,B})))+$$
$$((-1+f)(-1+m_2)\, w_{A,a}+f\, w_{A,A}-w_{B,a}+f\, w_{B,a}+m_2\, w_{B,a}-f\, m_2\, w_{B,a}-f\, w_{B,B})$$
$$((-1+f)^2\, z\, m_2\, w_{a,a}\, w_{B,a}^2-(-1+f)\, z\, m_2\, w_{a,a}\, w_{B,a}\,((-2+f)\, w_{a,a}+2\,(-1+f)(-1+m_2)\, w_{A,a}+f\, w_{A,A}+2\, m_2\, w_{B,a}-2\, f\, m_2\, w_{B,a})-$$
$$t_1\,(w_{a,a}+(-1+f+m_2-f\, m_2)\, w_{A,a}-f\, w_{A,A})\,((-1+f)(-1+m_2)\, w_{A,a}+f\, w_{A,A})\,(w_{a,a}+(-1+f)\, w_{B,a}-f\, w_{B,B})-(-1+f)\, z\, m_2\, w_{a,a}$$
$$w_{B,a}\,(w_{a,a}+(-1+f)\, w_{B,a}-f\, w_{B,B})+c\,(1-f)\, m_2\,(w_{a,a}+(-1+f+m_2-f\, m_2)\, w_{A,a}-f\, w_{A,A})\, w_{B,a}\,(w_{a,a}+(-1+f)\, w_{B,a}-f\, w_{B,B})-$$
$$(1-f)\, z\, m_2\, w_{B,a}\,((1-f)\, m_2\, w_{B,a}\,(2\,(-1+f)\, w_{B,a}-f\, w_{B,B})+(2-f)\, w_{a,a}\,(w_{a,a}+(-1+f+m_2-f\, m_2)\, w_{B,a}-f\, w_{B,B})+$$
$$(f\, w_{A,A}+2\,(-1+f)\,((-1+m_2)\, w_{A,a}-m_2\, w_{B,a}))\,(-w_{a,a}-(-1+f)\, w_{B,a}+f\, w_{B,B}))+$$
$$(1-f)\, m_2\, w_{B,a}\,(t_2\,(w_{a,a}+(-1+f+m_2-f\, m_2)\, w_{A,a}-f\, w_{A,A})\,(w_{a,a}+(-1+f)\, w_{B,a}-f\, w_{B,B})+$$
$$z\, w_{a,a}\,(w_{a,a}+(-1+f+m_2-f\, m_2)\, w_{B,a}-f\, w_{B,B})+(1-f)\, z\, w_{a,a}\,((-1+m_1)\, w_{A,B}-2\, m_1\, w_{B,B})))-$$
$$2\, z\,((-1+f)(-1+m_2)\, w_{A,a}+f\, w_{A,A}-w_{B,a}+f\, w_{B,a}+m_2\, w_{B,a}-f\, m_2\, w_{B,a}-f\, w_{B,B})$$
$$((w_{a,a}-(-1+f)\, w_{a,a}-f\, w_{A,A}-2\,(-1+f)\,((-1+m_2)\, w_{A,a}-m_2\, w_{B,a}))$$
$$((1-f)\, m_2\, w_{B,a}\,(2\,(-1+f)\, w_{B,a}-f\, w_{B,B})+(2-f)\, w_{a,a}\,(w_{a,a}+(-1+f+m_2-f\, m_2)\, w_{B,a}-f\, w_{B,B})+$$
$$(f\, w_{A,A}+2\,(-1+f)\,((-1+m_2)\, w_{A,a}-m_2\, w_{B,a}))\,(-w_{a,a}-(-1+f)\, w_{B,a}+f\, w_{B,B}))+$$
$$(1-f)\, w_{a,a}\,(-((-1+f)\, m_2\, w_{B,a}^2)-w_{A,A}\,(w_{a,a}+(-1+f)\, w_{B,a}-f\, w_{B,B})-((-1+m_2)\, w_{A,a}-m_2\, w_{B,a})$$
$$(w_{a,a}+(-1+f)\, w_{B,a}-f\, w_{B,B})-w_{a,a}\,(w_{a,a}+(-1+f+m_2-f\, m_2)\, w_{B,a}-f\, w_{B,B})-$$
$$((-1+m_2)\, w_{A,a}-m_2\, w_{B,a})\,(w_{a,a}+(-1+f+m_2-f\, m_2)\, w_{B,a}-f\, w_{B,B})+(1-f)\, m_2\, w_{B,a}\,((-1+m_1)\, w_{A,B}-m_1\, w_{B,B})))+$$
$$(1-f)\, m_2\, w_{B,a}\,(f\, u\, w_{A,A}\,(w_{a,a}+(-1+f+m_2-f\, m_2)\, w_{A,a}-f\, w_{A,A})\,(w_{a,a}+(-1+f)\, w_{B,a}-f\, w_{B,B})-$$
$$c\, f\,(w_{a,a}+(-1+f+m_2-f\, m_2)\, w_{A,a}-f\, w_{A,A})\, w_{B,B}\,(w_{a,a}+(-1+f)\, w_{B,a}-f\, w_{B,B})-(1-f)\, w_{B,a}$$
$$(c\,(w_{a,a}+(-1+f+m_2-f\, m_2)\, w_{A,a}-f\, w_{A,A})\,(w_{a,a}+(-1+f)\, w_{B,a}-f\, w_{B,B})-z\,(w_{a,a}+(-1+f+m_2-f\, m_2)\, w_{A,a}-f\, w_{A,A})$$
$$(w_{a,a}+(-1+f)\, w_{B,a}-f\, w_{B,B})-z\, w_{a,a}\,(w_{a,a}+(-1+f+m_2-f\, m_2)\, w_{B,a}-f\, w_{B,B}))+$$
$$(1-f)\,((u-z)\,(1-m_2)\, w_{A,a}\,(w_{a,a}+(-1+f+m_2-f\, m_2)\, w_{A,a}-f\, w_{A,A})\,(w_{a,a}+(-1+f)\, w_{B,a}-f\, w_{B,B})+$$
$$(c-z)\, m_2\,(w_{a,a}+(-1+f+m_2-f\, m_2)\, w_{A,a}-f\, w_{A,A})\, w_{B,a}\,(w_{a,a}+(-1+f)\, w_{B,a}-f\, w_{B,B})+$$
$$z\, w_{a,a}\,((-1+m_2)\, w_{A,a}-m_2\, w_{B,a})\,(w_{a,a}+(-1+f+m_2-f\, m_2)\, w_{B,a}-f\, w_{B,B}))+(-1+f)\, z\,(-1+m_2)$$
$$(w_{A,a}-w_{B,a})\,((1-f)\, m_2\, w_{B,a}\,(2\,(-1+f)\, w_{B,a}-f\, w_{B,B})+(2-f)\, w_{a,a}\,(w_{a,a}+(-1+f+m_2-f\, m_2)\, w_{B,a}-f\, w_{B,B})+$$
$$(f\, w_{A,A}+2\,(-1+f)\,((-1+m_2)\, w_{A,a}-m_2\, w_{B,a}))\,(-w_{a,a}-(-1+f)\, w_{B,a}+f\, w_{B,B}))-$$
$$z\,(f\, w_{A,A}+2\,(-1+f)\, w_{B,a}+2\,(-1+f)\,((-1+m_2)\, w_{A,a}-m_2\, w_{B,a})-f\, w_{B,B})\,((-3+f)\, w_{a,a}^2+$$
$$2\,(-1+f)(-1+m_2)\, w_{A,a}\,((-1+f)\, w_{B,a}-f\, w_{B,B})+w_{a,a}\,(2\,(-1+f)(-1+m_2)\, w_{A,a}+f\, w_{A,A}+3\, w_{B,a}-4\, f\, w_{B,a}+f^2\, w_{B,a}-$$
$$m_2\, w_{B,a}+2\, f\, m_2\, w_{B,a}-f^2\, m_2\, w_{B,a}+3\, f\, w_{B,B}-f^2\, w_{B,B})+f\,((-1+f)\, m_2\, w_{B,a}\, w_{B,B}+w_{A,A}\,((-1+f)\, w_{B,a}-f\, w_{B,B})))+$$
$$2\, z\,(-((f\, w_{A,A}+2\,(-1+f)\, w_{B,a}+2\,(-1+f)\,((-1+m_2)\, w_{A,a}-m_2\, w_{B,a})-f\, w_{B,B})$$
$$((1-f)\, m_2\, w_{B,a}\,(2\,(-1+f)\, w_{B,a}-f\, w_{B,B})+(2-f)\, w_{a,a}\,(w_{a,a}+(-1+f+m_2-f\, m_2)\, w_{B,a}-f\, w_{B,B})+$$
$$(f\, w_{A,A}+2\,(-1+f)\,((-1+m_2)\, w_{A,a}-m_2\, w_{B,a}))\,(-w_{a,a}-(-1+f)\, w_{B,a}+f\, w_{B,B})))+(1-f)\, w_{a,a}$$
$$(-((-1+f)\, m_2\, w_{B,a}\, w_{B,B})-w_{A,A}\,(w_{a,a}+(-1+f)\, w_{B,a}-f\, w_{B,B})-w_{B,a}\,(w_{a,a}+(-1+f+m_2-f\, m_2)\, w_{B,a}-f\, w_{B,B})-$$
$$((-1+m_2)\, w_{A,a}-m_2\, w_{B,a})\,(w_{a,a}+(-1+f+m_2-f\, m_2)\, w_{B,a}-f\, w_{B,B})+$$
$$(1-f)\, m_2\, w_{B,a}\,((-1+m_1)\, w_{A,B}-m_1\, w_{B,B})+(-w_{a,a}-(-1+f)\, w_{B,a}+f\, w_{B,B})\,((-1+m_1)\, w_{A,B}-m_1\, w_{B,B})))))\Big/$$
$$(w_{a,a}\,(w_{a,a}+(-1+f+m_2-f\, m_2)\, w_{A,a}-f\, w_{A,A})\,(w_{a,a}+(-1+f)\, w_{B,a}-f\, w_{B,B})\,((-1+f)(-1+m_2)\, w_{A,a}+f\, w_{A,A}-w_{B,a}+f\, w_{B,a}-f\, w_{B,B}))$$
$$+\,O[\zeta^2]$$

$$\lambda_{(2)} \sim \frac{w_{B,a}-f\, w_{B,a}+f\, w_{B,B}}{w_{a,a}}+$$

$$(w_{a,a}^2\,(-z\, w_{A,B}\, w_{B,a}+2\, f\, z\, w_{A,B}\, w_{B,a}-f^2\, z\, w_{A,B}\, w_{B,a}+z\, m_1\, w_{A,B}\, w_{B,a}-2\, f\, z\, m_1\, w_{A,B}\, w_{B,a}+f^2\, z\, m_1\, w_{A,B}\, w_{B,a}-z\, m_2\, w_{A,B}\, w_{B,a}+$$
$$2\, f\, z\, m_2\, w_{A,B}\, w_{B,a}-f^2\, z\, m_2\, w_{A,B}\, w_{B,a}+z\, m_1\, m_2\, w_{A,B}\, w_{B,a}-2\, f\, z\, m_1\, m_2\, w_{A,B}\, w_{B,a}+f^2\, z\, m_1\, m_2\, w_{A,B}\, w_{B,a}+c\, w_{B,a}^2-$$

$$2\,c\,f\,w_{B,a}^2+c\,f^2\,w_{B,a}^2-z\,w_{B,a}^2+3\,f\,z\,w_{B,a}^2-3\,f^2\,z\,w_{B,a}^2+f^3\,z\,w_{B,a}^2+f\,z\,m_2\,w_{B,a}^2-2\,f^2\,z\,m_2\,w_{B,a}^2+f^3\,z\,m_2\,w_{B,a}^2+t_2\,w_{B,a}^2-$$
$$2\,f\,t_2\,w_{B,a}^2+f^2\,t_2\,w_{B,a}^2-m_2\,t_2\,w_{B,a}^2+2\,f\,m_2\,t_2\,w_{B,a}^2-f^2\,m_2\,t_2\,w_{B,a}^2-f\,z\,w_{A,B}\,w_{B,B}+f^2\,z\,w_{A,B}\,w_{B,B}+f\,z\,m_1\,w_{A,B}\,w_{B,B}-$$
$$f^2\,z\,m_1\,w_{A,B}\,w_{B,B}+2\,c\,f\,w_{B,a}\,w_{B,B}-2\,c\,f^2\,w_{B,a}\,w_{B,B}-3\,f\,z\,w_{B,a}\,w_{B,B}+5\,f^2\,z\,w_{B,a}\,w_{B,B}-2\,f^3\,z\,w_{B,a}\,w_{B,B}-2\,z\,m_1\,w_{B,a}\,w_{B,B}+$$
$$4\,f\,z\,m_1\,w_{B,a}\,w_{B,B}-2\,f^2\,z\,m_1\,w_{B,a}\,w_{B,B}+f^2\,z\,m_2\,w_{B,a}\,w_{B,B}-f^3\,z\,m_2\,w_{B,a}\,w_{B,B}+2\,f\,t_2\,w_{B,a}\,w_{B,B}-2\,f^2\,t_2\,w_{B,a}\,w_{B,B}-$$
$$f\,m_2\,t_2\,w_{B,a}\,w_{B,B}+f^2\,m_2\,t_2\,w_{B,a}\,w_{B,B}+c\,f^2\,w_{B,B}^2-2\,f^2\,z\,w_{B,B}^2+f^3\,z\,w_{B,B}^2-2\,f\,z\,m_1\,w_{B,B}^2+2\,f^2\,z\,m_1\,w_{B,B}^2+f^2\,t_2\,w_{B,B}^2+(-1+f)$$
$$(-1+m_2)\,w_{A,a}\left((-1+f)\,z\,(-1+m_1)\,w_{A,B}+(-1+f)\,(c+(-1+f)\,z+t_2)\,w_{B,a}-\left(c\,f+z\left(f^2+2\,f\,(-1+m_1)-2\,m_1\right)+f\,t_2\right)w_{B,B}\right)+$$
$$f\,w_{A,A}\left((-1+f)\,z\,(-1+m_1)\,w_{A,B}+(-1+f)\,(c+z\,(-1+f+m_2)+t_2)\,w_{B,a}-\left(c\,f+z\left(f^2+2\,f\,(-1+m_1)-2\,m_1\right)+f\,t_2\right)w_{B,B}\right)\Big)+$$
$$w_{a,a}\left(c\,f^2\,w_{A,A}^2\,w_{B,a}-c\,f^3\,w_{A,A}^2\,w_{B,a}-f^2\,z\,w_{A,A}^2\,w_{B,a}+f^3\,z\,w_{A,A}^2\,w_{B,a}+f^2\,t_2\,w_{A,A}^2\,w_{B,a}-f^3\,t_2\,w_{A,A}^2\,w_{B,a}-f\,z\,w_{A,A}\,w_{A,B}\,w_{B,a}+\right.$$
$$2\,f^2\,z\,w_{A,A}\,w_{A,B}\,w_{B,a}-f^3\,z\,w_{A,A}\,w_{A,B}\,w_{B,a}+f\,z\,m_1\,w_{A,A}\,w_{A,B}\,w_{B,a}-2\,f^2\,z\,m_1\,w_{A,A}\,w_{A,B}\,w_{B,a}+f^3\,z\,m_1\,w_{A,A}\,w_{A,B}\,w_{B,a}+$$
$$f^2\,z\,w_{A,A}\,w_{B,a}^2-2\,f^3\,z\,w_{A,A}\,w_{B,a}^2+f^4\,z\,w_{A,A}\,w_{B,a}^2-2\,f^2\,z\,m_2\,w_{A,A}\,w_{B,a}^2+4\,f^3\,z\,m_2\,w_{A,A}\,w_{B,a}^2-2\,f^4\,z\,m_2\,w_{A,A}\,w_{B,a}^2+$$
$$f\,m_2\,t_2\,w_{A,A}\,w_{B,a}^2-2\,f^2\,m_2\,t_2\,w_{A,A}\,w_{B,a}^2+f^3\,m_2\,t_2\,w_{A,A}\,w_{B,a}^2+z\,w_{A,B}\,w_{B,a}^2-3\,f\,z\,w_{A,B}\,w_{B,a}^2+3\,f^2\,z\,w_{A,B}\,w_{B,a}^2-$$
$$f^3\,z\,w_{A,B}\,w_{B,a}^2-z\,m_1\,w_{A,B}\,w_{B,a}^2+3\,f\,z\,m_1\,w_{A,B}\,w_{B,a}^2-3\,f^2\,z\,m_1\,w_{A,B}\,w_{B,a}^2+f^3\,z\,m_1\,w_{A,B}\,w_{B,a}^2+z\,m_2\,w_{A,B}\,w_{B,a}^2-$$
$$3\,f\,z\,m_2\,w_{A,B}\,w_{B,a}^2+3\,f^2\,z\,m_2\,w_{A,B}\,w_{B,a}^2-f^3\,z\,m_2\,w_{A,B}\,w_{B,a}^2-z\,m_1\,m_2\,w_{A,B}\,w_{B,a}^2+3\,f\,z\,m_1\,m_2\,w_{A,B}\,w_{B,a}^2-$$
$$3\,f^2\,z\,m_1\,m_2\,w_{A,B}\,w_{B,a}^2+f^3\,z\,m_1\,m_2\,w_{A,B}\,w_{B,a}^2-c\,w_{B,a}^3+3\,c\,f\,w_{B,a}^3-3\,c\,f^2\,w_{B,a}^3+c\,f^3\,w_{B,a}^3+z\,w_{B,a}^3-4\,f\,z\,w_{B,a}^3+$$
$$6\,f^2\,z\,w_{B,a}^3-4\,f^3\,z\,w_{B,a}^3+f^4\,z\,w_{B,a}^3+2\,z\,m_2\,w_{B,a}^3-5\,f\,z\,m_2\,w_{B,a}^3+3\,f^2\,z\,m_2\,w_{B,a}^3+f^3\,z\,m_2\,w_{B,a}^3-f^4\,z\,m_2\,w_{B,a}^3-t_2\,w_{B,a}^3+$$
$$3\,f\,t_2\,w_{B,a}^3-3\,f^2\,t_2\,w_{B,a}^3+f^3\,t_2\,w_{B,a}^3+m_2\,t_2\,w_{B,a}^3-3\,f\,m_2\,t_2\,w_{B,a}^3+3\,f^2\,m_2\,t_2\,w_{B,a}^3-f^3\,m_2\,t_2\,w_{B,a}^3+c\,f^3\,w_{A,A}^2\,w_{B,B}-$$
$$f^3\,z\,w_{A,A}^2\,w_{B,B}+f^3\,t_2\,w_{A,A}^2\,w_{B,B}-f^2\,z\,w_{A,A}\,w_{A,B}\,w_{B,B}+f^3\,z\,w_{A,A}\,w_{A,B}\,w_{B,B}+f^2\,z\,m_1\,w_{A,A}\,w_{A,B}\,w_{B,B}-f^3\,z\,m_1\,w_{A,A}\,w_{A,B}\,w_{B,B}-$$
$$f^2\,z\,w_{A,A}\,w_{B,a}\,w_{B,B}+3\,f^3\,z\,w_{A,A}\,w_{B,a}\,w_{B,B}-2\,f^4\,z\,w_{A,A}\,w_{B,a}\,w_{B,B}-2\,f\,z\,m_1\,w_{A,A}\,w_{B,a}\,w_{B,B}+4\,f^2\,z\,m_1\,w_{A,A}\,w_{B,a}\,w_{B,B}-$$
$$2\,f^3\,z\,m_1\,w_{A,A}\,w_{B,a}\,w_{B,B}+2\,f\,z\,m_2\,w_{A,A}\,w_{B,a}\,w_{B,B}-2\,f^2\,z\,m_2\,w_{A,A}\,w_{B,a}\,w_{B,B}-2\,f^3\,z\,m_2\,w_{A,A}\,w_{B,a}\,w_{B,B}+$$
$$2\,f^4\,z\,m_2\,w_{A,A}\,w_{B,a}\,w_{B,B}+f^2\,m_2\,t_2\,w_{A,A}\,w_{B,a}\,w_{B,B}-f^3\,m_2\,t_2\,w_{A,A}\,w_{B,a}\,w_{B,B}+2\,f\,z\,w_{A,B}\,w_{B,a}\,w_{B,B}-4\,f^2\,z\,w_{A,B}\,w_{B,a}\,w_{B,B}+$$
$$2\,f^3\,z\,w_{A,B}\,w_{B,a}\,w_{B,B}-2\,f\,z\,m_1\,w_{A,B}\,w_{B,a}\,w_{B,B}+4\,f^2\,z\,m_1\,w_{A,B}\,w_{B,a}\,w_{B,B}-2\,f^3\,z\,m_1\,w_{A,B}\,w_{B,a}\,w_{B,B}+f\,z\,m_2\,w_{A,B}\,w_{B,a}\,w_{B,B}-$$
$$2\,f^2\,z\,m_2\,w_{A,B}\,w_{B,a}\,w_{B,B}+f^3\,z\,m_2\,w_{A,B}\,w_{B,a}\,w_{B,B}-f\,z\,m_1\,m_2\,w_{A,B}\,w_{B,a}\,w_{B,B}+2\,f^2\,z\,m_1\,m_2\,w_{A,B}\,w_{B,a}\,w_{B,B}-$$
$$f^3\,z\,m_1\,m_2\,w_{A,B}\,w_{B,a}\,w_{B,B}-3\,c\,f\,w_{B,a}^2\,w_{B,B}+6\,c\,f^2\,w_{B,a}^2\,w_{B,B}-3\,c\,f^3\,w_{B,a}^2\,w_{B,B}+4\,f\,z\,w_{B,a}^2\,w_{B,B}-11\,f^2\,z\,w_{B,a}^2\,w_{B,B}+$$
$$10\,f^3\,z\,w_{B,a}^2\,w_{B,B}-3\,f^4\,z\,w_{B,a}^2\,w_{B,B}+2\,z\,m_1\,w_{B,a}^2\,w_{B,B}-6\,f\,z\,m_1\,w_{B,a}^2\,w_{B,B}+6\,f^2\,z\,m_1\,w_{B,a}^2\,w_{B,B}-2\,f^3\,z\,m_1\,w_{B,a}^2\,w_{B,B}-$$
$$2\,z\,m_2\,w_{B,a}^2\,w_{B,B}+7\,f\,z\,m_2\,w_{B,a}^2\,w_{B,B}-6\,f^2\,z\,m_2\,w_{B,a}^2\,w_{B,B}-f^3\,z\,m_2\,w_{B,a}^2\,w_{B,B}+2\,f^4\,z\,m_2\,w_{B,a}^2\,w_{B,B}-3\,f\,t_2\,w_{B,a}^2\,w_{B,B}+$$
$$6\,f^2\,t_2\,w_{B,a}^2\,w_{B,B}-3\,f^3\,t_2\,w_{B,a}^2\,w_{B,B}+2\,f\,m_2\,t_2\,w_{B,a}^2\,w_{B,B}-4\,f^2\,m_2\,t_2\,w_{B,a}^2\,w_{B,B}+2\,f^3\,m_2\,t_2\,w_{B,a}^2\,w_{B,B}-f^3\,z\,w_{A,A}\,w_{B,B}^2+$$
$$f^4\,z\,w_{A,A}\,w_{B,B}^2-2\,f^2\,z\,m_1\,w_{A,A}\,w_{B,B}^2+2\,f^3\,z\,m_1\,w_{A,A}\,w_{B,B}^2+f^2\,z\,w_{A,B}\,w_{B,B}^2-f^3\,z\,w_{A,B}\,w_{B,B}^2-f^2\,z\,m_1\,w_{A,B}\,w_{B,B}^2+$$
$$f^3\,z\,m_1\,w_{A,B}\,w_{B,B}^2-3\,c\,f^2\,w_{B,a}\,w_{B,B}^2+3\,c\,f^3\,w_{B,a}\,w_{B,B}^2+5\,f^2\,z\,w_{B,a}\,w_{B,B}^2-8\,f^3\,z\,w_{B,a}\,w_{B,B}^2+3\,f^4\,z\,w_{B,a}\,w_{B,B}^2+$$
$$4\,f\,z\,m_1\,w_{B,a}\,w_{B,B}^2-8\,f^2\,z\,m_1\,w_{B,a}\,w_{B,B}^2+4\,f^3\,z\,m_1\,w_{B,a}\,w_{B,B}^2-2\,f\,z\,m_2\,w_{B,a}\,w_{B,B}^2+3\,f^2\,z\,m_2\,w_{B,a}\,w_{B,B}^2-$$
$$f^4\,z\,m_2\,w_{B,a}\,w_{B,B}^2-3\,f^2\,t_2\,w_{B,a}\,w_{B,B}^2+3\,f^3\,t_2\,w_{B,a}\,w_{B,B}^2+f^2\,m_2\,t_2\,w_{B,a}\,w_{B,B}^2-f^3\,m_2\,t_2\,w_{B,a}\,w_{B,B}^2-c\,f^3\,w_{B,B}^3+2\,f^3\,z\,w_{B,B}^3-$$
$$f^4\,z\,w_{B,B}^3+2\,f^2\,z\,m_1\,w_{B,B}^3-2\,f^3\,z\,m_1\,w_{B,B}^3-f^3\,t_2\,w_{B,B}^3-(-1+f)^2\,(-1+m_2)^2\,(c-2\,z+t_2)\,w_{A,a}^2\,((-1+f)\,w_{B,a}-f\,w_{B,B})-$$
$$(-1+f)\,(-1+m_2)\,w_{A,a}\left(-z\,w_{B,a}^2+f\,z\,w_{B,a}^2+f^2\,z\,w_{B,a}^2-f^3\,z\,w_{B,a}^2+z\,m_2\,w_{B,a}^2-3\,f^2\,z\,m_2\,w_{B,a}^2+2\,f^3\,z\,m_2\,w_{B,a}^2-\right.$$
$$m_2\,t_2\,w_{B,a}^2+2\,f\,m_2\,t_2\,w_{B,a}^2-f^2\,m_2\,t_2\,w_{B,a}^2-f\,z\,w_{B,a}\,w_{B,B}-f^2\,z\,w_{B,a}\,w_{B,B}+2\,f^3\,z\,w_{B,a}\,w_{B,B}+2\,z\,m_1\,w_{B,a}\,w_{B,B}-$$
$$4\,f\,z\,m_1\,w_{B,a}\,w_{B,B}+2\,f^2\,z\,m_1\,w_{B,a}\,w_{B,B}-2\,z\,m_2\,w_{B,a}\,w_{B,B}+3\,f\,z\,m_2\,w_{B,a}\,w_{B,B}+f^2\,z\,m_2\,w_{B,a}\,w_{B,B}-$$
$$2\,f^3\,z\,m_2\,w_{B,a}\,w_{B,B}-f\,m_2\,t_2\,w_{B,a}\,w_{B,B}+f^2\,m_2\,t_2\,w_{B,a}\,w_{B,B}-f^3\,z\,w_{B,B}^2+2\,f\,z\,m_1\,w_{B,B}^2-2\,f^2\,z\,m_1\,w_{B,B}^2+$$
$$f\,(2\,c-3\,z+2\,t_2)\,w_{A,A}\,((-1+f)\,w_{B,a}-f\,w_{B,B})-(-1+f)\,z\,(-1+m_1)\,w_{A,B}\,((-1+f)\,w_{B,a}-f\,w_{B,B})\big)\big)+$$
$$((-1+f)\,w_{B,a}-f\,w_{B,B})\left(-(-1+f)^2\,(-1+m_2)^2\,(c-2\,z+t_2)\,w_{A,a}^2\,((-1+f)\,w_{B,a}-f\,w_{B,B})+\right.$$
$$(-1+f)\,(-1+m_2)\,w_{A,a}\left(-(-1+f)^2\,(c-z\,(2+m_2)-(-1+m_2)\,t_2)\,w_{B,a}^2+(-1+f)\,f\,(2\,c+z\,(-4+m_2)-(-2+m_2)\,t_2)\,w_{B,a}\,w_{B,B}-\right.$$
$$f^2\,(c-2\,z+t_2)\,w_{B,B}^2-f\,(2\,c-3\,z+2\,t_2)\,w_{A,A}\,((-1+f)\,w_{B,a}-f\,w_{B,B})\big)+$$
$$f\left(-f\,(c-z+t_2)\,w_{A,A}^2\,((-1+f)\,w_{B,a}-f\,w_{B,B})+(-1+f)\,z\,m_2\,w_{B,a}\,w_{B,B}\,((-1+f)\,w_{B,a}-f\,w_{B,B})+w_{A,A}\left(-(-1+f)^2\,(c-z\,(1+m_2)-\right.\right.$$
$$(-1+m_2)\,t_2)\,w_{B,a}^2+(-1+f)\,f\,(2\,c+z\,(-2+m_2)-(-2+m_2)\,t_2)\,w_{B,a}\,w_{B,B}-f^2\,(c-z+t_2)\,w_{B,B}^2\big)\big)\big)\big)\big)\Big/$$
$$(w_{a,a}\,(w_{a,a}+(-1+f+m_2-f\,m_2)\,w_{A,a}-f\,w_{A,A})\,(w_{a,a}+(-1+f)\,w_{B,a}-f\,w_{B,B})\,((-1+f)\,(-1+m_2)\,w_{A,a}+f\,w_{A,A}-w_{B,a}+f\,w_{B,a}-f\,w_{B,B}))$$
$$+O\left[\zeta^2\right]$$

Numerically evaluating the approximated eigenvalues for local stability such that $|\lambda_{(1)}| < 1$ and $|\lambda_{(2)}| < 1$ (Tables E17 and E18):

Table E17: Numerically evaluating the approximated eigenvalues to determine local stability as the paramutation rate varies across a given range

| $m_2$ | $\sim\lambda_{(1)}$ | $\sim\lambda_{(2)}$ | Local Stability |
|---|---|---|---|
| 0.055 | 0.916299 | 0.980631 | Stable |
| 0.105 | 0.916299 | 0.98063 | Stable |
| 0.155 | 0.916299 | 0.980628 | Stable |
| 0.205 | 0.916299 | 0.980626 | Stable |
| 0.255 | 0.916299 | 0.980625 | Stable |
| 0.305 | 0.916299 | 0.980623 | Stable |
| 0.355 | 0.916299 | 0.980621 | Stable |
| 0.405 | 0.916299 | 0.98062 | Stable |
| 0.455 | 0.916299 | 0.980618 | Stable |
| 0.505 | 0.916299 | 0.980616 | Stable |
| 0.555 | 0.916299 | 0.980615 | Stable |
| 0.605 | 0.916299 | 0.980613 | Stable |
| 0.655 | 0.916299 | 0.980612 | Stable |
| 0.705 | 0.916299 | 0.98061 | Stable |
| 0.755 | 0.916299 | 0.980608 | Stable |
| 0.805 | 0.916299 | 0.980607 | Stable |
| 0.855 | 0.916299 | 0.980605 | Stable |

Parameter values: $w_{A,A} = 1$, $w_{A,a} = 0.99$, $w_{A,B} = 0.985$, $w_{B,B} = 0.98$,
$w_{B,a} = 0.96$, $w_{a,a} = 0.97$, f = 0.1, m1 = 0.1, $t_1 = 10^{-5}$, $t_2 = 10^{-4}$,
$u = 10^{-9}$, $c = 10^{-9}$, $z = 10^{-9}$

Table E18: Numerically evaluating the approximated eigenvalues to determine local stability as the inbreeding coefficient varies across a given range

| f | $\sim\lambda_{(1)}$ | $\sim\lambda_{(2)}$ | Local Stability |
|---|---|---|---|
| 0.01 | 0.91016 | 0.979733 | Stable |
| 0.06 | 0.915717 | 0.980246 | Stable |
| 0.11 | 0.921274 | 0.98076 | Stable |
| 0.16 | 0.92683 | 0.981275 | Stable |
| 0.21 | 0.932385 | 0.981791 | Stable |
| 0.26 | 0.937939 | 0.982308 | Stable |
| 0.31 | 0.943491 | 0.982827 | Stable |
| 0.36 | 0.94904 | 0.983348 | Stable |
| 0.41 | 0.954586 | 0.983873 | Stable |
| 0.46 | 0.960125 | 0.984405 | Stable |
| 0.51 | 0.965653 | 0.984948 | Stable |
| 0.56 | 0.971157 | 0.985515 | Stable |
| 0.61 | 0.976596 | 0.986146 | Stable |
| 0.66 | 0.981706 | 0.987108 | Stable |
| 0.71 | 0.990276 | 0.984608 | Stable |
| 0.76 | 0.994071 | 0.986884 | Stable |
| 0.81 | 0.999428 | 0.987597 | Stable |

Parameter values: $w_{A,A} = 1$, $w_{A,a} = 0.99$, $w_{A,B} = 0.975$, $w_{B,B} = 0.97$,
$w_{B,a} = 0.96$, $w_{a,a} = 0.98$, m2 = 0.1, m1 = 0.1, $t_1 = 10^{-5}$, $t_2 = 10^{-4}$,
$u = 10^{-9}$, $c = 10^{-9}$, $z = 10^{-9}$
