# Supplementary material for: Deleterious mutation/epimutation–selection balance with and without inbreeding: a population (epi)genetics model
Source: Genetics. 2024 May 11;227(3):iyae080. doi: 10.1093/genetics/iyae080 (PMC11228854; doi:10.1093/genetics/iyae080)
Supplement: iyae080_Supplementary_Data [file iyae080_supplementary_data.zip › File_S3_GENETICS-2024-306923.pdf]

S3: Complete Dominance

Introduction

For this set of cases we assume complete dominance of the wild-type allele (A) for all genotypes where it is present, and complete dominance of the epiallele (*B*) over the deleterious allele (a) (see below).

Fitness assumptions for (epi) genotypes cases A – D

Allele/Epiallele	A	B	a
A	1	1	1
B	1	$1 - s_2$	$1 - s_2$
a	1	$1 - s_2$	$1 - s$

The fitness terms were substituted into the general recursion equations (File S1, eq. 2) for all cases A-D.

Case A: Random Mating (*f* = 0) and without Paramutation (m = 0)

Determining equilibria (following Appendix Section A1)

Assuming there is no inbreeding or paramutation (f = 0, m = 0), and making this substitution into (S1, eq. 2) gives the recursion equations

$$p_B' = -\Big((-1+c)\Big(-(-1+p_a+p_B)^2\, t_1 - (1-p_a-p_B)\,((p_a+p_B)\, t_1 - p_B\,(-1+t_2)) - p_B\,(-p_a\,(1-s_2) - p_B\,(1-s_2))\,(-1+t_2)\Big)\Big) \Big/ \Big(-\big((1-s)\, p_a^2\big) + 2\,(-p_a-p_B)\,(1-p_a-p_B) - (-1+p_a+p_B)^2 - 2\, p_a\, p_B\,(1-s_2) - p_B^2\,(1-s_2)\Big)$$

$$p_a' = \Big(-\big((1-s)\,(-1+z)\, p_a^2\big) - (-1+p_a+p_B)^2\,(-u+(-c+u)\, t_1) - p_B^2\,(1-s_2)\,(-c+(c-u)\, t_2) - p_a\, p_B\,(1-s_2)\,(-1-c+z+(c-u)\, t_2) + (1-p_a-p_B)\,(-p_a\,(-1-u+z+(-c+u)\, t_1) - p_B\,(-c-u-(c-u)\, t_1 - (-c+u)\, t_2))\Big) \Big/ \Big((1-s)\, p_a^2 - 2\,(-p_a-p_B)\,(1-p_a-p_B) + (-1+p_a+p_B)^2 + 2\, p_a\, p_B\,(1-s_2) + p_B^2\,(1-s_2)\Big)$$

The case assumptions and the approximation methods (Appendix section A1) did not allow for biologically valid equilibrium to be determined analytically. Therefore, numerical methods were used to determine equilibria, varying the key parameters across an empirically reasonable range.

Summary of Results

The numerical results indicate the presence of the epiallele at equilibrium ($\hat{p}_B > 0$) reduced $\hat{p}_a$ from the classic two-allele mutation-selection balance expectations by ~ 2 orders of magnitude across the range of parameter values explored (see figures A1-2). This is under the assumption that both forward and backward rates of spontaneous epimutation were relatively weak compared to selection but still significantly higher than mutation (s, $s_2 >> t_1$, $t_2 >> u$, c, z). The reduction in $\hat{p}_a$ may be due to heterozygous pairings of the deleterious allele and the epiallele occurring at the expense of heterozygous pairings with the wild-type allele, reducing the marginal fitness of the deleterious allele. Therefore, the presence of the epiallele reduces the amount of segregating deleterious genetic variation unlike with

incomplete dominance (see main text, case A, eq. 1).

$\hat{p}_B$ ranged from ~ 0.03 - 0.13 (Figures A1-2), indicating a significant amount of segregating deleterious epigenetic variation, largely due to the relative magnitude of 't_1' being higher than 'u'. This caused $\hat{p}_A$ to be significantly decreased from classic expectations (Crow and Kimura 2010). As t_2 reaches higher rates, such as occurs with unstable epialleles, we observe that the deleterious allele increases in frequency approaching that of classic mutation-selection balance, with significant decreases in the epiallele equilibrium frequency (figures A3), with a corresponding increase of $\hat{p}_A$ (not shown). Of note, the deleterious allele did not reach equilibrium in figure 3A (and was as assessed up to > 100, 000 generations).

Deleterious allele and epiallele frequency across forward spontaneous epimutation rate

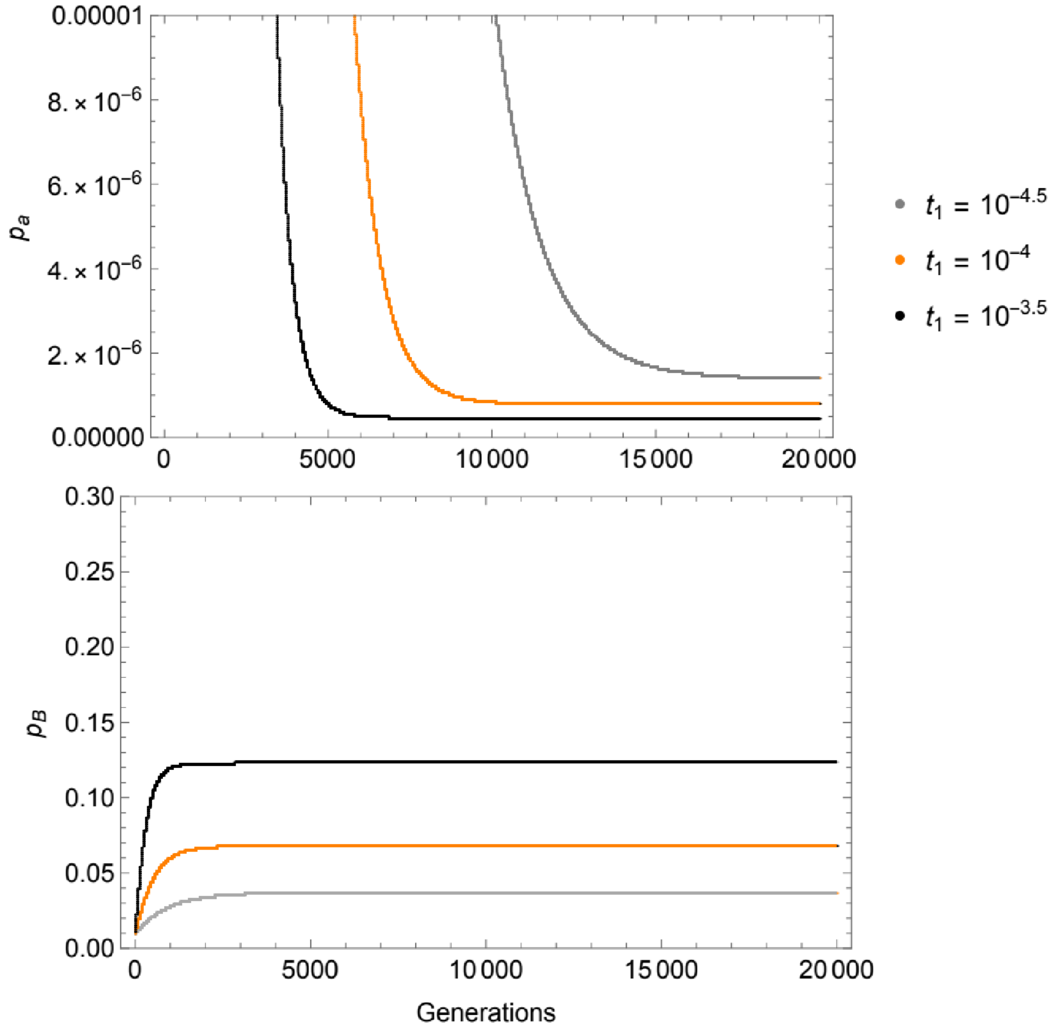

Figure A1 : Numerical simulations of the recursion equations showing the impacts of the forward spontaneous epimutation rate on equilibria as it increases going from grey to orange to black. Other parameter values were held constant: s = 0.02, s_2 = 0.02, t_2 = 10^-4, u = 10^-9, z = 10^-9, c = 10^-9.

Deleterious allele and epiallele frequency as a function of the reverse spontaneous epimutation rate (which is assumed to be small in magnitude)

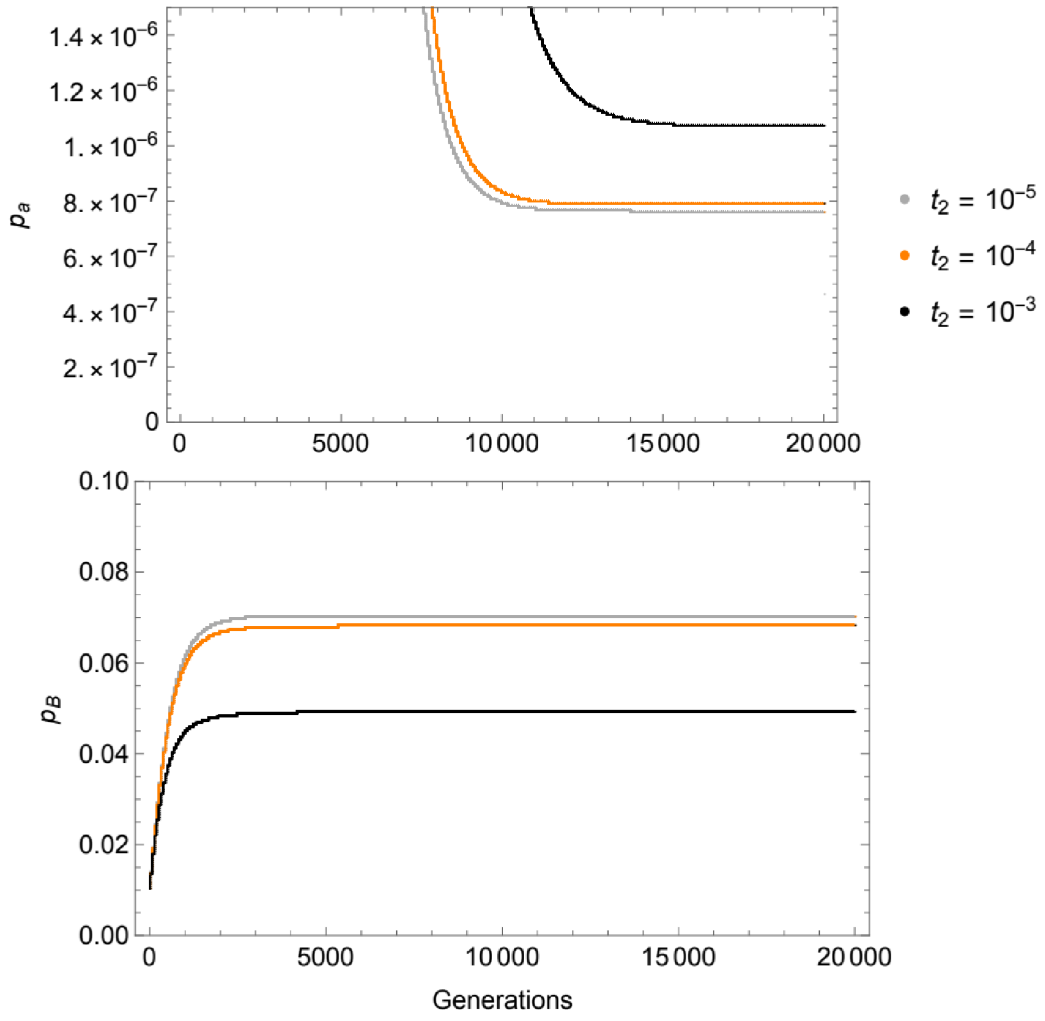

Figure A2: Numerical simulations of the recursion equations showing the impacts of the reverse spontaneous epimutation rate on equilibria as it increases going from grey to orange to black. Other parameter values were held constant: s = 0.02, s_2= 0.02, t_1 = 10^-4, u = 10^-9, z = 10^-9, c = 10^-9.

Deleterious allele and epiallele frequency as a function of the reverse spontaneous epimutation rate

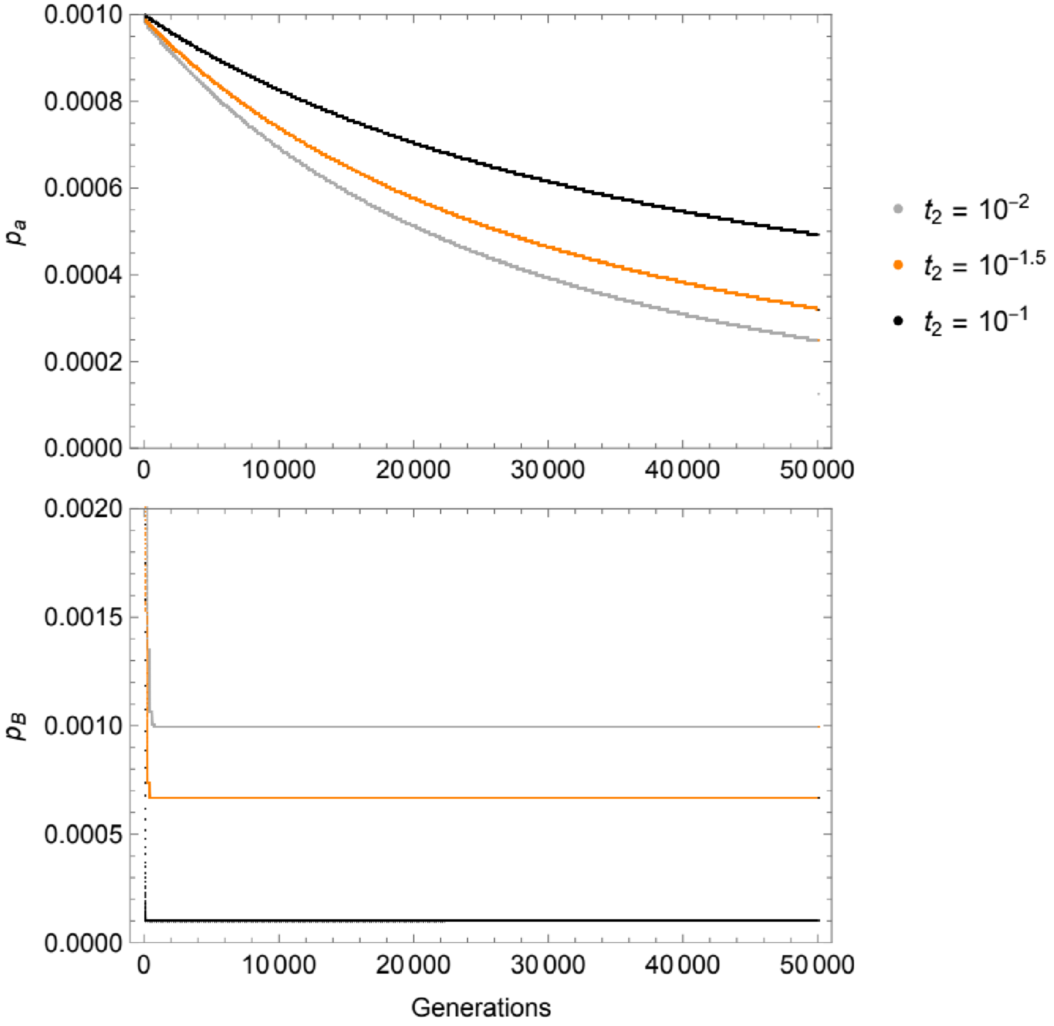

Figure A3: Numerical simulations of the recursion equations showing the impacts of the reverse spontaneous epimutation rate on equilibria as it increases going from grey to orange to black. Other parameter values were held constant: s = 0.02, s_2= 0.02, t_1 = 10^-5, u = 10^-9, z = 10^-9, c = 10^-9.

Case B: With Inbreeding (*f* > 0) and without Paramutation (m = 0)

Following the case assumptions, the recursion equations for the deleterious allele

and epiallele respectively are

$$p_B' = -\Big((-1+c)\big((-1+f)(-1+p_a+p_B)^2 t_1 - (1-p_a-p_B)((f+(-1+f)(-p_a-p_B))t_1 + (-1+f)p_B(-1+t_2)) - p_B((-1+f)p_a(1-s_2) + (-f+(-1+f)p_B)(1-s_2))(-1+t_2)\big)\Big) \Big/ \Big((1-s)p_a(-f+(-1+f)p_a) - (f+2(-1+f)(-p_a-p_B))(1-p_a-p_B) + (-1+f)(-1+p_a+p_B)^2 - p_B(f(1-s_2) - 2(-1+f)p_a(1-s_2)) + (-1+f)p_B^2(1-s_2)\Big)$$

$$p_a' = \Big((1-s)(-1+z)p_a(-f+(-1+f)p_a) + (-1+f)(-1+p_a+p_B)^2(-u+(-c+u)t_1) + (-1+f)p_B^2(1-s_2)(-c+(c-u)t_2) + p_B((-1+f)p_a(1-s_2)(-1-c+z+(c-u)t_2) + f(1-s_2)(c+(-c+u)t_2)) + (1-p_a-p_B)(f(u+(c-u)t_1) + (-1+f)(p_a(-1-u+z+(-c+u)t_1) + p_B(-c-u-(c-u)t_1-(-c+u)t_2)))\Big) \Big/ \Big((1-s)p_a(f-(-1+f)p_a) + (f+2(-1+f)(-p_a-p_B))(1-p_a-p_B) - (-1+f)(-1+p_a+p_B)^2 + p_B(f(1-s_2) - 2(-1+f)p_a(1-s_2)) - (-1+f)p_B^2(1-s_2)\Big)$$

Summary of Results

With inbreeding, one biologically valid equilibrium arose (eq. B1), and interestingly $\hat{p}_a$ was identical to classic expectations of two-allele mutation-selection balance (Hamilton 2009). This indicates the previous impact of the epiallele on the marginal fitness of the deleterious allele was negligible to the degree of approximation, likely as a direct result of inbreeding reducing the amount of B/a heterozygotes. The expression for $\hat{p}_B$ took on the same form as $\hat{p}_a$, whereby the major distinction is the greater expected magnitude of $\hat{p}_B$ as a result of the difference in spontaneous forward epimutation and the mutation rate (t_1 > u).

Overall, the additional segregating epigenetic variation at equilibrium decreased $\hat{p}_A$ below classic expectations. Intuitively, when t_2 is not assumed to be small in magnitude, $\hat{p}_B$ decreases and $\hat{p}_A$ increases (eq. B2). Also, interestingly, as t_2 increases, the combined effects of inbreeding and selection contribute less to the equilibrium frequency of the epiallele, likely due to the overall reduction in epialleles.

Determining equilibria (following Appendix Section A1)

Solving for equilibria, assuming spontaneous epimutation and mutation are of order ζ (see appendix section A1 for details). Inputting these substitutions ($t_1 \to t_1\zeta$, $t_2 \to t_2\zeta$, $u \to u\zeta$, $c \to c\zeta$, $z \to z\zeta$, $p_B \to p_{B,0} + p_{B,1}\zeta$, $p_a \to p_{a,0} + p_{a,1}\zeta$) into the equilibrium condition for the deleterious allele and epiallele frequencies, and taking the first order Taylor's approximation. Then solving for equilibrium terms up to first order. After substituting in the original parameters ($t\zeta \to t$, $u\zeta \to u$) and simplifying, the equilibrium approximation to first order is

$$\hat{p}_a \sim \frac{u}{f\,s} + O[\zeta^2]$$

$$\hat{p}_B \sim \frac{t_1}{f\,s_2} + O[\zeta^2]$$

$$\hat{p}_A \sim 1 - \frac{u}{f\,s} - \frac{t_1}{f\,s_2} + O[\zeta^2]$$

(Eq. B1)

Evaluating the accuracy of the analytical approximation in comparison to numerical equilibria solutions (Tables B1-B4).

Table B1: Evaluating the accuracy of the equilibrium approximation for the deleterious allele as the mutation rate of the wild-type allele increases from a lower to upper limit

u	~ $\hat{p}_a$ Analytical	$\hat{p}_a$ Numerical	Percentage Difference
$1.\times10^{-9}$	$1.\times10^{-7}$	$1.\times10^{-7}$	0.
1.1×10^{-8}	1.1×10^{-6}	1.1×10^{-6}	0.
2.1×10^{-8}	2.1×10^{-6}	2.1×10^{-6}	0.
3.1×10^{-8}	3.1×10^{-6}	3.1×10^{-6}	0.
4.1×10^{-8}	4.1×10^{-6}	4.1×10^{-6}	0.
5.1×10^{-8}	5.1×10^{-6}	5.1×10^{-6}	0.
6.1×10^{-8}	6.1×10^{-6}	6.1×10^{-6}	0.
7.1×10^{-8}	7.1×10^{-6}	7.1×10^{-6}	0.
8.1×10^{-8}	8.1×10^{-6}	8.1×10^{-6}	0.
9.1×10^{-8}	9.1×10^{-6}	9.1×10^{-6}	0.

Parameter values: $f = 0.5$, $s = 0.02$, $s_2 = 0.01$, $t_1 = 10$^−5, $t_2 = 10$^−4, $c = 10$^−9, $z = 10$^−9

Table B2: Evaluating the accuracy of the equilibrium approximation for the deleterious allele as the forward spontaneous epimutation rate increases from a lower to upper limit

t_1	~ $\hat{p}_B$ Analytical	$\hat{p}_B$ Numerical	Percentage Difference
0.00001	0.0004	0.0003983	0.425
0.00011	0.0044	0.0043639	0.820455
0.00021	0.0084	0.0082985	1.20833
0.00031	0.0124	0.0122029	1.58952
0.00041	0.0164	0.0160778	1.96463
0.00051	0.0204	0.0199238	2.33431
0.00061	0.0244	0.0237415	2.69877
0.00071	0.0284	0.0275317	3.05739
0.00081	0.0324	0.0312947	3.41142
0.00091	0.0364	0.0350313	3.76016
0.00101	0.0404	0.038742	4.10396

Parameter values: f = 0.5, s = 0.02, s_2 = 0.05, u = 10 ^ −9, t_2 = 10 ^ −4,
c = 10 ^ −9, z = 10 ^ −9

Table B3: Evaluating the accuracy of the equilibrium approximation for the deleterious allele as the inbreeding coefficient varies from a lower to upper limit

f	~ $\hat{p}_a$ Analytical	$\hat{p}_a$ Numerical	Percentage Difference
0.01	$5.\times 10^{-6}$	1.5×10^{-6}	70.
0.11	$5.\times 10^{-7}$	$4.\times 10^{-7}$	20.
0.21	$2.\times 10^{-7}$	$2.\times 10^{-7}$	0.
0.31	$2.\times 10^{-7}$	$2.\times 10^{-7}$	0.
0.41	$1.\times 10^{-7}$	$1.\times 10^{-7}$	0.
0.51	$1.\times 10^{-7}$	$1.\times 10^{-7}$	0.
0.61	$1.\times 10^{-7}$	$1.\times 10^{-7}$	0.
0.71	$1.\times 10^{-7}$	$1.\times 10^{-7}$	0.
0.81	$1.\times 10^{-7}$	$1.\times 10^{-7}$	0.
0.91	$1.\times 10^{-7}$	$1.\times 10^{-7}$	0.

Parameter values: s = 0.02, s_2 = 0.05, u = 10 ^ −9, t_1 = 10 ^ −5, t_2 = 10 ^ −4,
c = 10 ^ −9, z = 10 ^ −9

Table B4: Evaluating the accuracy of the equilibrium approximation for the epiallele as the inbreeding coefficient varies from a lower to upper limit

f	~ $\hat{p}_B$ Analytical	$\hat{p}_B$ Numerical	Percentage Difference
0.01	0.02	0.0093853	53.0735
0.11	0.0018182	0.0017612	3.13497
0.21	0.0009524	0.0009402	1.28097
0.31	0.0006452	0.0006402	0.774954
0.41	0.0004878	0.0004851	0.553506
0.51	0.0003922	0.0003905	0.433452
0.61	0.0003279	0.0003268	0.335468
0.71	0.0002817	0.0002809	0.28399
0.81	0.0002469	0.0002463	0.243013
0.91	0.0002198	0.0002193	0.22748

Parameter values: $s = 0.02$, $s_2 = 0.05$, $u = 10^{-9}$, $t_1 = 10^{-5}$, $t_2 = 10^{-4}$, $c = 10^{-9}$, $z = 10^{-9}$

Now, relaxing the assumption that reverse spontaneous epimutation is of order ζ.

One biologically valid equilibrium occurs, and approximated to first order is

$$\hat{p}_a \sim \frac{u}{f\,s} + O[\zeta^2]$$

$$\hat{p}_B \sim \frac{t_1}{f\,s_2\,(1 - t_2) + t_2} + O[\zeta^2]$$

$$\hat{p}_A \sim 1 - \frac{u}{f\,s} - \frac{t_1}{f\,s_2\,(1 - t_2) + t_2} + O[\zeta^2]$$

(Eq. B2)

Evaluating the accuracy of the analytical approximation in comparison to numerical equilibria solutions (Tables B5-B8).

Table B5: Evaluating the accuracy of the equilibrium approximation for the deleterious allele as the mutation rate of the wild-type allele increases from a lower to upper limit

u	~ $\hat{p}_a$ Analytical	$\hat{p}_a$ Numerical	Percentage Difference
$1.\times 10^{-9}$	$1.\times 10^{-7}$	$1.\times 10^{-7}$	0.
1.1×10^{-8}	1.1×10^{-6}	1.1×10^{-6}	0.
2.1×10^{-8}	2.1×10^{-6}	2.1×10^{-6}	0.
3.1×10^{-8}	3.1×10^{-6}	3.1×10^{-6}	0.
4.1×10^{-8}	4.1×10^{-6}	4.1×10^{-6}	0.
5.1×10^{-8}	5.1×10^{-6}	5.1×10^{-6}	0.
6.1×10^{-8}	6.1×10^{-6}	6.1×10^{-6}	0.
7.1×10^{-8}	7.1×10^{-6}	7.1×10^{-6}	0.
8.1×10^{-8}	8.1×10^{-6}	8.1×10^{-6}	0.
9.1×10^{-8}	9.1×10^{-6}	9.1×10^{-6}	0.

Parameter values: $f = 0.5$, $s = 0.02$, $s_2 = 0.01$, $t_1 = 10^{\wedge}-5$, $t_2 = 0.2$, $c = 10^{\wedge}-9$, $z = 10^{\wedge}-9$

Table B6: Evaluating the accuracy of the equilibrium approximation for the epiallele as the forward spontaneous epimutation rate increases from a lower to upper limit

t_1	~ $\hat{p}_B$ Analytical	$\hat{p}_B$ Numerical	Percentage Difference
0.00001	0.000049	0.000049	0.
0.00011	0.0005392	0.0005389	0.06
0.00021	0.0010294	0.0010284	0.1
0.00031	0.0015196	0.0015173	0.15
0.00041	0.0020098	0.0020058	0.2
0.00051	0.0025	0.0024938	0.25
0.00061	0.0029902	0.0029813	0.3
0.00071	0.0034804	0.0034684	0.34
0.00081	0.0039706	0.003955	0.39
0.00091	0.0044608	0.0044411	0.44
0.00101	0.004951	0.0049267	0.49

Parameter values: $f = 0.5$, $s = 0.02$, $s_2 = 0.01$, $t_1 = 10$^−5, $t_2 = 0.2$,
$c = 10$^−9, $z = 10$^−9

Table B7: Evaluating the accuracy of the equilibrium approximation for the deleterious allele as the inbreeding coefficient varies from a lower to upper limit

f	~ $\hat{p}_a$ Analytical	$\hat{p}_a$ Numerical	Percentage Difference
0.01	$5.\times 10^{-6}$	$5.\times 10^{-6}$	0.
0.11	$5.\times 10^{-7}$	$5.\times 10^{-7}$	0.
0.21	$2.\times 10^{-7}$	$2.\times 10^{-7}$	0.
0.31	$2.\times 10^{-7}$	$2.\times 10^{-7}$	0.
0.41	$1.\times 10^{-7}$	$1.\times 10^{-7}$	0.
0.51	$1.\times 10^{-7}$	$1.\times 10^{-7}$	0.
0.61	$1.\times 10^{-7}$	$1.\times 10^{-7}$	0.
0.71	$1.\times 10^{-7}$	$1.\times 10^{-7}$	0.
0.81	$1.\times 10^{-7}$	$1.\times 10^{-7}$	0.
0.91	$1.\times 10^{-7}$	$1.\times 10^{-7}$	0.

Parameter values: $s = 0.02$, $s_2 = 0.01$, $t_1 = 10^{-5}$, $t_2 = 0.2$, $u = 10^{-9}$,
$c = 10^{-9}$, $z = 10^{-9}$

Table B8: Evaluating the accuracy of the equilibrium approximation for the epiallele as the inbreeding coefficient varies from a lower to upper limit

f	~ $\hat{p}_B$ Analytical	$\hat{p}_B$ Numerical	Percentage Difference
0.01	0.00005	0.00005	0.
0.11	0.0000498	0.0000498	0.
0.21	0.0000496	0.0000496	0.
0.31	0.0000494	0.0000494	0.
0.41	0.0000492	0.0000492	0.
0.51	0.000049	0.000049	0.
0.61	0.0000488	0.0000488	0.
0.71	0.0000486	0.0000486	0.
0.81	0.0000484	0.0000484	0.
0.91	0.0000482	0.0000482	0.

Parameter values: $s = 0.02$, $s_2 = 0.01$, $t_1 = 10^{-5}$, $t_2 = 0.2$, $u = 10^{-9}$,
$c = 10^{-9}$, $z = 10^{-9}$

Local stability analysis (following appendix section A2):

The approximated eigenvalues corresponding with eq. B1 are

$$\lambda_{(1)} \sim 1 - f\,s + \frac{-\left(\left(2 - 3 f + f^2 s\right) u\right) + f\,(-1 + f\,s)\,z - \left(1 - 2 f + f^2 s\right) t_1}{f} + O\left[\zeta^2\right]$$

$$\lambda_{(2)} \sim 1 - f\,s_2 + \frac{s_2\left(c\,f^2 s - u + f\,u - f^2 s\,u - f^2 s\,t_1 + f^2 s\,t_2\right) - s\,((2 - 3 f)\,t_1 + f\,(c - u + t_2))}{f\,s} + O\left[\zeta^2\right]$$

Numerically evaluating the approximated eigenvalues for local stability such that $|\lambda_{(1)}| < 1$ and $|\lambda_{(2)}| < 1$ (Tables B9-B11).

Table B9: Numerically evaluating the approximated eigenvalues to determine local stability as the mutation rate of the wild-type allele varies across a given range

u	$\sim\lambda_1$	$\sim\lambda_2$	Local Stability
$1.\times 10^{-9}$	0.99	0.99489	Stable
1.1×10^{-8}	0.99	0.99489	Stable
2.1×10^{-8}	0.99	0.99489	Stable
3.1×10^{-8}	0.99	0.99489	Stable
4.1×10^{-8}	0.99	0.99489	Stable
5.1×10^{-8}	0.99	0.99489	Stable
6.1×10^{-8}	0.99	0.99489	Stable
7.1×10^{-8}	0.99	0.99489	Stable
8.1×10^{-8}	0.99	0.99489	Stable
9.1×10^{-8}	0.99	0.99489	Stable

Parameter values : $f = 0.5$, $s = 0.02$, $s_2 = 0.01$, $t_1 = 10^{\wedge}-5$, $t_2 = 10^{\wedge}-4$, $c = 10^{\wedge}-9$, $z = 10^{\wedge}-9$

Table B10: Numerically evaluating the approximated eigenvalues to determine local stability as the forward spontaneous epimutation rate varies across a given range

t_1	$\sim\lambda_1$	$\sim\lambda_2$	Local Stability
0.00001	0.99	0.974892	Stable
0.00011	0.989999	0.97479	Stable
0.00021	0.989998	0.974687	Stable
0.00031	0.989997	0.974585	Stable
0.00041	0.989996	0.974482	Stable
0.00051	0.989995	0.97438	Stable
0.00061	0.989994	0.974277	Stable
0.00071	0.989993	0.974175	Stable
0.00081	0.989992	0.974072	Stable
0.00091	0.989991	0.97397	Stable
0.00101	0.98999	0.973867	Stable

Parameter values : $f = 0.5$, $s = 0.02$, $s_2 = 0.05$,
$u = 10$^−9, $t_2 = 10$^−4, $c = 10$^−9, $z = 10$^−9

Table B11: Numerically evaluating the eigenvalue approximations to determine local stability as the inbreeding coefficient varies from near random mating to near fully inbreeding

f	$\sim\lambda_1$	$\sim\lambda_2$	Local Stability
0.01	0.99882	0.99743	Stable
0.11	0.997729	0.994249	Stable
0.21	0.995772	0.989336	Stable
0.31	0.993788	0.984367	Stable
0.41	0.991796	0.979383	Stable
0.51	0.9898	0.974393	Stable
0.61	0.987803	0.9694	Stable
0.71	0.985806	0.964405	Stable
0.81	0.983807	0.959409	Stable
0.91	0.981809	0.954412	Stable

Parameter values : $s = 0.02$, $s_2 = 0.05$, $u = 10$^−9,
$t_1 = 10$^−5, $t_2 = 10$^−4, $c = 10$^−9, $z = 10$^−9

The approximated eigenvalues corresponding with eq. B2 are

$$\lambda_{(1)} \sim 1 - f s + \frac{1}{f (f s_2 (-1 + t_2) - t_2)} \left(f s_2 \left(\left(1 - 2 f + f^2 s\right) t_1 - \left(\left(2 - 3 f + f^2 s\right) u + f (1 - f s) z\right) (-1 + t_2)\right) + \left(\left(2 - 3 f + f^2 s\right) u + f (1 - f s) z\right) t_2\right) + O\left[\zeta^2\right]$$

$$\lambda_{(2)} \sim (-1 + f s_2) (-1 + t_2) + \frac{1}{f s (f s_2 (-1 + t_2) - t_2)} \left(-f s_2^2 \left(f^2 s t_1 + \left(c f^2 s + \left(-1 + f - f^2 s\right) u\right) (-1 + t_2)\right) (-1 + t_2) + f s (t_1 - (c - u) (-1 + t_2)) t_2 + s_2 \left(f s t_1 (2 - 3 f + 2 (-1 + f) t_2) + (-1 + t_2) \left(f^2 s (-c + u) + \left(2 c f^2 s + \left(-1 + f - 2 f^2 s\right) u\right) t_2\right)\right)\right) + O\left[\zeta^2\right]$$

Numerically evaluating the approximated eigenvalues for local stability such that $|\lambda_{(1)}| < 1$ and $|\lambda_{(2)}| < 1$ (Tables B12-B14).

Table B12: Numerically evaluating the approximated eigenvalues to determine local stability as the mutation rate of the wild-type allele varies across a given range

u	$\sim\lambda_1$	$\sim\lambda_2$	Local Stability
$1. \times 10^{-9}$	0.99	0.79599	Stable
1.1×10^{-8}	0.99	0.79599	Stable
2.1×10^{-8}	0.99	0.79599	Stable
3.1×10^{-8}	0.99	0.79599	Stable
4.1×10^{-8}	0.99	0.79599	Stable
5.1×10^{-8}	0.99	0.79599	Stable
6.1×10^{-8}	0.99	0.79599	Stable
7.1×10^{-8}	0.99	0.79599	Stable
8.1×10^{-8}	0.99	0.79599	Stable
9.1×10^{-8}	0.99	0.79599	Stable

Parameter values : $f = 0.5$, $s = 0.02$, $s_2 = 0.01$,

$t_1 = 10$ ^ −5, $t_2 = 0.2$, c = 10 ^ −9, z = 10 ^ −9

Table B13: Numerically evaluating the approximated eigenvalues to determine local stability as the forward spontaneous epimutation rate varies across a given range

t_1	$\sim\lambda_1$	$\sim\lambda_2$	Local Stability
0.00001	0.99	0.79599	Stable
0.00011	0.99	0.795891	Stable
0.00021	0.99	0.795791	Stable
0.00031	0.99	0.795691	Stable
0.00041	0.99	0.795592	Stable
0.00051	0.99	0.795492	Stable
0.00061	0.99	0.795393	Stable
0.00071	0.99	0.795293	Stable
0.00081	0.99	0.795194	Stable
0.00091	0.99	0.795094	Stable
0.00101	0.99	0.794995	Stable

Parameter values : f = 0.5, s = 0.02, $s_2 = 0.01$,
u = 10 ^ −9, $t_2 = 0.2$, c = 10 ^ −9, z = 10 ^ −9

Table B14: Numerically evaluating the approximated eigenvalues to determine local stability as the inbreeding coefficient varies across a given range

f	$\sim\lambda_1$	$\sim\lambda_2$	Local Stability
0.01	0.999799	0.799909	Stable
0.11	0.9978	0.799109	Stable
0.21	0.9958	0.79831	Stable
0.31	0.9938	0.79751	Stable
0.41	0.9918	0.79671	Stable
0.51	0.9898	0.79591	Stable
0.61	0.9878	0.79511	Stable
0.71	0.9858	0.79431	Stable
0.81	0.9838	0.793511	Stable
0.91	0.9818	0.792711	Stable

Parameter values : s = 0.02, $s_2 = 0.01$, $t_1 = 10$ ^ −5,

$t_2 = 0.2$, $u = 10^{-9}$, $c = 10^{-9}$, $z = 10^{-9}$

Case C: Random Mating ($f = 0$) and With Paramutation ($m > 0$)

The recursion equations for this case are

$$p_B' = -\big((-1 + c)\,\big(-(-1 + p_a + p_B)^2\, t_1 - (1 - p_a - p_B)\,((p_a - (-1 + m)\, p_B)\, t_1 + p_B\, (-1 + m - 2\, m\, (1 - s_2))\, (-1 + t_2)) - p_B\, (-p_a\, (1 - s_2) - p_B\, (1 - s_2))\, (-1 + t_2)\big)\big) \Big/ \big(-((1 - s)\, p_a^2) - (-1 + p_a + p_B)^2 + 2\, (1 - p_a - p_B)\, (-p_a + p_B\, (-1 + m - m\, (1 - s_2))) - 2\, p_a\, p_B\, (1 - s_2) - p_B^2\, (1 - s_2)\big)$$

$$p_a' = \big(-((1 - s)\, (-1 + z)\, p_a^2) - (-1 + p_a + p_B)^2\, (-u + (-c + u)\, t_1) - p_B^2\, (1 - s_2)\, (-c + (c - u)\, t_2) - p_a\, p_B\, (1 - s_2)\, (-1 - c + z + (c - u)\, t_2) + (1 - p_a - p_B)\, (-p_a\, (-1 - u + z + (-c + u)\, t_1) - p_B\, (2\, m\, (1 - s_2)\, (-c + (c - u)\, t_2) + (-1 + m)\, (c + u + (c - u)\, t_1 + (-c + u)\, t_2)))\big) \Big/ \big((1 - s)\, p_a^2 + (-1 + p_a + p_B)^2 - 2\, (1 - p_a - p_B)\, (-p_a + p_B\, (-1 + m - m\, (1 - s_2))) + 2\, p_a\, p_B\, (1 - s_2) + p_B^2\, (1 - s_2)\big)$$

Similar to case A, the assumptions of this case did not give biologically valid equilibrium approximations for the relevant equilibria. In turn, numerical methods were used to obtain equilibria, with assessments of the impacts of varying the mutation rate, spontaneous epimutation rate, selection coefficients, and

paramutation rate on the equilibria. We present the key findings with figures.

Summary of Results

$\hat{p}_a$ was generally ~ 2 orders of magnitude below the two-allele mutation-selection balance expectations (Crow and Kimura 2010) with the condition that m was sufficiently greater than t_2. Similar to case A, this is likely a result of the epiallele being present at a sufficient frequency that it has non-negligible pairings with the deleterious allele, reducing the marginal fitness of the deleterious allele (figure C1). Therefore, this resulted in a reduction in the amount of segregating deleterious genetic variation. When t_2 is equal or greater than paramutation, the deleterious allele gets closer to classic mutation selection balance. Intuitively, $\hat{p}_B$ was at a high frequency when paramutation was significantly greater than t_2, but as t_2 became equal or greater than paramutation, there is a large drop in $\hat{p}_B$ to low frequencies with corresponding increases in the wild-type allele.

Interesting behaviour of the deleterious allele and epiallele frequencies over time is observed when paramutation is strong relative to selection and t_2, paralleling that of the incomplete dominance results. When s = s_2, the epiallele reaches a very high

equilibrium frequency as a result of paramutation, with a marginally higher frequency when s > s_2 (see figure C2). However, when s < s_2, cyclical behaviour of p_a and p_B is observed as the equilibrium is approached, and p_a reaches a high frequency and p_B reaches a low frequency for the given parameter values (see figure C2). Analogous to the incomplete dominance context, this may be a result of a twofold effect of paramutation, converting wild-type alleles at a high rate, maintaining p_A at low frequencies, while also inputting new epialleles into the most deleterious (epi)genotype. This allows a balance to unfold based on the differences in marginal fitness of the deleterious allele and epiallele, which favours the deleterious allele (when s < s_2).

Deleterious allele and epiallele frequency as a function of the reverse spontaneous epimutation rate

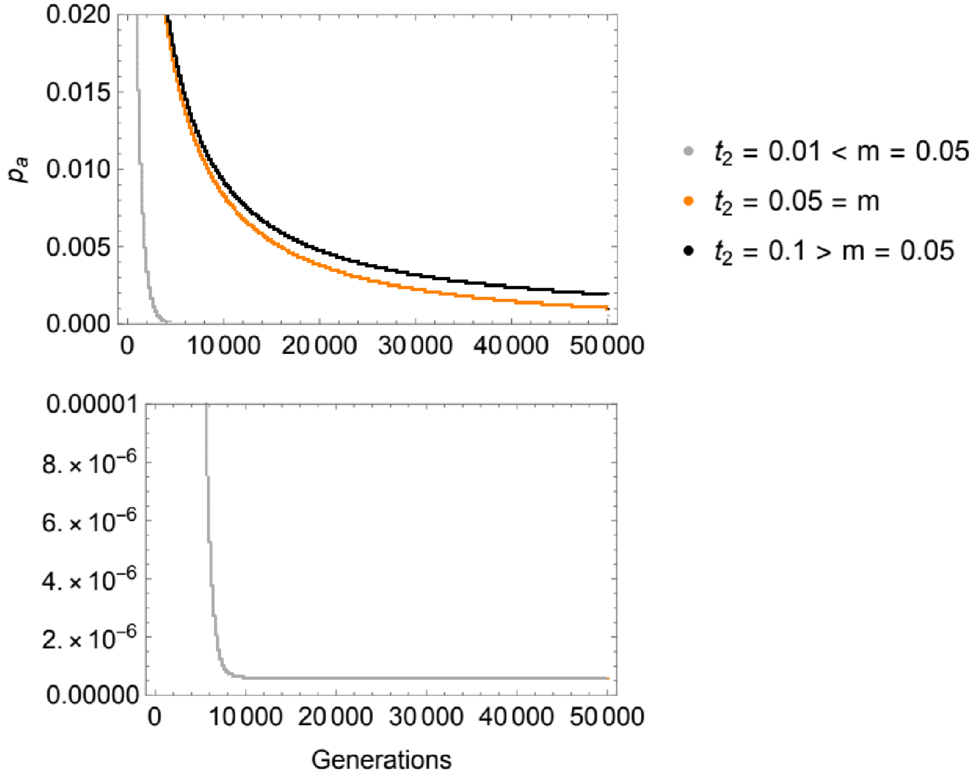

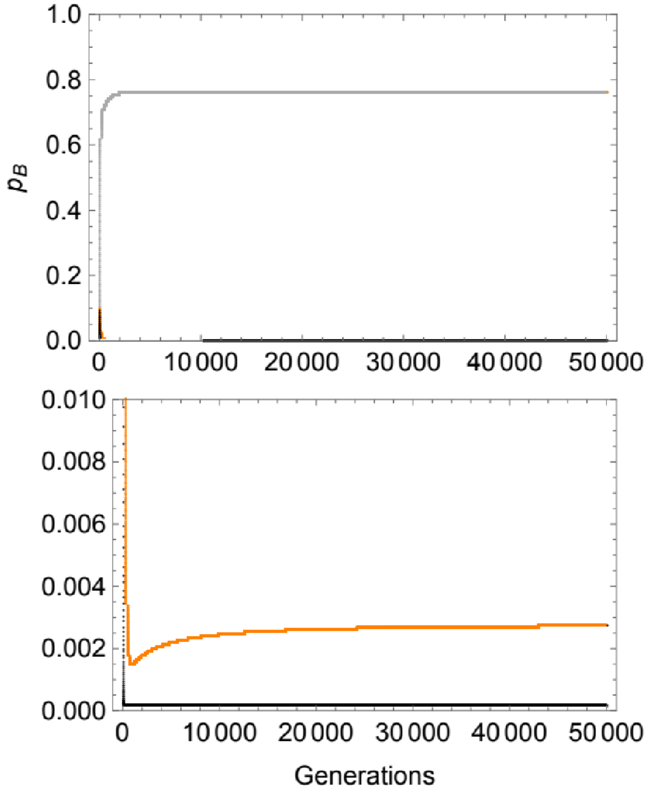

Figure C1: Numerical simulations of the recursion equations showing the impacts of the reverse spontaneous epimutation rate when it is weaker than the paramutation rate (gray), equal to the paramutation rate (orange), and greater than paramutation (black) on equilibria. Top panels shows the impacts on the deleterious allele with the second panel showing a magnification of the gray line. The 3rd panel demonstrates the effects on the epiallele frequency and the last panel has a magnified view of the black and orange lines. Other parameter values were held constant: s = 0.01, s_2= 0.01, t_1 = 10^-5, u = 10^-9, z = 10^-9, c =10^-9, m = 0.05.

Deleterious allele and epiallele frequency as a function of the selection coefficients

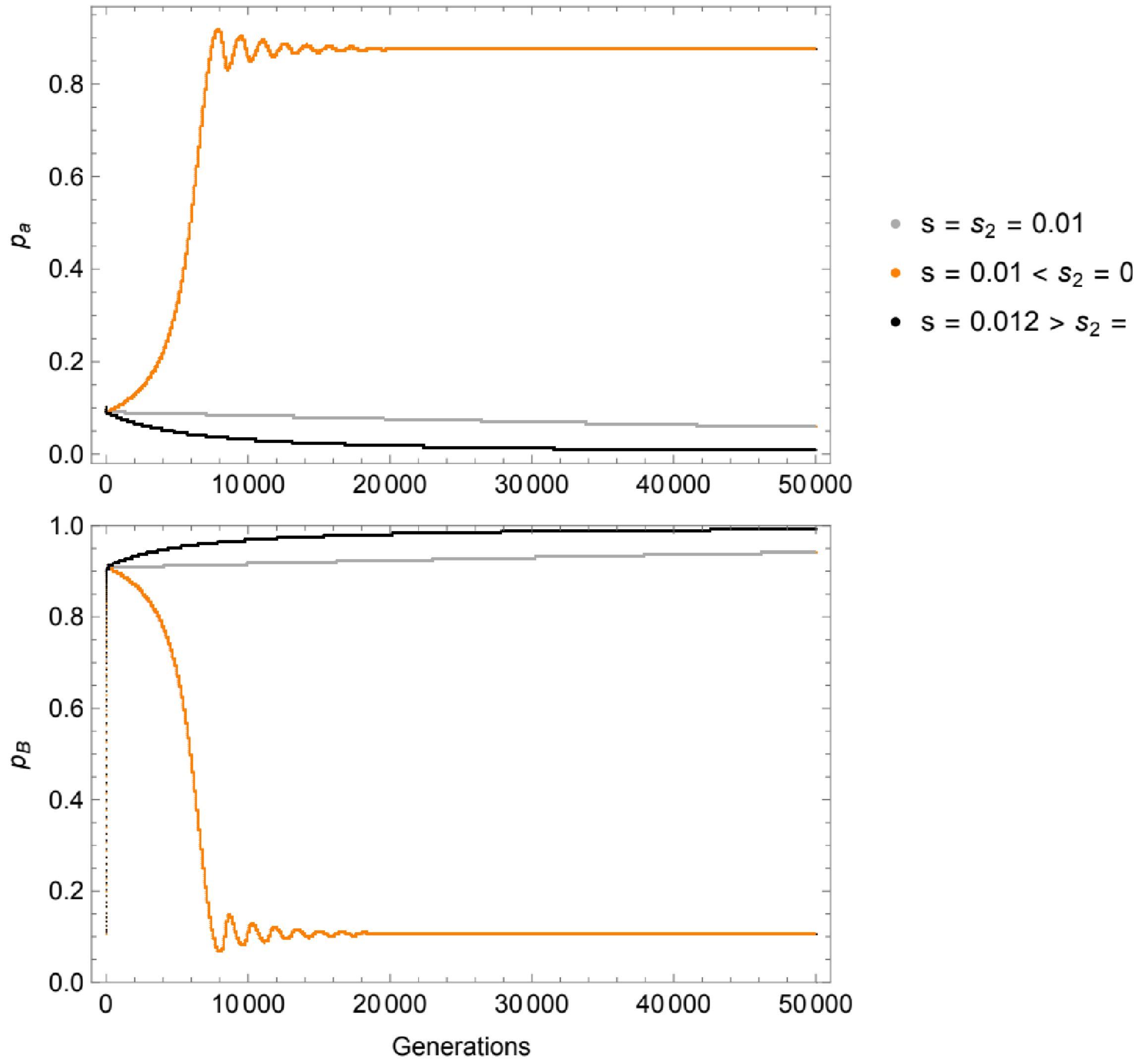

Figure C2 : Numerical simulations of the recursion equations showing the impacts for different fitness relationships. The epiallele: has relatively lower fitness (orange), equal fitness as the deleterious allele (gray) and higher relative fitness (black). Other parameter values were held constant: t_1 = 10^-5, t_2 = 10^-4, u = 10^-9, z = 10^-9, c = 10^-9, m = 0.1.

Case D: With Inbreeding ($f > 0$) and With Paramutation (m > 0)

The recursion equations for this case are

$$p_B{}' = -\Big((-1+c)\,\Big((-1+f)\,(-1+p_a+p_B)^2\,t_1 - (1-p_a-p_B)\,((f+(-1+f)\,(-p_a+(-1+m)\,p_B))\,t_1 - (-1+f)\,p_B\,(-1+m-2\,m\,(1-s_2))\,(-1+t_2)) - p_B\,((-1+f)\,p_a\,(1-s_2) + (-f+(-1+f)\,p_B)\,(1-s_2))\,(-1+t_2)\Big)\Big) \Big/ \Big((1-s)\,p_a\,(-f+(-1+f)\,p_a) + (-1+f)\,(-1+p_a+p_B)^2 - (1-p_a-p_B)\,(f+2\,(-1+f)\,(-p_a+p_B\,(-1+m-m\,(1-s_2)))) - p_B\,(f\,(1-s_2) - 2\,(-1+f)\,p_a\,(1-s_2)) + (-1+f)\,p_B^2\,(1-s_2)\Big)$$

$$p_a{}' = \Big((1-s)\,(-1+z)\,p_a\,(-f+(-1+f)\,p_a) + (-1+f)\,(-1+p_a+p_B)^2\,(-u+(-c+u)\,t_1) + (-1+f)\,p_B^2\,(1-s_2)\,(-c+(c-u)\,t_2) + p_B\,((-1+f)\,p_a\,(1-s_2)\,(-1-c+z+(c-u)\,t_2) + f\,(1-s_2)\,(c+(-c+u)\,t_2)) + (1-p_a-p_B)\,(f\,(u+(c-u)\,t_1) + (-1+f)\,(p_a\,(-1-u+z+(-c+u)\,t_1) + p_B\,(2\,m\,(1-s_2)\,(-c+(c-u)\,t_2) + (-1+m)\,(c+u+(c-u)\,t_1+(-c+u)\,t_2))))\Big) \Big/ \Big((1-s)\,p_a\,(f-(-1+f)\,p_a) - (-1+f)\,(-1+p_a+p_B)^2 + (1-p_a-p_B)\,(f+2\,(-1+f)\,(-p_a+p_B\,(-1+m-m\,(1-s_2)))) + p_B\,(f\,(1-s_2) - 2\,(-1+f)\,p_a\,(1-s_2)) - (-1+f)\,p_B^2\,(1-s_2)\Big)$$

Summary of Results

With inbreeding and paramutation ($m > 0$, $f > 0$), we identify four mutually exclusive equilibria, two of which were analytically interpretable and the other two were analyzed numerically. The equilibria were analogous to the results of the incomplete dominance context (case D) but with a key difference being one less equilibrium, which depends on incomplete dominance to in order to occur. Also, analogous to the incomplete dominance case, the effective rate of paramutation relative to

selection, and the relative fitnesses of the deleterious (epi)genotypes were the two major determining factors for which equilibrium arises in a biologically valid and locally stable form.

The analytical results demonstrate one equilibrium arose due to selection against the epiallele being of greater strength than the effective paramutation rate, resulting in the wild-type allele reaching the highest frequency (eq. D1). Specifically, the constraint $f s_2 > m(1 - f)(1 - 2s_2))$ was required for the equilibrium to occur in a biologically valid form. If the epiallele is very deleterious ($s_2 > 1/2$), the equilibrium will generally occur in a biologically valid form and interestingly, paramutation would reduce the amount of segregating deleterious epigenetic variation compared to when it is not occurring. Also, if inbreeding levels are sufficiently high, the equilibrium is always expected to arise as the effects of paramutation become small such that efficient selection in favour of the wild-type is the dominant force. At this equilibrium, when $s_2 < 1/2$, paramutation always increases $\hat{p}_B$ compared to when it is absent. The deleterious allele was at the classic expectation of mutation-selection balance with inbreeding (Hamilton 2009), suggesting no effect of paramutation on the amount of segregating deleterious genetic variation.

A second equilibrium arose whereby the epiallele was at the highest frequency as result of paramutation (eq. D2). For biological validity, this required the general constraints: $s > s_2$ and $m(1 - f) > s_2$ were met. Therefore, the deleterious allele must be more deleterious than the epiallele, and the effective paramutation must be greater than the strength of selection against the epiallele for this equilibrium to arise. The form of $\hat{p}_a$ was distinct from classic expectations of two-allele mutation-selection balance with inbreeding (Hamilton 2009), such that it was larger in magnitude as a result of the epiallele being at a high frequency. A possible reason for this is that it now mainly depends on the term $w_{BB} - w_{aa}$ instead of $w_{AA} - w_{aa}$ (classic expectation), whereby the first term is always smaller by assumption of the model, causing a relative increase of $\hat{p}_a$. Therefore, paramutation indirectly can cause increased segregating deleterious genetic variation in addition to the large degree of deleterious epigenetic variation at this equilibrium.

When t_2 is no longer assumed small in magnitude, an equilibrium occurs where the wild-type allele is at a higher frequency due to the higher reversion rate with a corresponding decrease in the epiallele (eq. D3). As noted in other cases, as t_2 increases, selection has smaller impacts on $\hat{p}_B$. The equilibrium where the epiallele can reach a very high frequency no longer arises. However, we again see that when m

is sufficiently strong relative to t_2, two equilibria mirroring that of the incomplete dominance context (case D) occurs (data not shown).

Determining equilibria (Appendix A1)

Assuming inbreeding is occurring and making substitutions assuming mutation and spontaneous epimutation are of order ζ, and substituting in the equilibrium terms up to the order of approximation,

$$\Big\{\Big((1-s)(-1+u)\,p_a\,(-f+(-1+f)\,p_a)-(-1+f)\,u\,(1-p_a-p_B)^2+(1-p_a-p_B)\,(f\,u+(-1+f)\,(-p_a+2\,u\,p_B\,(-1+m-m\,(1-s_2))))+p_B\,(f\,u\,(1-s_2)-(-1+f)\,p_a\,(1-s_2))-(-1+f)\,u\,p_B^2\,(1-s_2)\Big)\Big/\Big((1-s)\left(f\,p_a+(1-f)\,p_a^2\right)+f\,(1-p_a-p_B)+2\,(1-f)\,p_a\,(1-p_a-p_B)+(1-f)\,(1-p_a-p_B)^2+2\,(1-f)\,(1-m)\,(1-p_a-p_B)\,p_B+2\,(1-f)\,p_a\,p_B\,(1-s_2)+\left(f\,p_B+2\,(1-f)\,m\,(1-p_a-p_B)\,p_B+(1-f)\,p_B^2\right)(1-s_2)\Big)-p_a,$$
$$((1-u)\,(t\,(1-p_a-p_B)\,(f-(-1+f)\,(1-p_a-p_B)+(-1+f)\,(-p_a+(-1+m)\,p_B))+(1-t)\,p_B\,((-1+f)\,(1-p_a-p_B)\,(-1+m-2\,m\,(1-s_2))-(-1+f)\,p_a\,(1-s_2)+(f-(-1+f)\,p_B)\,(1-s_2))))\Big/\Big((1-s)\left(f\,p_a+(1-f)\,p_a^2\right)+f\,(1-p_a-p_B)+2\,(1-f)\,p_a\,(1-p_a-p_B)+(1-f)\,(1-p_a-p_B)^2+2\,(1-f)\,(1-m)\,(1-p_a-p_B)\,p_B+2\,(1-f)\,p_a\,p_B\,(1-s_2)+\left(f\,p_B+2\,(1-f)\,m\,(1-p_a-p_B)\,p_B+(1-f)\,p_B^2\right)(1-s_2)\Big)-p_B\Big\}\,/.$$
$$\{p_B=(p_{B,0}+p_{B,1}\,\zeta),\ p_a\to(p_{a,0}+p_{a,1}\,\zeta),\ t\to t\,\zeta,\ u\to u\,\zeta\};$$

After further analysis we identify two biologically valid, locally stable, and

analytically tractable equilibria, corresponding to the zero order terms

$\{p_{a,0} \to 0, p_{B,0} \to 0\}, \{p_{a,0} \to 0, p_{B,0} \to 1\}$.

After substituting in $\{p_{a,0} \to 0, p_{B,0} \to 0\}$ into first order terms, and solving for first order terms ($p_{a,1}$, $p_{B,1}$),

```
In[•]:= Solve[{-((-1 + f) u) + f u - pa,1 - (-1 + f) pa,1 + f (1 - s) pa,1 == 0,
          t - pB,1 + (f (1 - s2) + (-1 + f) (-1 - m + 2 m s2)) pB,1 == 0}, {pa,1, pB,1}]
```

Out[•]= $\left\{\left\{p_{a,1} \to \frac{u}{f\, s},\ p_{B,1} \to -\frac{t}{m - f\, m - f\, s_2 - 2\, m\, s_2 + 2\, f\, m\, s_2}\right\}\right\}$

Substituting in the original parameters and simplifying, the equilibrium approximation is

$$\hat{p}_a \sim \frac{u}{f\, s} + O[\zeta^2]$$

$$\hat{p}_B \sim \frac{t_1}{f\, s_2 - m\,(1 - f)\,(1 - 2\, s_2)} + O[\zeta^2]$$

$$\hat{p}_A \sim 1 - \frac{u}{f\, s} - \frac{t_1}{f\, s_2 - m\,(1 - f)\,(1 - 2\, s_2)} + O[\zeta^2]$$

(Eq. D1)

Evaluating the accuracy of the analytical approximation in comparison to numerical equilibria solutions (Tables D1-D6).

Table D1: Evaluating the accuracy of the equilibrium approximation for the deleterious allele as the paramutation rate increases from a lower to upper limit such that the equilibrium is biologically valid

m	~ $\hat{p}_a$ Analytical	$\hat{p}_a$ Numerical	Percentage Difference
0.001	$1.\times10^{-7}$	$1.\times10^{-7}$	0.
0.0015	$1.\times10^{-7}$	$1.\times10^{-7}$	0.
0.002	$1.\times10^{-7}$	$1.\times10^{-7}$	0.
0.0025	$1.\times10^{-7}$	$1.\times10^{-7}$	0.
0.003	$1.\times10^{-7}$	$1.\times10^{-7}$	0.
0.0035	$1.\times10^{-7}$	$1.\times10^{-7}$	0.
0.004	$1.\times10^{-7}$	$1.\times10^{-7}$	0.
0.0045	$1.\times10^{-7}$	$1.\times10^{-7}$	0.
0.005	$1.\times10^{-7}$	$1.\times10^{-7}$	0.

Parameter values: $f = 0.5$, $s = 0.02$, $s_2 = 0.01$, $t_1 = 10$^−5, $t_2 = 10$^−4,
$u = 10$^−9, $c = 10$^−9, $z = 10$^−9

Table D2: Evaluating the accuracy of the equilibrium approximation for the epiallele as the paramutation rate increases from a lower to upper limit such that the equilibrium is biologically valid

m	~ $\hat{p}_B$ Analytical	$\hat{p}_B$ Numerical	Percentage Difference
0.001	0.0022173	0.0021642	2.39
0.0015	0.0023447	0.0022851	2.54
0.002	0.0024876	0.0024202	2.71
0.0025	0.002649	0.0025722	2.9
0.003	0.0028329	0.0027446	3.12
0.0035	0.0030441	0.0029415	3.37
0.004	0.0032895	0.0031688	3.67
0.0045	0.0035778	0.0034339	4.02
0.005	0.0039216	0.0037472	4.45

Parameter values: $f = 0.5$, $s = 0.02$, $s_2 = 0.01$, $t_1 = 10^{-5}$, $t_2 = 10^{-4}$,
$u = 10^{-9}$, $c = 10^{-9}$, $z = 10^{-9}$

Table D3: Evaluating the accuracy of the equilibrium approximation for the deleterious allele as the forward spontaneous epimutation rate increases from a lower to upper limit

t_1	~ $\hat{p}_a$ Analytical	$\hat{p}_a$ Numerical	Percentage Difference
0.00001	$1.\times 10^{-7}$	$1.\times 10^{-7}$	0.
0.00011	$1.\times 10^{-7}$	$1.\times 10^{-7}$	0.
0.00021	$1.\times 10^{-7}$	$1.\times 10^{-7}$	0.
0.00031	$1.\times 10^{-7}$	$1.\times 10^{-7}$	0.
0.00041	$1.\times 10^{-7}$	$1.\times 10^{-7}$	0.
0.00051	$1.\times 10^{-7}$	$1.\times 10^{-7}$	0.
0.00061	$1.\times 10^{-7}$	$1.\times 10^{-7}$	0.
0.00071	$1.\times 10^{-7}$	$1.\times 10^{-7}$	0.
0.00081	$1.\times 10^{-7}$	$1.\times 10^{-7}$	0.
0.00091	$1.\times 10^{-7}$	$1.\times 10^{-7}$	0.
0.00101	$1.\times 10^{-7}$	$1.\times 10^{-7}$	0.

Parameter values: $m = 0.001$, $f = 0.5$, $s = 0.02$, $s_2 = 0.05$, $u = 10^{-9}$,
$t_2 = 10^{-4}$, $u = 10^{-9}$, $c = 10^{-9}$, $z = 10^{-9}$

Table D4: Evaluating the accuracy of the equilibrium approximation for the epiallele as the forward spontaneous epimutation rate increases from a lower to upper limit

t_1	~ $\hat{p}_B$ Analytical	$\hat{p}_B$ Numerical	Percentage Difference
0.00001	0.0004073	0.0004056	0.42
0.00011	0.0044807	0.0044429	0.84
0.00021	0.008554	0.0084476	1.24
0.00031	0.0126273	0.0124205	1.64
0.00041	0.0167006	0.0163623	2.03
0.00051	0.0207739	0.0202738	2.41
0.00061	0.0248473	0.0241557	2.78
0.00071	0.0289206	0.0280085	3.15
0.00081	0.0329939	0.031833	3.52
0.00091	0.0370672	0.0356297	3.88
0.00101	0.0411405	0.0393993	4.23

Parameter values: m = 0.001, f = 0.5, s = 0.02, s_2 = 0.05, u = 10 ^ −9,
t_2 = 10 ^ −4, u = 10 ^ −9, c = 10 ^ −9, z = 10 ^ −9

Table D5: Evaluating the accuracy of the equilibrium approximation for the deleterious allele as the inbreeding coefficient varies from a lower to upper limit whereby the equilibrium is biologically valid

f	~ $\hat{p}_a$ Analytical	$\hat{p}_a$ Numerical	Percentage Difference
0.4	$1.\times 10^{-7}$	$1.\times 10^{-7}$	0.
0.45	$1.\times 10^{-7}$	$1.\times 10^{-7}$	0.
0.5	$1.\times 10^{-7}$	$1.\times 10^{-7}$	0.
0.55	$1.\times 10^{-7}$	$1.\times 10^{-7}$	0.
0.6	$1.\times 10^{-7}$	$1.\times 10^{-7}$	0.
0.65	$1.\times 10^{-7}$	$1.\times 10^{-7}$	0.
0.7	$1.\times 10^{-7}$	$1.\times 10^{-7}$	0.
0.75	$1.\times 10^{-7}$	$1.\times 10^{-7}$	0.
0.8	$1.\times 10^{-7}$	$1.\times 10^{-7}$	0.
0.85	$1.\times 10^{-7}$	$1.\times 10^{-7}$	0.
0.9	$1.\times 10^{-7}$	$1.\times 10^{-7}$	0.

Parameter values: $m = 0.001$, $s = 0.02$, $s_2 = 0.01$, $u = 10$^−9, $t_1 = 10$^−5, $t_2 = 10$^−4, $c = 10$^−9, $z = 10$^−9

Table D6: Evaluating the accuracy of the equilibrium approximation for the epiallele as the inbreeding coefficient varies from a lower to upper limit whereby the equilibrium is biologically valid

f	~ $\hat{p}_B$ Analytical	$\hat{p}_B$ Numerical	Percentage Difference
0.4	0.0029308	0.0028337	3.31
0.45	0.0025246	0.0024544	2.78
0.5	0.0022173	0.0021642	2.39
0.55	0.0019767	0.0019352	2.1
0.6	0.0017832	0.0017499	1.87
0.65	0.0016242	0.0015969	1.68
0.7	0.0014912	0.0014685	1.52
0.75	0.0013784	0.0013591	1.4
0.8	0.0012814	0.0012649	1.29
0.85	0.0011972	0.0011829	1.19
0.9	0.0011233	0.0011108	1.11

Parameter values: m = 0.001, s = 0.02, s_2 = 0.01, u = 10 ^ −9, t_1 = 10 ^ −5, t_2 = 10 ^ −4, c = 10 ^ −9, z = 10 ^ −9

Next, substituting $\{p_{a,0} \to 0, p_{B,0} \to 1\}$ into first order terms, and solving for the corresponding first order terms,

In[•]:= Solve$\left[\left\{-p_{a,1}+\frac{(-1+f)\,u\,(-1+s_2)-f\,u\,(-1+s_2)+f\,(1-s)\,p_{a,1}+(-1+f)\,(-1+s_2)\,p_{a,1}}{1-s_2}==0,\right.\right.$

$-p_{B,1}+\frac{1}{1-s_2}\left((-1+f)\,(-1+s_2)\,p_{a,1}+(-1+f)\,(-1+s_2)\,p_{B,1}+(1-s_2)\,(-t+p_{B,1})-(-1+f)\,(-1-m+2\,m\,s_2)\,(p_{a,1}+p_{B,1})\right)+(1-s_2)\left(-\frac{u}{1-s_2}+\frac{1}{(1-s_2)^2}\left(f\,s\,p_{a,1}+2\,s_2\,p_{a,1}-2\,f\,s_2\,p_{a,1}-2\,m\,s_2\,p_{a,1}+2\,f\,m\,s_2\,p_{a,1}+2\,s_2\,p_{B,1}-f\,s_2\,p_{B,1}-2\,m\,s_2\,p_{B,1}+2\,f\,m\,s_2\,p_{B,1}\right)\right)==0\Big\},$

$\left.\{p_{a,1},\,p_{B,1}\}\right]$

Out[•]= $\left\{\left\{p_{a,1}\to-\frac{u\,(-1+s_2)}{f\,(s-s_2)},\;p_{B,1}\to-\frac{(-1+s_2)\,(-f\,s\,t-m\,u+f\,m\,u+f\,t\,s_2+u\,s_2)}{f\,(-m+f\,m+s_2)\,(-s+s_2)}\right\}\right\}$

Substituting in the original parameters and simplifying, the equilibrium approximation to first order is

$$\hat{p}_a\sim\frac{c\,(1-s_2)}{f\,(s-s_2)}+O[\zeta^2]$$

$$\hat{p}_B\sim 1-\frac{c\,(1-s_2)}{f\,(s-s_2)}-\frac{t_2\,(1-s_2)}{(1-f)\,m-s_2}+O[\zeta^2]$$

$$\hat{p}_A\sim\frac{t_2\,(1-s_2)}{(1-f)\,m-s_2}+O[\zeta^2]$$

(Eq. D2)

Evaluating the accuracy of the analytical approximation in comparison to numerical equilibria solutions (Tables D7-D12).

Table D7: Evaluating the accuracy of the equilibrium approximation for the deleterious allele as the paramutation rate increases from a lower to upper limit such that the equilibrium is biologically valid

m	~ $\hat{p}_a$ Analytical	$\hat{p}_a$ Numerical	Percentage Difference
0.06	$1.\times 10^{-6}$	$1.\times 10^{-6}$	0.
0.11	$1.\times 10^{-6}$	$1.\times 10^{-6}$	0.
0.16	$1.\times 10^{-6}$	$1.\times 10^{-6}$	0.
0.21	$1.\times 10^{-6}$	$1.\times 10^{-6}$	0.
0.26	$1.\times 10^{-6}$	$1.\times 10^{-6}$	0.
0.31	$1.\times 10^{-6}$	$1.\times 10^{-6}$	0.
0.36	$1.\times 10^{-6}$	$1.\times 10^{-6}$	0.
0.41	$1.\times 10^{-6}$	$1.\times 10^{-6}$	0.
0.46	$1.\times 10^{-6}$	$1.\times 10^{-6}$	0.
0.51	$1.\times 10^{-6}$	$1.\times 10^{-6}$	0.
0.56	$1.\times 10^{-6}$	$1.\times 10^{-6}$	0.
0.61	$1.\times 10^{-6}$	$1.\times 10^{-6}$	0.
0.66	$1.\times 10^{-6}$	$1.\times 10^{-6}$	0.
0.71	$1.\times 10^{-6}$	$1.\times 10^{-6}$	0.
0.76	$1.\times 10^{-6}$	$1.\times 10^{-6}$	0.
0.81	$1.\times 10^{-6}$	$1.\times 10^{-6}$	0.
0.86	$1.\times 10^{-6}$	$1.\times 10^{-6}$	0.
0.91	$1.\times 10^{-6}$	$1.\times 10^{-6}$	0.

Parameter values: $f = 0.1$, $s = 0.02$, $s_2 = 0.01$, $t_1 = 10$^−5, $t_2 = 10$^−4, $u = 10$^−9, $c = 10$^−9, $z = 10$^−9

Table D8: Evaluating the accuracy of the equilibrium approximation for the epiallele as the paramutation rate increases from a lower to upper limit such that the equilibrium is biologically valid

m	~ $\hat{p}_B$ Analytical	$\hat{p}_B$ Numerical	Percentage Difference
0.06	0.99775	0.997749	0.
0.11	0.998887	0.998887	0.
0.16	0.99926	0.99926	0.
0.21	0.999446	0.999446	0.
0.26	0.999557	0.999557	0.
0.31	0.999631	0.999631	0.
0.36	0.999684	0.999684	0.
0.41	0.999723	0.999723	0.
0.46	0.999754	0.999754	0.
0.51	0.999779	0.999779	0.
0.56	0.999799	0.999799	0.
0.61	0.999815	0.999815	0.
0.66	0.99983	0.99983	0.
0.71	0.999842	0.999842	0.
0.76	0.999852	0.999852	0.
0.81	0.999861	0.999861	0.
0.86	0.999869	0.999869	0.
0.91	0.999877	0.999877	0.

Parameter values: $f = 0.1$, $s = 0.02$, $s_2 = 0.01$, $t_1 = 10^{-5}$, $t_2 = 10^{-4}$, $u = 10^{-9}$, $c = 10^{-9}$, $z = 10^{-9}$

Table D9: Evaluating the accuracy of the equilibrium approximation for the deleterious allele as the forward spontaneous epimutation rate increases from a lower to upper limit

t_1	~ $\hat{p}_a$ Analytical	$\hat{p}_a$ Numerical	Percentage Difference
0.00001	$1.\times10^{-6}$	$1.\times10^{-6}$	0.
0.00011	$1.\times10^{-6}$	$1.\times10^{-6}$	0.
0.00021	$1.\times10^{-6}$	$1.\times10^{-6}$	0.
0.00031	$1.\times10^{-6}$	$1.\times10^{-6}$	0.
0.00041	$1.\times10^{-6}$	$1.\times10^{-6}$	0.
0.00051	$1.\times10^{-6}$	$1.\times10^{-6}$	0.
0.00061	$1.\times10^{-6}$	$1.\times10^{-6}$	0.
0.00071	$1.\times10^{-6}$	$1.\times10^{-6}$	0.
0.00081	$1.\times10^{-6}$	$1.\times10^{-6}$	0.
0.00091	$1.\times10^{-6}$	$1.\times10^{-6}$	0.
0.00101	$1.\times10^{-6}$	$1.\times10^{-6}$	0.

Parameter values: f = 0.1, m = 0.1, s = 0.02, s_2 = 0.01, u = 10^−9, t_2 = 10^−4,
u = 10^−9, c = 10^−9, z = 10^−9

Table D10: Evaluating the accuracy of the equilibrium approximation for the epiallele as the forward spontaneous epimutation rate increases from a lower to upper limit

t_1	~ $\hat{p}_B$ Analytical	$\hat{p}_B$ Numerical	Percentage Difference
0.00001	0.998762	0.998762	0.
0.00011	0.998762	0.998763	0.
0.00021	0.998762	0.998765	0.
0.00031	0.998762	0.998766	0.
0.00041	0.998762	0.998767	0.
0.00051	0.998762	0.998769	0.
0.00061	0.998762	0.99877	0.
0.00071	0.998762	0.998771	0.
0.00081	0.998762	0.998773	0.
0.00091	0.998762	0.998774	0.
0.00101	0.998762	0.998776	0.

Parameter values: $f = 0.1$, $m = 0.1$, $s = 0.02$, $s_2 = 0.01$, $u = 10^{-9}$, $t_2 = 10^{-4}$,
$u = 10^{-9}$, $c = 10^{-9}$, $z = 10^{-9}$

Table D11: Evaluating the accuracy of the equilibrium approximation for the deleterious allele as the inbreeding coefficient varies from a lower to upper limit such that the equilibrium is biologically valid

f	~ $\hat{p}_a$ Analytical	$\hat{p}_a$ Numerical	Percentage Difference
0.01	9.9×10^{-6}	9.1×10^{-6}	8.08
0.03	3.3×10^{-6}	3.2×10^{-6}	3.03
0.05	$2.\times10^{-6}$	1.9×10^{-6}	5.
0.07	1.4×10^{-6}	1.4×10^{-6}	0.
0.09	1.1×10^{-6}	1.1×10^{-6}	0.
0.11	$9.\times10^{-7}$	$9.\times10^{-7}$	0.
0.13	$8.\times10^{-7}$	$8.\times10^{-7}$	0.
0.15	$7.\times10^{-7}$	$7.\times10^{-7}$	0.
0.17	$6.\times10^{-7}$	$6.\times10^{-7}$	0.
0.19	$5.\times10^{-7}$	$5.\times10^{-7}$	0.
0.21	$5.\times10^{-7}$	$5.\times10^{-7}$	0.
0.23	$4.\times10^{-7}$	$4.\times10^{-7}$	0.
0.25	$4.\times10^{-7}$	$4.\times10^{-7}$	0.
0.27	$4.\times10^{-7}$	$4.\times10^{-7}$	0.
0.29	$3.\times10^{-7}$	$3.\times10^{-7}$	0.

Parameter values: $m = 0.1$, $s = 0.02$, $s_2 = 0.01$, $u = 10^{-9}$, $t_1 = 10^{-5}$,
$t_2 = 10^{-4}$, $u = 10^{-9}$, $c = 10^{-9}$, $z = 10^{-9}$

Table D12: Evaluating the accuracy of the equilibrium approximation for the epiallele as the inbreeding coefficient varies from a lower to upper limit such that the equilibrium is biologically valid

f	~ $\hat{p}_B$ Analytical	$\hat{p}_B$ Numerical	Percentage Difference
0.01	0.998878	0.998879	0.
0.03	0.998859	0.998859	0.
0.05	0.998833	0.998834	0.
0.07	0.998806	0.998806	0.
0.09	0.998777	0.998777	0.
0.11	0.998746	0.998746	0.
0.13	0.998714	0.998714	0.
0.15	0.998679	0.99868	0.
0.17	0.998643	0.998643	0.
0.19	0.998605	0.998605	0.
0.21	0.998565	0.998565	0.
0.23	0.998522	0.998522	0.
0.25	0.998477	0.998477	0.
0.27	0.998428	0.998429	0.
0.29	0.998377	0.998377	0.

Parameter values: m = 0.1, s = 0.02, s_2 = 0.01, u = 10^−9, t_1 = 10^−5, t_2 = 10^−4, u = 10^−9, c = 10^−9, z = 10^−9

Relaxing the assumption that the reverse spontaneous epimutation rate is $O(\zeta)$, one biologically valid and analytically tractable equilibrium approximation occurs, and approximated to first order is

$$\hat{p}_a \sim \frac{u}{f\, s} + O[\zeta^2]$$

$$\hat{p}_B \sim \frac{t_1}{t_2 - (1 - t_2)\,((1 - f)\,(1 - 2\, s_2) - f\, s_2)} + O[\zeta^2]$$

$$\hat{p}_A \sim 1 - \frac{u}{f\, s} - \frac{t_1}{t_2 - (1 - t_2)\left((1 - f)(1 - 2\, s_2) - f\, s_2\right)} + O[\zeta^2]$$

(Eq. D3)

Evaluating the accuracy of the analytical approximation in comparison to numerical equilibria solutions (Tables D12-D17).

Table D13: Evaluating the accuracy of the equilibrium approximation for the deleterious allele as the paramutation rate increases from a lower to upper limit such that the equilibrium is biologically valid

m	~ $\hat{p}_a$ Analytical	$\hat{p}_a$ Numerical	Percentage Difference
0.005	$5. \times 10^{-7}$	$5. \times 10^{-7}$	0.
0.025	$5. \times 10^{-7}$	$5. \times 10^{-7}$	0.
0.045	$5. \times 10^{-7}$	$5. \times 10^{-7}$	0.
0.065	$5. \times 10^{-7}$	$5. \times 10^{-7}$	0.
0.085	$5. \times 10^{-7}$	$5. \times 10^{-7}$	0.
0.105	$5. \times 10^{-7}$	$5. \times 10^{-7}$	0.
0.125	$5. \times 10^{-7}$	$5. \times 10^{-7}$	0.
0.145	$5. \times 10^{-7}$	$5. \times 10^{-7}$	0.
0.165	$5. \times 10^{-7}$	$5. \times 10^{-7}$	0.
0.185	$5. \times 10^{-7}$	$5. \times 10^{-7}$	0.
0.205	$5. \times 10^{-7}$	$5. \times 10^{-7}$	0.

Parameter values: $f = 0.1$, $s = 0.02$, $s_2 = 0.01$, $u = 10^{\wedge}{-9}$, $t_1 = 10^{\wedge}{-5}$, $t_2 = 0.2$, $u = 10^{\wedge}{-9}$, $c = 10^{\wedge}{-9}$, $z = 10^{\wedge}{-9}$

Table D14: Evaluating the accuracy of the equilibrium approximation for the epiallele as the paramutation rate increases from a lower to upper limit such that the equilibrium is biologically valid

m	~ $\hat{p}_B$ Analytical	$\hat{p}_B$ Numerical	Percentage Difference
0.005	0.0000507	0.0000507	0.
0.025	0.0000546	0.0000546	0.
0.045	0.0000592	0.0000592	0.
0.065	0.0000645	0.0000645	0.
0.085	0.000071	0.000071	0.
0.105	0.0000789	0.0000789	0.
0.125	0.0000888	0.0000888	0.
0.145	0.0001015	0.0001015	0.
0.165	0.0001185	0.0001185	0.
0.185	0.0001423	0.0001423	0.
0.205	0.0001781	0.000178	0.06

Parameter values: f = 0.1, s = 0.02, s_2 = 0.01, u = 10 ^ −9, t_1 = 10 ^ −5,
t_2 = 0.2, u = 10 ^ −9, c = 10 ^ −9, z = 10 ^ −9

Table D15: Evaluating the accuracy of the equilibrium approximation for the deleterious allele as the forward spontaneous epimutation rate increases from a lower to upper limit

t_1	~ $\hat{p}_a$ Analytical	$\hat{p}_a$ Numerical	Percentage Difference
0.00001	$5.\times10^{-7}$	$5.\times10^{-7}$	0.
0.00011	$5.\times10^{-7}$	$5.\times10^{-7}$	0.
0.00021	$5.\times10^{-7}$	$5.\times10^{-7}$	0.
0.00031	$5.\times10^{-7}$	$5.\times10^{-7}$	0.
0.00041	$5.\times10^{-7}$	$5.\times10^{-7}$	0.
0.00051	$5.\times10^{-7}$	$5.\times10^{-7}$	0.
0.00061	$5.\times10^{-7}$	$5.\times10^{-7}$	0.
0.00071	$5.\times10^{-7}$	$5.\times10^{-7}$	0.
0.00081	$5.\times10^{-7}$	$5.\times10^{-7}$	0.
0.00091	$5.\times10^{-7}$	$5.\times10^{-7}$	0.
0.00101	$5.\times10^{-7}$	$5.\times10^{-7}$	0.

Parameter values: m = 0.1, f = 0.1, s = 0.02, s_2 = 0.01, u = 10^−9, t_2 = 0.2, u = 10^−9, c = 10^−9, z = 10^−9

Table D16: Evaluating the accuracy of the equilibrium approximation for the epiallele as the forward spontaneous epimutation rate increases from a lower to upper limit

t_1	~ $\hat{p}_B$ Analytical	$\hat{p}_B$ Numerical	Percentage Difference
0.00001	0.0000768	0.0000768	0.
0.00011	0.0008446	0.0008434	0.14
0.00021	0.0016124	0.0016081	0.27
0.00031	0.0023802	0.0023708	0.39
0.00041	0.003148	0.0031316	0.52
0.00051	0.0039158	0.0038905	0.65
0.00061	0.0046837	0.0046475	0.77
0.00071	0.0054515	0.0054026	0.9
0.00081	0.0062193	0.0061558	1.02
0.00091	0.0069871	0.0069071	1.14
0.00101	0.0077549	0.0076565	1.27

Parameter values: $m = 0.1$, $f = 0.1$, $s = 0.02$, $s_2 = 0.01$, $u = 10^{\wedge}-9$, $t_2 = 0.2$,
$u = 10^{\wedge}-9$, $c = 10^{\wedge}-9$, $z = 10^{\wedge}-9$

Table D17: Evaluating the accuracy of the equilibrium approximation for the deleterious allele as the inbreeding coefficient varies from a lower to upper limit such that the equilibrium is biologically valid

f	$\sim \hat{p}_a$ Analytical	$\hat{p}_a$ Numerical	Percentage Difference
0.01	$5. \times 10^{-6}$	$5. \times 10^{-6}$	0.
0.06	$8. \times 10^{-7}$	$8. \times 10^{-7}$	0.
0.11	$5. \times 10^{-7}$	$5. \times 10^{-7}$	0.
0.16	$3. \times 10^{-7}$	$3. \times 10^{-7}$	0.
0.21	$2. \times 10^{-7}$	$2. \times 10^{-7}$	0.
0.26	$2. \times 10^{-7}$	$2. \times 10^{-7}$	0.
0.31	$2. \times 10^{-7}$	$2. \times 10^{-7}$	0.
0.36	$1. \times 10^{-7}$	$1. \times 10^{-7}$	0.
0.41	$1. \times 10^{-7}$	$1. \times 10^{-7}$	0.
0.46	$1. \times 10^{-7}$	$1. \times 10^{-7}$	0.

Parameter values: $m = 0.1$, $s = 0.02$, $s_2 = 0.01$, $u = 10^{\wedge}-9$, $t_1 = 10^{\wedge}-5$,
$t_2 = 0.2$, $u = 10^{\wedge}-9$, $c = 10^{\wedge}-9$, $z = 10^{\wedge}-9$

Table D18: Evaluating the accuracy of the equilibrium approximation for the epiallele as the inbreeding coefficient varies from a lower to upper limit such that the equilibrium is biologically valid

f	~ $\hat{p}_B$ Analytical	$\hat{p}_B$ Numerical	Percentage Difference
0.01	0.0000817	0.0000816	0.12
0.06	0.0000789	0.0000789	0.
0.11	0.0000763	0.0000763	0.
0.16	0.0000738	0.0000738	0.
0.21	0.0000716	0.0000716	0.
0.26	0.0000694	0.0000694	0.
0.31	0.0000674	0.0000674	0.
0.36	0.0000655	0.0000655	0.
0.41	0.0000637	0.0000637	0.
0.46	0.000062	0.000062	0.

Parameter values: $m = 0.1$, $s = 0.02$, $s_2 = 0.01$, $u = 10^{-9}$, $t_1 = 10^{-5}$, $t_2 = 0.2$, $u = 10^{-9}$, $c = 10^{-9}$, $z = 10^{-9}$

Local Stability Analysis (see Appendix A2 for details)

Calculating the Jacobian matrix for the recursion equations and then calculating the approximated characteristic equation. This is followed by substituting in explicit equilibrium terms

$(p_{a,0} \to 0,\ p_{B,0} \to 0,\ p_{a,1} \to \frac{u}{f\,s},\ p_{B,1} \to \frac{t}{f\,s_2 + m\,(1-f)\,(2\,s_2 - 1)})$, and solving for λ_0:

In[ ]:= $\text{Solve}[(1 - f\,s - \lambda_0)\,(f\,(1 - s_2) + (-1 + f)\,(-1 - m + 2\,m\,s_2) - \lambda_0) == 0,\ \lambda_0]$

Out[ ]= $\{\{\lambda_0 \to 1 - f\,s\},\ \{\lambda_0 \to 1 + m - f\,m - f\,s_2 - 2\,m\,s_2 + 2\,f\,m\,s_2\}\}$

Using both the λ_0 terms to solve for the corresponding λ_1 terms, and then back substituting the original parameters and simplifying. The approximated eigenvalues corresponding with eq. D1 are

$$\lambda_{(1)} \sim 1 - f\, s + \left((-1+f)\, m \left(\left(2 - 3 f + f^2 s\right) u + f\,(1 - f\, s)\, z\right) + s_2 \left(-\left((-2 m + f\,(-1 + 2 m))\left(\left(2 - 3 f + f^2 s\right) u + f\,(1 - f\, s)\, z\right)\right) + f\left(1 - 2 m + f^2 (s - 2 m\, s) + 2 f\,(-1 + m + m\, s)\right) t_1\right)\right) / (f\,(m - f\, m + (-2 m + f\,(-1 + 2 m))\, s_2)) + O\left[\zeta^2\right]$$

$$\lambda_{(2)} \sim 1 + m - f\, m - f\, s_2 - 2 m\, s_2 + 2 f\, m\, s_2 + \left(-\left((-1+f)\, m \left(-c\, f\, s - c\, f\, m\, s + c\, f^2 m\, s - m\, u + f\, m\, u + f\, s\, u + f\, m\, s\, u - f^2 m\, s\, u + f\,(1 + (-1+f)\, m)\, s\, t_1 + f\,(-1 + (-1+f)\, m)\, s\, t_2\right)\right) - (-2 m + f\,(-1 + 2 m))\, s_2^2 \left(-c\, f^2 s - 2 c\, f\, m\, s + 2 c\, f^2 m\, s + u - f\, u - 2 m\, u + 2 f\, m\, u + f^2 s\, u + 2 f\, m\, s\, u - 2 f^2 m\, s\, u + f\,(f + 2 m - 2 f\, m)\, s\, t_1 + f\,(-f - 2 m + 2 f\, m)\, s\, t_2\right) + s_2 \left(c\, f^2 s + 2 c\, f\, m\, s - 2 c\, f^3 m\, s + 4 c\, f\, m^2 s - 8 c\, f^2 m^2 s + 4 c\, f^3 m^2 s - m\, u + 3 f\, m\, u - 2 f^2 m\, u + 4 m^2 u - 8 f\, m^2 u + 4 f^2 m^2 u - f^2 s\, u - 2 f\, m\, s\, u + 2 f^3 m\, s\, u - 4 f\, m^2 s\, u + 8 f^2 m^2 s\, u - 4 f^3 m^2 s\, u + f\,(2 - 6 m + f\,(-3 + 6 m))\, s\, t_1 + f\left(f - 8 f\, m^2 + 2 f^2 m\,(-1 + 2 m) + 2 m\,(1 + 2 m)\right) s\, t_2\right)\right) / (f\, s\,(m - f\, m + (-2 m + f\,(-1 + 2 m))\, s_2)) + O\left[\zeta^2\right]$$

Numerically evaluating the approximated eigenvalues for local stability such that $|\lambda_{(1)}| < 1$ and $|\lambda_{(2)}| < 1$ (Tables D18-D20).

Table D19: Numerically evaluating the approximated eigenvalues to determine local stability as the paramutation rate varies across a given range

m	$\sim\lambda_{(1)}$	$\sim\lambda_{(2)}$	Stability
0.001	0.99	0.995378	Stable
0.0015	0.99	0.995622	Stable
0.002	0.99	0.995866	Stable
0.0025	0.99	0.996109	Stable
0.003	0.99	0.996352	Stable
0.0035	0.99	0.996595	Stable
0.004	0.99	0.996838	Stable
0.0045	0.99	0.99708	Stable
0.005	0.99	0.997321	Stable

Parameter values : $f = 0.5$, $s = 0.02$, $s_2 = 0.01$, $t_1 = 10^{-5}$, $t_2 = 10^{-4}$, $u = 10^{-9}$, $c = 10^{-9}$, $z = 10^{-9}$

Table D20: Numerically evaluating the approximated eigenvalues to determine local stability as the forward spontaneous epimutation rate varies across a given range

t_1	$\sim\lambda_{(1)}$	$\sim\lambda_{(2)}$	Stability
0.00001	0.99	0.975342	Stable
0.00011	0.989999	0.975236	Stable
0.00021	0.989998	0.97513	Stable
0.00031	0.989997	0.975024	Stable
0.00041	0.989997	0.974919	Stable
0.00051	0.989996	0.974813	Stable
0.00061	0.989995	0.974707	Stable
0.00071	0.989994	0.974601	Stable
0.00081	0.989993	0.974495	Stable
0.00091	0.989993	0.97439	Stable
0.00101	0.989992	0.974284	Stable

Parameter values : $m = 0.001$, $f = 0.5$, $s = 0.02$, $s_2 = 0.05$, $u = 10^{-9}$, $t_2 = 10^{-4}$, $u = 10^{-9}$, $c = 10^{-9}$, $z = 10^{-9}$

Table D21: Numerically evaluating the approximated eigenvalues to determine local stability as the inbreeding coefficient varies across a given range

f	$\sim\lambda_{(1)}$	$\sim\lambda_{(2)}$	Stability
0.4	0.991994	0.996463	Stable
0.45	0.990997	0.995922	Stable
0.5	0.99	0.995378	Stable
0.55	0.989002	0.994834	Stable
0.6	0.988003	0.994288	Stable
0.65	0.987005	0.993742	Stable
0.7	0.986006	0.993196	Stable
0.75	0.985007	0.992649	Stable
0.8	0.984008	0.992102	Stable
0.85	0.983008	0.991554	Stable
0.9	0.982009	0.991007	Stable

Parameter values : $m = 0.001$, $s = 0.02$, $s_2 = 0.01$,
$u = 10\wedge -9$, $t_1 = 10\wedge -5$, $t_2 = 10\wedge -4$, $c = 10\wedge -9$, $z = 10\wedge -9$

The approximated eigenvalues corresponding with eq. D2 are

$$\lambda_{(1)} \sim \frac{-1 + f\,s - (-1+f)\,s_2}{-1+s_2} + \frac{1}{f\,(-1+s_2)\,((-1+f)\,m+s_2)} \Big((-1+f)\,m\left(c\left(2-3\,f+f^2\,s\right)+f\,(1-f\,s)\,z\right) - (-1+f)\,s_2^2\,(c\,(-2+f)-f\,z+(-1+f)\,f\,(-1+2\,m)\,t_2) + s_2\left(c\left(-f^3\,m+2\,(1+m)-f\,(3+5\,m)+f^2\,(4\,m+s)\right)+f\left(1+(-1+f)^2\,m-f\,s\right)z + f\left(-1+2\,f\,s-f^2\,s+2\,(-1+f)\,m\,(-1+f\,s)\right)t_2\right)\Big) + O[\zeta^2]$$

$$\lambda_{(2)} \sim \frac{-1+m-f\,m}{-1+s_2} + \Big(s_2^2\,(-c\,f-c\,m+2\,c\,f\,m-c\,f^2\,m+f\,u-f\,m\,u+f^2\,m\,u+(f+(-1+f)\,f\,m)\,t_1 + f\left(-3+5\,m+2\,f^2\,(1-2\,m)\,m-4\,m^2+f\left(2-7\,m+8\,m^2\right)\right)t_2) + (-1+f)\,m\,\big(c\,m-c\,f\,m+c\,f\,s-c\,f\,m\,s+c\,f^2\,m\,s-f\,s\,u+f\,m\,s\,u-$$

$$f^2\,m\,s\,u - f\,(1 + (-1 + f)\,m)\,s\,t_1 + f\,(1 + m - f\,m)\,s\,t_2\big) +$$
$$s_2\,\big(c\,m - c\,f^2\,m + c\,m^2 - 3\,c\,f\,m^2 + 3\,c\,f^2\,m^2 - c\,f^3\,m^2 + c\,f\,s - c\,f\,m\,s +$$
$$c\,f^2\,m\,s - f\,m\,u + f^2\,m\,u + f\,m^2\,u - 2\,f^2\,m^2\,u + f^3\,m^2\,u - f\,s\,u +$$
$$f\,m\,s\,u - f^2\,m\,s\,u + f\,(1 + (-1 + f)\,m)\,((-1 + f)\,m - s)\,t_1 +$$
$$f\,\big((3 - 2\,f)\,s + (-1 + f)^2\,m^2\,(1 + 4\,s) - (-1 + f)\,m\,(1 - 5\,s + 2\,f\,s)\big)\,t_2\big)\big)\big/$$
$$(f\,(-1 + s_2)\,((-1 + f)\,m + s_2)\,(-s + s_2)) +$$
$$O[\zeta^2]$$

Numerically evaluating the approximated eigenvalues for local stability such that $|\lambda_{(1)}| < 1$ and $|\lambda_{(2)}| < 1$ (Tables D21-D23).

Table D22: Numerically evaluating the approximated eigenvalues to determine local stability as the paramutation rate varies across a given range

m	$\sim\lambda_{(1)}$	$\sim\lambda_{(2)}$	Stability
0.06	0.99897	0.955618	Stable
0.11	0.998981	0.910189	Stable
0.16	0.998985	0.864746	Stable
0.21	0.998986	0.819299	Stable
0.26	0.998988	0.773852	Stable
0.31	0.998988	0.728403	Stable
0.36	0.998989	0.682954	Stable
0.41	0.998989	0.637505	Stable
0.46	0.998989	0.592056	Stable
0.51	0.99899	0.546607	Stable
0.56	0.99899	0.501158	Stable
0.61	0.99899	0.455708	Stable
0.66	0.99899	0.410259	Stable
0.71	0.99899	0.364809	Stable
0.76	0.99899	0.31936	Stable
0.81	0.998991	0.27391	Stable
0.86	0.998991	0.228461	Stable
0.91	0.998991	0.183011	Stable

Parameter values : $f = 0.1$, $s = 0.02$, $s_2 = 0.01$, $t_1 = 10^{-5}$, $t_2 = 10^{-4}$, $u = 10^{-9}$, $c = 10^{-9}$, $z = 10^{-9}$

Table D23:Numerically evaluating the approximated eigenvalues to determine local stability as the forward spontaneous epimutation rate varies across a given range

t_1	$\sim\lambda_{(1)}$	$\sim\lambda_{(2)}$	Stability
0.00001	0.99898	0.919276	Stable
0.00011	0.99898	0.919184	Stable
0.00021	0.99898	0.919093	Stable
0.00031	0.99898	0.919001	Stable
0.00041	0.99898	0.918909	Stable
0.00051	0.99898	0.918817	Stable
0.00061	0.99898	0.918725	Stable
0.00071	0.99898	0.918633	Stable
0.00081	0.99898	0.918541	Stable
0.00091	0.99898	0.918449	Stable
0.00101	0.99898	0.918357	Stable

Parameter values : $f = 0.1$, $m = 0.1$, $s = 0.02$, $s_2 = 0.01$, $u = 10^{-9}$, $t_2 = 10^{-4}$, $u = 10^{-9}$, $c = 10^{-9}$, $z = 10^{-9}$

Table D24: Numerically evaluating the approximated eigenvalues to determine local stability as the inbreeding coefficient varies across a given range

f	$\sim\lambda_{(1)}$	$\sim\lambda_{(2)}$	Stability
0.01	0.99989	0.910188	Stable
0.03	0.999688	0.912207	Stable
0.05	0.999485	0.914227	Stable
0.07	0.999283	0.916247	Stable
0.09	0.999081	0.918266	Stable
0.11	0.998878	0.920286	Stable
0.13	0.998676	0.922306	Stable
0.15	0.998474	0.924326	Stable
0.17	0.998271	0.926346	Stable
0.19	0.998069	0.928366	Stable
0.21	0.997867	0.930386	Stable
0.23	0.997664	0.932406	Stable
0.25	0.997462	0.934425	Stable
0.27	0.997259	0.936445	Stable
0.29	0.997057	0.938465	Stable

Parameter values : $m = 0.1$, $s = 0.02$, $s_2 = 0.01$, $u = 10 \wedge -9$, $t_1 = 10 \wedge -5$, $t_2 = 10 \wedge -4$, $u = 10 \wedge -9$, $c = 10 \wedge -9$, $z = 10 \wedge -9$

The approximated eigenvalues corresponding with eq. D3 are

$$\lambda_{(1)} \sim 1 - f\, s + \left(-s_2 \left(f\left(1 - 2\, m + f^2\, (s - 2\, m\, s) + 2\, f\, (-1 + m + m\, s)\right) t_1 + (-2\, m + f\, (-1 + 2\, m))\left(\left(2 - 3\, f + f^2\, s\right) u + f\, (1 - f\, s)\, z\right)(-1 + t_2)\right) + \left(\left(2 - 3\, f + f^2\, s\right) u + f\, (1 - f\, s)\, z\right)(m - f\, m + (-1 + (-1 + f)\, m)\, t_2)\right) \Big/ (f\, ((-1 + f)\, m + (-2\, m + f\, (-1 + 2\, m))\, s_2\, (-1 + t_2) + (1 + m - f\, m)\, t_2)) + O\left[\zeta^2\right]$$

$$\lambda_{(2)} \sim -((1 + m - f\, m - f\, s_2 - 2\, m\, s_2 + 2\, f\, m\, s_2)\, (-1 + t_2)) + \Big((-2\, m + f\, (-1 + 2\, m))\, s_2^2 \Big(f\, (-2\, m + f\, (-1 + 2\, m))\, s\, t_1 + \Big(c\, f\, (-2\, m + f\, (-1 + 2\, m))\, s + \left(1 - 2\, m + f^2\, (s - 2\, m\, s) + f\, (-1 + 2\, m\, (1 + s))\right) u\Big)\, (-1 + t_2)\Big)\, (-1 + t_2) + (c\, f\, (-1 + (-1 + f)\, m)\, s + (f\, s - (-1 + f)\, m\, (-1 + f\, s))\, u)\, (-1 + t_2)\, (m - f\, m + (-1 + (-1 + f)\, m)\, t_2) -$$

$$f\,s\,t_1 \left(-((-1+f)\,m\,(1+(-1+f)\,m)) + \left(1+(-1+f)^2\,m^2\right) t_2\right) -$$
$$s_2 \left(f\,s\,t_1\,(2-6\,m+f\,(-3+6\,m)-2\,(-1+f)\,(-1+2\,m)\,t_2) +\right.$$
$$(-1+t_2)\left(-c\,f\left(f-8\,f\,m^2+2\,f^2\,m\,(-1+2\,m)+2\,m\,(1+2\,m)\right)s +\right.$$
$$\left(f^2\,s+4\,(-1+f)^2\,m^2\,(-1+f\,s)+m\left(1-3\,f+2\,f^2+2\,f\,s-2\,f^3\,s\right)\right)u +$$
$$\left(2\,c\,f\left(2\,m\,(1+m)+f^2\,m\,(-1+2\,m)-f\left(-1+m+4\,m^2\right)\right)s +\right.$$
$$\left(-1+m+4\,m^2+2\,f^3\,(1-2\,m)\,m\,s +\right.$$
$$f\left(1+m-4\,m\,s-4\,m^2\,(2+s)\right) +$$
$$\left.\left.\left.\left.2\,f^2\left(m\,(-1+s)-s+m^2\,(2+4\,s)\right)\right)u\right)t_2\right)\right)\Big) \Big/$$
$$(f\,s\,((-1+f)\,m+(-2\,m+f\,(-1+2\,m))\,s_2\,(-1+t_2)+(1+m-f\,m)\,t_2)) +$$
$$O[\zeta^2]$$

Numerically evaluating the approximated eigenvalues for local stability such that

$|\lambda_{(1)}| < 1$ and $|\lambda_{(2)}| < 1$ (Tables D24-D26).

Table D25: Numerically evaluating the approximated eigenvalues to determine local stability as the paramutation rate varies across a given range

m	$\sim\lambda_{(1)}$	$\sim\lambda_{(2)}$	Stability
0.005	0.998	0.802717	Stable
0.025	0.998	0.816827	Stable
0.045	0.998	0.830937	Stable
0.065	0.998	0.845047	Stable
0.085	0.998	0.859156	Stable
0.105	0.997999	0.873265	Stable
0.125	0.997999	0.887372	Stable
0.145	0.997999	0.901479	Stable
0.165	0.997999	0.915584	Stable
0.185	0.997999	0.929686	Stable
0.205	0.997999	0.943784	Stable

Parameter values : $f = 0.1$, $s = 0.02$, $s_2 = 0.01$, $u = 10\text{^}-9$,
$t_1 = 10\text{^}-5$, $t_2 = 0.2$, $u = 10\text{^}-9$, $c = 10\text{^}-9$, $z = 10\text{^}-9$

Table D26: Numerically evaluating the approximated eigenvalues to determine local stability as the forward spontaneous epimutation rate varies across a given range

t_1	$\sim\lambda_{(1)}$	$\sim\lambda_{(2)}$	Stability
0.00001	0.998	0.869738	Stable
0.00011	0.997995	0.869513	Stable
0.00021	0.99799	0.869289	Stable
0.00031	0.997985	0.869064	Stable
0.00041	0.99798	0.86884	Stable
0.00051	0.997976	0.868616	Stable
0.00061	0.997971	0.868391	Stable
0.00071	0.997966	0.868167	Stable
0.00081	0.997961	0.867942	Stable
0.00091	0.997957	0.867718	Stable
0.00101	0.997952	0.867494	Stable

Parameter values : $m = 0.1$, $f = 0.1$, $s = 0.02$, $s_2 = 0.01$,
$u = 10\,^\wedge{-9}$, $t_2 = 0.2$, $u = 10\,^\wedge{-9}$, $c = 10\,^\wedge{-9}$, $z = 10\,^\wedge{-9}$

Table D27: Numerically evaluating the approximated eigenvalues to determine local stability as the inbreeding coefficient varies across a given range

f	$\sim\lambda_{(1)}$	$\sim\lambda_{(2)}$	Stability
0.01	0.999799	0.877511	Stable
0.06	0.998799	0.873193	Stable
0.11	0.9978	0.868874	Stable
0.16	0.9968	0.864555	Stable
0.21	0.9958	0.860236	Stable
0.26	0.9948	0.855917	Stable
0.31	0.9938	0.851598	Stable
0.36	0.9928	0.847279	Stable
0.41	0.9918	0.84296	Stable
0.46	0.9908	0.83864	Stable

Parameter values : $m = 0.1$, $s = 0.02$, $s_2 = 0.01$, $u = 10\,^\wedge{-9}$,
$t_1 = 10\,^\wedge{-5}$, $t_2 = 0.2$, $u = 10\,^\wedge{-9}$, $c = 10\,^\wedge{-9}$, $z = 10\,^\wedge{-9}$
