# Supplementary material for: Deleterious mutation/epimutation–selection balance with and without inbreeding: a population (epi)genetics model
Source: Genetics. 2024 May 11;227(3):iyae080. doi: 10.1093/genetics/iyae080 (PMC11228854; doi:10.1093/genetics/iyae080)
Supplement: iyae080_Supplementary_Data [file iyae080_supplementary_data.zip › Supplemental_Material_Legend_GENETICS-2024-306923.docx]

**Supplemental Material**

**File S1. Derivation of recursion equations for model.** This file goes through the detailed analytical derivation of the recursion equations used for the model.

**File S2. Incomplete Dominance.** This file goes through the detailed calculations and analysis corresponding to the main text with supporting figures.

**File S3. Complete Dominance.** This file demonstrates the results of the complete dominance context with detailed calculations and analysis with supporting figures.
